# S100a9 lactylation triggers neutrophil trafficking and cardiac inflammation in myocardial ischemia/reperfusion injury

**DOI:** 10.1172/JCI194664

**Published:** 2025-10-09

**Authors:** Xiaoqi Wang, Xiangyu Yan, Ge Mang, Yujia Chen, Shuang Liu, Jiayu Sui, Zhonghua Tong, Penghe Wang, Jingxuan Cui, Qiannan Yang, Yafei Zhang, Dongni Wang, Ping Sun, Weijun Song, Zexi Jin, Ming Shi, Peng Zhao, Jia Yang, Mingyang Liu, Naixin Wang, Tao Chen, Yong Ji, Bo Yu, Maomao Zhang

**Affiliations:** 1 Department of Cardiology, the Second Affiliated Hospital of Harbin Medical University, Harbin, Heilongjiang Province, China.; 2 The Key Laboratory of Myocardial Ischemia, Harbin Medical University, Ministry of Education, Harbin, Heilongjiang Province, China.; 3 Department of Cardiology, Beijing Anzhen Hospital, Capital Medical University, Beijing, China.; 4 Department of Ultrasound (P.S.), the Second Affiliated Hospital of Harbin Medical University, Harbin, Heilongjiang Province, China.; 5 School of Life Science and Technology, Harbin Institute of Technology, Harbin, China.; 6 Harbin Medical University, Harbin, China.; 7 State Key Laboratory of Frigid Zone Cardiovascular Disease, Harbin, Heilongjiang, China.

**Keywords:** Cardiology, Immunology, Cardiovascular disease, Cell migration/adhesion, Epigenetics

## Abstract

Lactylation, a posttranslational modification derived from glycolysis, plays a pivotal role in ischemic heart disease. Neutrophils are predominantly glycolytic cells that trigger intensive inflammation of myocardial ischemia/reperfusion (MI/R). However, whether lactylation regulates neutrophil function during MI/R remains unknown. We applied lactyl proteomics analysis and found that S100a9 was lactylated at lysine 26 (S100a9K26la) in neutrophils, with elevated levels observed in both patients with acute myocardial infarction (AMI) and MI/R model mice. We demonstrated that S100a9K26la drove the development of MI/R using mutant knockin mice. Mechanistically, lactylated S100a9 translocated to the nucleus of neutrophils, where it bound to the promoters of migration-related genes, thereby enhancing their transcription as a coactivator and promoting neutrophil migration and cardiac recruitment. Additionally, lactylated S100a9 was released during neutrophil extracellular trap (NET) formation, leading to cardiomyocyte death by disrupting mitochondrial function. The enzyme dihydrolipoyllysine-residue acetyltransferase (DLAT) was identified as the lactyltransferase facilitating neutrophil S100a9K26la following MI/R, a process that could be restrained by α-lipoic acid. Consistently, we found that targeting the DLAT/S100a9K26la axis suppressed neutrophil burden and improved cardiac function following MI/R. In patients with AMI, elevated S100a9K26la levels in plasma were positively correlated with cardiac death. These findings highlight S100a9 lactylation as a potential therapeutic target for MI/R and as a promising biomarker for evaluating poor MI/R outcomes.

## Abstract 2

This study identify DLAT-catalyzed S100a9 lactylation as a critical signaling that trigers neutrophil trafficking and acute inflammation burden and cardiac dysfunction post-MI/R.

## Graphical abstract

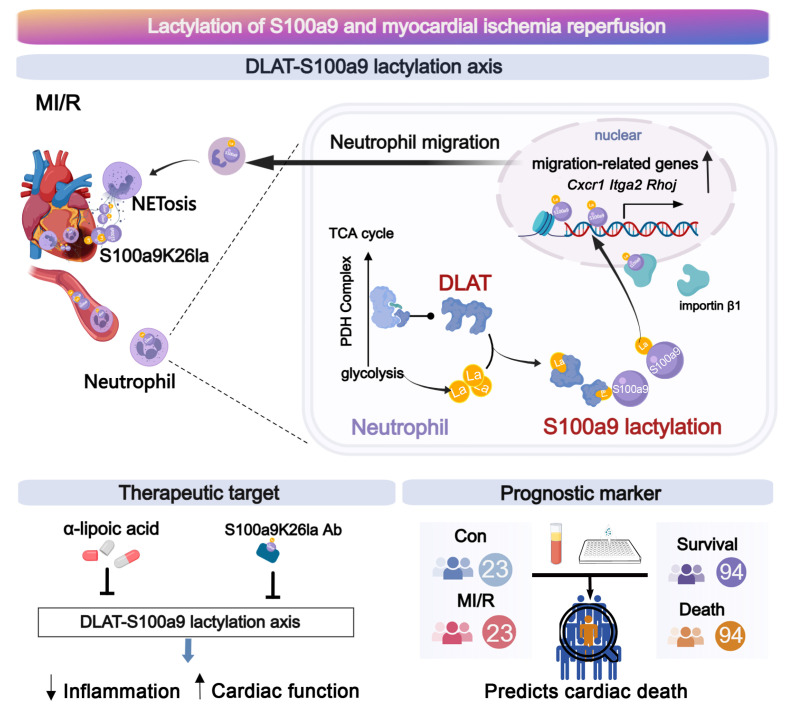

## Introduction

Reperfusion therapies can effectively restore blood flow to the ischemic myocardium in time, yet mortality rates remain high because of additional heart damage caused by reperfusion injury (1, 2). As sentinels of inflammation, neutrophils are mobilized and recruited to the infarcted heart. A high neutrophil count is a causal risk factor for ischemic heart disease (3). Prompt mitigation of the neutrophil burden may prevent adverse remodeling and improve cardiac outcomes (4). However, effective pharmacological treatments targeting acute neutrophil recruitment are still unavailable.

The significance of epigenetic modifiers and their genetic manipulation in influencing neutrophil behavior have been increasingly recognized (5–7). Neutrophil metabolism primarily relies on glycolysis under steady-state conditions and undergoes reprogramming to increase glycolysis during acute inflammatory attacks as previously reported (8, 9). This metabolic shift produces lactate (10), which serves as a precursor to stimulate lactylation (11).

Protein lactylation, as a form of epigenetic modification (11), appears to have some substantial effects in regulating cellular functions in various cardiovascular diseases, including atherosclerosis, heart failure, cardiac fibrosis, and myocardial infarction (12–15). Our previous study suggested that histone lactylation directly boosts the reparative transcriptional response in monocytes following MI (13). However, there is a lack of studies investigating the distribution and role of overall protein lactylation in neutrophils after myocardial ischemia reperfusion (MI/R). Through proteomics analysis, we identified the alarmin protein S100a9 lysine 26 (S100a9K26) as the primary modification target of early, remotely elevated neutrophil lactylation following MI/R. S100a8/a9 plays a critical role in inducing robust inflammation upon release (16). However, the function of lactylated S100a9 in neutrophils after MI/R remains unclear.

In this study, we provide, to our knowledge, the first evidence that the elevation of S100a9 lactylation aggravates cardiac dysfunction after MI/R. We identified dihydrolipoyllysine-residue acetyltransferase (DLAT), a component of the pyruvate dehydrogenase (PDH) complex (17), as a lactyltransferase that directly catalyzes lactylation of S100a9. Our findings highlight lactylation-related signaling as a potential therapeutic target for MI/R.

## Results

### S100a9 lactylation is elevated in neutrophils after MI/R.

Among several posttranslational modifications including crotonylation, phosphorylation, acetylation, and lactylation, only pan-lactylation exhibited significant upregulation in neutrophils on post-MI/R day 1 (Supplemental Figure 1A; supplemental material available online with this article; https://doi.org/10.1172/JCI194664DS1). Since lactylation is derived from glycolytic stress, we directed our attention to post-MI/R neutrophil lactylation. Pan-lactylation globally increased in the peripheral blood and cardiac-infiltrating neutrophils on day 1 after MI/R (Supplemental Figure 1, B and C).

**Figure 1:**
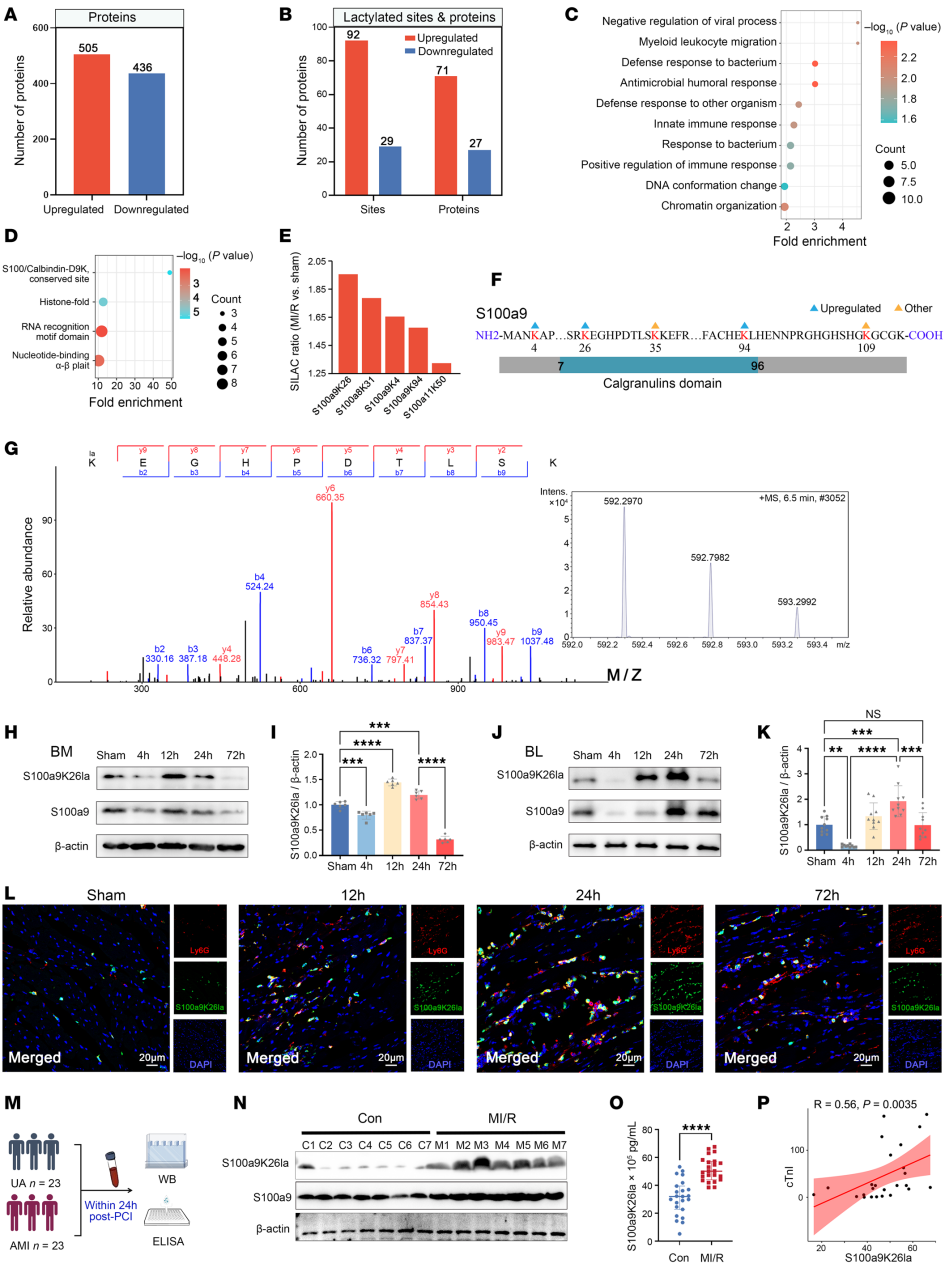
Global view of lactylated proteins and identification of S100a9 lactylation in neutrophils under MI/R. (**A**–**G**) Total and lactylated (Kla) proteomes of BM neutrophils from sham-treated and post-MI/R mice on day 1. (**A**) Number of proteins exhibiting substantial expression-level changes in the total proteome after MI/R. (**B**) Number of Kla sites and proteins exhibiting marked Kla changes after MI/R. (**C**) Bubble plot of the top 10 GO terms representing the functions of proteins that exhibited upregulated Kla changes. (**D**) Domain analysis of proteins with upregulated Kla expression. (**E**) Quantitation of Kla S100a8, S100a9, and S100a11 peptides in neutrophils by MS. SILAC, stable isotope labeling by amino acids in cell culture. (**F**) Illustration of S100a9 Kla sites identified in neutrophils. (**G**) MS/MS spectrum of the lysine 26 lactylated S100a9 peptide (S100a9K26la) derived from neutrophils. (**H**–**K**) S100a9K26la immunoblots of BM (**H**) and blood (BL) neutrophils (**J**) from sham-treated and post-MI/R mice at 4, 12, 24, and 72 hours (*n* = 6). Quantitation of S100a9K26la changes in BM (**I**) and BL (**K**) neutrophils normalized to β-actin. **P < 0.01, ***P < 0.001, and ****P < 0.0001 for the indicated comparisons in **I** and **K**, by 1-way ANOVA with Tukey’s multiple-comparison test (*P* values were adjusted for 6 comparisons). (**L**) Immunofluorescence costaining for Ly6G (red) with S100a9K26la (green) in infarcted hearts 12, 24, and 72 hours after MI/R. Scale bar: 20 μm. Inset: original magnification × 10, *n* = 3. (**M**) Blood samples were collected from patients with AMI undergoing PCI (MI/R, *n* = 23) and from control (Con) patients with UA (*n* = 23) within 24 hours. WB, Western blotting (WB). (**N**) Immunoblotting and quantification of S100a9K26la in neutrophils from the control and MI/R groups. (**O**) Measurement of plasma S100a9K26la levels by ELISA. Median (IQRs: 25th–75th percentiles). ****P < 0.0001 for the indicated comparisons in **O**, by 2-tailed Mann-Whitney *U* test. (**P**) Spearman’s correlation analysis of S100a9K26la and cTnI (excluding the cTnI values below the level of 0.2 μg/L). Data indicate the mean ± SD (**I** and **K)** or Median (interquartile ranges, 25th-75th percentile) (**O**).

To evaluate the significance of neutrophil lactylation in MI/R, we conducted proteomics and lactylation modification proteomics of bone marrow (BM) neutrophils from sham-treated mice and mice subjected to MI/R on day 1. Motif analysis revealed overrepresentation of glycine (G), whereas glutamine (Q) and arginine (R) were less abundant than expected at the −1 and +1 positions surrounding the lactylation sites (Supplemental Figure 1D). Kla proteins were predominantly concentrated in the nucleus, followed by the cytoplasm (Supplemental Figure 1E). On post-MI/R day 1, the expression of 505 proteins was substantially increased, indicating substantial association with leukocyte migration and chemotaxis (Figure 1A and Supplemental Figure 1F). Compared with the sham-treated group, 71 proteins showed substantial upregulation of Kla at 92 sites, whereas 27 proteins showed notable downregulation of Kla at 29 sites, with 73.9% of the sites displaying exclusive Kla alterations (Figure 1B). The top 10 Gene Ontology (GO) terms associated with Kla-modified proteins were mainly enriched in myeloid leukocyte migration and the innate immune response (Figure 1C). Additionally, Kla-modified proteins were prominently represented by the S100/calbindin-D9K domains, including S100a8, S100a9, and S100a11 (Figure 1D). Among these proteins, lysine at position 26 of S100a9 (S100a9K26) exhibited the highest modification intensity by lactylation following MI/R, as indicated by the ranking of their relative lactylation ratios (Figure 1E). Intriguingly, the K26 residue is located in the conserved calgranulin domain of S100a9 (Figure 1F and Supplemental Figure 1G). Thus, we identified S100a9K26 as a potential target of neutrophil lactylation following MI/R and presented its representative mass spectrum (Figure 1G). We generated an S100a9K26la-specific antibody through K26 lactylated peptides (Supplemental Figure 2B). To verify its specificity, we performed dot blot assays using lactylated peptides and unlactylated peptides and co-IP analysis of 32Dcl3-induced neutrophils with K26 arginine (R) and glutamine (Q) mutants, which mimicked the delactylated state of the protein (Supplemental Figure 2, A–D). As expected, both the K26R and K26Q mutants had lower S100a9K26la levels. Intriguingly, the pan-lactylation of S100a9 in immunoprecipitated K26R or K26Q mutants was also lower than that in the WT counterpart (Supplemental Figure 2E), indicating that K26 was the primary lactylation site of S100a9. Moreover, the K26R and K26Q mutants did not alter the levels of S100a8 in the flag-S100a9 complex, indicating that the mutation at the K26 locus and lactylation did not affect the binding of S100a8 with S100a9 (Supplemental Figure 2E).

Subsequently, we identified the expression profile of S100a9K26la in immune cells in heart, blood and BM 1 day after MI/R. S100a9K26la was mostly originated from neutrophils rather than monocytes/macrophages or DCs in heart, blood, and BM 1 day after MI/R (Supplemental Figure 3, A–D). Compared with the sham surgery group, S100a9K26la was highly increased in neutrophils of heart, blood, and BM 1 day after reperfusion (Supplemental Figure 3, E–G). Therefore, we assessed dynamic changes in S100a9K26la levels in neutrophils following MI/R. Immunoblots of BM and circulating neutrophils at various time points after MI/R revealed a significant upregulation of S100a9K26la (normalized to β-actin) at 24 hours, which returned to baseline levels by 72 hours after MI/R (Figure 1, H–K). Compared with S100a9, S100a9K26la (normalized to S100a9) in both BM and blood neutrophils notably increased at 12 hours following MI/R (Supplemental Figure 1, H and I). Consistently, we found sustained cardiac neutrophil S100a9 lactylation throughout the early stages of MI/R, but no significant change in S100a9 lactylation of cardiomyocytes (CMs) (Figure 1L and Supplemental Figure 1K).

To validate this discovery in humans, we examined S100a9K26la levels in circulating neutrophils and plasma between patients with AMI receiving percutaneous coronary intervention (PCI) within 24 hours and those with unstable angina (UA) (Figure 1M and Supplemental Table 1). Both intracellular S100a9K26la levels in circulating neutrophils and plasma S100a9K26la levels showed a notable increase in patients with AMI following PCI (Figure 1, N and O, and Supplemental Figure 1, K and L). Moreover, plasma S100a9K26la levels were significantly correlated with cardiac troponin I (cTnI) levels (Figure 1P).

In brief, neutrophil S100a9K26 lactylation increased early and extensively following MI/R.

### K26 mutation of S100a9 alleviates deleterious inflammation and cardiac dysfunction after MI/R.

**Figure 2:**
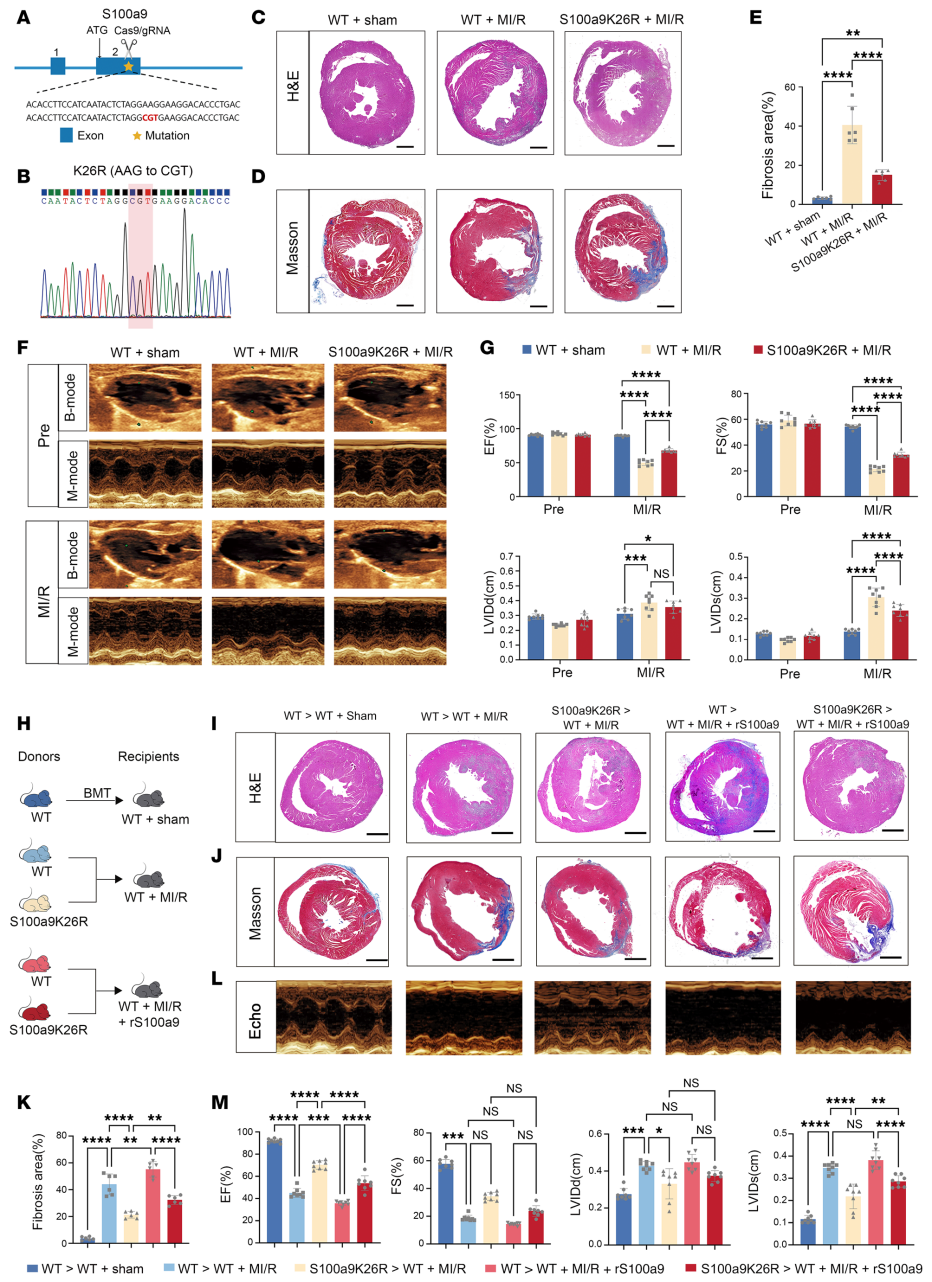
Deletion of S100a9K26-specific lactylation improves post-MI/R cardiac function. (**A**) Strategy for generating S100a9K26R-mutant mice and amino acid alterations at the S100a9 mutation site. (**B**) Nucleotide mutation site in the S100a9K26R mice. (**C**) H&E staining of heart tissue 3 days after MI/R (scale bars: 1 mm; *n* = 6). (**D** and **E**) Fibrotic area quantification and Masson’s trichrome staining 14 days after MI/R (scale bars: 1 mm; *n* = 6). Data indicate the mean ± SD. **P < 0.01 and ****P < 0.0001 for the indicated comparisons in **E**, by 1-way ANOVA with Tukey’s multiple-comparison test (*P* values were adjusted for 6 comparisons). (**F** and **G**) Representative B- and M-mode echocardiograms with measurements on day 14 after MI/R (*n* = 8). Data indicate the mean ± SD. *P < 0.05, ***P < 0.001, and ****P < 0.0001 for the indicated comparisons in **G**, by 2-way ANOVA with Tukey’s multiple-comparison test (*P* values were adjusted for 6 comparisons). (**H**) Schematic representation of the BMT protocol: BM samples from WT and S100a9K26R mice were transplanted into WT recipients and allowed to reconstitute for 6 weeks, after which the mice were subjected to MI/R. Neutrophil-specific S100a9K26R and WT mice were injected with 100 μL rS100a9 (1.5 mg/kg/BW) 4 hours after MI/R, and other control mice were injected with the same volume of saline. (**I**–**K**) H&E-stained images (**I**), Masson’s trichrome–stained images (**J**), and fibrotic area quantification (**K**) of the heart after MI/R. Scale bars: 1 mm (**I** and **J**). *n* = 6 (**I**–**K**). (**L** and **M**) Representative M-mode echocardiograms (**L**) with measurements of EF, FS, diastolic LVID (dLVID), and systolic LVID (sLVID) (**M**) (*n* = 8). Data indicate the mean ± SD. *P < 0.05, **P < 0.01, ***P < 0.001, and ****P < 0.0001 for the indicated comparisons in **K** and **M**, by 1-way ANOVA with Tukey’s multiple-comparison test (*P* values were adjusted for 6 comparisons).

To explore the role of S100a9K26la in MI/R, we generated S100a9K26R-mutant mice to abolish S100a9K26 lactylation, which was achieved by mutating lysine 26 (AAG) to arginine (CGT) (Figure 2, A and B). S100a9K26R mice exhibited lower plasma levels of inflammatory cytokines on post-MI/R day 1 (Supplemental Figure 5, A–C). According to the histopathological analysis, the hearts of S100a9K26R mice exhibited reduced inflammatory cell infiltration compared with those of WT mice on post-MI/R day 3 (Figure 2C). Immunofluorescence analysis revealed diminished neutrophils and myeloperoxidase (MPO) levels in S100a9K26R hearts on post-MI/R day 1 (Supplemental Figure 5D). Further flow cytometric analysis revealed that at 1 and 3 days after MI/R, neutrophils, ly6C^hi^ monocytes, and DCs were notably inhibited in S100a9K26R hearts compared with WT hearts (Supplemental Figure 4, A–E). However, both CD4^+^ and CD8^+^ T cells did not differ between WT and S100a9K26R hearts (Supplemental Figure 4, F–H). Subsequently, S100a9K26R reduced the expression levels of collagen genes and fibrosis-related genes in the heart on post-MI/R days 3 and 7 (Supplemental Figure 5G), as well as improved cardiac fibrosis at post-MI/R day 14 (Figure 2, D and E). Additionally, S100a9K26R mice showed a nonsignificant trend toward improved survival and reduced cardiac rupture incidence compared with WT controls (Supplemental Figure 5, E and F). Importantly, S100a9K26R mice showed significant improvements in post-injury ejection fraction (EF) and fractional shortening (FS), accompanied by a notable decrease in ventricular internal diameter (LVID) end systole (Figure 2, F and G). These findings indicate that the global deletion of S100a9K26la ameliorates MI/R.

We further generated myeloid cell S100a9K26la deletion mice by bone marrow transplantation (BMT). In brief, BM from WT and S100a9K26R mice was transplanted into WT mice before MI/R surgery (Figure 2H). The rate of engraftment is shown in Supplemental Figure 5K. As expected, the myeloid cell–specific S100a9K26R-mutant mice showed dramatically suppressed inflammation and cardiac fibrosis and dysfunction after MI/R (Figure 2, I–M, and Supplemental Figure 5, H–J). Additionally, we performed rescue experiments using recombinant, nonlactylated S100a9 following myeloid-specific deletion of S100a9K26la after MI/R (Figure 2H). The treatment of recombinant S100a9 (rS100a9) remarkably induced inflammation and cardiac dysfunction in MI/R mice with WT donors. However, compared with the MI/R mice with WT donors, the MI/R mice with S100a9K26R donors had significantly reduced inflammation and cardiac dysfunction induced by rS100a9, highlighting the specific damage caused by S100a9K26la in MI/R mice, which was different than that caused by S100a9 (Figure 2, I–M, and Supplemental Figure 5, H–J).

To confirm the specific role of S100a9K26la in neutrophils in MI/R, we i.v. injected recipients 1 hour after MI/R with Ly6G^+^ neutrophils from the BM of either WT or S100a9K26R mice (Supplemental Figure 6, A and B). The efficiency of adoptive transfer (AT) is shown in Supplemental Figure 6C. As expected, the mice receiving S100a9K26R neutrophils displayed a significant reduction of immune cell infiltration and improvement of cardiac dysfunction (Supplemental Figure 6, D–J). Nevertheless, the transfer of S100a9K26R myeloid cells, excluding neutrophils, did not result in any improvement in inflammation or cardiac dysfunction (Supplemental Figure 6, K–T). These findings suggest that targeting lysine at the S100a9K26 site to suppress S100a9K26la of neutrophils yields favorable post-MI/R outcomes.

### Lactylated S100a9 is released via neutrophil extracellular traps and triggers CM death by impairing mitochondrial function.

**Figure 3:**
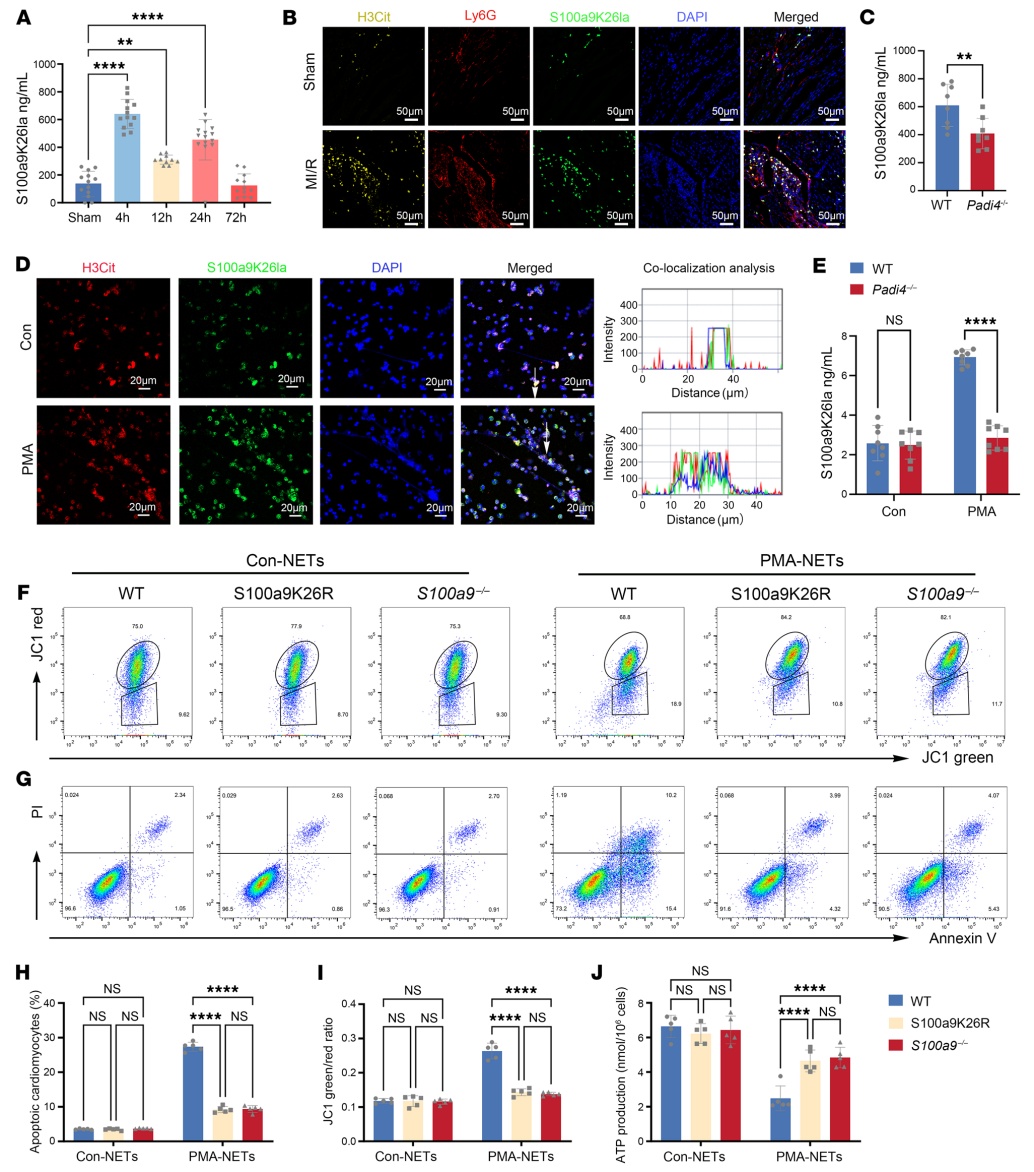
Lactylated S100a9 is released via NETs and triggers CM death by impairing mitochondrial function. (**A**) ELISA quantification of plasma S100a9K26la levels at various time points after MI/R (*n* = 9–13). **P < 0.01 and ****P < 0.0001 for the indicated comparisons in **A**, by 2-way ANOVA with Tukey’s multiple-comparison test (*P* values were adjusted for 6 comparisons). (**B**) Immunofluorescence costaining of heart tissue for H3Cit (orange), and Ly6G (red) with S100a9K26la (green). Scale bars: 50 μm (*n* = 5). Original magnification, ×10. (**C**) ELISA detection of S100a9K26la levels in plasma from WT and *Padi4*^–/–^ mice on day 1 after MI/R (*n* = 8). **P < 0.01 for the indicated comparisons, by unpaired, 2-tailed Student’s *t* test. (**D**) BM neutrophils were treated with vehicle or PMA (200 nM/L) for 4 hours. H3Cit (red) with S100a9K26la (green) immunofluorescence colocalization analysis was performed using Zeiss Zen microscope software. Scale bars: 2 μm (*n* = 5). Scale bars: 20μm, original magnification, ×20 (**E**) BM neutrophils from WT and *Padi4^–/–^* mice were cultured for 4 hours with PMA, and cell supernatants were collected for detection of S100a9K26la levels by ELISA (*n* = 8). ****P < 0.0001 for the indicated comparisons in **E**, by 2-way ANOVA with Tukey’s multiple-comparison test (*P* values were adjusted for 6 comparisons). (**F**–**J**) NCMs were cultured with NETs from WT and S100a9K26R neutrophils pretreated with PMA or untreated for 24 hours, then NCMs were collected to detect ATP levels (**J**) (*n* = 5), mitochondrial membrane depolarization (**F** and **I**, *n* = 5), and annexin V and propidium iodide (PI) by flow cytometry (**G** and **H**, *n* = 5). ****P < 0.0001 for the indicated comparisons in **H**–**J**, by 2-way ANOVA with Tukey’s multiple-comparison test (*P* values were adjusted for 6 comparisons). All data represent the mean ± SD.

Since S100a9 is widely recognized as a secreted alarmin, we checked the lactylated S100a9 in plasma following MI/R through indirect ELISA. We found that in the mouse model, similar to unlactylated S100a9, lactylated S100a9 increased until 72 hours after MI/R compared with the sham-operated mice (Figure 3A and Supplemental Figure 7A). Given that a proportion of S100a9 is bound to neutrophil extracellular traps (NETs) (18), we hypothesized that lactylated S100a9 is released by NETosis. We observed a robust increase in H3Cit, a specific histone H3 marker of NETs, as well as colocalization of S100a9K26la with H3Cit in the cardiac-infiltrated neutrophils after MI/R as well as in isolated neutrophils after 4 hours of phorbol myristate acetate (PMA) treatment (Figure 3, B and D). Flow cytometry analysis also confirmed the S100a9K26la^+^ H3Cit^+^ neutrophils (Supplemental Figure 7E). We established mice with KO of peptidylarginine deiminase 4 *(Padi4)*, an enzyme that is critical for citrullination of histones and NETs, and then subjected the mice to MI/R injury. Deficiency of *Padi4* resulted in a significant decrease in plasma levels of S100a9K26la, S100a9, and H3cit on day 1 after MI/R (Figure 3C and Supplemental Figure 7, B and C). To link NETs directly with extracellular release of S100a9K26la from neutrophils, we treated BM neutrophils from *Padi4^–/–^* or WT mice with PMA for 4 hours. We observed a robust increase of S100a9K29la levels in PMA-treated WT neutrophil supernatants compared with levels in the vehicle-treated group and a notable reduction of S100a9K29la levels in *Padi4^–/–^* cell supernatants compared with WT, which was consistent with S100a9 (Figure 3E and Supplemental Figure 7D). These results suggest that NETs are required for secretion of S100a9K26la.

Extracellular S100a8/a9 was reported to trigger CM death by impairing mitochondria (18). In vitro, we treated BM neutrophils with or without PMA for 4 hours and then collected the conditioned medium or NETs for coculturing with mouse neonatal CMs (NCMs). The NETs, but not the conditioned medium, contributed to CM death by impairing mitochondria (Supplemental Figure 8, A–F). To explore whether lactylated S100a9 directly caused CM death like S100a9 did, NCMs were cocultured with NETs from WT, S100a9K26R, and *S100a9^–/–^* neutrophils stimulated with PMA. Compared with vehicle, the NETs from PMA-treated WT neutrophils notably inhibited ATP production and increased the mitochondrial membrane potential loss in NCMs, resulting in increased NCM death (Figure 3, F–J). Compared with the NETs from WT neutrophils, the NETs from S100a9K26R neutrophils exposed to PMA induced ATP production, lessened the mitochondrial membrane potential loss in NCMs, and attenuated NCM death (Figure 3, F–J). However, DNase I (inhibitor of NETs) eliminated the difference between the WT and S100a9K26R neutrophils (Supplemental Figure 7, G–K). In addition, the NETs from *Padi4^–/–^* neutrophils also reduced mitochondrial dysfunction and NCM death compared with the NETs from WT neutrophils (Supplemental Figure 8, G–K). Of note, S100a9K26R and *S100a9^–/–^* neutrophils showed no difference in their ability to improve mitochondrial dysfunction and NCM death caused by PMA-treated WT neutrophils (Supplemental Figure 7, G–K). Furthermore, BM neutrophils were isolated from WT, S100a9K26R, and *S100a9^–/–^* mice on sham-operated mice or day-1 MI/R mice, and then NETs were collected for coculturing with NCMs in the presence or absence of DNase I. This ex vivo experiment also confirmed that lactylated S100a9 was released via NETosis and directly impaired mitochondria and caused CM death, but lactylation did not contribute to the additional CM death caused by unlactylated S100a9 (Supplemental Figure 7F and Supplemental Figure 8, L–P).

### Lactylated S100a9 translocates into the nucleus and boosts the transcription of intracellular signals directing neutrophil migration.

**Figure 4:**
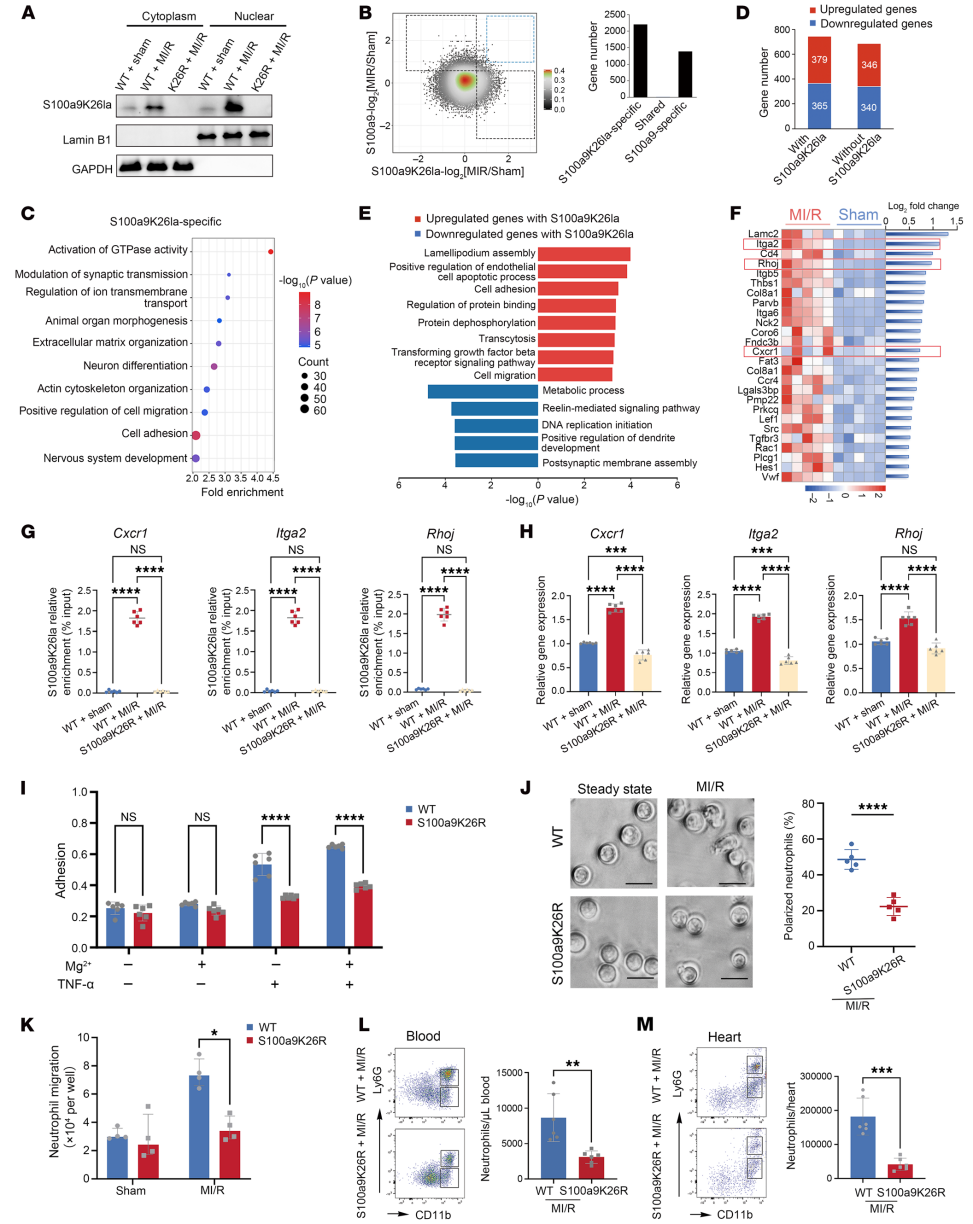
S100a9K26 lactylation boosts the transcription of neutrophil migration and promotes neutrophil recruitment following MI/R. (**A**) Immunoblotting for S100a9K26la, lamin B1, and GAPDH in cellular fractions of BM neutrophils from WT and S100a9K26R mice on post-MI/R day 1 (*n* = 4). (**B** and **C**) CUT-Tag analysis of S100a9K26la in blood neutrophils from sham-treated and post-MI/R mice on day 1. (**B**) Genes marked by exclusively increased in S100a9K26la (S100a9K26la-log_2_[MI/R/sham] ≥0.5 and S100a9-log_2_[MI/R/sham] ≤0.5, S100a9K26la-specific); increased in both S100a9K26la and S100a9 (S100a9K26la-log_2_[MI/R/sham] >1 and S100a9-log_2_[MI/R/sham] >1, shared); or exclusively increased in S100a9 (S100a9-log_2_[MI/R/sham] ≥0.5 and S100a9K26la-log_2_[MI/R/sham] ≤0.5, S100a9-specific). (**C**) Top 10 GO terms for genes with S100a9K26la-specific modifications. (**D**) Number of upregulated and downregulated genes with and without S100a9K26la binding. (**E**) GO terms of upregulated (red) and downregulated (blue) genes with S100a9K26la modification. (**F**) Representative migratory genes ranked by S100a9K26la binding signal (right) and mRNA expression according to the RNA-Seq data (left). (**G** and **H**) S100a9K26la occupancy (**G**) (*n* = 6) and gene expression (**H**) (*n* = 6) in circulating neutrophils at day 1 after MI/R were analyzed using ChIP-qPCR or RT-qPCR. (**I**) Adhesion of BM neutrophils on day 1 after MI/R in the presence or absence of Mg^2+^ (1 mmol/L) was determined on CMECs pretreated or untreated with TNF-α (20 ng/mL, *n* = 6). (**J**) Percentage of polarized neutrophils (with ruffled or extended pseudopods) on day 1 after MI/R after CXCL2 stimulation. Scale bars: 10 μm (*n* = 5). (**K**) Transwell assay of BM neutrophils on day 1 after MI/R with CXCL2 treatment (30 ng/mL) for 2 hours (*n* = 4). Median (IQR: 25th–75th percentiles). (**L** and **M**) Representative flow cytometry plots and quantification of BL (**L**) and heart (**M**) neutrophils (CD45^+^CD11b^+^Ly6G^+^) from WT and S100a9K26R mice on day 1 after MI/R (*n* = 5). *P < 0.05, **P < 0.01, ***P < 0.001, and ****P < 0.0001 for the indicated comparisons, by 1-way ANOVA with Tukey’s multiple-comparison test (*P* values were adjusted for 6 comparisons) (**G** and **H**), 2-way ANOVA with Tukey’s multiple-comparison test (*P* values were adjusted for 6 comparisons) (**I**), 2-tailed Mann-Whitney *U* test (**K**), and unpaired, 2-tailed Student’s *t* test (**J**, **L**, and **M)**. All data indicate the mean ± SD.

To explore the role of lactylated S100a9 within intracellular neutrophils, we examined the distribution of S100a9K26la and observed strong nuclear localization of S100a9K26la in neutrophils following MI/R (Figure 4A). Confocal analysis of circulating neutrophils on post-MI/R day 1 confirmed a strong increase in nuclear S100a9K26la following MI/R (Supplemental Figure 9A). Using co-IP coupled to mass spectrometry (MS), we found that S100a9K26la was bound to importin β1, which was further confirmed using co-IP analysis (Supplemental Figure 9, B–D). The importin β1 inhibitor blocked the translocation of S100a9K26la into nuclei, leading to a decrease in nuclear S100a9K26la (Supplemental Figure 9E). Additionally, in circulating neutrophils, S100a9K26la was observed to bind to histones, and its fractions were identified within nuclear proteins using MS (Supplemental Figure 9, F and G). These findings suggest that S100a9K26la translocates into nuclei in an importin β1–dependent manner and potentially regulates or influences gene transcription.

Next, we performed cleavage under targets and tagmentation (CUT-Tag) assays of circulating neutrophils from sham-operated and MI/R mice 24 hours after MI/R using anti-S100a9K26la and anti-S100a9 antibodies (Supplemental Figure 10A). S100a9K26la was predominantly enriched in the promoter regions of these genes (Supplemental Figure 10B). The majority of genes exhibiting increased S100a9K26la marking were not marked by S100a9. S100a9K26la marked more genes (61% of all genes marked by S100a9K26la or S100a9) than did S100a9 (39% of all marked genes) (Figure 4B). S100a9K26la-specific genes had a substantial effect on genes involved in cell migration and adhesion, whereas the S100a9-specific genes were enriched in the positive regulation of the force of heart contractions (Figure 4C and Supplemental Figure 10C). To identify S100a9K26la target genes in neutrophils after MI/R, we integrated the CUT-Tag and RNA-Seq data on circulating neutrophils from sham and MI/R-mice 24 hours after MI/R and then classified the genes into 4 categories on the basis of their expression levels and S100a9K26la modification (Figure 4D). Genes with altered expressions that were marked by S100a9K26la underwent various biological processes. As expected, upregulated genes with S100a9K26la binding were largely associated with cell migration and adhesion (Figure 4E). Key intracellular signals for cell migration included chemokine receptors, Rho and Rac GTPase signals governing neutrophil polarization, and adhesion receptor integrins (Figure 4F). Based on the fold-change rank of the S100a9K26la binding signal, integrin α 2 (*Itga2*), ras homolog family member J (*Rhoj)*, and C-X-C motif chemokine receptor 1 (*Cxcr1)* were substantially upregulated (Figure 4F).

We further conducted reverse transcription quantitative PCR (RT-qPCR) and ChIP-qPCR analysis to confirm the increased expression and S100a9K26la enrichment in the promoter regions of Itga*2*, *Rhoj*, and *Cxcr1* in circulating neutrophils 24 hours after MI/R. Deletion of S100a9K26la sharply attenuated mRNA levels and S100a9K26la marks at the *Itga2*, *Rhoj*, and *Cxcr1* promoters (Figure 4, G and H). Furthermore, a genomic snapshot suggested that S100a9K26la strongly binds to *Itga2*, *Rhoj*, and *Cxcr1*, whereas the binding of S100a9 was weak (Supplemental Figure 10D). ChIP-qPCR analysis of S100a9 indicated that S100a9 did not enrich in the target genes and did not contribute to migratory gene activity (Supplemental Figure 10E).

We further established BM neutrophil–activated models in vitro with LPS stimulation for 4 hours and found that glycolysis and S100a9 lactylation in activated neutrophils dramatically increased (Supplemental Figure 11, A–C). Next, we treated activated BM neutrophils with exogenous sodium lactate (NaLa) or the lactate dehydrogenase inhibitor sodium oxamate (Oxa) for 4 hours. Exogenous NaLa augmented S100a9K26la levels in a dose-dependent manner, leading to increased mRNA expression of *Itga2*, *Rhoj*, and *Cxcr1*, along with increased S100a9K26la marks at their promoter regions and enhanced neutrophil migratory ability, whereas Oxa attenuated these effects (Supplemental Figure 11, D–K). Therefore, we identified *Itga2*, *Rhoj*, and *Cxcr1* as potential targets of neutrophil S100a9K26la following MI/R.

Collectively, S100a9K26la acted as a coactivator and selectively bound to the promoter region of target genes, thereby directly promoting the expression of migration-related molecules following MI/R.

### S100a9K26R suppresses neutrophil migration and recruitment after MI/R.

RNA-Seq analysis of neutrophils isolated from the blood and heart of S100a9K26R and WT mice on day 1 after MI/R revealed the inhibition of migration-related pathways in S100a9K26R neutrophils from blood and heart (Supplemental Figure 12, A and B). The expression of genes involved in neutrophil migration, such as integrin and chemokine receptor genes, was markedly reduced in the blood and heart of S100a9K26R mice, owing to the lack of S100a9K26la (Supplemental Figure 12C). Therefore, S100a9K26R damaged the migratory transcriptional landscape of neutrophils. Notably, S100a9K26R mice exhibited decreased protein expression of CXCR1, C-X-C chemokine receptor 2 (CXCR2), integrin αMβ2 (Mac-1), and lymphocyte function–associated antigen 1 (LFA-1) in neutrophils from blood and BM on day 1 after MI/R compared with WT (Supplemental Figure 12, D–G). Consistently, the Transwell assay and neutrophil polarization analysis confirmed that S100a9K26R neutrophils isolated from the BM on post-MI/R day 1 exhibited significantly impaired migration toward CXCL2 in vitro (Figure 4, J and K). In static adhesion on endothelial cells in vitro, in the presence of Mg^2+^, there was significantly reduced adhesion of S100a9K26R neutrophils on TNF-α–treated mouse cardiac microvascular endothelial cells (CMECs) compared with WT controls (Figure 4I), indicating an adhesion defect of S100a9K26R neutrophils. Additionally, S100a9K26R neutrophils also exhibited significantly impaired adhesion and polarization on the integrin ligand intercellular cell adhesion molecule 1 (ICAM-1) (Supplemental Figure 12, H–J). Importantly, addition of functional blocking antibodies specific for Mac-1 or LFA-1 markedly reduced the adhesion and polarization of WT neutrophils on ICAM-1, without further inhibiting the defective adhesion and polarization of S100a9K26R neutrophils (Supplemental Figure 12, H–J). During the acute setting of MI/R, S100a9K26R significantly reduced neutrophil accumulation in both the blood and injured heart, but increased the retention of neutrophils in BM (Figure 4, L and M, and Supplemental Figure 12K). Furthermore, we observed no difference in the frequency of apoptotic neutrophils in the heart, blood, or BM between WT and S100a9K26R mice on day 1 following MI/R (Supplemental Figure 12, L–N), suggesting that neutrophil apoptosis was not the cause of reduced cardiac neutrophil counts in S100a9K26R MI/R mice. Thus, S100a9K26R led to defective neutrophil migration and the reduction of cardiac neutrophil accumulation after MI/R due to deletion of S100a9K26-specific lactylation.

### DLAT directly catalyzes S100a9 lactylation in neutrophils.

**Figure 5:**
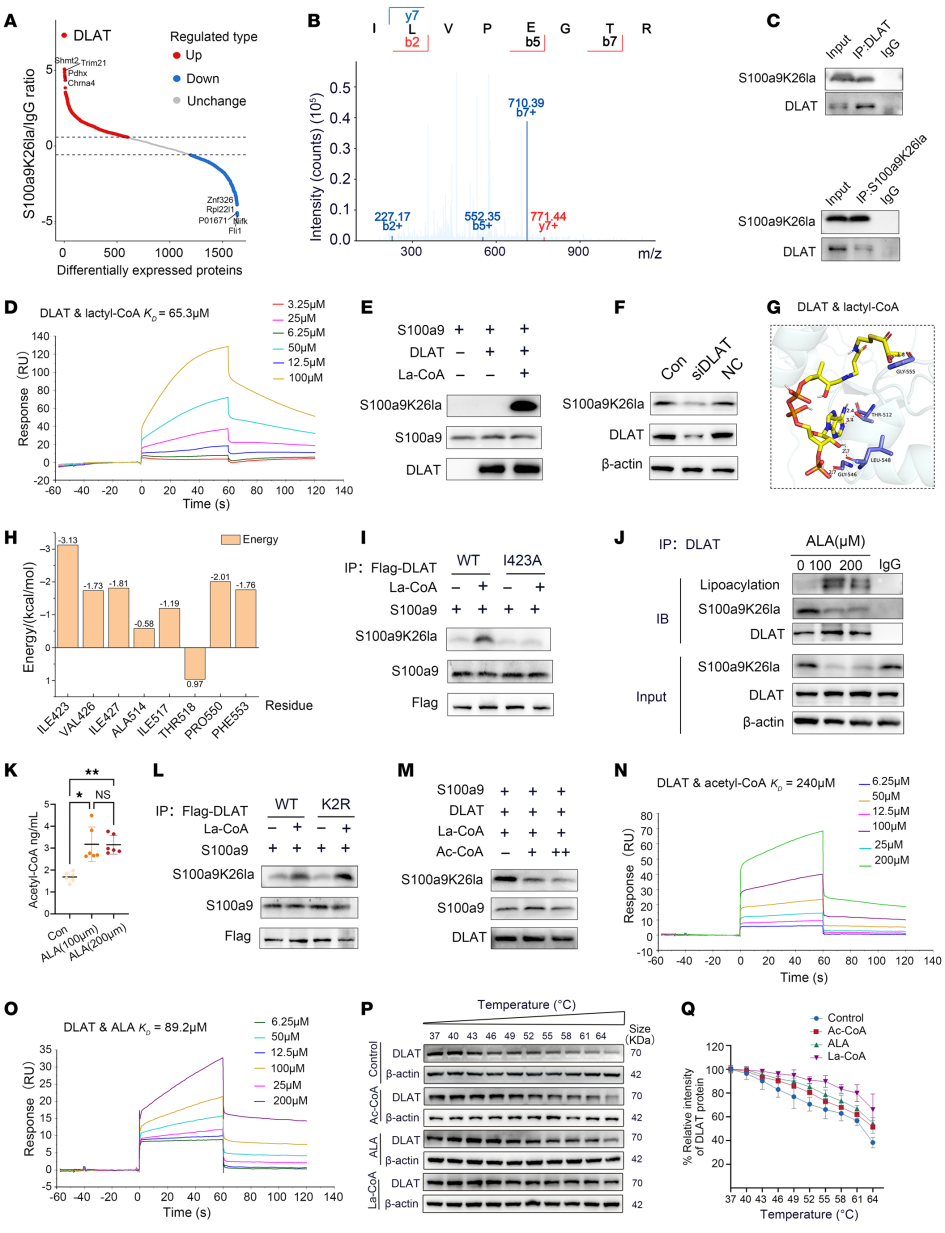
DLAT-catalyzed S100a9 lactylation in neutrophils can be antagonized by ALA. (**A**) Differentially abundant proteins were identified in neutrophils by IP and MS analysis using anti-S100a9K26la and IgG antibodies. (**B**) MS/MS spectrum of DLAT. (**C**) IP assay of BM neutrophils following MI/R with the indicated antibodies (*n* = 4). (**D**) SPR analysis of lactyl-CoA binding to the recombinant DLAT protein. (**E**) An in vitro lactylation assay was performed in the presence or absence of lactyl-CoA (*n* = 4). (**F**) 32Dcl3 cells were differentiated into granulocytes with addition of G-CSF for 4 days and transfected with control siRNA (NC) or DLAT specific siRNA (siDLAT) for 24 hours, and then collected for immunoblot analysis (*n* = 4). (**G**) Molecular docking between DLAT and lactyl-CoA. (**H**) Per-residue energy contributions of key residues involved in lactyl-CoA combined with DLAT. (**I**) Purified rS100a9 was incubated with immunoprecipitates of WT Flag-DLAT or the I423A mutant from HEK293T cells followed by detection of S100a9K26 lactylation (*n* = 6). (**J** and **K**) BM neutrophils were stimulated with the indicated doses of ALA and then collected for IP with anti-DLAT (**J**) (*n* = 4) and intracellular acetyl-CoA detection by ELISA (**K**) (*n* = 6). Data indicate the mean ± SD. *P < 0.05 and **P < 0.01 for the indicated comparisons, by 1-way ANOVA with Tukey’s multiple-comparison test (**K**). (*P* values were adjusted for 6 comparisons.) (**L**) Purified rS100a9 was incubated with immunoprecipitates of WT Flag-DLAT or the K131/258R mutant (K2R)) from HEK293T cells followed by detection of S100a9K26la (*n* = 6). (**M**) In vitro lactylation assay in the presence or absence of acetyl-CoA (*n* = 4). (**N** and **O**) SPR analysis of acetyl-CoA (**N**) and ALA (**O**) binding to the recombinant DLAT protein. (**P** and **Q**) Flag DLAT plasmid was transfected into HEK293T cells, and then cell lysates were collected and incubated with vehicle or lactyl-CoA, acetyl-CoA, or ALA, followed by a CETSA (*n* = 4).

To determine the underlying enzyme that catalyzed S100a9 lactylation in neutrophils, we used MS to analyze anti-S100a9K26la immunoprecipitates from BM neutrophils 1 day after MI/R. As shown in Figure 5A, DLAT was the protein that most substantially bound to S100a9K26la. DLAT functions to catalyze protein acetylation as reported previously (17, 19), thus, we asked whether it lactylates S100a9 (Figure 5B). The interaction between DLAT and S100a9K26la was validated by reciprocal IP analyses with either an anti-S100a9K26la or anti-DLAT antibody (Figure 5C) and was primarily localized in the cytosol (Supplemental Figure 13A).

We further used purified recombinant protein of DLAT to examine its interaction with lactyl–coenzyme A (lactyl-CoA) by surface plasmon resonance (SPR) assay. Indeed, lactyl-CoA was found to bind DLAT with a *K_D_* value of 65.3 μM, as did the positive control acetyl-CoA (*K_D_* = 240 μM) (Figure 5D). In vitro lactylation experiments suggested that DLAT lactylated S100a9 (Figure 5E). Moreover, knockdown of DLAT in neutrophils by siRNA remarkably inhibited the lactylation levels of S100a9 (Figure 5F and Supplemental Figure 13B). Furthermore, molecular docking analysis indicated that amino acid residues of DLAT interacted with lactyl-CoA through hydrogen bonds (Figure 5G), and the critical residue with the top average energy value was isoleucine 423 (I423), as indicated by dynamics simulations (Figure 5H). Therefore, we generated catalytically fewer active forms of DLAT (I423A, alanine), which demonstrated a decreased ability to lactylate S100a9 at K26 compared with the corresponding control WT acetyltransferases in the in vitro lactyltransferase activity assay (Figure 5I and Supplemental Figure 13C). Therefore, DLAT is the potential lactyltransferase of S100a9 lactylation, whose catalytical activity depends on the critical residue I423.

Lipoic acid is a typical substrate of DLAT, promoting DLAT lipoacylation and PDH activity, thereby leading to increased acetyl-CoA (20, 21). Consistently, the treatment of α-lipoic acid (ALA) significantly increased the DLAT lipoacylation and PDH activity, as well as the acetyl-CoA level in neutrophils, while the deletion of S100a9K26la did not affect the PDH activity and acetyl-CoA level (Figure 5, J and K, and Supplemental Figure 13, D–F). Instead, ALA inhibited S100a9 lactylation and the binding of S100a9K26la to DLAT (Figure 5J and Supplemental Figure 13D). This might be due to the competitive combination of ALA or acetyl-CoA with DLAT. To test this hypothesis, we generated delipoacylated DLAT by mutating both K131 and K258 residues to R, which showed an increased ability to catalyze S100a9 lactylation compared with the corresponding WT (Figure 5L and Supplemental Figure 13G). Moreover, the presence of acetyl-CoA reduced S100a9 lactylation levels (Figure 5M and Supplemental Figure 13H), suggesting that ALA served as a lactyltransferase inhibitor by providing a lipoacyl group and inducing acetyl-CoA. Actually, according to the SPR assay, lactyl-CoA revealed a higher efficient binding affinity to DLAT than did acetyl-CoA or ALA (Figure 5, N and O), which was further verified by a cellular thermal shift assay (CETSA) (Figure 5, P and Q). Therefore, DLAT-mediated S100a9 lactylation in neutrophils was efficiently antagonized by ALA.

### Targeting DLAT/S100a9K26la signaling improves acute inflammation and cardiac dysfunction following MI/R.

**Figure 6:**
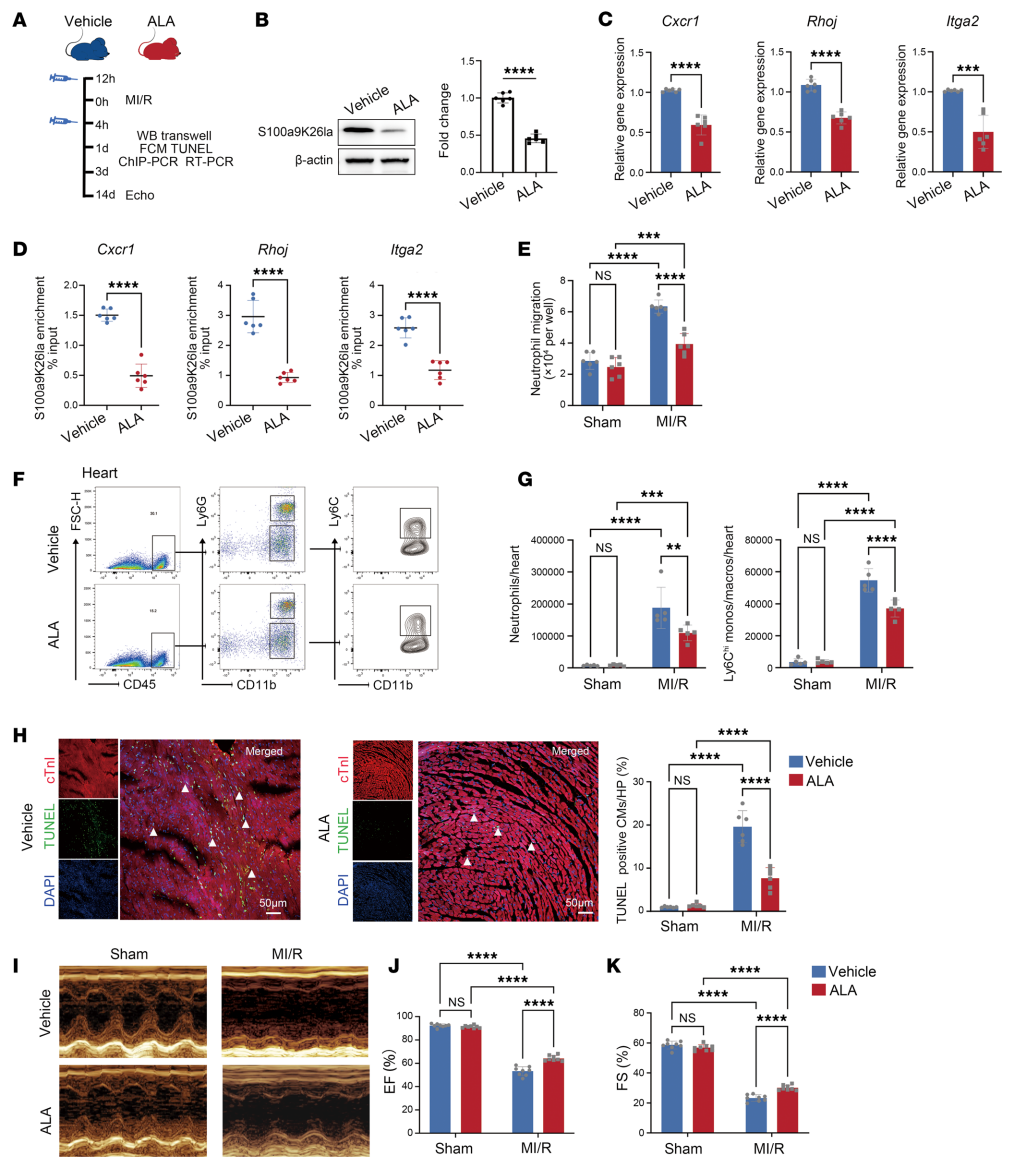
ALA inhibits S100a9 lactylation and improves MI/R outcomes**.** (**A**) Experimental protocol: mice received i.p. injections of vehicle or ALA (30 mg/kg) at different time points. (**B**) S100a9K26la levels in circulating neutrophils were assayed by WB (*n* = 6). (**C**) Gene expression analysis by RT-qPCR (*n* = 6). (**D**) S100a9K26la occupancy analysis by ChIP-qPCR (*n* = 6). (**E**) BM neutrophils from vehicle- or ALA-treated mice 1 day after MI/R were collected and treated with CXCL2 (30 ng/mL) for 2 hours, and migration was determined by Transwell assay (*n* = 6). (**F** and **G**) Representative flow cytometry plots and quantification of heart-infiltrating CD45^+^CD11b^+^Ly6G^–^Ly6C^hi^ monocytes/macrophages and CD45^+^CD11b^+^Ly6G^+^ neutrophils 1 day after MI/R (*n* = 6). (**H**) Representative images of TUNEL and cTnI double staining and quantitation of TUNEL^+^ CMs (*n* = 6). Scale bars: 50 μm, original magnification: ×20. (**I** and **K**) Representative M-mode echocardiograms and echocardiogram measurements (*n* = 8). All data indicate the mean ± SD. **P < 0.01, ***P < 0.001, and ****P < 0.0001 for the indicated comparisons, by 2-tailed Student’s *t* test (**B**–**D**) and 2-way ANOVA with Tukey’s multiple-comparison test (**E**, **G**, **H**, **J**, and **K**). (*P* values were adjusted for 6 comparisons.)

Given that ALA is a S100a9 lactylation inhibitor, we next determined whether ALA could mitigate MI/R injury. WT mice were i.p. injected with ALA or vehicle before and after MI/R (Figure 6A). In ALA-treated mice, S100a9K26la levels in circulating neutrophils significantly decreased, which resulted in a significant reduction of S100a9K26la marks on target genes and mRNA expression of these genes (Figure 6, B–D), as well as the expression levels of adhesion molecules of neutrophils (Supplemental Figure 14, L and M). Consistently, neutrophils from BM treated with ALA displayed a damaged ability to migrate toward CXCL2 compared with the vehicle (Figure 6E). Moreover, ALA injection significantly inhibited neutrophil and Ly6C^hi^ monocyte and macrophage infiltration into heart after MI/R (Figure 6, F and G). The gating strategy for identification of leukocytes is shown in Supplemental Figure 16, A and B. Consistently, CM death, determined by TUNEL and cTnI double staining, was markedly reduced in ALA-treated mice compared with WT mice after MI/R (Figures 6H). Importantly, ALA-treated mice showed significant improvements in post-injury EF and FS 14 days after MI/R (Figure 6, I–K). Furthermore, we observed that administration of ALA markedly mitigated MI/R in a dose-dependent manner through inhibition of S100a9K26la, reducing neutrophil recruitment to the heart and CM death and improving cardiac dysfunction (Supplemental Figure 14, A–K).

Additionally, S100a9K26la custom antibodies were adopted to block S100a9K26la. To test whether S100a9K26la-neutralizing antibodies could enter the cells, we isolated circulating neutrophils from WT mice 1 day after MI/R and treated them with 10 μg S100a9K26la antibody or IgG conjugated with 488 dye for 2 hours. Immunofluorescence showed that S100a9K26la antibody conjugated to Alexa Fluor 488 dye (green) was located in the intracellular space of neutrophils (Supplemental Figure 15A). Using HRP-coupled anti–rabbit IgG antibodies that were directly incubated on PVDF membranes containing neutrophil cytoplasmic protein, (Supplemental Figure 15B). These data suggested that the antibody could enter the cells. Furthermore, functional analysis using ChIP-PCR, PCR, and Transwell assays suggested that, compared with IgG antibody, S100a9K26la antibody inhibited the S100a9K26la marks on target genes and the mRNA expression of these genes in neutrophils, as well as the migratory ability of the neutrophils (Supplemental Figure 15, C–E). In summary, S100a9K26la antibody entered the neutrophils partially via caveolae-dependent endocytosis and inhibited the transcription of target genes and neutrophil migratory ability. In vivo, S100a9K26la custom antibodies reduced inflammatory cytokine levels in plasma, inhibited the accumulation of neutrophils, Ly6Chi monocytes, and macrophages in the blood and heart, and improved myocardial fibrosis and cardiac dysfunction after MI/R.(Supplemental Figure 15, F–L).

### S100a9K26la is associated with cardiac death in patients with AMI who underwent PCI.

**Figure 7:**
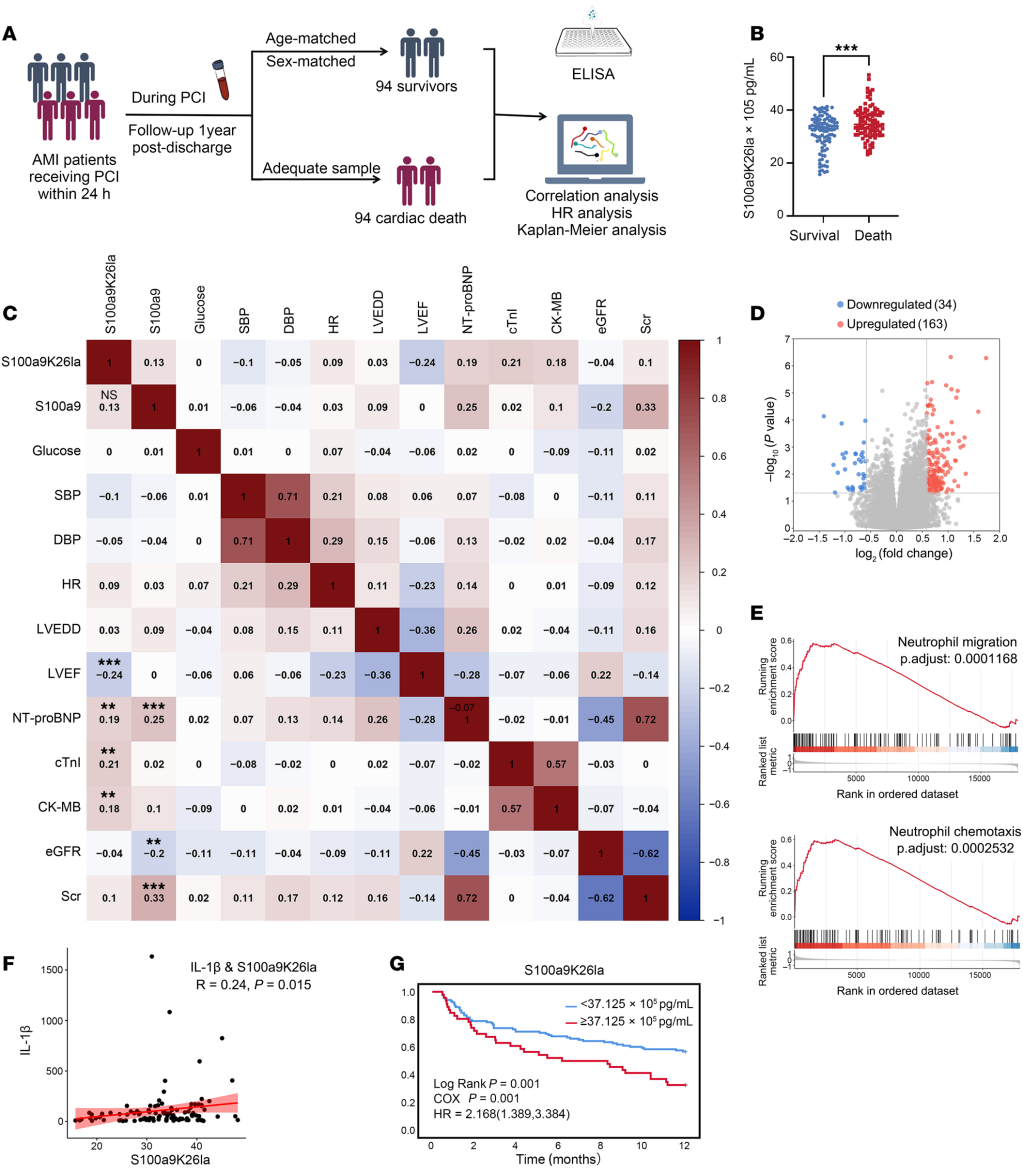
Lactylated S100a9 is associated with cardiac death in patients with AMI. (**A**) Flow diagrams of the cardiac death cohort for assessment of plasma S100a9K26la levels and its prognostic value for cardiac death in patients with AMI receiving PCI. The final eligible 94 patients who died and the 94 survivor-matched controls were enrolled in this cohort according to the occurrence of cardiac death during 1-year follow-up period. Blood samples were collected from the arterial access site after stent implantation during the interventional procedures. (**B**) Plasma levels of S100a9K26la were measured by ELISA. Median (IQRs: 25th–75th percentiles). ***P < 0.001 for the indicated comparisons, by 2-tailed Mann-Whitney *U* test. (**C**) Spearman’s correlation analysis of S100a9K26la and S100a9 with myocardial injury markers and cardiac function indexes. (**D** and **E**) RNA-Seq of circulating CD45^+^ immune cells from 55 pairs of surviving patients and patients who died, with a volcano plot (**D**) of all genes and GSEA (**E**) of genes. (**F**) Spearman’s correlation analysis of S100a9K26la and IL-1β. (**G**) Kaplan-Meier plots of long-term cardiac death based on high or low S100a9K26la levels on day 1 after PCI in patients with AMI.

To further evaluate the clinical utility of S100a9K26la in predicting cardiac risk following MI/R injury, we conducted a retrospective study involving 188 patients with AMI who presented within 12 hours of symptom onset and underwent PCI within 24 hours. Among these patients, 94 were categorized as being at high risk for AMI and experienced cardiac death within a 1-year follow-up period, while the remaining 94 low-risk patients survived (Figure 7A and Supplemental Table 2). The levels of both S100a9K26la and S100a9 were significantly elevated in AMI patients who died of cardiac causes compared with patients who survived following PCI (Figure 7B and Supplemental Figure 17B). Furthermore, S100a9K26la showed significant associations with baseline myocardial injury markers (creatine kinase isoenzyme MB [CK-MB] and cTnI) and baseline cardiac function indexes (left ventricular EF [LVEF] and N-terminal pro–brain natriuretic peptide [NT-proBNP]), whereas S100a9 showed no such associations with myocardial injury markers (Figure 7C and Supplemental Table 3). Of note, during the follow-up period, the frequency of intercurrent heart failure (HF) events was markedly increased in the patients who died compared with the survivor-matched controls. However, there was no difference in the frequency of recurrent MI between these 2 groups (Supplemental Figure 17G). Additionally, S100a9K26la levels were remarkably negatively correlated with LVEF at the 1-month follow-up in patients with AMI (Supplemental Figure 17H). We also observed a consistent negative trend, but statistically nonsignificant association, between S100a9K26la levels and LVEF at the 3-, 6-, and 12-month follow-up points for patients with AMI (Supplemental Figure 17I). We further analyzed 55 pairs of patients who survived or experienced cardiac death during the follow-up period by conducting RNA-Seq analysis to delineate transcriptional profiles associated with high-risk AMI patients who die of cardiac causes. The genes with differential expression between survivors and deceased patients were substantially enriched in pathways related to inflammatory responses and neutrophil chemotaxis (Figure 7D and Supplemental Figure 17A). Subsequent gene set enrichment analysis (GSEA) indicated that the predominant pathological process underlying cardiac death was associated with neutrophil migration and neutrophil chemotaxis (Figure 7E). S100a9K26la levels exhibited a significant correlation with IL-1β expression (Figure 7F). The HR analysis revealed that elevated S100a9K26la levels were associated with a significantly increased risk of cardiac death (HR 2.168 [1.389, 3.384], Figure 7G). Importantly, based on the optimal cut-off value, patients with high S100a9K26la levels (≥37.125 × 10^5^ pg/mL) on post-PCI day 1 were more likely to experience cardiac death during long-term follow-up (Figure 7G). Furthermore, considering that bacterial stimulation can induce lactate production and lactylation, we excluded the patients with infection (Supplemental Table 4). S100a9K26la also served as a marker associated with a heightened risk of cardiac death and relatively poor outcomes in patients with AMI without infections (Supplemental Figure 17, C–F).

## Discussion

The early burst of excessive inflammation after AMI contributes to reperfusion injury (1). Despite the benefits of antiinflammation therapies targeting residual inflammatory risk in patients in the chronic post-AMI phase (22), the early inflammatory response during AMI has not shown any breakthrough clinical success so far (23, 24). Thus, the task remains to identify ideal targets for acute excessive inflammation outbreaks and develop effective and safe agents against these targets in AMI. Our study elucidates the role of S100a9 lactylation in acute inflammation as an intrinsic trigger following MI/R, identifies lactylation as a potential therapeutic target and biomarker, and establishes a foundation for the development of pharmacological interventions. Key findings of our study include: (a) the demonstration of neutrophil S100a9 lactylation and its transcriptional regulation; (b) the release of lactylated S100a9 via NETs, which triggered CM death by impairing mitochondrial function; (c) the identification of specific lactyltransferase DLAT and its inhibitor ALA; (d) the blockade of lactylation signaling driven by DLAT improved cardiac function following MI/R; (e) an independent association of S100a9K26la with cardiac death for patients with AMI following PCI.

Recently, histone lactylation, as a posttranscriptional modification, was found to play diverse roles in different cells during MI and other diseases (25–28). In addition, nonhistone protein lactylation, a precise and direct factor, plays a critical role in cardiac fibrosis and HF (12, 14). However, the function of lactylation in neutrophils during MI/R remains unexplored. In this study, we demonstrate that neutrophil S100a9 lactylation was elevated early and triggered CM death and neutrophil recruitment following MI/R. Given that lactylated S100a9 and unlactylated S100a9 had a similar effect on mitochondrial dysfunction and CM death, lactylation did not contribute to the effect of secreted S100a9 on CM death. However, S100a9K26R substantially reduced the inflammation and cardiac dysfunction induced by recombinant S100a9, indicating that lactylated S100a9 has a specific disadvantage in MI/R mice that differs from the effects of S100a9. Actually, lactylation modified S100a9 and promoted S100a9 translocation into the nuclei of neutrophils. There, lactylated S100a9 specifically boosted the expression of *Cxcr1*, *Rhoj*, and *Itga2* after MI/R, directing cell polarization, adhesion, and migration. As sentinels of inflammation, neutrophils are primarily mobilized from the BM and recruited to the infarcted heart after MI/R. Then, in the heart, neutrophils aggravate the damage through secretion of damage-associated molecular patterns (DAMPs) and increase CM death in a process involving NETs. Our data indicated that S100a9 lactylation plays an important role in the process of neutrophil migration and recruitment during MI/R. Considering the immune privilege of BM, where the stem cell niche is protected from the remnants of lytic cell death, especially of neutrophils (29), we propose a hypothetical paradigm about the functional pattern of S100a9 lactylation. First, in response to MI/R, BM and circulating neutrophils upregulate S100a9 lactylation, which serves as a coactivator in neutrophil nuclei to manipulate the transcription of migration-related genes and promote their migration toward ischemic heart tissue. Subsequently, neutrophils may undergo NETosis and release lactylated S100a9 locally within the ischemic microenvironment, where they additionally cause CM death by impairing mitochondrial function.

From a clinical perspective, S100a9 lactylation levels were significantly elevated in neutrophils and plasma in patients with AMI receiving PCI, who had a significantly increased risk of cardiac death and were more likely to experience cardiac death during long-term follow-up. Although the mechanism by which this might occur is unclear, during the follow-up period, both the significantly increased frequency of intercurrent HF and the significant negative correlation between S100a9K26la levels and LVEF at baseline or the first month of follow-up suggested that cardiac dysfunction is the important functional mechanism of S100a9 lactylation. In addition, we found that S100a9 lactylation levels had obvious associations with the proinflammatory cytokine IL-1β as well as myocardial injury (CK-MB and cTnI) in patients with AMI. Moreover, despite S100a9 being widely recognized as a secreted alarmin and the fact that numerous studies have reported the cardiac benefits of blocking S100a9 (30–34), the transcriptional regulatory role of S100a9 in neutrophil nuclei has not to our knowledge been reported yet. We demonstrate a nuclear function of a lactylated form of S100a9 protein, discriminating from the extracellular mechanism of secreted S100a9 We demonstrate a nuclear function of a lactylated form of S100a9 that is distinct from the extracellular mechanism of secreted S100a9. Deletion of neutrophil S100a9K26 lactylation resulted in the suppression of cardiac inflammation and cardiac dysfunction after MI/R. These findings underscore the direct pathological effect of S100a9 lactylation on MI/R. Collectively, S100a9 lactylation represents a pathological mechanism and probably be a key acute inflammatory target to specifically minimize cardiac neutrophil counts and acute excessive inflammation following MI/R and is a promising alternative biomarker for evaluating acute inflammatory injury and late prognosis.

Neutrophil counts positively correlate with adverse clinical outcomes in patients with cardiovascular disease (3). Targeting neutrophil numbers may represent a strategy for treating MI/R (35, 36). Currently, reducing neutrophil burden by inhibiting neutrophil migration and recruitment constitutes an important therapeutic approach (37). However, the redundancy of chemokines during neutrophil recruitment poses challenges in transitioning chemokine targeting from preclinical models to clinical trials (38). Therefore, targeting the intrinsic pathway that induces neutrophil migration may represent a therapeutic approach for reducing neutrophil burden (39, 40). Our study revealed that S100a9 lactylation was elevated during neutrophil burst following MI/R and declined as cardiac-infiltrating neutrophils gradually decreased, serving as an intrinsic regulator that enhances neutrophil migration to the injured heart. Although this study did not specifically investigate cardiac-resident neutrophils or BM granulopoiesis, impaired neutrophil migration resulting from lactylation offers a crucial mechanistic basis for the observed reduction in cardiac neutrophil infiltration. These findings highlight the potential application of targeting neutrophil S100a9 lactylation to specifically minimize cardiac neutrophil counts during the neutrophil overload period after MI/R.

Neutrophils are generally considered detrimental in the setting of AMI, yet they have been shown to be required for the subsequent cardiac repair processes through antiinflammation, proangiogenesis, and induction of a proreparative macrophage phenotype (41–43). As reported before, 7–14 days after infarction, neutrophil depletion leads to worsening of heart function (41). Additionally, short-term S100A9 blockade after injury improves cardiac function, but prolonged inhibition for 7 or 21 days leads to progressive deterioration of cardiac function and LV dilation (30, 44). Therefore, the precise timing of targeting neutrophils and their inflammatory mediators is crucial in determining the outcome of MI. In our study, pharmacological and genetic inhibition of the DLAT/S100a9K26la pathway during the early stage of MI/R substantially inhibited neutrophil infiltration and improved cardiac function. However, it is uncertain whether targeting this pathway more than 7–21 days after MI/R is detrimental to cardiac healing through disruption of the beneficial roles of neutrophils in antiinflammation, proangiogenesis, and promotion of a proreparative macrophage phenotype. Although S100a9K26R knock-in did not affect immune cells on day 14 after MI/R, further research is needed to evaluate the long-term consequences of modulating this pathway after MI/R.

Our study elucidates the mechanism underlying the increase in S100a9 lactylation during MI/R, identifying the lactyltransferase DLAT. As an indispensable part of cellular metabolism (45), DLAT catalyzes the conversion of pyruvate to acetyl CoA. We revealed that DLAT functions as a lactyltransferase to catalyze lactylation of S100a9, thereby mediating migratory gene expression, highlighting a nonmetabolic function of this protein in regulating neutrophil behavior. ALA was confirmed to be able to inhibit lactylation and reduce the migration ability of neutrophils, leading to improvement of cardiac function of MI/R mice. Importantly, as a clinical medicine for the treatment of diabetic peripheral neuropathy, clinical trials suggested that lipoic acid could reduce peripheral neuropathy pain intensity experienced by patients with type 2 diabetes mellitus and improve insulin sensitivity in prediabetic individuals (46–48). Moreover, lipoic acid can improve endothelial dysfunction in individuals with impaired fasting glucose or type 2 diabetes (49–51). Based on our work and previous reports, lipoic acid is may have potential application in a precisely targeted antiinflammatory strategy for patients with AMI, especially those with diabetes. Thus, ALA might be a promising pharmacological therapy to protect against MI/R and deserves further clinical research to explore its clinical utility.

In summary, our results provide the first evidence to our knowledge of the elevation of S100a9 lactylation in neutrophils in MI/R mice and patients with AMI undergoing PCI. DLAT-catalyzed S100a9 lactylation triggered migration-related gene activation in neutrophils in the early stage of MI/R and aggravated the reperfusion injury through amplification of acute inflammation. The use of ALA or S100a9K26la antibodies constitute a promising strategy to attenuate post-MI/R cardiac dysfunction through blocking S100a9 lactylation driven by DLAT. Given the marked association with cardiac death in patients with AMI, S100a9K26la may be a therapeutic target and clinical prognostic biomarker for MI/R.

This study had several limitations. First, we did not investigate other modifications of S100a9, such as phospho-S100a9 (52), and the complex crosstalk mechanism of the posttranslational modification of S100a9 warrants further investigation. Second, the reliability of our clinical conclusions is limited by the single-center, small-sample studies; therefore, further validation through multicenter studies with larger sample sizes is imperative. Additionally, only male mice were used in the mice study, while female individuals were involved in the human analyses; therefore, additional validation of this pathway is required in female mice.

## Methods

A detailed description of the Methods is provided in the supplemental materials.

### Sex as a biological variable.

Our study exclusively examined male mice. It is unknown whether the findings are relevant for female mice.

### Statistics.

Data are reported as the mean ± SD or the median (IQR range, 25th–75th percentiles). We used the Shapiro-Wilk, Kolmogorov-Smirnov, or q-q-plots for normality and log normality tests. For comparisons, normally distributed data were analyzed by unpaired, 2-tailed Student *t* test (2-group analysis), and 1- or 2-way ANOVA with Turkey’s test was used to compare multiple data groups affected by 1 or 2 independent variables, respectively. Data sets that did not follow a normal distribution were analyzed by Mann-Whitney *U* test (2-group analysis) or Dunn’s multiple-comparison test (multiple-group analysis). All statistical analyses were conducted using GraphPad Prism 9.0 (GraphPad Software). A *P* value of less than 0.05 was considered significant. A Cox regression model of S100a9K26la levels for events of survival or death, estimated HR, 95% CIs, and *P* values were calculated. The optimum cutoff of S100a9K26la for discerning MI/R patients and events of survival or death was assessed by receiver-operating curve (ROC) analysis. Kaplan-Meier survival curves (log-rank test) were used for overall survival analysis using SPSS version 26.0 (IBM Corp.). The correlation between S100a9K26la levels and other molecules was analyzed by Spearman’s test. A linear regression line (red line) with 95% CIs (red area) is shown (*P* value from the unpaired, 2-sided *t* test).

### Study approval.

All human studies were approved by the Research Ethics Committee of the Second Affiliated Hospital of Harbin Medical University Heilongjiang, China (approval KY2023-055). All animal experiments in this study were approved by the Research Ethics Committee of the Second Affiliated Hospital of Harbin Medical University Heilongjiang, China (approval YJSDW2022-167).

### Data availability.

The supporting data for the findings of this study are available from the corresponding authors upon request. Transcriptome data can be accessed from the Gene Expression Omnibus (GEO) database (GEO GSE221740). MS proteomics data from this study have been deposited in the iProX database (https://www.iprox.cn//page/SCV017.html?query=IPX0006753000) under accession number IPX0006753000. The values for all data points in the graphs are reported in the Supporting Data Values file.

## Author contributions

XW, XY, and GM performed study concept and design and reviewed and revised the manuscript. XW, XY, YC, JS, and SL developed the study methodology and performed investigations and validation. XW, XY, DW, PS, WS, ZJ, and PZ performed investigations and validation. XW, XY, ZT, PW, JY, JC, QY, and YZ acquired data, analyzed and interpreted data, and performed statistical analysis. GM, MS, ML, NW, and TC provided technical and material support. MZ, YJ, GM, XW, and BY supervised the study and handled project administration. The order of the co–first authors names was determined by the volume of work each contributed to the study. All authors read and approved the final version of the manuscript.

## Funding support

- National Natural Science Foundation of China (82270525, 82202272, and 82470521).
- Key Research and Development Program of Heilongjiang Province (GA23C006).

## Supplementary Material

Supplemental data

Unedited blot and gel images

Supporting data values

## Version 1 10/09/2025

In-Press Preview

## Version 2 12/15/2025

Electronic publication
