# Supplementary material for: S100a9 lactylation triggers neutrophil trafficking and cardiac inflammation in myocardial ischemia/reperfusion injury
Source: J Clin Invest. 2025 Oct 9;135(24):e194664. doi: 10.1172/JCI194664 (PMC12700540; doi:10.1172/JCI194664)
Supplement: Supplemental data [file jci-135-194664-s186.pdf]

# Supplementary Materials

## Supplementary Methods

### Animals

Our study exclusively examined male mice. It is unknown whether the findings are relevant for female mice. C57BL/6J mice were purchased from Beijing Vital River Laboratory Animal Technology (Beijing, China). We used 8-12 weeks old male mice in all experiments. *Padi4*$^{-/-}$, *S100a9*$^{-/-}$ and S100a9K26R mutant knock in mice in C57BL/6 backgrounds were purchased from Cyagen Biosciences lnc (Suzhou, China). All the mice were housed at the Second Affiliated Hospital of Harbin Medical University animal facility on a 12-hour/12-hour light/dark cycle with free access to normal food and water. All animal experiments have been approved by the Research Ethics Committee of the Second Affiliated Hospital of Harbin Medical University (Heilongjiang, China). The animal care and surgery protocols complied with the Principles of Animal Care provided by the National Society for Medical Research and the Guide for the Care and Use of Laboratory Animals (NIH publication).

Genotype of mice with point mutation p.K26R at S100a9 locus were as follows:

-TCATCGACACCTTCCATCAATACTCTAGG(**AGG**)GAAGGACACCCTGAC-

-TCATCGACACCTTCCATCAATACTCTAGG(**CAG**)GAAGGACACCCTGAC-

### Animal Models and Treatments

### Mice Myocardial I/R (MI/R) Model

Specifically, in the ischemia/reperfusion experiments, all mice were anesthetized with 2% isoflurane, intubated, and ventilated with a MiniVent mouse ventilator (Hugo Sachs Elektronik; stroke volume, 250 μL; respiratory rate, 105 breaths per minute). Following left thoracotomy between the fourth and fifth ribs, ischemia was achieved by ligating the LAD using a 7-0 silk suture around fine PE-10 tubing with a slipknot. Complete occlusion of the vessel was confirmed by the presence of myocardial blanching in the perfusion bed. After occlusion for 45 min, reperfusion was initiated by releasing the ligature and removing the PE-10 tubing, the chest was closed in layers and the mice were allowed to recover. Sham-operated animals were subjected to the same surgical procedures, except that the suture was passed under the LAD artery, but not tied. For S100a9K26la blocking, customized anti-S100a9K26la (pAB) or isotype control IgG antibody were administrated to WT mice as previously described, at the optimal dose (100 μg/mouse) via the angular vein at 1 h before MI/R, and, 4h, 1d, 2d and 3d after MI/R. The polyclonal antibody recognizing S100a9 K26 lactylation (anti-S100a9K26la) was generated using a K26-lactylated S100a9 peptide (QYSR-lactylK-EGHPDTC, PTM BIO). α-Lipoic Acid (HY-N0492, 30mg/kg, MedChemExpress) was injected to mice at 12 h before MI/R, and 4h after MI/R.

**Cell Isolation and Culture**

Mice neutrophils were isolated from mice bone marrow or peripheral blood using Neutrophil Isolation Kit (Miltenyi Biotec) and incubated cultured in RPMI 1640 supplemented with 10% fetal bovine serum (FBS) and penicillin-streptomycin (100 U/ml) in a humidified incubator at 37°C with 5% CO2. HEK293T (ATCC, CCL11268) cells were maintained in DMEM

supplemented with 10% heat-inactivated FBS. 32Dcl3 cells (kindly provided by Dr. Jiaqi Jin, ATCC, CRL-3594) were maintained in RPMI 1640 supplemented with 10% FBS and IL-3 (5 ng/ml; PeproTech). Induction of granulocytic differentiation was performed with removal of IL-3 by three washes with PBS and addition of recombinant murine G-CSF (100 ng/ml; PeproTech). Cells were harvested for western blot analysis 4 days post induction.

Cellular transfection of siRNAs and plasmid were performed using Lipofectamine 3000 (Invitrogen, USA) according to the manufacturer's instructions. The corresponding sequences were listed in Table S7. For the indicated experiments, mouse neutrophils were treated with recombinant LPS (L2880, Sigma-Aldrich, USA), sodium lactate (S108838, Aladdin, China), lactate dehydrogenase inhibitor sodium oxamate (Aladdin, China), PMA (P8139, Sigma-Aldrich, USA), DNase I (10104159001, Sigma-Aldrich, USA), α-Lipoic Acid (HY-N0492, MedChemExpress, China).

Human neutrophils were isolated from peripheral blood by dextran sedimentation prior to centrifugation on a Ficoll-Hypaque gradient. Remaining erythrocytes were then lysed for 10 min in red blood cell lysis buffer for western blot analysis.

**Antibodies**

The following antibodies were used in this study: anti-Ly6G (65078-1-Ig, Proteintech), anti-Pan Kla (PTM1401, PTMBio), anti-Caveolin-1 (66067-1-lg, Proteintech), anti-β-actin (TA-09, ZSGB-BIO), anti-histone H3 (citrulline R2+R8+R17, ab5103, Abcam), anti-VDAC-1 (55259-1-AP, Proteintech), anti-GAPDH (TA-08, ZSGB-BIO), anti-Histone H3 (ab1791, Abcam), anti-Importin β1 (ab313370, Abcam), anti-DLAT (ab172617, Abcam), anti-Lamin

B1 (AC057, ABclonal), anti-Phosphotyrosine (PTM-701, PTMBio), anti-Crotonyllysin (PTM-501, PTMBio), anti-Acetyllysine (PTM-101, PTMBio), anti-Lipoacylation (ab58724, Abcam), anti-Flag (T0003, Affinity), anti-S100a9 (ab234989, Abcam), anti-S100a8 (ab92331, Abcam), anti-MPO (A22900, ABclonal), rabbit IgG (ab172730, Abcam), rat IgG (ab136125, Abcam, USA), cTnI (ab47003, Abcam), anti-CD45 PE-Cy7 (103114, Biolegend), anti-CD45 BV650 (103151, Biolegend), anti-CD11b(MAC-1) PerCP-Cy5.5 (101227, Biolegend), anti-Ly-6G APC-Cy7 (127652, Biolegend), anti-Ly-6C FITC (128006, Biolegend), anti-Ly-6G PE (127608, Biolegend), anti-Ly-6C APC (128015, Biolegend, USA), anti-CXCR2 APC (149613, Biolegend), anti-CXCR1 APC (FAB8628A-025, R&D Systems), anti-CD45.1 APC (110713, Biolegend), anti-CD45.2 FITC (109805, Biolegend), anti-MHC II Pacific Blue™ (116421, Biolegend), anti-CD11c PE-Cy7 (117317, Biolegend), anti-CD64 BV711 (39311, Biolegend), anti-CD4 FITC (100406, Biolegend), anti-CD8 APC (155006, Biolegend), anti-LFA-1 (H155-78, Biolegend), Goat Anti-Rabbit IgG H&L (Alexa Fluor® 488, ab150081, Abcam), Goat Anti-Rat IgG H&L (Alexa Fluor® 555, ab150158, abcam, USA), Alexa Fluor® 647 Conjugation Kit (Fast) - Lightning-Link®° (ab269823, abcam, USA), anti-Mac-1–blocking antibody (M1/70, HY-P99121, MedChemExpress), an anti-LFA-1 –blocking antibody (M17/4, HY-P990801, MedChemExpress), Rat IgG2b kappa (HY-P990682, MedChemExpress).

Anti-S100a9K26la synthesized by PTM Bio (Hangzhou) Ltd. was used to detect S100a9K26 lactylation (CM2022012501, PTMBio). The antigenic peptides were a conserved sequence within amino acids (HQYSR-(lactyl)K-EGHPDTLC) in human and mouse. The detailed information was shown in Supplementary Figure 2.

**Adenovirus Gene Transfer**

For investigating the role of S100a9K26 lactylation, recombinant Adenovirus packing S100a9$^{K26WT\text{-}Flag\text{-}EGFP}$, S100a9$^{K26R\text{-}Flag\text{-}EGFP}$, and S100a9$^{K26Q\text{-}Flag\text{-}EGFP}$ were purchased from HANBIO (ShangHai, China). 32Dcl3 cells differentiated neutrophils (1x$10^5$ cells/well) were treated with the control virus, S100a9$^{WT\text{-}Flag\text{-}EGFP}$, S100a9$^{K26R\text{-}Flag\text{-}EGFP}$ or S100a9$^{K26Q\text{-}Flag\text{-}EGFP}$ (1 × $10^{11}$ PFU). Ad vectors were labeled with a green fluorescent protein (GFP) dye, therefore, after 24h viral transfection, the transfection efficiency was detected by fluorescence microscopy and flow cytometry.

**Bone Marrow Transplantation**

Recipient mice were given 100mg/L neomycin 2 weeks before and after BMT. Eight-week-old C57BL/6J male mice (CD45.1) were subjected to lethal total body irradiation with a total dose of 800 cGy and transplanted with BM (5x$10^6$ cells) from donor WT or S100a9K26R mutant mice (CD45.2) and re-suspended in PBS were administered to each recipient mouse via the tail vein. The mice were used for experiments 6 weeks after bone marrow transfer. MI/R was induced as described above. The recipient mouse received S100a9K26R mutant mouse bone marrow was injected rS100a9 (ab109951, Abcam, 1.5mg/kg) 24h after MI/R for rescue experiments.

**Adoptive transfer (AT) studies.**

For studies involving adoptive transfers, neutrophils and non-neutrophils from the BM of donor mice (CD45.2) were isolated using neutrophil isolation kits (Miltenyi Biotec) according to the manufacturer's instructions. Recipient mice (CD45.1) were subjected to MI/R. One hour after the MI/R procedure, 5.0×10^6 bone marrow neutrophils and non-neutrophils from WT or S100a9K26R mice were transferred to the recipient mice by retro-orbital injection. Echocardiogram was performed one week after the MI/R to determine cardiac function.

**Echocardiography**

The mice were anesthetized with 2% isoflurane. Fix the mice in a supine position on a temperature-controlled electrocardiogram board and monitor its heart rate. Two-dimensional and M-mode echocardiographic images were obtained in the parasternal long-axis view using a high-resolution in vivo VIVID E9 imaging system (GE Healthcare, USA). 2-dimensional guided M-mode was used for evaluating cardiac function (2). The left ventricular inner diameters, including left ventricular internal diameter at end-diastole (LVIDd) and left ventricular internal diameter at end-systole (LVIDs), as well as left ventricular ejection fraction (EF) and fractional shortening (FS) were measured and calculated from three independent cardiac cycles.

**Histological Studies**

Histological analysis of heart slices was performed at different time points. The heart sample was immersed in 4% paraformaldehyde at 4°C for 24 h. After dehydration through an alcohol

gradient, clearing, and embedding in paraffin wax, the sliced sections (4μm) were stained with hematoxylin and eosin (HE) or Masson's trichrome stain. Masson staining was used to analyze myocardial fibrosis. HE stains to assess the infiltration of cardiac inflammatory cells.

**Immunofluorescence and TUNEL staining**

For immunofluorescence, tissues were embedded within an optimal cutting temperature (Tissue-Tek) and cut to 6μm. The sections were blocked with 5% bovine serum albumin (BSA) for 30 minutes and stained overnight at 4 °C with the following primary antibodies: anti-S100a9K26la (CM2022012501, PTMBio, China), anti-Ly6G (65078-1-Ig, Proteintech, China), anti-Pan Kla (PTM1401, PTMBio, China), anti-Caveolin-1 (66067-1-lg, Proteintech, China), anti-MPO (A22900, ABclonal), anti-histone H3 (citrulline R2+R8+R17, ab5103, Abcam), then incubated with the secondary antibodies at 37°C for 1 hour. Cell nuclei were counterstained with 4',6-Diamidin-2'-phenylindole dihydrochloride (DAPI, Beyotime, China). Isotype controls were performed using rabbit IgG (ab172730, Abcam, USA), rat IgG (ab136125, Abcam, USA) at concentrations the same as the primary antibodies. Secondary antibody controls were performed to distinguish genuine target staining from background. Sections were analysed with a confocal microscope (Zeiss, LSM 800). Cardiomyocyte apoptosis was determined by terminal deoxynucleotidyl transferase dUTP nick end labeling (TUNEL) and cTnI double staining. TUNEL staining was conducted using TUNEL Apoptosis Detection Kit (FITC, 40306ES50, Yeasen Bio, China) according to the manufacturer's instructions. Briefly, after the sections were blocked and permeabilized,

samples were stained with TUNEL staining mixture at 37 °C for 1 h, followed by the cTnI (ab47003, Abcam, USA) and secondary antibody staining.

**Flow Cytometry**

The heart was extensively rinsed and then removed. Use scissors to mince the heart tissue and digest it with collagenase II (450 U/mL) and trypsin (0.25%) at 37°Cand 750 rpm for 1 hour. The tissue suspension was centrifuged at 1000 rpm for 5min, and subsequently treated with erythrocyte lysis buffer, then the cells were centrifuged at 1500 rpm for 8 min, resuspended in 100μl PBS to obtain cell suspensions. The bone marrow from the tibia of the mice was rinsed with PBS, and collected all bone marrow tissue suspensions to centrifuge at 1000 rpm for 5min, and subsequently treated with erythrocyte lysis buffer, then the cells were centrifuged at 1500 rpm for 8 min, resuspended in 100μl PBS to obtain cell suspensions. Blood sample was treated with red blood cell lysis buffer (eBioscience) to eliminate red blood cells. The prepared cell suspensions were stained in PBS with the following fluorochrome-labelled anti-mouse antibodies: anti-CD45 PE-Cy7 (103114, Biolegend), anti-CD45 BV650 (103151, Biolegend), anti-CD11b(MAC-1) PerCP-Cy5.5 (101227, Biolegend), anti-Ly-6G APC-Cy7 (127652, Biolegend), anti-Ly-6C FITC (128006, Biolegend), anti-Ly-6G PE (127608, Biolegend), anti-Ly-6C APC (128015, Biolegend, USA), anti-CXCR2 APC (149613, Biolegend), anti-CXCR1 APC (FAB8628A-025, R&D Systems), anti-CD45.1 APC (110713, Biolegend), anti-CD45.2 FITC (109805, Biolegend), anti-MHC II Pacific Blue™ (116421, Biolegend), anti-CD11c PE-Cy7 (117317, Biolegend), anti-CD64 BV711 (39311, Biolegend),

anti-CD4 FITC (100406, Biolegend), anti-CD8 APC (155006, Biolegend), anti-LFA-1 (H155-78, Biolegend).

For intracellular S100a9K26la content in neutrophils, the cell suspensions were stained in PBS with the following fluorochrome-labelled anti-mouse antibodies: anti-CD45 BV650, anti-CD11b PerCP-Cy5.5, anti-Ly-6G PE, anti-Ly-6C APC, anti-MHC II Pacific Blue™ , anti-CD11c PE-Cy7, and anti-CD64 BV711, then fixed and infiltrated with the Phosflow kit (Thermo Fisher Scientific, USA), and stained with anti-S100a9K26la (CM2022012501, PTMBio, China) to evaluate S100a9K26la levels in gated neutrophils ($CD45^+CD11B^+Ly6G^+$), gated ly6C$^{hi}$ monocytes ($CD45^+CD11B^+Ly6G^-CD64^+Ly6C^+$) and dendritic cells ($CD45^+CD11B^+Ly6G^-CD64^-MHCII^+CD11C^+$) populations. In addition, the conjugated secondary antibody (Goat Anti-Rabbit IgG H&L (Alexa Fluor® 488), ab150081, abcam, USA) was used for indirect flow cytometry analysis to measure S100a9K26la. Flow cytometry data were determined using a FACSCanto II flow cytometer (BD Biosciences, CA, USA).

**Mouse cardiomyocyte isolation**

Mouse neonatal cardiomyocytes (NCMs) were isolated from newborn mice (1–3 days old). Briefly, harvested hearts were cut into 1–2 mm pieces in 1×ADS buffered saline solution (10×ADS 100mL: NaCl 6.8g, HEPES 4.76g, $Na_2HPO_4$ 0.138g, Glucose 0.6g, KCl 0.4g, $MgSO_4$ 0.102g, $H_2O$ 100mL, and pH 7.35–7.45). Then, tissues were digested using 1×ADS buffer containing 0.4mg/mL collagenase II (LS004177, Worthington, USA) and 0.6mg/mL pancreatin (P7545, Sigma-Aldrich, USA) on a rotary shaker at 37 °C for several rounds lasting seven minutes each. Cells were filtered through a 70 μm cell strainer and cell

suspensions were centrifuged at 1000 rpm for 5 min and resuspended in DMEM supplemented with 10% fetal bovine serum, and 1% penicillin-streptomycin. Cells were allowed to attach for 20 min, then NCMs were collected and maintained in DMEM containing 10% FBS and 1% penicillin/streptomycin.

Mouse adult cardiomyocytes (ACMs) were isolated according to our previously published study. Briefly, adult male C57BL/6N mice aged 8 to 10 weeks were anesthetized, and the chest cavity was opened to expose the heart completely. The inferior vena cava and descending aorta were excised, and an EDTA buffer was injected into the right ventricle. The hearts were then removed and perfused with a collagenase buffer containing type II and IV collagenase. Once the tissues appeared slightly pale and flaccid, the left ventricles were minced into 1-mm3 fragments using a stop buffer. Subsequently, extracellular $Ca^{2+}$ levels were gradually restored to 1.2 mM. The attainment of a minimum yield of 80% rod-shaped CMs was considered to be indicative of success.

**Measurement of the mitochondrial membrane potential**

NCMs were harvested before suspension and incubated in media containing 50 M of the red fluorescent dye tetramethylrhodamineethyl ester for 30 minutes at 37°C in the dark. Fluorescence was measured by flow cytometry using a BD FACS Aria II. Mitochondrial membrane potential was measured in NCMs that were gated for GFP positivity

**ATP measurements**

ATP levels were measured via ATP bioluminescence kit (HYK0314, MCE). NCMs were lysed by incubation with lysis buffer. 10 μL of standard ATP solution (final concentration ranged from 100–5 μM), lysed NCMs were added to 96-wells plates, and 90 μL of reaction buffer was added to each well. Measurements were performed with a plate reader (absorbance of 560 nm). ATP concentrations were calculated using a standard curve.

**Assessment of neutrophil and NCM apoptosis**

An Annexin V-fluorescein isothiocyanate (FITC) Apoptosis Detection Kit (eBioscience; San Diego, CA, USA) was used according to the manufacturer's instructions. Briefly, neutrophils isolated from bone marrow or peripheral blood and NCMs were resuspended in PBS at a concentration of $1 \times 10^6$ cells/ml. Subsequently, cells were stained with FITC-conjugated annexin V and PI for 15 min at room temperature. Flow cytometry data were determined using a FACSCanto II flow cytometer (BD Biosciences, CA, USA).

**Transwell migration assay for neutrophils**

Isolated neutrophils were resuspended in serum-free RPMI-1640 medium at $10^6$/ml. Thereafter, 200 μl of cells were seeded in the upper well of a Transwell chamber with microporous filters (3-μm pores, Millpore; Billerica, MA, USA), and 500 μl serum-free RPMI-1640 medium in the presence of CXCL2 (30 ng/ml, R&D Systems) was added into the bottom chamber. After 2 h, cells that migrated through the membrane were stained with anti-Ly-6G APC-Cy7 (127652, Biolegend, USA) and determined using a FACSCanto II flow cytometer (BD Biosciences, CA, USA).

**Neutrophil polarization**

Neutrophils isolated from bone marrow were seeded in 96-well plates at a density of 1 × $10^4$ cells/well. Neutrophils were treated with recombinant LPS for 4h in a humidified incubator at 37°C with 5% CO2. Images were captured at Inverted fluorescence microscopy, and quantification of the proportion of polarized neutrophils through the ratio of the number of polarized neutrophils to the total number of neutrophils in each well. Five independent experiments were performed.

**Neutrophil Adhesion Assay**

In brief, confluent CMEC cells seeded in a 96-well plate were stimulated with or without murine TNF-α (tumor necrosis factor alpha; 20 ng/mL) for 4 hours. Murine neutrophils (2×106 per mL) were stained with calcein AM (2 μmol/L) for 20 minutes in the dark. After washing with D-Hank buffer, neutrophils were added into the prepared 96-well plates in the presence or absence of Mg2+ (1 mmol/L). After incubation in a CO2 incubator for 20 minutes, the fluorescence in each well was measured at 485/520 nm (Excitation/Emission) on a plate reader. After washing with D-Hank buffer 3×, the fluorescence was measured again at the same wavelength. The percentage adhesion of neutrophils was calculated as the ratio of the second readout to the first one.

**NET formation and preparation**

Freshly isolated neutrophils were seeded on six-well plates ($1\times10^6$/well) and stimulated with PMA (P8139, Sigma-Aldrich, USA), or DNase I (10104159001, Sigma-Aldrich, USA) for 4 hours. Subsequently, the cell culture medium was carefully discharged via slow suction and washed twice without disturbing NETs. Serum-free RPMI 1640 (2 mL) was added to blow the cells down. The supernatant containing NETs was transferred to 15 mL centrifuge tubes and centrifuged at 2400 rpm for 10 min to eliminate cell debris. Isolated NETs were stored at −80°C for further use. The suspension containing NETs or cell culture medium were used to treat NCM cells.

**ELISA**

The peripheral blood of mice and human was collected and centrifuged with a centrifugal force of 3000 rpm for 10 minutes to remove sediment. The levels of IL-1β, IL-6, TNF-α, and H3cit in mice plasma were determined according to the ELISA instructions (Multi Sciences, China). The levels of S100a9 in human plasma were determined according to Human S100A9 ELISA Kit (Multi Sciences, China). The levels of S100a9 in mice plasma were determined according to Mouse S100A9 DuoSet ELISA kit (DY2065, R&D Systems, Minneapolis MN). The PDH activity and the acetyl-CoA level of neutrophils were determined according to the ELISA instructions (Elabscience Biotechnology, China).

S100a9K26la level was detected using an indirect ELISA protocol, and the steps are as follows:1) Dilute the antigen to a suitable concentration in Coating buffer (PBS,CST, USA), pipet 100 μl of the antigen dilution in the top wells of the plate. 2) Cover the plate with an adhesive plastic and incubate at 4° overnight; 3) Remove the coating solution and wash the

plate four times by filling the wells with 400 µl Wash buffer (CST, USA). The remaining drops are removed by patting the plate on a paper towel; 4) Block plates by adding 300µL of Reagent Diluent (CST, USA) to each well. Incubate at room temperature for a minimum of 2h. 5) Repeat the aspiration/wash as in step 3;6) Add 100 µl of diluted primary antibody to each well and incubate for 1.5 h at 37°C(Anti-S100A9K26la dilution ratio is 1:2000 in Reagent Diluent); 7) Repeat the aspiration/wash as in step 3; 8) Add 100 µl of conjugated secondary antibody and incubate for 30min at 37°C, avoid placing the plate in direct light (Goat-anti-Rabbit (abcam, USA) dilution ratio is 1:10000 in Reagent Diluent); 9) Wash the plate five times with Wash buffer; 9) Add 100µL of Substrate Solution to each well. Incubate for 10 minutes at room temperature. Avoid placing the plate in direct light; 10) Add 50µL of Stop Solution to each well. Gently tap the plate to ensure thorough mixing; 11) Determine the optical density of each well immediately, using a microplate reader set to 450 nm.

**Mitochondrial Oxygen Consumption and Extracellular Acidification Rate**

OCR and ECAR were analyzed using an XF24 Extracellular Flux Analyzer (Seahorse Biosciences) according to the manufacturer's instructions. Neutrophils treated with LPS were seeded at $8*10^5$ cells/well. Cells were cultured for the indicated time followed by incubating with the assay medium. Mitochondrial oxygen consumption was measured under basal conditions throughout treatment with addition of oligomycin (1µM), FCCP (1µM) and rotenone/antimycin A (0.5µM) to assess maximal mitochondrial respiration. Extracellular acidification rate was measured under basal conditions throughout treatment with addition of

glucose (10mM), oligomycin (1µM) and 2-Deoxy-DGlucose (50 mM) to assess glycolytic capacity.

**Cytoplasmic and nuclear extraction of cells**

Specifically, nucleocytoplasmic fractionation was performed using the Minute Cytoplasmic and Nuclear Extraction Kit for Cells (Invent Biotechnologies Inc., Plymouth, MN, USA). A total of 1 x 106 of neutrophils were isolated from mouse bone marrow using Neutrophil Isolation Kit (Miltenyi Biotec). To prepare total cell extracts, neutrophils were harvested by centrifugation, washed in PBS, and 300 µL of cytoplasmic extraction buffer was added to the cells, and the tissue culture was placed on ice for 5 min. Then, the cell lysate was transferred to pre-chilled 1.5 mL microcentrifuge tube. After vigorously vortexing the tube for 15 s, the tube was centrifuged for 5 min at top speed in a microcentrifuge at 4 °C. Afterward, the supernatant (cytosolic fraction) was transferred to a fresh pre-chilled 1.5 mL tube, and 150 µL of nuclear extraction buffer was added to the pellet, the tube was vortexed vigorously for 15 s, and then incubated on ice for 1 min. Then, the 15 s vortexing and 1 min incubation on ice steps were repeated 4 times. Immediately afterward, the nuclear extract was transferred to a pre-chilled filter cartridge with a collection tube and centrifuged at high speed (14,000–15,000 g) in a microcentrifuge for 30 s. After the centrifugation, the filter cartridge was discarded, and the nuclear extract was stored at 80 °C until use. All fractions were collected for subsequent Western blot analysis.

**Western Blotting**

First extract the protein according to the conventional method. Samples were separated by SDS-PAGE and transferred to polyvinylidene fluoride membrane (PVDF). PVDF membrane was blocked by 5% milk and incubated with the corresponding primary antibody overnight at 4°C. After washing with phosphate buffered saline with 0.05% Tween-20 (PBST) buffer three times, membranes were incubated with horseradish peroxidase (HRP)-conjugated second antibody at room temperature for 1 hour. The band signals were visualized using Tanon Image software (version 5100, Tanon, Shanghai, China). β-actin was used as an internal control, then calculated as fold changes vs the control groups. Primary antibodies were anti-S100a9K26la (CM2022012501, PTMBio), anti-Ly6G (65078-1-Ig, Proteintech), anti-Pan Kla (PTM1401, PTMBio), anti-β-actin (TA-09, ZSGB-BIO), anti-VDAC-1 (55259-1-AP, Proteintech), anti-GAPDH (TA-08, ZSGB-BIO), anti-Histone H3 (ab1791, Abcam), anti-Importin β1 (ab313370, Abcam), anti-DLAT (ab172617, Abcam), anti-Lamin B1 (AC057, ABclonal), anti-Phosphotyrosine (PTM-701, PTMBio), anti-Crotonyllysin (PTM-501, PTMBio), anti-Acetyllysine (PTM-101, PTMBio), anti-Lipoacylation (ab58724, Abcam), anti-Flag (T0003, Affinity), anti-S100a9 (ab234989, Abcam), anti-S100a8 (ab92331, Abcam).

**Co-Immunoprecipitations Assay**

The total protein from cell lysate was immunoprecipitated. Firstly, the extract was incubated with anti-Flag (T0003, Affinity Biosciences, USA), anti-S100a9 (ab234989, Abcam, USA), anti-S100a9K26la (CM2022012501, PTMBio, China), and anti-Importinβ1 (ab313370, Abcam) for 24 h at 4 °C. The protein A/G magnetic beads were added and further incubated overnight at 4 °C followed by centrifugation at 12000 rpm for 5 min. Then recovered the

precipitate for washing, resuspended it in 40μl SDS lysis buffer and boiled for 5 minutes, and finally analyzed the precipitate by immunoblotting with the indicated antibody.

**Immunoprecipitation coupled with mass spectrometry assay**

The antigen-antibody-magnetic bead complex was obtained as described above. Then the mass spectrometry (PTM Bio, China) was conducted to evaluate the isolated immunoprecipitates. Briefly, the extract was incubated with anti-S100a9K26la or rabbit IgG and then incubated with magnetic beads overnight at 4 °C. 0.1% trifluoroacetic acid was used to elute the beads-bound peptide and then desalted with C18 ZipTips (Millipore). The peptides were further separated by a NanoElute ultrahigh performance liquid system. Samples subjected to NSI source for ionization followed by tandem mass spectrometry in Q Exactive Plus (Thermo Scientific).

**In vitro lactylation assay**

200 ng of S100a9 substrate was mixed with 50ng DLAT acetyltransferase in HAT buffer (40 mM Tris-HCl pH 8.0, 75 mM potassium chloride (KCl), and 10 uM lactyl -CoA) in a final volume of 30 uL for 45 minutes at 30°C, and terminated by the addition of SDS-PAGE sample buffer and heating the samples to 95°C for 5 minutes. Lactylated proteins were visualized by SDS-PAGE.

**The cellular thermal shift assay (CETSA)**

The DLAT plasmid was transiently transfected into HEK293T cells as instructed by the manufacturer. After transfection for 8 h, the cells were collected to prepare the cell lysates and then incubated with ALA, lactyl-CoA or acetyl-CoA for 4 h, digested and counted, and then resuspended in PBS with 1 mM PMSF, with a final density of 4 × 107 cells/ml. The cell lysates were divided into 10 parts on average, and the 3 min was heated with a thermal gradient from 37 °C to 64 °C, then collected for Western blotting.

**Surface Plasmon Resonance (SPR)**

SPR technology-based binding assays were performed using a Biacore T200 instrument (GE Healthcare) with running buffer HBS-EP (10 mM HEPES (pH 7.4), 150 mM NaCl, 3 mM EDTA, and 0.5% (v/v) surfactant P20,5% DMSO) at 25 °C. The DLAT were covalently immobilized onto sensor CM5 chips by a standard amine-coupling procedure in 10 mM sodium acetate (pH 4.5). Compounds were serially diluted and injected onto a sensor chip at a flow rate of 30 μl/min for 120 s (contact phase), followed by 120 s of buffer flow (dissociation phase). The KD value was derived using Biacore T200 Evaluation software Version 1.0 (GE Healthcare) and steady state analysis of data at equilibrium.

**Molecular Docking**

The three-dimensional (3D) structure of DLAT and Lactyl-CoA were downloaded from PDB database (https://www1.rcsb.org/). The active center of POCASA v1.1 predicted protein (center x = -13.1, center y = -0.5, center z = 3.0) was used, and docking boxes with the length, width and height of 40Å were set, and molecular docking was performed using AutoDock

Vina v1.1.2. According to the minimum energy and molecular conformation principle, the optimal molecular docking structure model was selected. The docking score of lactyl-CoA and DLAT was -6.5 kcal/mol.

**Molecular Dynamics**

Molecular dynamics simulation was performed using Gromacs2022 software. In order to restore the real experimental environment, Charmm 36 and Gaff 2 were selected as the protein force field and the ligand force field, respectively, while TIP 3 P water model was selected to add solvent to the protein ligand system. Then, a water box with periodic boundary of 1.2nm was established, and sodium ions and chloride ions were added to equilibrize charges in the system. After assignment of force field to the protein and a series of energy minimizations, the simulation was performed

**RNA Extraction, Reverse Transcription and Quantitative RT-PCR**

Total RNA was extracted using TRIzol™ reagent (Invitrogen, Carlsbad, CA, USA) and reverse-transcribed using Transcriptor First Strand cDNA Synthesis Kit (Roche Diagnostics, Risch-Rotkreuz, Switzerland) following the manufacturer's protocol. Quantitative real-time PCR was performed using Fast Start Universal SYBR® Green Master Mix (Roche Diagnostics). The mRNA expression levels detected in each sample were normalized to β-actin levels. The primers were listed in Table S5.

**RNA-Seq**

Extract RNA with Trizol reagent. Use TruSeq Stranded mRNA LT Sample Prep Kit (Illumina, San Diego, CA, USA) to construct RNA-seq library. OE Biotechnology Co. Ltd (Shanghai, China) is responsible for transcriptome sequencing and analysis. Pvalue < 0.05 and |log2(Fold Change)| > 0.58 were set as threshold for significant differential expression. Volcano plot depicts the distribution of differentially expressed genes (DEG). Use DAVID for gene ontology analysis.

**CUT&Tag**

In brief, blood neutrophils from sham (pooled n = 4 mice) and MI/R (pooled n = 4 mice, day 1) were harvested, counted and centrifuged for 3 min at 600×g at room temperature. $1 \times 10^5$ cells were washed twice in 500μL wash buffer by gentle pipetting. Concanavalin A coated magnetic beads were prepared as the kit manual described, 10 μL of activated beads were added per sample and incubated at RT for 10 min. According to the kit instructions, cells were sequentially incubated with ConA Beads, primary antibody (anti-S100a9K26La, CM2022012501, PTMBio, China, and anti-S100a9 ab234989, Abcam, USA), secondary antibody (Goat anti-Rabbit IgG H&L, AB206-01-AA) and Hyperactive PG-TN5 / PA-TN5 Transposon and then fragmented. The fragmented DNA was extracted from the samples and amplified by PCR. CUT&Tag libraries were constructed using HyperactiveTM In-Situ ChIP Library Prep Kit (TD901, Vazyme, China) and sequenced on an Illumina NovaSeq platform, and 150-bp paired-end reads were generated. Fastp v 0.20.0 was used to remove adapter and low-quality reads. Align paired-end reads using Bowtie2 version 2.3.4.3 with options: --end-to-end –sensitive. Duplicated reads are removed using Picard v2.18.17 with this

parameter: REMOVE_DUPLICATES = true. Peak calling uses SEACR v1.3 with threshold: 0.01. Scatterplots, correlation plots, and heatmaps are displayed using deepTools v2.27.1. Annotation of peaks is performed using the R package ChIPseeker v1.12.1. MEME-ChIP v 5.0.5 was used to search for the binding site. Peaks with |M-value| > 0.45 and P value < 0.05 are defined as specific peaks.

**Chromatin immunoprecipitation (ChIP)-qPCR**

Chromatin immunoprecipitation was performed using the SimpleChIP Enzymatic Chromatin IP Kit (Cell Signalling Technology, 9003S) according to the manufacturer's instructions. In brief, cells were cross-linked with 1% formaldehyde (Sigma) for 10 min at room temperature and quenched with 0.125 M of glycine for 5 mins. Then the cells were washed three times with pre-iced PBS and centrifuged at 2500 rpm for 5 min. Cell lysis, sonication and immunoprecipitation were performed according to the manufacturer's instructions. Then pre-cleared DNA was immunoprecipitated with S100a9K26la (CM2022012501, PTMBio, China), S100a9 antibody (ab234989, Abcam, USA) or the IgG as nonspecific control. The precipitated chromatin DNA was purified and detected by qPCR using specific primers shown in Table S6.

**Proteomic Analysis**

**Procedures for Proteomic Experiment**

The proteomic experiment was done by Jingjie PTM BioLab (Hangzhou), Co. , Ltd in China. In brief, bone marrow neutrophils from sham (pooled n = 5 mice) and MI/R (pooled n = 5

mice, day 1) were collected, and the protein was extracted for trypsin digestion. The four samples were dissolved in NETN buffer (100 mmol/L NaCl, 1 mmol/L EDTA, 50 mmol/L Tris-HCl, 0.5% NP-40, pH 8.0) and incubated with prewashed Kla antibody beads (PTM-1404, PTMbio, China) at 4°C overnight with gentle shaking for lactylation proteomic experiment. 0.1% trifluoroacetic acid was used to elute the beads-bound peptide and then desalted with C18 ZipTips (Millipore). The Kla enriched peptides were further separated by a NanoElute ultrahigh performance liquid system. Mobile phases A (2% acetonitrile in HPLC water) and B (100% acetonitrile in HPLC water) were used. The solvent gradient was set as follows:0-42 min, 7%~24%B, 42-54 min, 24%~32%B, 54-57 min, 32%~80%B, 57-60 min, 80%B. For the lactylation-free proteomic, the solvent gradient was set as follows:0-70 min,6%~24%B;70-84 min, 24%~35%B, 84-87 min, 35%~80%B, 87-90 min, 80%B. Tryptic peptides were separated at a fluent flow rate of 450nL/min. Samples subjected to NSI source for ionization followed by tandem mass spectrometry in Q Exactive Plus (Thermo Scientific) coupled online to the ultrahigh performance liquid system.

**Database Search**

The resulting tandem mass spectrometry data were processed using Maxquant (v1.6.15.0). The parameter setting for searching: the database was Mus_musculus_10090_(17045 sequences), a reverse decoy database was added to calculate the false positive rate caused by random matching, and a common pollution database was added to the database to eliminate the influence of contaminated proteins in the identification results. The enzyme digestion method was set as Trypsin/P. The number of missing cleavages was set to 2. The minimum length of the peptide segment was set as 7 amino acid residues. The maximum modification

number of peptide segment was set as 5. The mass tolerance for precursorions was set as 20 ppm in first search and 20 ppm in main search, and the mass tolerance for fragment ions was set as 20 ppm. Carbamidomethyl on cysteine was specified as fixed modification, and acetylation on protein N-terminal, oxidation on methionine, and lactylation on Lys were specified as variable modifications and the protein identification and PSM identified false positive rate were set as 1%. Compared to that in the sham injury group, only an average Kla enrichment or protein in the MI/R mice increase above 1.25 or below 0.8 was considered as a remarkable change and further analyzed.

**Functional Enrichment Analysis**

The protein–protein Interaction (PPI) Network was constructed on STRING website and subsequently imported into the Cytoscape software. Gene Ontology enrichment analysis of differentially expressed genes was implemented with database for Annotation Visualization and Integrated Discovery (DAVID, version 6.8).

**Study Population**

**Inclusion and Exclusion Criteria of MI/R Cohort**

23 patients with AMI who were admitted to the cardiology department of the 2nd Affiliated Hospital of Harbin Medical University (Harbin, China) were enrolled as the MI/R cohort. Patients with AMI were referred within 12 h of symptom onset for percutaneous coronary intervention (PCI). The diagnosis of AMI, including ST-segment elevation myocardial infarction (STEMI) or non-STEMI, followed the Fourth Universal Definition of Myocardial Infarction (3). STEMI was defined as persistent chest pain that lasted at least 30 minutes,

arrival at the hospital within 12 hours from symptom onset, 12-lead electrocardiogram changes (ST-segment elevation of >0.1 mV in ≥2 contiguous leads or new-onset left bundle branch block), and elevation of cardiac biomarkers (creatine kinase-MB or troponin T/I). Non-STEMI was defined as ischemic symptoms in the absence of ST-segment elevation on the electrocardiogram with elevated cardiac biomarkers.

The exclusion criteria were as follows: 1) aged ≤18 years; 2) patients with cardiogenic shock at admission; 3) those with active infections, systemic inflammatory disease, known hepatic, hematological, malignant disease, end-stage renal disease, or previous surgery in 3 months. 23 age-matched (±2 years) and sex-matched unstable angina pectoris (UA) patients was assigned as the control. The patient characteristics in the control and MI/R cohorts were listed in Table S1.

After informed consent was obtained, patients in the MI/R cohorts recruited had blood drawn from the inguinal arterial sheath after percutaneous coronary intervention (PCI) within 24h. The plasma samples were separated by centrifugation at 1000 ×g for 30 min and stored at −80 °C in a biobank until further analysis. Neutrophils were isolated by Ficoll-gradient centrifugation to generate a peripheral blood neutrophils pellet, immediately snap-frozen at -80 °C for western blot analysis.

**Inclusion and Exclusion Criteria of Cardiac Death Cohort**

This is an analysis of patients with AMI who were referred within 12 h of symptom onset and underwent PCI within 24 h at the 2nd Affiliated Hospital of Harbin Medical University (Harbin, China) between February 2017 to May 2020. In the Cardiac Death Cohort,

we retrospectively enrolled the final eligible 94 death cases and 94 survivor-matched controls according to the occurrence of cardiac death events during 1 year follow-up period. Cardiac death was defined as death from any death in which a cardiac cause could not be excluded during the follow up period (4). All death cases suffered sudden and unexpected death occurring either within 1 hour of symptom onset (if witnessed) or within 24 hours of being observed alive and symptom-free (if unwitnessed) during 1 year follow-up period after hospital discharge.

The key exclusion criteria were as follows: 1) age < 18 years old; 2) history of MI, heart failure (HF), PCI, or coronary artery bypass grafting (CABG); 3) MI caused by surgery, trauma, gastrointestinal bleeding, PCI, or their complications; 4) patients with incomplete demographic or clinical data; 5) patients with inadequate blood samples. For each cardiac death case, an age-matched (±2 years) and sex-matched survivor with complete blood samples was assigned among eligible participants who survived during the follow-up, and whenever possible, additional matching was performed for percutaneous coronary intervention and multivessel disease. All patients provided written informed consent. The patient characteristics in the Survival and Death cohorts were listed in Table S2.

After informed consent was obtained, the blood samples were prospectively collected from the arterial access site after stent implantation during interventional procedures within 24h of symptom onset and separated into whole blood, plasma, plasma and white blood cells, and then stored at -80 ℃ in a biobank until further analysis.

**Statistical Analysis**

Data were reported as mean ± standard deviation (SD) or median (interquartile ranges, 25th-75th percentile). We use Shapiro-Wilk test, Kolmogorov-Smirnov or q-q-plots for normality and lognormality tests. For comparisons, normally distributed data were analyzed by unpaired 2-tailed Student t test (two-group analysis), and one-way ANOVA or two-way ANOVA with Turkey's test was used to compare multiple data groups affected by one or two independent variables, respectively. Data sets that did not follow a normal distribution were analyzed by Mann-Whitney test (two-group analysis) or Dunn's multiple comparisons test (multiple-group analysis). Tests used to assess significance are detailed in each Figure legend. All statistical analyses were conducted using GraphPad Prism 9.0 software. $P<0.05$ was considered as significant. COX regression model of S100a9K26la levels for events of survival or death, estimated HR, 95% CIs, and p values were calculated. The optimum cutoff of S100a9K26la for discerning MI/R patients and events of survival or death was assessed by receiver-operating curve (ROC) analysis. Kaplan-Meier survival curves (log-rank test) were used for overall survival (OS) analysis using SPSS V.26.0 (IBM Corp, Armonk, NY, USA). The correlation between S100a9K26la levels and other molecules was analysed by Spearman. A linear regression line (red line) with 95% confidence intervals (red area) is shown (P value from the unpaired two-sided t test).

For animal studies, the sample size for experiments was based on power calculations, published reports, and our experience. Mice in the study were randomly allocated to experimental groups using simple random assignment. Whenever possible, the investigators were blinded to group allocation during experiments and outcome assessment. The

representative image was selected from one of the repeated experiments that best matched the mean value.

**Study approval**

All human studies were approved by the Research Ethics Committee of the Second Affiliated Hospital of Harbin Medical University Heilongjiang, China (approval #KY2023-055). All animal experiments in this study were approved by the Research Ethics Committee of the Second Affiliated Hospital of Harbin Medical University Heilongjiang, China (approval #YJSDW2022-167). Further details about Inclusion and Exclusion Criteria of MI/R Cohort and Cardiac Death Cohort are provided in the Supplementary Materials.

**Supplementary Tables and Figures**

**Supplementary Table 1: Baseline characteristics of controls and MI/R patients.**

| | Con(n=23) | MI/R(n=23) | P-value |
|---|---|---|---|
| Basic Information | | | |
| Age(years) | 63.26±10.046 | 63.52±10.246 | 0.931 |
| Male sex, n(%) | 16(69.57) | 16(69.57) | 1 |
| Clinical Profile | | | |
| Symptom onset-to-perfusion (time, h), median (IQR) | NA | 7.239(4,9.5) | |
| TIMI flow before PCI, n(%) | | | ＜0.001 |
| ≤2 | 0(0) | 22(95.65) | |
| 3 | 23(100) | 1(4.35) | |
| TIMI flow after PCI, n(%) | | | |
| ≤2 | NA | 0(0) | |
| 3 | NA | 23(100) | |
| LVEF(%), median (IQR) | 61.627(62,65) | 46.154(39,58) | 0.001 |
| Medical history, n(%) | | | |
| Hypertension | 9(39.13) | 11(47.83) | 0.552 |
| Hyperlipoidemia | 6(26.09) | 11(47.83) | 0.127 |
| Diabetes | 3(13.04) | 7(30.43) | 0.153 |

| | | | |
|---|---|---|---|
| Chronic kidney disease | 11(47.83) | 11(47.83) | 1.000 |
| Cardiac arrhythmia | 4(17.39) | 5(21.74) | 0.710 |
| Biochemistry at admission | | | |
| Total cholesterol (mmol/L) | 4.27±0.991 | 4.972±1.055 | 0.001 |
| Total glyceride (mmol/L), median (IQR) | 1.625 (0.985，2.255) | 1.745 (0.915，1.92) | 0.642 |
| HDL cholesterol (mmol/L), median (IQR) | 1.210 (0.865，1.30) | 1.129 (0.93，1.245) | 0.772 |
| LDL cholestrol (mmol/L) | 2.404±0.990 | 3.155±0.910 | 0.010 |
| NT-proBNP (pg/mL), median (IQR) | 2643.65(58，846) | 4211.13 (318，5010) | 0.004 |
| TNI (μg/L), median (IQR) | 1.211(0,0.021) | 49.14048 (4.2,89.742) | ＜0.001 |
| CKMB (μg/L), median (IQR) | 14.384(0.5,6) | 491.496(30,402) | ＜0.001 |
| Scr (μmol/L), median (IQR) | 86.22(68，99) | 92.74(70，110) | 0.644 |
| Lactate (mmol/L), median (IQR) | 1.52 (0.675，2.125) | 2.517(1.4,2.8) | 0.089 |
| Hemoglobin (g/L) | 143.3±22.792 | 140.83±19.551 | 0.694 |
| Medication usage, n (%) | | | |
| Statins | 15(65.22) | 22(95.65) | 0.009 |
| Anti-platelet therapy | 17(73.91) | 22(95.65) | 0.04 |
| ACE inhibitor | 3(13.04) | 14(60.87) | 0.001 |

| | | | |
|---|---|---|---|
| Beta blocker | 5(21.74) | 13(56.52) | 0.011 |
| Diuretics | 6(26.09) | 5(21.74) | 0.793 |

Data are expressed as mean ± SEM or median (interquartile ranges, 25th-75th percentile) or number of subjects (%). TIMI, thrombolysis in myocardial infarction; PCI, percutaneous coronary intervention; LVEF, left ventricular ejection fraction; HDL, high density lipoprotein; LDL, low density lipoprotein; NT-proBNP, N-Terminal Pro-Brain Natriuretic Peptide; TNI, troponinL; CKMB, MB fraction of creatinine kinase; Scr, serum creatinine; ACE, angiotensin converting enzyme; IQR, interquartile range. The number in the parentheses indicates the percentage. N/A, Data not available. Group comparisons used appropriate statistical tests, including the chi-square test, fisher exact test, unpaired two-tailed t-test, or Wilcoxon test.

**Supplementary Table 2: Baseline characteristics of survival and death patients.**

| | Survival(n=94) | Death(n=94) | P-value |
|---|---|---|---|
| Basic Information | | | |
| Age (years) | 68.28±9.850 | 68.31±9.754 | 0.982 |
| Male sex, n (%) | 56(59.57) | 56(59.57) | 1 |
| Clinical Profile | | | |
| BMI (kg/m$^2$) | 23.83±3.365 | 23.941±4.010 | 0.847 |
| LVEDD (mm), median (IQR) | 45.781(42.6,48) | 49.794 (44.2,55.9) | <0.001 |
| LVEF (%), median (IQR) | 55.996(51,62) | 47.768(40.5,56) | <0.001 |

| | | | |
|---|---|---|---|
| Biochemistry at admission | | | |
| LDL cholestrol (mmol/L) | 2.900±0.791 | 2.750±0.895 | 0.233 |
| NT-proBNP (pg/mL), median (IQR) | 1390.33(139,1940) | 7400.01 (678,8103) | <0.001 |
| TNI (μg/L), median (IQR) | 28.306 (0.665,77.347) | 27.411 (0.714,19.783) | 0.831 |
| CKMB (μg/L), median (IQR) | 59.298(3.55,85.5) | 69.667(3.2,44.7) | 0.541 |
| Scr(μmol/L), median (IQR) | 89.37(74,101) | 134.53(81.5,129) | <0.001 |
| eGFRmL (min*1.73m$^2$) | 72.933±20.697 | 59.895±24.756 | <0.001 |
| Glucose (mg/dL), median (IQR) | 8.056(5.46,9.64) | 8.543(5.84,9.46) | 0.338 |
| Medical history,n(%) | | | |
| Hypertension | 50(53.19) | 58(61.70) | 0.238 |
| Hyperlipoidemia | 17(18.09) | 21(22.34) | 0.468 |
| Diabetes | 23(24.47) | 53(56.38) | 0.111 |
| Chronic kidney disease | 5(5.32) | 9(9.57) | 0.266 |
| Stroke | 20(21.28) | 31(32.98) | 0.071 |
| Management, n (%) | | | |
| Thrombolysis | 3(3.19) | 2(2.13) | 0.650 |
| Multivessel disease | 78(82.98) | 80(85.11) | 0.690 |
| Infarct-related artery, n (%) | | | 0.709 |
| LAD | 53(56.38) | 51(54.26) | |

| | | | |
|---|---|---|---|
| LCX | 3(3.19) | 13(13.83) | |
| LM | 8(8.51) | 3(3.19) | |
| RCA | 30(31.91) | 27(28.72) | |

Data are expressed as mean ± SEM or median (interquartile ranges, 25th-75th percentile) or number of subjects (%). BMI, body mass index; LVEDD, left ventricular end-diastolic dimension; eGFR estimated glomerular filtration rate; LAD, left anterior descending; LCX, left circumflex; LM, Left main; RCA, right coronary artery. The number in the parentheses indicate the percentage. N/A, Data not available. Group comparisons used appropriate statistical tests, including the chi-square test, fisher exact test, unpaired two-tailed t-test, or Wilcoxon test.

**Supplementary Table 3: P value of spearman's correlation analysis of S100a9K26la and S100a9 with myocardial injury markers and cardiac function indexes**

| sample | S100a9 K26la | s100a9 | Glucose | SBP | DBP | HR | LVEDD | LVEF | NT-proBNP | cTnI | CK-MB | eGFR | Scr |
|---|---|---|---|---|---|---|---|---|---|---|---|---|---|
| S100a9K26la | 0.0000 | 0.0666 | 0.9873 | 0.1539 | 0.4621 | 0.2396 | 0.7128 | 0.0010 | 0.0083 | 0.0044 | 0.0126 | 0.6074 | 0.1729 |
| s100a9 | 0.0666 | 0.0000 | 0.8797 | 0.4179 | 0.5950 | 0.6618 | 0.2213 | 0.9841 | 0.0006 | 0.7725 | 0.1663 | 0.0065 | 0.0000 |
| Glucose | 0.9873 | 0.8797 | 0.0000 | 0.8789 | 0.9512 | 0.3375 | 0.5717 | 0.4196 | 0.8003 | 0.9526 | 0.2413 | 0.1355 | 0.7588 |
| SBP | 0.1539 | 0.4179 | 0.8789 | 0.0000 | 0.0000 | 0.0043 | 0.2650 | 0.4003 | 0.3078 | 0.2688 | 0.9807 | 0.1365 | 0.1290 |
| DBP | 0.4621 | 0.5950 | 0.9512 | 0.0000 | 0.0000 | 0.0000 | 0.0414 | 0.4304 | 0.0753 | 0.7485 | 0.7383 | 0.5407 | 0.0206 |
| HR | 0.2396 | 0.6618 | 0.3375 | 0.0043 | 0.0000 | 0.0000 | 0.1246 | 0.0017 | 0.0512 | 0.9807 | 0.8699 | 0.2112 | 0.0944 |
| LVEDD | 0.7128 | 0.2213 | 0.5717 | 0.2650 | 0.0414 | 0.1246 | 0.0000 | 0.0000 | 0.0004 | 0.8249 | 0.6092 | 0.1391 | 0.0285 |
| LVEF | 0.0010 | 0.9841 | 0.4196 | 0.4003 | 0.4304 | 0.0017 | 0.0000 | 0.0000 | 0.0001 | 0.3068 | 0.3942 | 0.0030 | 0.0588 |
| NT-proBNP | 0.0083 | 0.0006 | 0.8003 | 0.3078 | 0.0753 | 0.0512 | 0.0004 | 0.0001 | 0.0000 | 0.7831 | 0.9279 | 0.0000 | 0.0000 |
| cTnI | 0.0044 | 0.7725 | 0.9526 | 0.2688 | 0.7485 | 0.9807 | 0.8249 | 0.3068 | 0.7831 | 0.0000 | 0.0000 | 0.6868 | 0.9838 |
| CK-MB | 0.0126 | 0.1663 | 0.2413 | 0.9807 | 0.7383 | 0.8699 | 0.6092 | 0.3942 | 0.9279 | 0.0000 | 0.0000 | 0.3350 | 0.6175 |
| eGFR | 0.6074 | 0.0065 | 0.1355 | 0.1365 | 0.5407 | 0.2112 | 0.1391 | 0.0030 | 0.0000 | 0.6868 | 0.3350 | 0.0000 | 0.0000 |
| Scr | 0.1729 | 0.0000 | 0.7588 | 0.1290 | 0.0206 | 0.0944 | 0.0285 | 0.0588 | 0.0000 | 0.9838 | 0.6175 | 0.0000 | 0.0000 |

**Supplementary Table 4: Baseline characteristics of cardiac death cohort after excluding patients with infections.**

| | Survival(n=62) | Death(n=62) | P-value |
|---|---|---|---|
| Basic Information | | | |
| Age(years) | 67.39±10.661 | 67.40±10.489 | 0.993 |
| Male sex, n(%) | 35(56.45) | 35(56.45) | 1 |
| Clinical Profile | | | |
| BMI(kg/m$^2$) | 23.565±3.261 | 24.0771±3.962 | 0.459 |
| LVEDD(mm), median (IQR) | 45.215(42.6,47.475) | 48.482（42.9,55.9) | 0.007 |
| LVEF(%), median (IQR) | 57.579(52.1,62) | 48.627(42.875,56.25) | <0.001 |
| Biochemistry at admission | | | |
| TNI(μg/L), median (IQR) | 29.986(0.670,17.304) | 28.592(0.638,21.625) | 0.734 |
| NT-proBNP(pg/mL), median (IQR) | 875.61(135.75,1038.50) | 6479.65(447.50,7154.25) | <0.001 |
| CKMB(μg/L), median (IQR) | 48.658(3.575,60.80) | 68.779(2.80,45.575) | 0.879 |
| LDL cholesterol (mmol/L),median (IQR) | 2.977±0.860 | 2.705±0.835 | 0.08 |
| Glucose(mg/dL), median (IQR) | 7.905(5.545,9.748) | 8.413(5.495,9.390) | 0.455 |

| | | | |
|---|---|---|---|
| eGFRmL(min*1.73$m^2$),median (IQR) | 77.455±19.810 | 63.530±24.487 | 0.001 |
| Scr(μmol/L), median (IQR) | 82.66(67,94.25) | 123.69(79.5,112.75) | 0.002 |
| Medical history, n(%) | | | |
| Hypertension | 30(48.39) | 33(53.23) | 0.59 |
| Diabetes | 12(19.35) | 17(27.42) | 0.289 |
| Hyperlipoidemia | 12(19.35) | 12(19.35) | 1 |
| Stroke | 12(19.35) | 17(27.42) | 0.289 |
| Chronic kidney disease | 3(4.84) | 7(11.29) | 0.196 |
| Management, n(%) | | | |
| Thrombolysis | 1(1.61) | 1(1.61) | 1.000 |
| Multivessel disease | 51(82.26) | 52(83.87) | 0.811 |
| Infarct-related artery, n(%) | | | |
| LAD | 32(51.61) | 32(51.61) | |
| LCX | 5(8.06) | 7(11.29) | |
| LM | 1(1.61) | 1(1.61) | |
| RCA | 24(38.71) | 22(35.48) | |

Data are expressed as mean ± SEM or median (interquartile ranges, 25th-75th percentile) or number of subjects (%). BMI, body mass index; LVEDD, left ventricular end-diastolic dimension; eGFR estimated glomerular filtration rate; LAD, left anterior descending; LCX, left circumflex; LM, left main; RCA, right coronary artery. The number in the parentheses

indicates the percentage. N/A, Data not available. Group comparisons used appropriate statistical tests, including the chi-square test, fisher exact test, unpaired two-tailed t-test, or Wilcoxon test.

**Supplementary Table 5: Oligonucleotides (Primer Sequences)**

| Gene | Forward primer | Reverse primer |
|---|---|---|
| β-actin | GGACCTGACAGACTACCTCAT | GCTCGAAGTCTAGAGCAACATAG |
| Cxcr1 | GCTGCCCACTGGAGATTATT | GAAGGGACACCAGTGCATAAA |
| Itga2 | TTGATGCGGACGGACATTCA | GATGTCCACACGCAGATCCA |
| Rhoj | GGGAAGACCTGCTTGCTGAT | CACGGTAACTGCGTAGTGGT |
| Comp | CGTGGGCTGGAAGGATAAA | TACTAGCTCAGGACCCTCATAG |
| Postn | CAAGGCAGTCTTCAGCCTATTA | CACCGTTTCGCCTTCTTTAATC |
| Col1a1 | AGACCTGTGTGTTCCCTACT | GAATCCATCGGTCATGCTCTC |
| Col3a1 | GTGACTCAGGATCTGTCCTTTG | GTAGAAGGCTGTGGGCATATT |

**Supplementary Table 6: ChIP-qPCR Primer Sequences**

| Gene | Forward primer | Reverse primer |
|---|---|---|
| Cxcr1 | ACCTTGAGTTTGCTCCCAGAAT | CCAGAGGCTAGAGGACAATTTTAAG |
| Itga2 | TTAGGCTCAGGGATTAGGGTG | GAAAGGTAGGTGATCGTGGATT |
| Rhoj | TTTGTCCCAACCTCCCTGAC | AGCAGAAGGGAGACTGGATCT |

**Supplementary Table 7: siRNA Primer Sequences**

| Gene | Forward primer | Reverse primer |
|---|---|---|
| siRNA-Dlat | GCAUCAGAAGGUUCCAUUATT | UAAUGGAACCUUCUGAUGCTT |
| siRNA-NC | UUCUCCGAACGUGUCACGUTT | ACGUGACACGUUCGGAGAATT |

**Supplementary Figure 1**

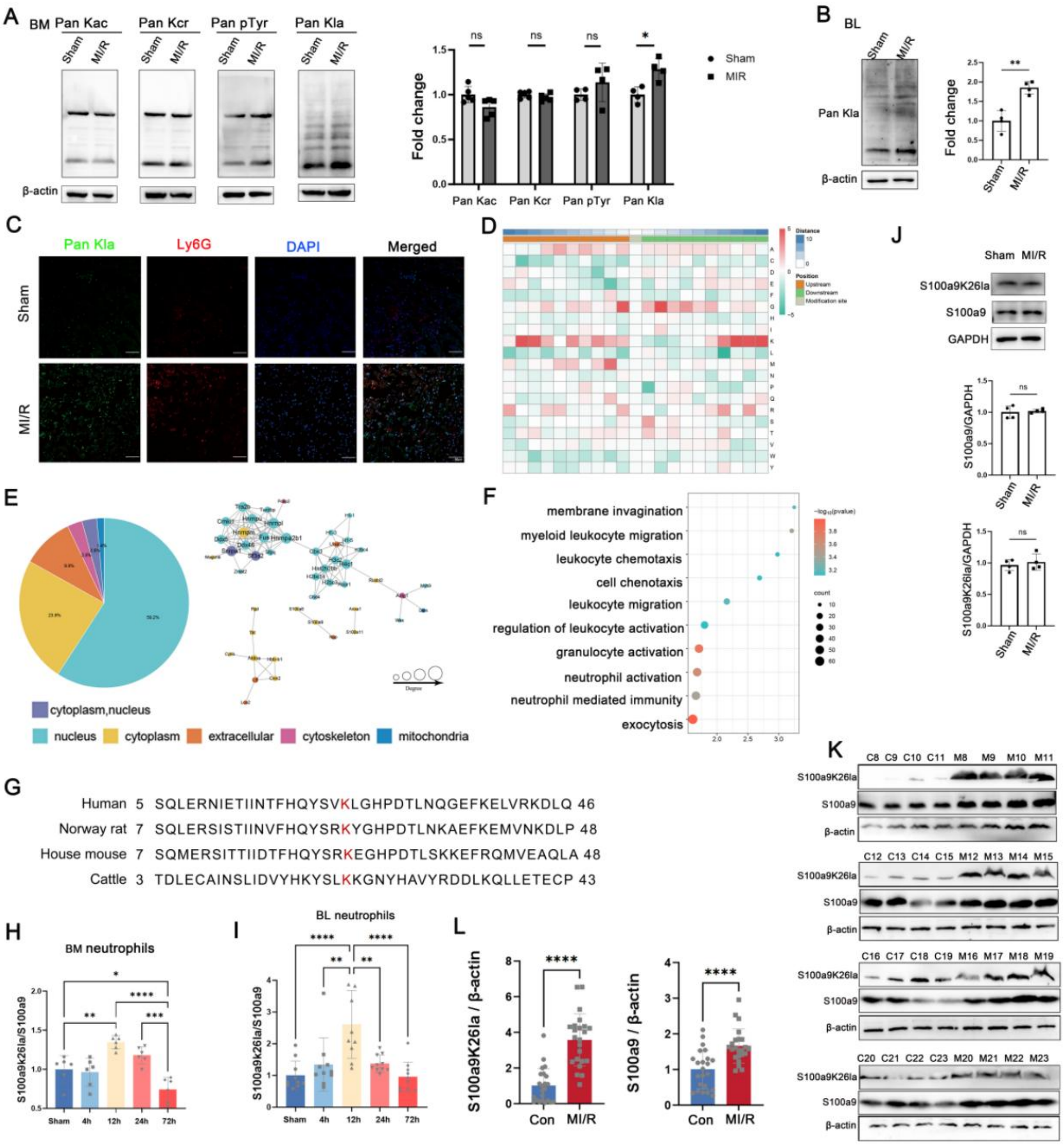


**Supplementary Figure 1. Global view of lactylated proteins and identification of S100a9 lactylation in neutrophils under MI/R.**

**A,** The immunoblots of crotonylation, phosphorylation, acetylation, and lactylation modification in the BM neutrophils post-MI/R (n=4). **B**, The Pan Kla immunoblots of peripheral blood (BL) neutrophils from MI/R mice (n=4). Mean ± SD. Statistical tests for (**A–B**): unpaired 2-tailed Student t t-test ($^{*}P$ < 0.05, and $^{****}P$ < 0.0001 for indicated comparisons. ns, not significant). **C**, Immunofluorescence co-staining for Ly6G (red) with Pan Kla (green) in infarcted hearts 1 day post-MI/R (scale bar=40 μm, n=6). **D**, Motif analysis of all identified Kla proteins. **E**, Subcellular location and protein-protein interaction network diagram of proteins exhibiting remarkable upregulated Kla changes. **F,** Bubble plot of the top 10 Gene Ontology terms of remarkably upregulated proteins. **G,** The peptide containing lactylated lysine of S100a9 is conserved in human, mouse, rat, cattle. **H-I**, Quantification of relative changes in S100a9K26la and S100a9 levels in BM (**H**) and BL (**I**) neutrophils at different time after MI/R (n=6). **J,** Immunoblots analysis of isolated adult cardiomyocytes from sham-operated mice or day1 post-MI/R suggested that the expression of S100a9 and S100a9K26la in the cardiac cells remained unchanged (n=4). Mean ± SD. Statistical tests for (**J**): unpaired 2-tailed Student t t-test. ns, not significant. **K-L,** Blood samples were collected from patients with AMI undergoing percutaneous coronary intervention (MI/R, n=23) or from patients with UA (Con, n=23) 24 h post PCI. Immunoblotting and quantification of S100a9K26la and S100a9 in neutrophils from the control and MI/R groups. Median (interquartile ranges, 25th-75th percentile). Statistical tests for (**L**): 2-tailed Mann–Whitney test (****$P$ < 0.0001 for indicated comparisons).

**Supplementary Figure 2**

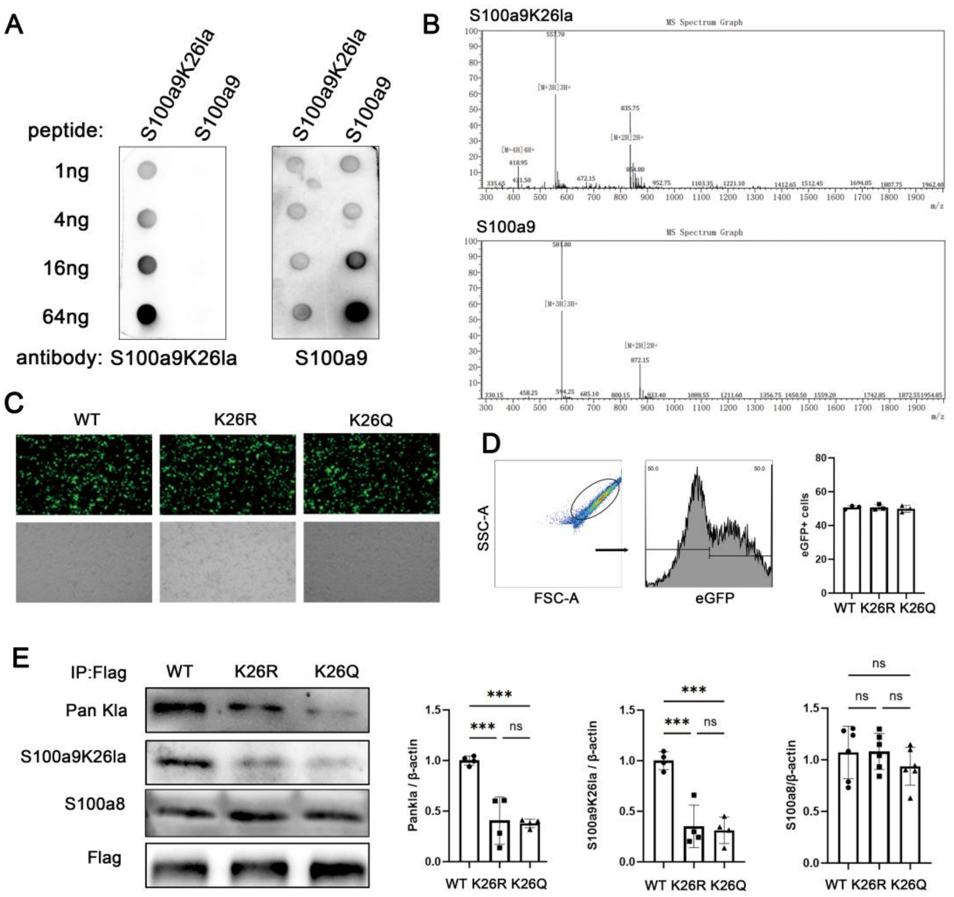


**Supplementary Figure 2: Experimental verification of customized S100a9K26la antibody**

**A**, Antibody specificity was analysed by dot blot assay. **B**, MS/MS spectra of S100a9K26la and S100a9 peptides derived from the synthetic counterparts. **C**, S100a9K26WT-Flag (WT), S100a9 K26R-Flag (K26R) and S100a9 K26R-Flag (K26Q) adenovirus were transfected into 32Dcl3-induced neutrophils for 24h under fluorescence microscopy. **D**, The proportions of transfected neutrophils ($eGFP^{+}$) in WT, K26R and K26Q groups, as determined by FACS (n=3). **E**, Mutants of K26 decrease S100a9 lactylation. Lactylation of S100a9 WT, K26R, or K26Q was detected by pan-Kla, S100a9K26la, and S100a8-Antibody (n=4). Mean ± SD. Statistical tests for (**E**): 1-way ANOVA with Tukey's

multiple comparison test ($P$ values adjusted for 6 comparisons; $^{***}P < 0.001$ for indicated comparisons, ns, not significant).

**Supplementary Figure 3**

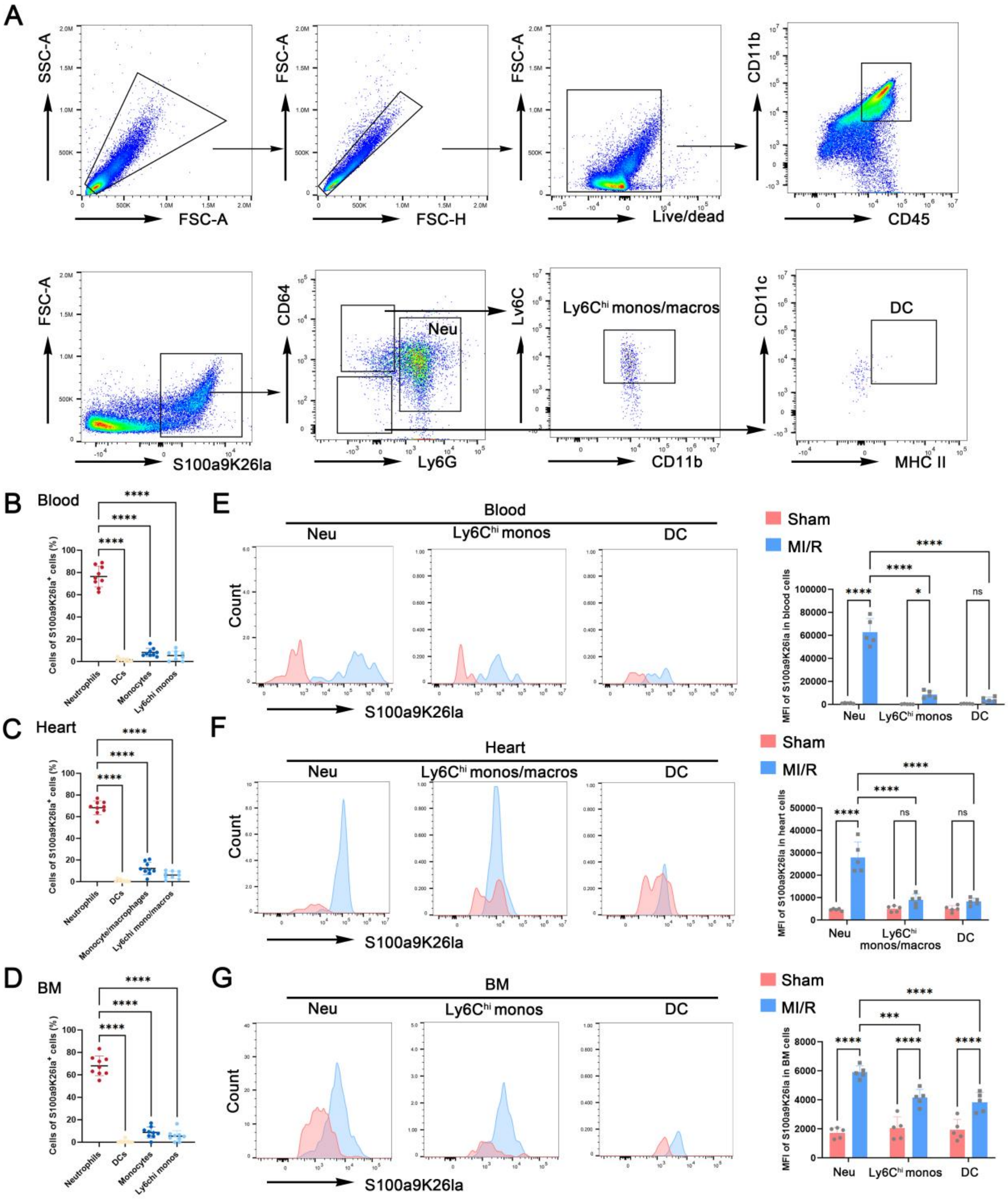


**Supplementary Figure 3: S100a9K26la is mainly derived from neutrophils post MI/R.**

**A-D,** Gating strategy (A) and statistical analysis of the main source of S100a9K26la in blood (B), heart (C) and BM (D) 1 day post-MI/R (n = 8). Mean ± SD. Statistical tests for (**B-D**): 1-way ANOVA with Tukey's multiple comparison test (*P* values adjusted for 6 comparisons; ****$P < 0.0001$ for indicated comparisons). **D-F,** MFI of S100a9K26la protein expression in neutrophils (Neu), Ly6C$^{hi}$ monocytes/macrophages (Ly6C$^{hi}$ monos/macros) or dendritic cells (DC) from the blood (**E**) heart (F) or BM (G) on day 1 post-MI/R (n=5). MFI, mean fluorescence intensity. Mean ± SD. Statistical tests for (**E-G**): 2-way ANOVA with Tukey's multiple comparison test (*P* values adjusted for 6 comparisons; ***$P < 0.001$, and ****$P < 0.0001$ for indicated comparisons. ns, not significant).

**Supplementary Figure 4**

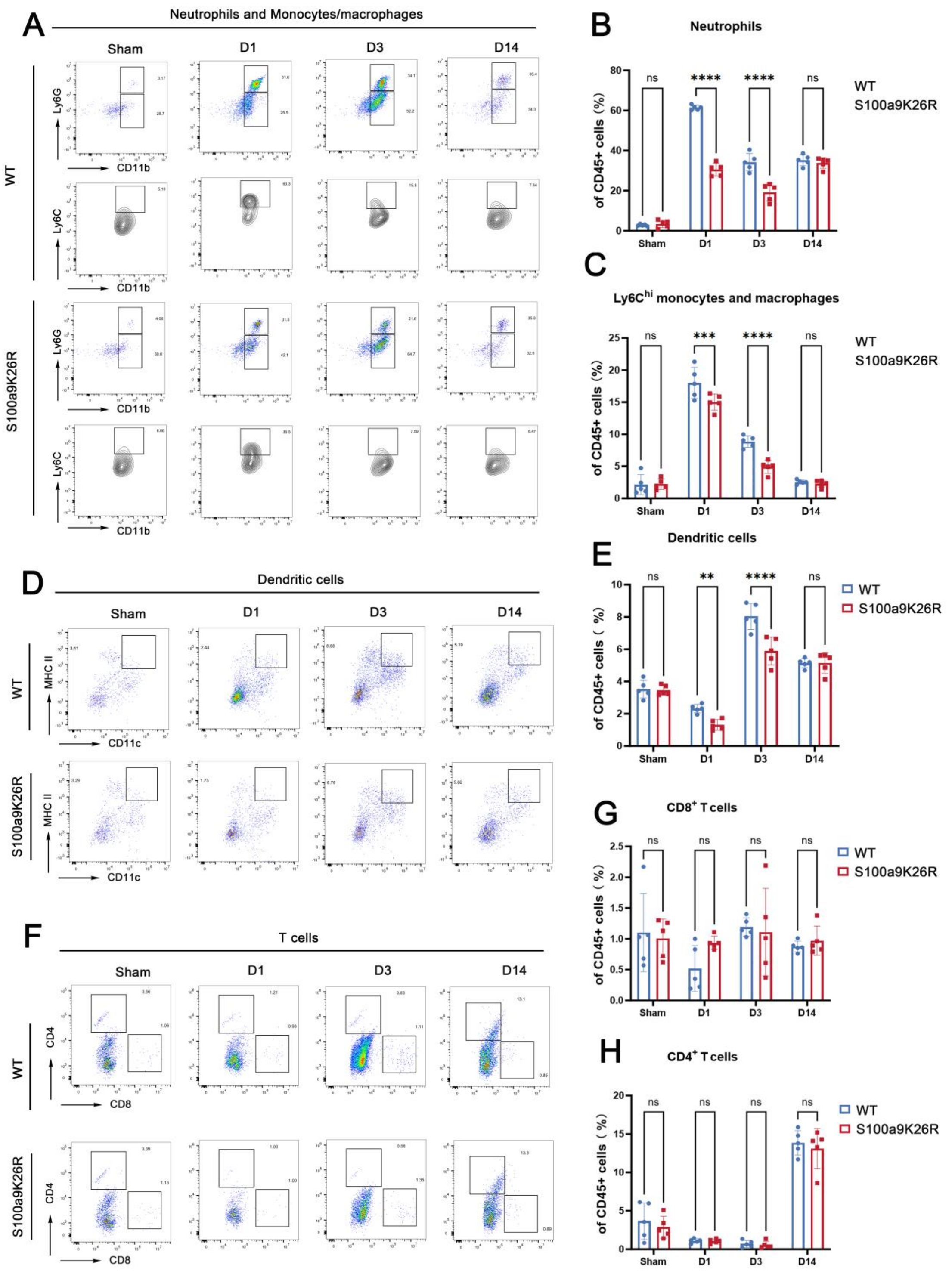


**Supplementary Figure 4: S100a9K26R affects acute immune cell accumulation in the heart post-MI/R.**

**A-C,** FACS quantification of CD45+CD11b+Ly6G+ neutrophils and CD45+CD11b+Ly6G-Ly6C$^{hi}$ monocytes/macrophages in percentage of total CD45+ cells at day 1, 3 and 14 post-MI/R (n=5). **D-E,** FACS quantification of CD45+CD11c+MHC-II+ dendritic cells in percentage of total CD45+ cells at day 1, 3 and 14 post-MI/R (n=5). **F-H,** FACS quantification of CD45+CD4+CD8– and CD4–CD8+ T cells in percentage of total CD45+ cells at day 1, 3 and 14 post-MI/R (n=5). Mean ± SD. Statistical tests for (**B, C, E, G, and H**): 2-way ANOVA with Tukey's multiple comparison test (*P* values adjusted for 6 comparisons; $^{**}P < 0.01$, $^{***}P < 0.001$, and $^{****}P < 0.0001$ for indicated comparisons. ns, not significant).

**Supplementary Figure 5**

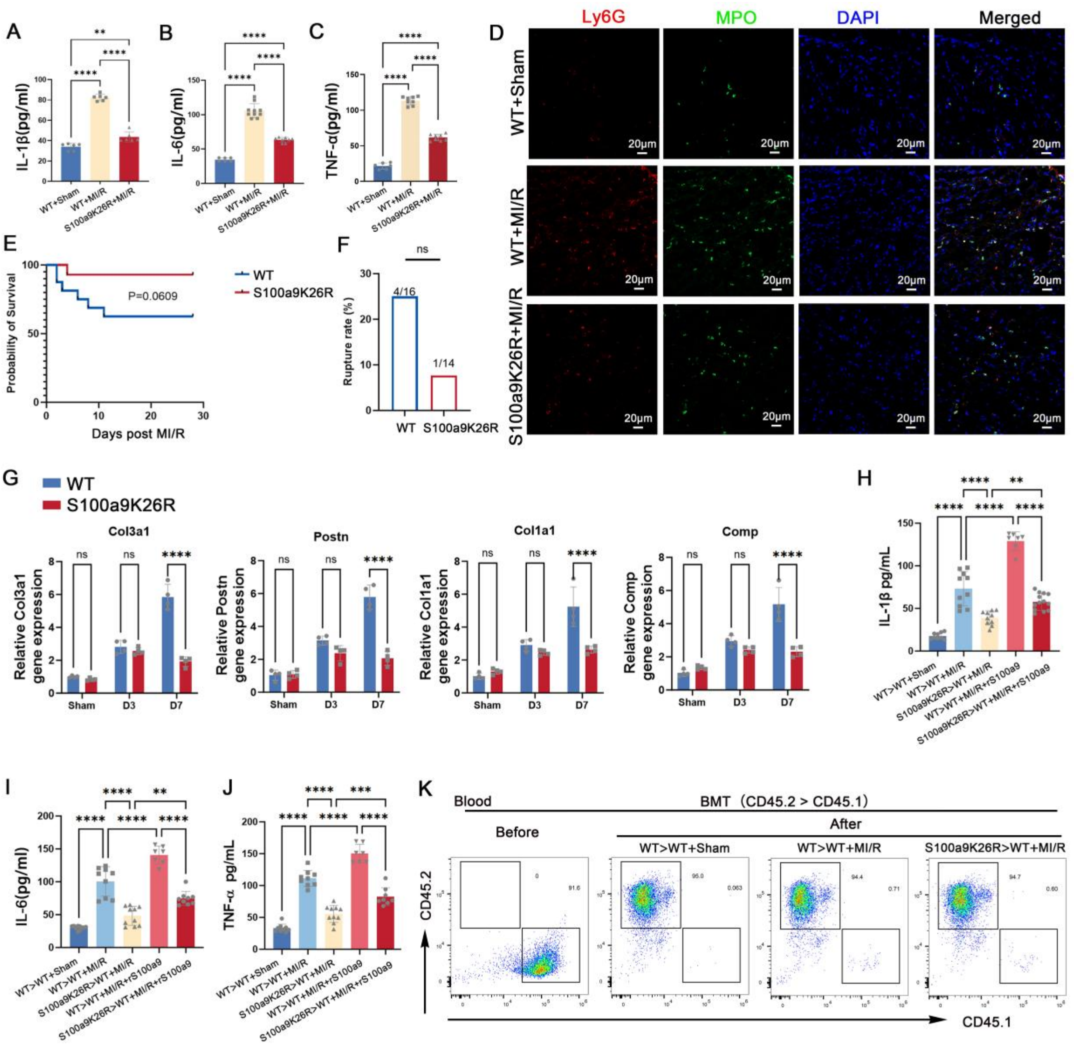


**Supplementary Figure 5: S100a9K26R reduces the systemic inflammation post MI/R**

**A-C**, Levels of IL-1β(A), IL-6(B) and TNF-α(C) in the plasma were measured by ELISA in the WT and S100a9K26R mice 1day post MI/R (n=6-10). Mean ± SD. Statistical tests for (**A-C**): 1-way ANOVA with Tukey's multiple comparison test (*P* values adjusted for 6 comparisons; $^{**}P < 0.01$, and $^{****}P < 0.0001$ for indicated comparisons). **D,** Immunofluorescence co-staining for Ly6G (red) with MPO (green) in infarcted hearts 1 day post-MI/R (scale bar=20 μm, n=4). **E**, Survival analysis of wild-type (WT) and S100a9K26R mice post-MI/R or sham operation (n=14-16 mice per group, log-rank test). **F,** Cardiac rupture rate during the 4 weeks of follow-up; P=0.1755 (two-sided Fisher's test). **G,** Relative fold

change of collagen genes and fibrosis-related genes in sham and post-MI/R day3 and 7 WT and S100a9K26R hearts (n=4). Mean ± SD. Statistical tests for (**G**): 2-way ANOVA with Tukey's multiple comparison test (*P* values adjusted for 6 comparisons; $^{****}P < 0.0001$ for indicated comparisons. ns, not significant). **H-K,** WT recipient mice (CD45.1) were transplanted with BM from WT or S100a9K26R donor mice (CD45.2), and subjected to MI/R. Recipient mice receiving BM from S100a9K26R mice were infused intravenously with rS100a9 after MI/R. Levels of IL-1β(H), IL-6(I) and TNF-α(J) in the plasma were measured by ELISA in recipient mice (n=8 to 13 per group). Mean ± SD. Statistical tests for (**H-J**): 1-way ANOVA with Tukey's multiple comparison test (*P* values adjusted for 6 comparisons; $^{**}P < 0.01$, $^{***}P < 0.001$, and $^{****}P < 0.0001$ for indicated comparisons). **K,** Flow cytometry analysis of recipient CD45.1 before and after bone marrow transplantation (BMT).

**Supplementary Figure 6**

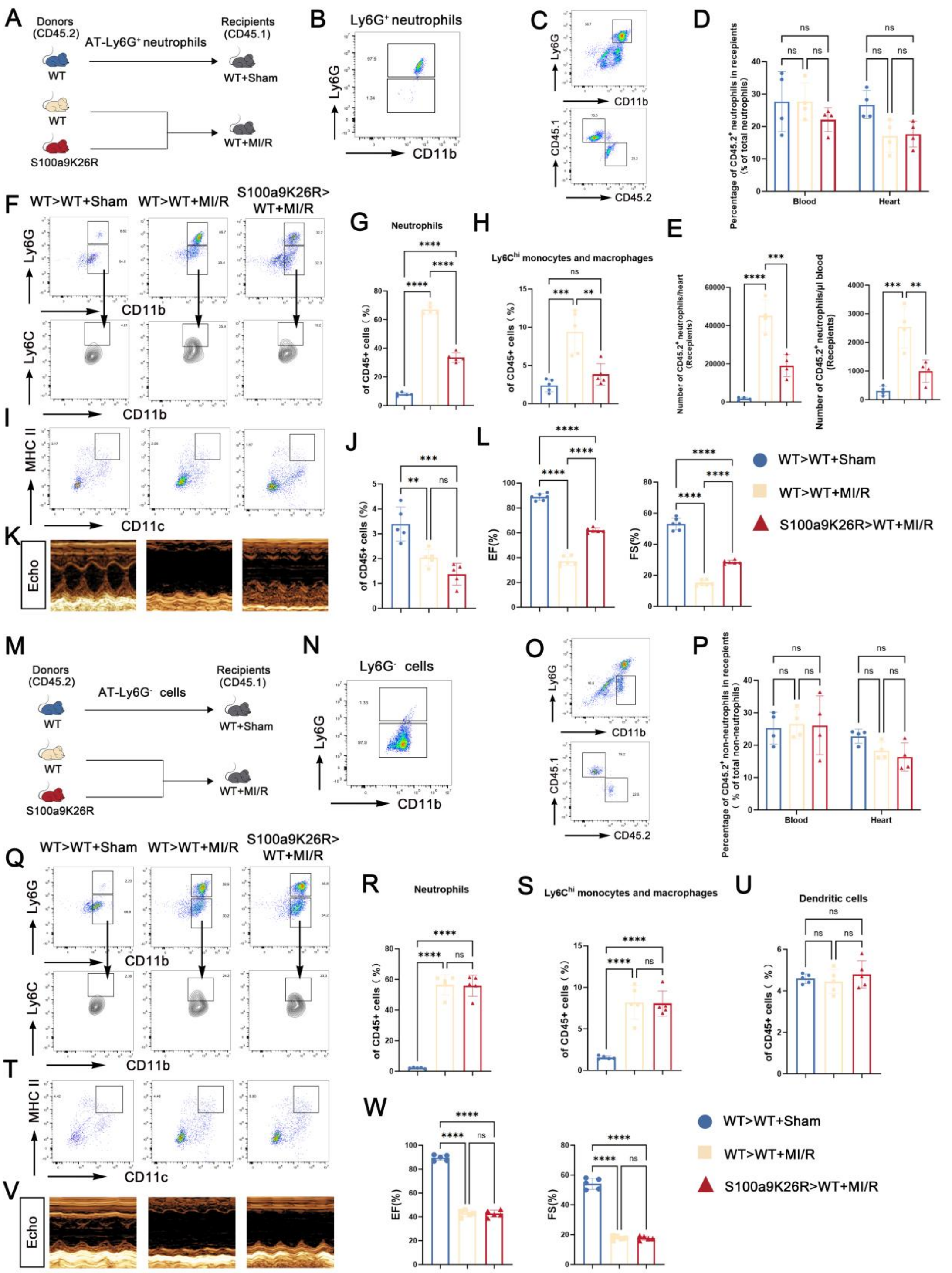


**Supplementary Figure 6: Neutrophil transfer from S100a9K26R to WT mice worsens MI/R**

**A,** Experimental overview of adoptive transfer (AT): MI was generated in WT mice (recipients, CD45.1) , followed by BM neutrophils (donors CD45.2) isolated from WT and

S100a9K26R mice transferred one hour after surgery. **B,** Representative flow cytometric images of neutrophils for AT. **C,** Representative flow cytometric images of CD45.2 neutrophils remaining in CD45.1 WT recipient mice in the blood and heart tissues at 24 hours post-AT. **D-E,** The percentage (**D**) of CD45.2 neutrophils to total neutrophils and number (**E**) of CD45.2 neutrophils in the blood and heart tissues at 24 hours post-AT (n=4). **F-H,** FACS quantification of CD45+CD11b+Ly6G+ neutrophils (F, G) and CD45+CD11b+Ly6G-Ly6C$^{hi}$ monocytes/macrophages (F, H) in percentage of total CD45 + cells in heart at day 1 post-AT (n=5). **I-J,** FACS quantification of CD45+CD11c+MHC-II+ dendritic cells in percentage of total CD45 + cells in heart at day 1 post-AT (n=5). **K-L,** Representative M-mode echocardiograms with measurements on day 7 after MI/R (n=5). Mean ± SD. Statistical tests for (**D, E, G, H, J, L**): 1-way ANOVA with Tukey's multiple comparison test (*P* values adjusted for 6 comparisons; $^{**}P < 0.01$, $^{***}P < 0.001$, and $^{****}P < 0.0001$ for indicated comparisons. ns, not significant). **M,** Experimental overview of adoptive transfer (AT): MI was generated in WT mice (recipients, CD45.1) , followed by BM non-neutrophils (donors CD45.2) isolated from WT and S100a9K26R mice transferred one hour after surgery. **N,** Representative flow cytometric images of non-neutrophils for AT. **O-P,** Representative flow cytometric images and the percentage of CD45.2 non-neutrophils remaining in CD45.1 WT recipient mice in the blood and heart tissues at 24 hours post-AT (n=4). **Q-S,** FACS quantification of CD45+CD11b+Ly6G+ neutrophils (Q, R) and CD45+CD11b+Ly6G-Ly6C$^{hi}$ monocytes/macrophages (Q, S) in percentage of total CD45 + cells in heart at day 1 post-AT (n=5). **T-U,** FACS quantification of CD45+CD11c+MHC-II+ dendritic cells in percentage of total CD45+ cells in heart at day 1 post-AT (n=5). **V-W,** Representative M-mode

echocardiograms with measurements on day 7 after MI/R (n=5). Mean ± SD. Statistical tests for (**P, R, S, U, W**): 1-way ANOVA with Tukey's multiple comparison test (*P* values adjusted for 6 comparisons; $^{****}P < 0.0001$ for indicated comparisons. ns, not significant).

**Supplementary Figure 7**

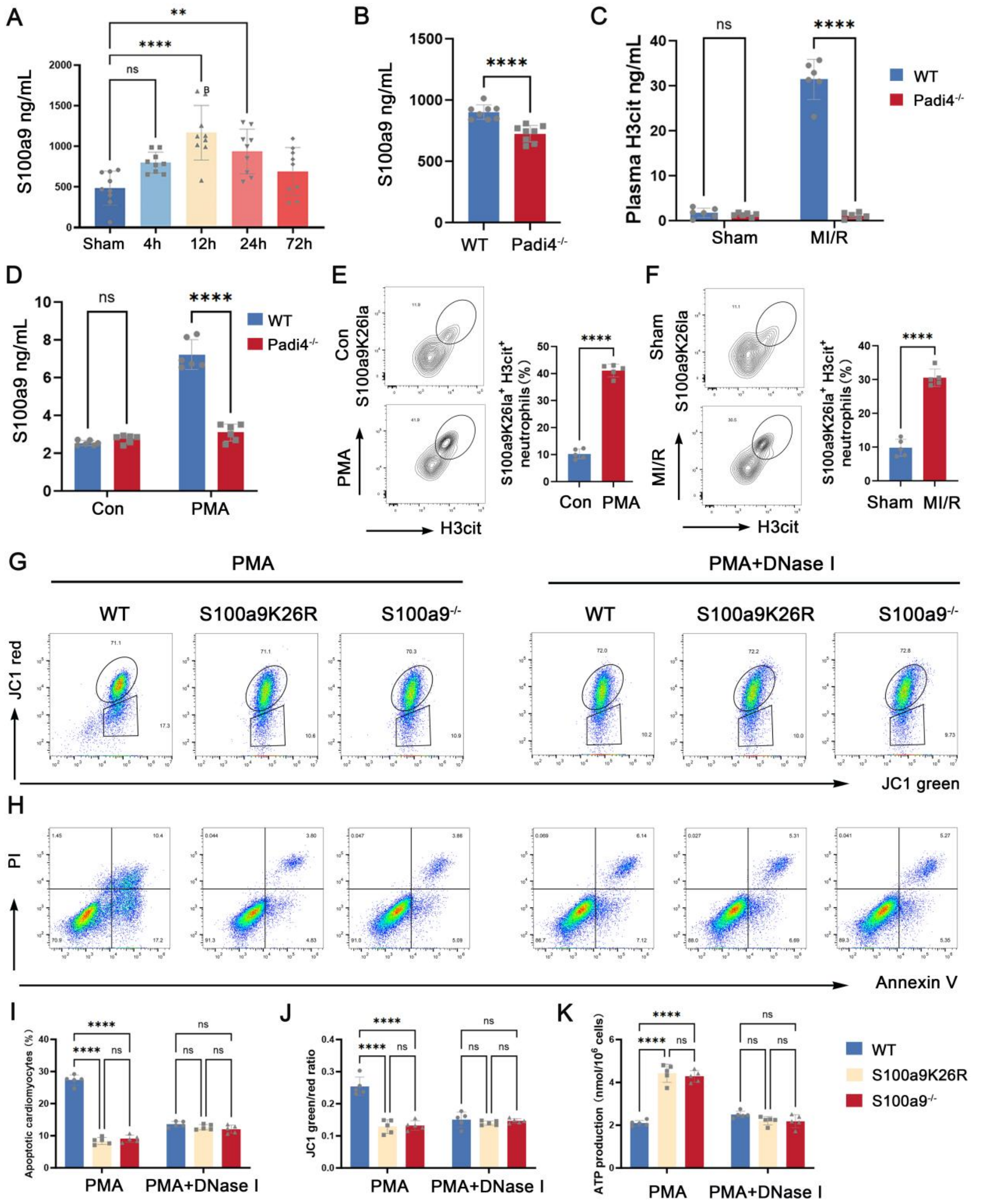


**Supplementary Figure 7: Lactylated S100a9 is released via NETs and triggers cardiomyocyte death by impairing mitochondrial function**

**A,** ELISA quantification of plasma S100a9 levels at various time points post-MI/R (A, n=9–13). Mean ± SD. Statistical tests for (**A**): 1-way ANOVA with Tukey's multiple comparison test (*P* values adjusted for 6 comparisons; $^{**}P < 0.01$ and $^{****}P < 0.0001$ for indicated comparisons). **B,** Detection of Plasma S100a9 levels from WT and Padi4$^{-/-}$ mice 1

day post MI/R by Elisa (n=8). Mean ± SD. Statistical tests for (**B**): unpaired 2-tailed Student t t-test ($^{****}P < 0.0001$ for indicated comparisons). **C**, Detection of Plasma H3cit levels from WT and *Padi4*$^{-/-}$ mice 1 day post MI/R by Elisa (n=6). **D,** BM neutrophils from WT and *Padi4*$^{-/-}$ mice were cultured for 4h with PMA stimulated, cell supernatants were collected for detection of S100a9 levels by Elisa (n=6). Mean ± SD. Statistical tests for (**C-D**): 2-way ANOVA with Tukey's multiple comparison test (*P* values adjusted for 6 comparisons; ****P < 0.0001 for indicated comparisons). ns, not significant. **E,** Representative FACS plots and proportion of the H3cit$^{+}$S100a9K26la$^{+}$ neutrophils in BM neutrophils treated with PMA (n=5). **F,** Representative FACS plots and proportion of the H3cit$^{+}$S100a9K26la$^{+}$ neutrophils in BM neutrophils isolated from sham and MI/R mice (n=5). Mean ± SD. Statistical tests for (**E-F**): unpaired 2-tailed Student t t-test ($^{****}P < 0.0001$ for indicated comparisons). **G-K,** NCMs were cultured with supernatants from WT and S100a9K26R neutrophils pretreated with PMA (phorbol myristate acetate; 200 nM/L) for 24 hours. DNase I (0.25 mg/ml) was employed to interrupt the formation of NETs, then collected NCMs to detect ATP levels (**K, n=5**), mitochondrial membrane depolarization (**G** and **J, n=6**) and Annexin V-APC and PI by flow cytometry (**H** and **I, n=6**). Mean ± SD. Statistical tests for (**I–K**): 2-way ANOVA with Tukey's multiple comparison test (*P* values adjusted for 6 comparisons; ****P < 0.0001 for indicated comparisons; ns, not significant).

**Supplementary Figure 8**

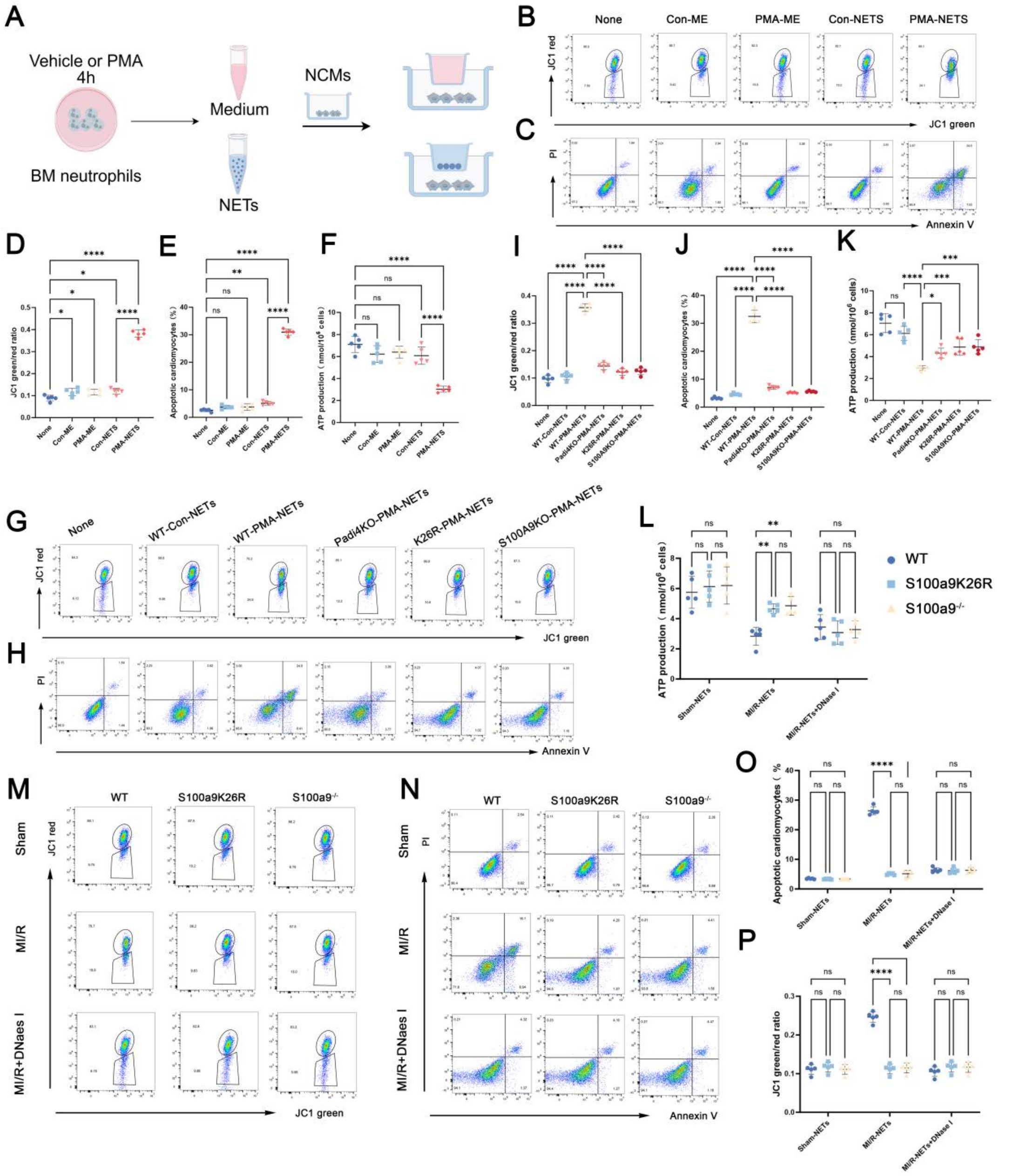


**Supplementary Figure 8: Lactylated S100a9 is released via NETs and triggers cardiomyocyte death by impairing mitochondrial function**

**A-F,** BM neutrophils were isolated from WT mice and treated with PMA for 4h, then conditioned medium and NETs were co-cultured with NCM cells respectively for 24 h, then collected NCMs to detect ATP levels (**F,** n=5), mitochondrial membrane depolarization (**B and D**, n=5) and Annexin V-APC and PI by flow cytometry (**C and E**, n=5). **G-K,** BM

neutrophils were isolated from WT, Padi4 knockout, S100a9K26R and S100a9 knockout mice and treated with PMA for 4h, then NETs were co-cultured with NCM cells respectively for 24 h, then collected NCMs to detect ATP levels (**K,** n=5), mitochondrial membrane depolarization (**G and I**, n=5) and Annexin V-APC and PI by flow cytometry (**H and J**, n=5). Mean ± SD. Statistical tests for (**D–F, I-K**): 1-way ANOVA with Tukey's multiple comparison test (*P* values adjusted for 6 comparisons; **P* < 0.05, ***P* < 0.01, ****P* < 0.001, *****P* < 0.0001 for indicated comparisons. ns, not significant). **L-P,** BM neutrophils were isolated from WT, S100a9K26R and *S100a9*$^{-/-}$ mice on sham-operated or day 1 MI/R, then NETs were collected to co-culture with NCMs in the presence or absence of DNase I for 24 h, then collected NCMs to detect ATP levels (**L,** n=5), mitochondrial membrane depolarization (**M and P**, n=5) and Annexin V-APC and PI by flow cytometry (**N and O**, n=5). Mean ± SD. Statistical tests for (**L, O, P**): 2-way ANOVA with Tukey's multiple comparison test (*P* values adjusted for 6 comparisons; ***P* < 0.01, *****P* < 0.0001 for indicated comparisons. ns, not significant).

**Supplementary Figure 9**

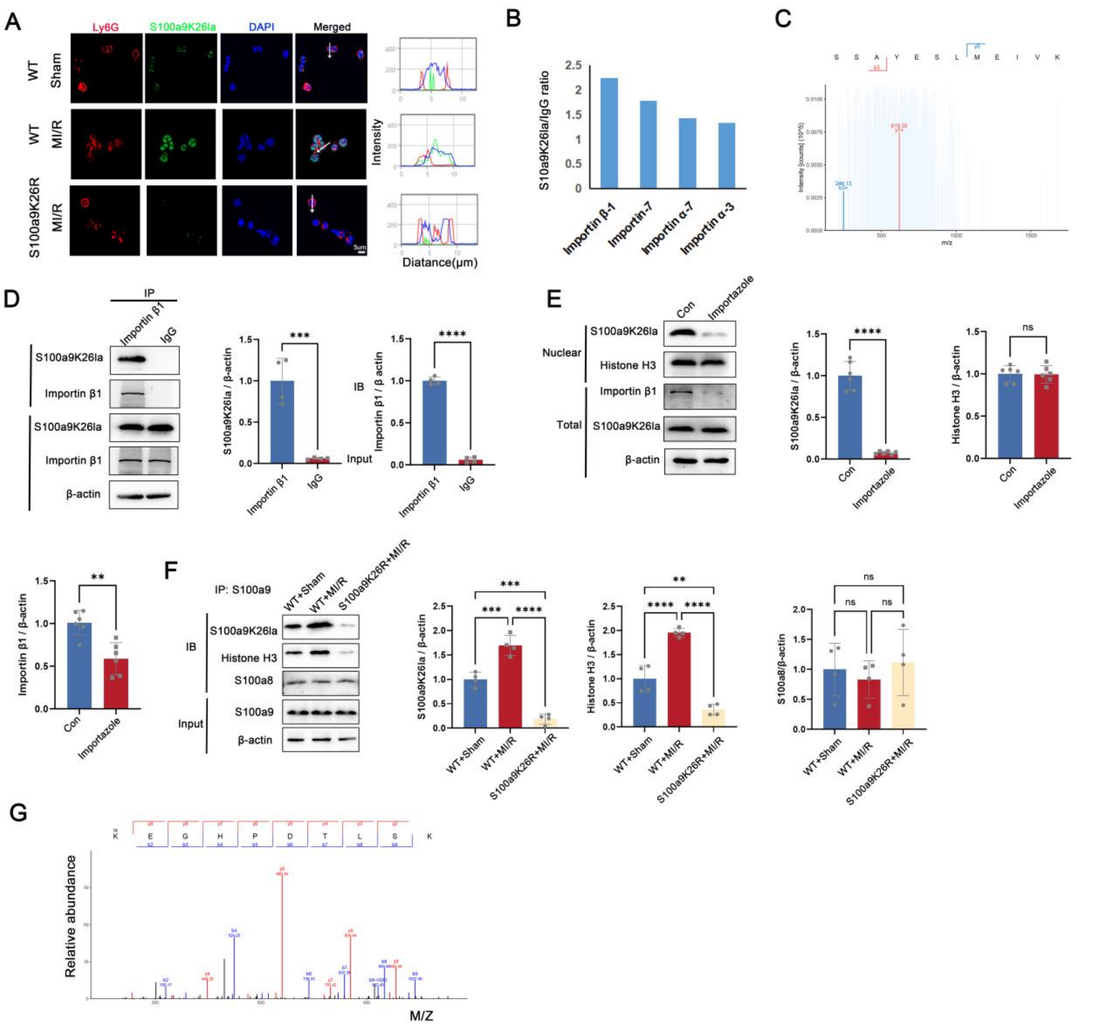


**Supplementary Figure 9: S100a9K26la accumulates in the nucleus of neutrophils via importin β1**

**A**, Immunofluorescence co-staining for S100a9K26la(green) with Ly6G(red) in BM neutrophils from WT and S100a9K26R mice day 1 post-MI/R (scale bar=1μm, n=5). **B**, Quantitation of importin proteins in neutrophils by IP-MS analysis using anti-S100a9K26la and IgG. **C**, Importin β1 were identified from nuclear proteins by MS. **D,** Interaction of S100a9K26la and Importinβ1 was detected by immunoprecipitation using anti-Importinβ1 and analyzed by Western blot with anti-S100a9K26la (n=4). **E**, BM neutrophils were cultured

with importinβ1 inhibitor importazole (20μm) for 4h with LPS stimulation, immunoblotting for S100a9K26la and Importinβ1 in cellular fractions of BM neutrophils (n=6). Mean ± SD. Statistical tests for (**D and E**): unpaired 2-tailed Student t t-test ($^{**}P < 0.01$, $^{***}P < 0.001$, and $^{****}P < 0.0001$ for indicated comparisons. ns, not significant). **F**, Interaction of S100a9K26la and Histone H3 was detected by immunoprecipitation using anti-S100a9 and analyzed by western blot with anti-S100a9K26la and anti-Histone H3 (n=4). Mean ± SD. Statistical tests for (**F**): 1-way ANOVA with Tukey's multiple comparison test ($P$ values adjusted for 6 comparisons; $^{**}P < 0.01$, $^{***}P < 0.001$, and $^{****}P < 0.0001$ for indicated comparisons. ns, not significant). **G,** S100a9K26la were identified from nuclear proteins by MS.

**Supplementary Figure 10**

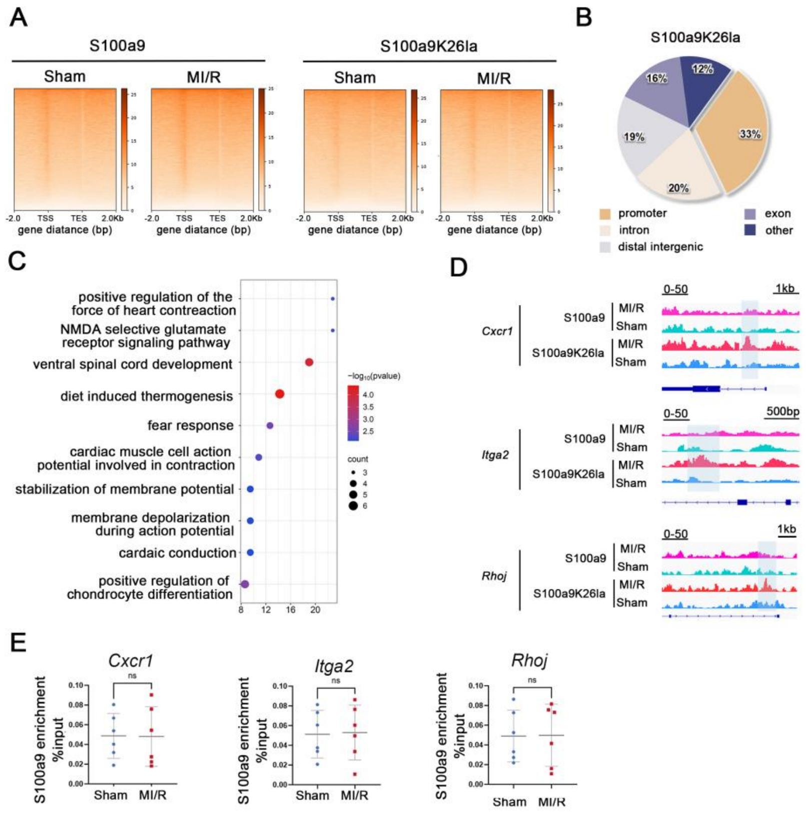

**Supplementary Figure 10: S100a9K26la binds to the TSS region of target genes in neutrophils**

**A**, Heatmaps for S100a9 and S100a9K26la binding peaks in neutrophils from sham-operated and MI/R mice. Color depth indicates the relative number of reads, genes with similar distribution patterns are clustered together through a clustering algorithm to show the binding trends of S100a9 and S100a9K26la on all genes. **B**, Pie charts displaying S100a9K26la binding site distribution, with the promoter defined as regions ±2 kb around the transcription start site (TSS). **C,** Bubble chart showing the top 10 Gene Ontology (GO) terms for genes with S100a9-specific modifications. **D**, IGV tracks for *Cxcr1*, *Itga2* and *Rhoj* from CUT&Tag analysis. **E**, S100a9 occupancy analysis by ChIP-qPCR (n=6). Mean ± SD. Statistical tests for (**E**): unpaired 2-tailed Student t t-test (ns, not significant).

**Supplementary Figure 11**

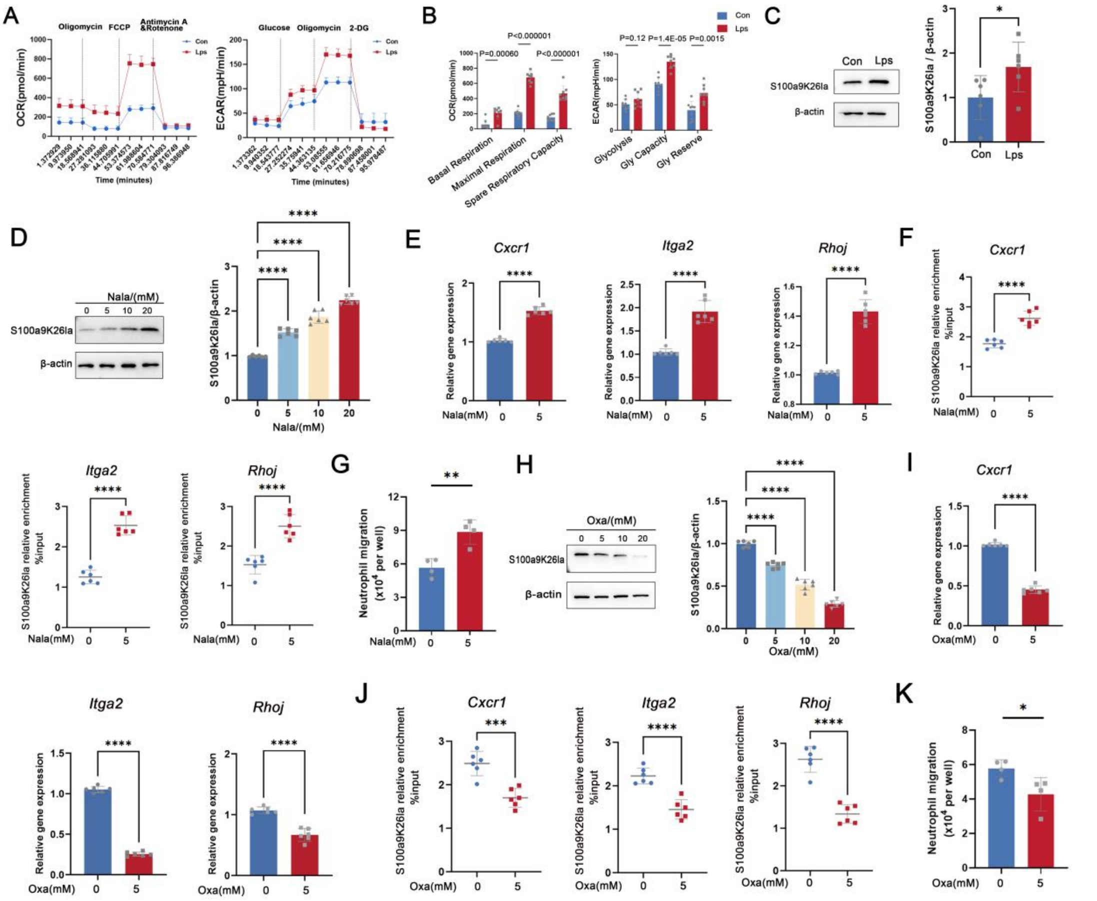


**Supplementary Figure11: S100a9K26 lactylation induces migratory genes of neutrophils in vitro**

**A-C,** BM neutrophils were treated with LPS for 4h, then collected for Mitochondrial stress test and glycolysis-stress test (A, n=8); Quantification of OCR values and extracellular acidification rate (ECAR) values (B, n=8); S100a9K26la immunoblot analysis (C, n=6). Mean ± SD. Statistical tests for (**B–C**): unpaired 2-tailed Student t t-test ($^{*}P$ < 0.05 for indicated comparisons). **D-G**, BM neutrophils were cultured with different concentrations of sodium lactate (NaLa) for 4h with LPS stimulation, followed by immunoblots and quantification of S100a9K26la (D, n=6), S100a9K26la occupancy analysis by ChIP-qPCR (E,

n=6), gene expression analysis by RT-qPCR (F, n=6), and neutrophil migration analysis by transwell (G, n=4). Mean ± SD. Statistical tests for (**D**): 1-way ANOVA with Tukey's multiple comparison test (*P* values adjusted for 6 comparisons; $^{****}P < 0.0001$ for indicated comparisons); Statistical tests for (**E-G**): unpaired 2-tailed Student t t-test ($^{**}P < 0.01$ and $^{****}P < 0.0001$ for indicated comparisons). **H-K**, BM neutrophils were cultured with different concentrations of lactate dehydrogenase inhibitor sodium oxamate (Oxa) for 4h with LPS stimulation, followed by immunoblots of S100a9K26la (H, n=6). S100a9K26la occupancy analysis by ChIP-qPCR (I, n=6), gene expression analysis by RT-qPCR (J, n=6), and neutrophil migration analysis by transwell (K, n=4). Mean ± SD. Statistical tests for (**H**): 1-way ANOVA with Tukey's multiple comparison test (*P* values adjusted for 6 comparisons; $^{****}P < 0.0001$ for indicated comparisons); Statistical tests for (**I-K**): unpaired 2-tailed Student t t-test ($^{*}P < 0.05$, $^{***}P < 0.001$, and $^{****}P < 0.0001$ for indicated comparisons).

1 **Supplementary Figure 12**

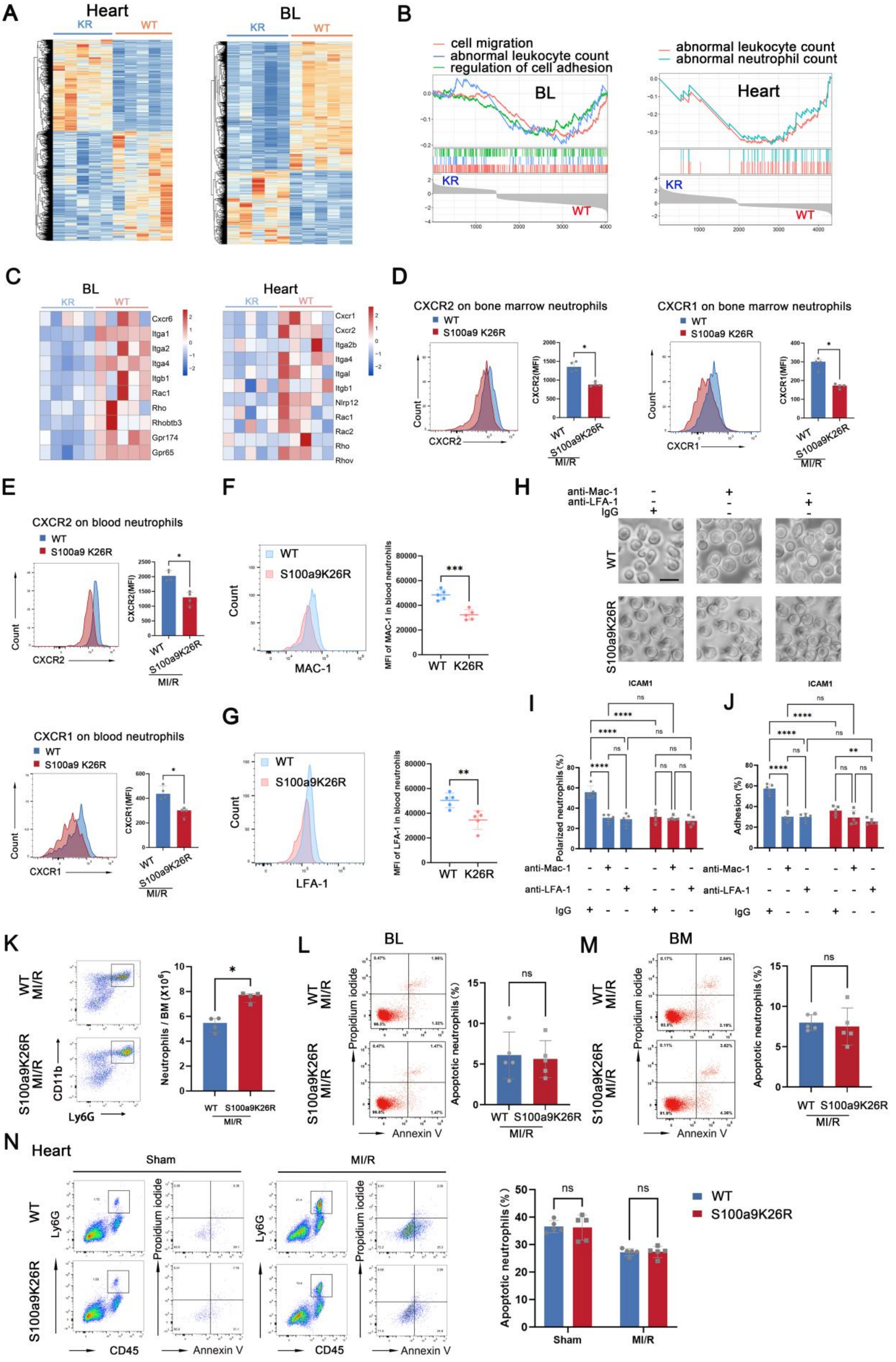


2

**Supplementary Figure 12: Inhibition of S100a9K26la in neutrophils impairs migratory transcription of neutrophils post-MI/R.**

**A,** Neutrophils isolated from the BL and heart of WT and S100a9K26R mice on day 1 post-MI/R were subjected to RNA-seq. **B,** Gene Set Enrichment Analysis of migration-related pathways. **C,** Heat map of migration genes in BL and heart neutrophils in S100a9K26R and WT mice on day 1 post-MI/R. **D-E**, FACS analysis of CXCR2 and CXCR1 protein expression on the surface of neutrophils from the blood (**E**) or bone marrow (BM) (**D**) of WT and S100a9K26R mice on day 1 post-MI/R (n=4). MFI, mean fluorescence intensity. Median (interquartile ranges, 25th-75th percentile). Statistical tests for (**D–E**): 2-tailed Mann–Whitney test ($^*P < 0.05$ for indicated comparisons). **F-G**, FACS analysis of MAC-1 (**F**) and LFA-1 (**G**) protein expression of neutrophils from the blood of WT and S100a9K26R mice on day 1 post-MI/R (n=4). MFI, mean fluorescence intensity. Mean ± SD. Statistical tests for (**F–I**): unpaired 2-tailed Student t t-test ($^*P < 0.05$ for indicated comparisons. ns, not significant). **H-J,** The adhesion (H-I) and polarization (J) assay of WT and S100a9K26R neutrophils isolated from BM at day 1 post-MI/R was determined on solid surface precoated with ICAM-1 in the presence of an anti-Mac-1–blocking antibody (M1/70), an anti-LFA-1 (lymphocyte function-associated antigen-1)–blocking antibody (M17/4), or IgG control (n=5). Mean ± SD. Statistical tests for (**I and J**): 2-way ANOVA with Tukey's multiple comparison test ($P$ values adjusted for 6 comparisons; $^{**}P < 0.01$, and $^{****}P < 0.0001$ for indicated comparisons. ns, not significant). **K,** Representative flow cytometry plots and quantification of BM neutrophils ($CD45^+CD11b^+Ly6G^+$) from WT and S100a9K26R mice on day 1 post-MI/R (n=4). **L-N,** FACS analysis of apoptotic neutrophils in

blood (**L**, n=5), BM (**M**, n=5) and heart (**N**, n=5) of WT and S100a9K26R mice on day 1 post-MI/R. Mean ± SD. Statistical tests for (**K–M**): unpaired 2-tailed Student t t-test ($^{*}P < 0.05$ for indicated comparisons. ns, not significant). Statistical tests for (**N**): 2-way ANOVA with Tukey's multiple comparison test (*P* values adjusted for 6 comparisons; ns, not significant).

**Supplementary Figure 13**

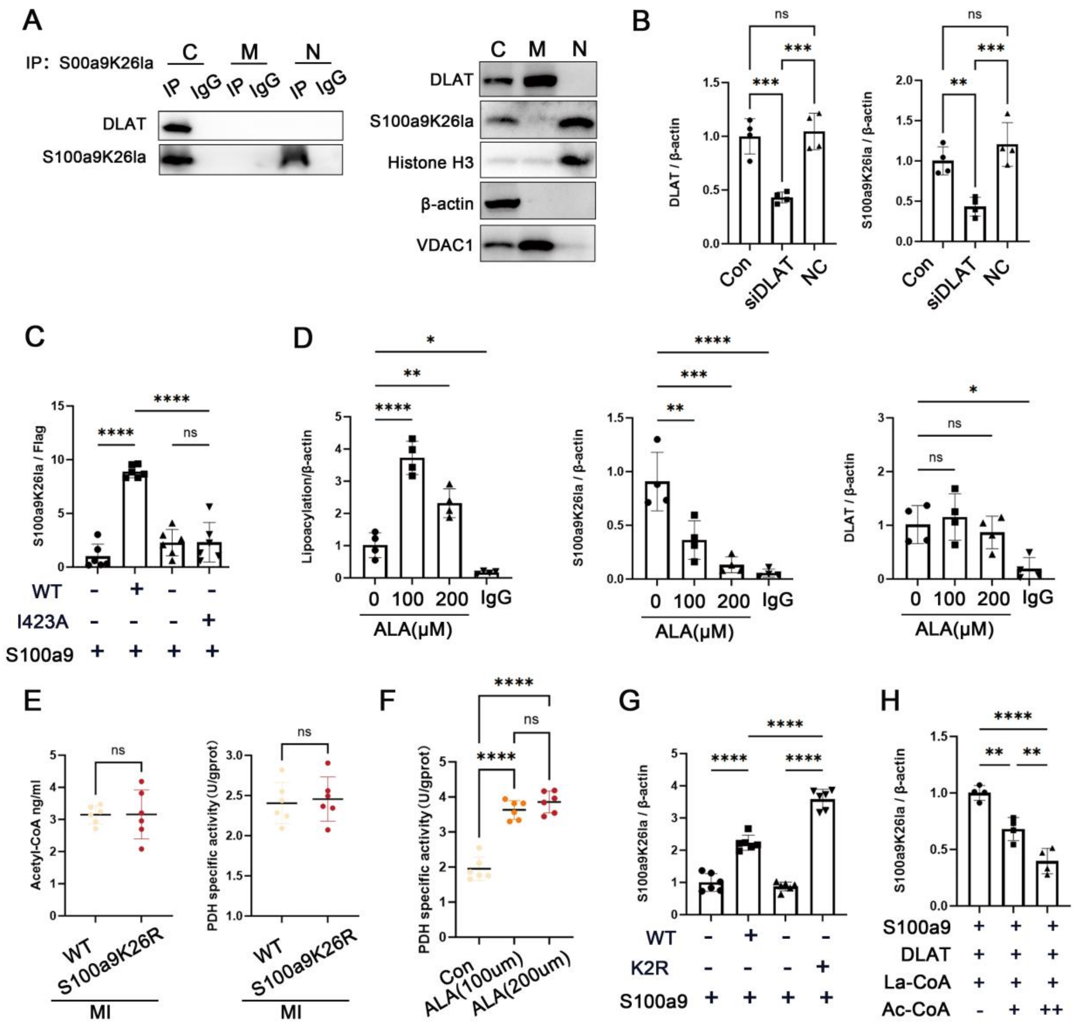


**Supplementary Figure 13: DLAT-catalyzed S100a9 lactylation in neutrophils can be antagonized by a-lipoic acid.**

**A,** Neutrophils of BM neutrophils post-MI/R were separated into the cytosolic (C), mitochondrial (M), and nuclear (N) fractions, and lysates were used for IP assay with the indicated antibody (n=4). **B**, 32Dcl3 murine IL-3 dependent cells were differentiated into granulocytes with the addition of G-CSF for 4 days and transfected with control small interfering RNA (siRNA; si-NC) or DLAT specific siRNA (si-DLAT) for 24 hours. The quantification of immunoblot in Figure 7F (n=4). Mean ± SD. Statistical tests for (**B**): 1-way ANOVA with Tukey's multiple comparison test (*P* values adjusted for 6 comparisons; $^{**}P < 0.01$, $^{***}P < 0.001$ for indicated comparisons. ns, not significant). **C,** Purified rS100a9 was incubated with immunoprecipitates of Flag-DLAT WT or enzyme-deficient I423A mutant from HEK-293 cells. The quantification of immunoblot in Figure 7I (n=6). Mean ± SD. Statistical tests for (**C**): 1-way ANOVA with Tukey's multiple comparison test (*P* values adjusted for 6 comparisons; $^{****}P < 0.0001$ for indicated comparisons. ns, not significant). **D**, BM neutrophils were stimulated with indicated doses of α-lipoic acid (ALA), then collected for IP assay with anti-DLAT and quantification of immunoblot in Figure 7J (n=4). Mean ± SD. Statistical tests for (**D**): 1-way ANOVA with Tukey's multiple comparison test (*P* values adjusted for 6 comparisons; $^{*}P < 0.05$, $^{**}P < 0.01$, $^{***}P < 0.001$, $^{****}P < 0.0001$ for indicated comparisons. ns, not significant). **E,** The PKH activity of BM neutrophils post-MI/R was examined using ELISA and the intracellular concentrations of acetyl-CoA were examined using enzyme-linked immunosorbent assay(n=6). Mean ± SD. Statistical tests for (**E**): unpaired 2-tailed Student t t-test (ns, not significant). **F,** BM neutrophils were stimulated with indicated doses of α-lipoic acid (ALA), then collected for the PKH activity assay (n=6). Mean ± SD. Statistical tests for (**F**): 1-way ANOVA with Tukey's multiple comparison test (*P* values

adjusted for 6 comparisons; $^{****}P < 0.0001$ for indicated comparisons. ns, not significant). **G,** Purified rS100a9 was incubated with immunoprecipitates of Flag-DLAT WT or K131/258R mutant from HEK293T cells, followed by western blot and quantification of immunoblot in Figure 7L (n=6). **H**, An in vitro lactylation assay was performed in the presence or absence of acetyl-CoA, followed by western blot and quantification of S100a9K26 lactylation in Figure 7M (n=4). Mean ± SD. Statistical tests for (**G and H**): 1-way ANOVA with Tukey's multiple comparison test ($P$ values adjusted for 6 comparisons; $^{*}P < 0.05$, $^{**}P < 0.01$, $^{***}P < 0.001$, $^{****}P < 0.0001$ for indicated comparisons. ns, not significant).

**Supplementary Figure 14**

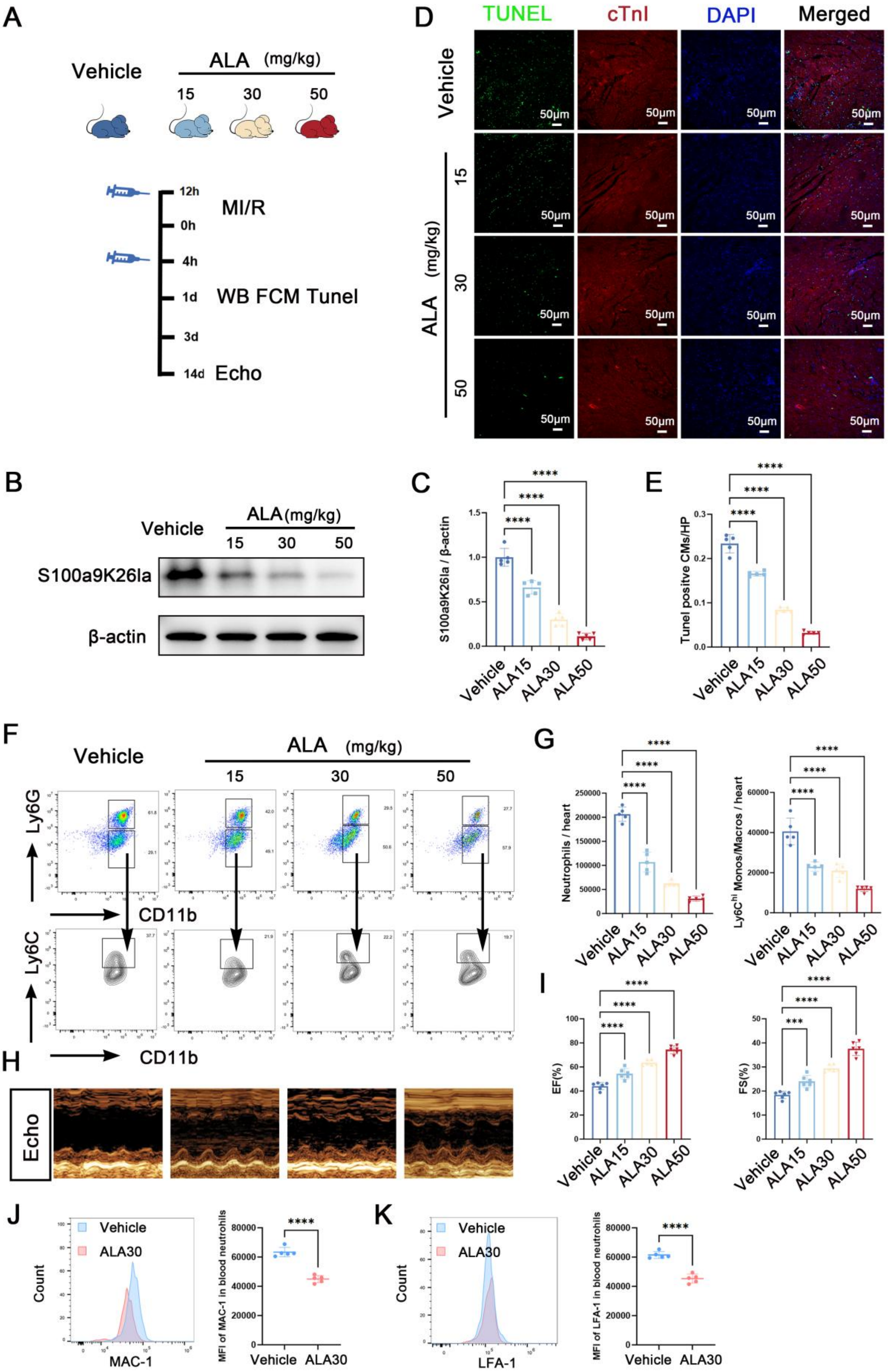


**Supplementary Figure 14:** ALA inhibits S100a9 lactylation and improves MI/R in a dose-dependent manner. **A,** Experimental protocol: Mice received intraperitoneal injection of

vehicle or ALA (15,30, 50mg/kg) at different time points. **B-C,** S100a9K26la levels in circulating neutrophils were assayed by western blot (n=5). **D-E,** Representative images of TUNEL and cTnI double staining and its quantitation (n=5). **F-G,** Representative flow cytometry plots and quantification of cardiac infiltrating $CD45^{+}CD11b^{+}Ly6G^{-}Ly6C^{hi}$ monocytes/macrophages and $CD45^{+}CD11b^{+}Ly6G^{+}$ neutrophils day 1 post-MI/R (n=5). **H-I,** Representative M-mode echocardiograms and echocardiogram measurements (n=6). Mean ± SD. Statistical tests for (**C, E, G, and I**): 1-way ANOVA with Tukey's multiple comparison test (*P* values adjusted for 6 comparisons; $^{***}P < 0.001$, $^{****}P < 0.0001$ for indicated comparisons). **J-K**: FACS analysis of MAC-1 (**J**) and LFA-1 (**K**) protein expression of neutrophils from the blood of WT and S100a9K26R mice on day 1 post-MI/R (n=4). MFI, mean fluorescence intensity. Mean ± SD. Statistical tests for (**J-K**): unpaired 2-tailed Student t t-test ($^{****}P < 0.0001$ for indicated comparisons. ns, not significant).

**Supplementary Figure 15**

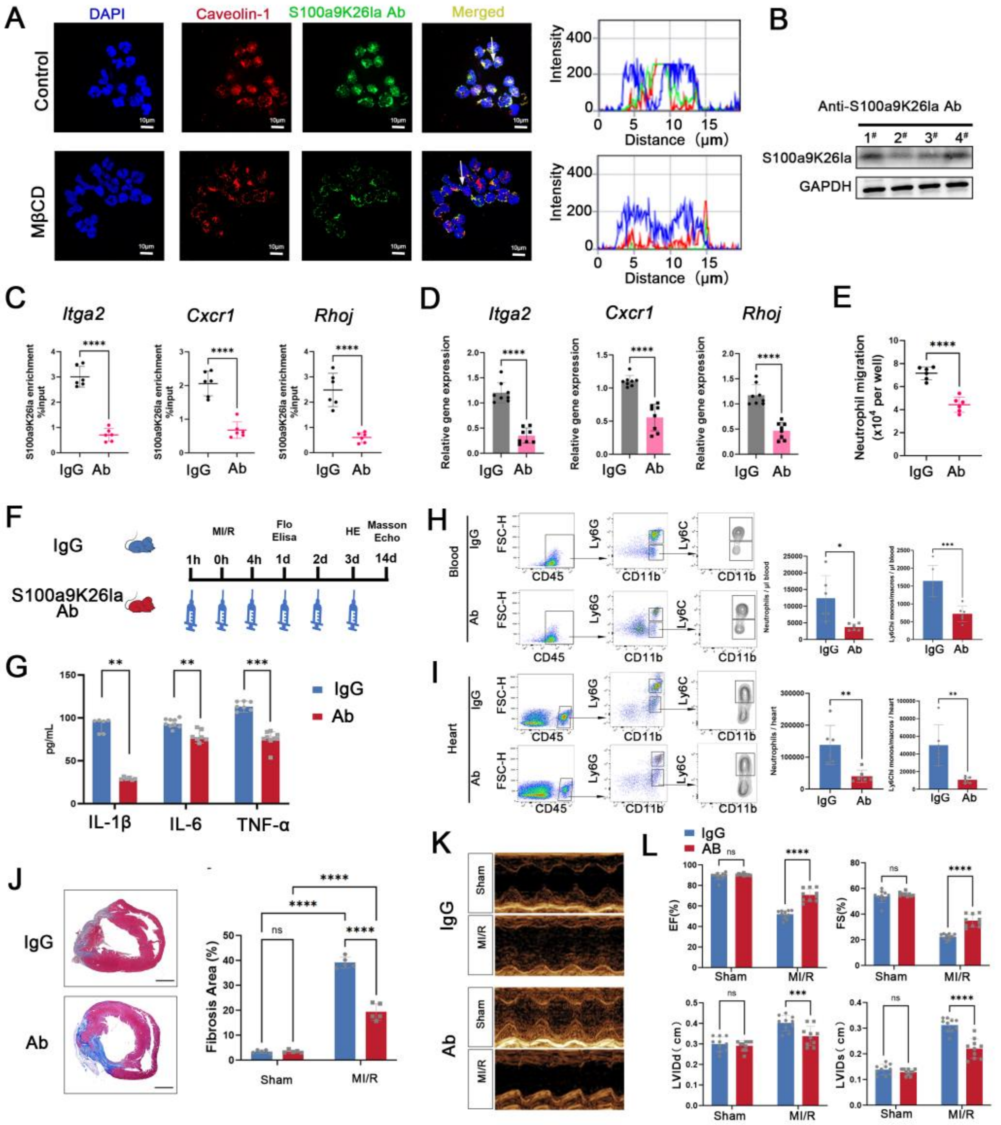


**Supplementary Figure 15: Blockade of S100a9K26la inhibits inflammation and improves cardiac function post MI/R.**

**A-E,** We isolated circulating neutrophils from wt mice 1 day post-MI/R and treated with 10 ug S100a9K26la antibody for 2 hours with or without methyl-ß-cyclodextrin (MβCD, an inhibitor of caveolae-dependent endocytosis). **A**, Immunofluorescence showed that S100a9K26la antibody conjugated with 488 dye (green) was located in the intracellular of neutrophils and bound to caveolin-1 (red, the specific marker of caveolae) on the surfaces of the cell membrane, which was presented with yellow fluorescence (scale bar=10 µm, n=4). **B**,

Antibody visualization in cytosolic fractions of neutrophils using HRP-coupled anti-rabbit-IgG antibodies (n=8). **C**, Gene expression analysis by RT-qPCR (n=8). **D**, S100a9K26la occupancy analysis by ChIP-qPCR (n=6). **E**, The migration assay was determined by Transwell (n=6). Mean ± SD. Statistical tests for (**C–E**): unpaired 2-tailed Student t t-test ($^{****}P < 0.0001$ for indicated comparisons). **F,** Experimental protocol. Mice received injection intravenously of S100a9K26la mAb or isotype control IgG (100μg/mouse) at different time points. **G,** IL-1β, IL-6 and TNF-α in serum were assayed by ELISA (n≥6). **H-I,** Representative flow cytometry plots and quantification of peripheral blood (H, n=6) and cardiac infiltrating (I, n=6) $CD45^+CD11b^+Ly6G^-Ly6C^{hi}$ monocytes/macrophages and $CD45^+CD11b^+Ly6G^+$ neutrophils day 1 post-MI/R. Statistical tests for (**H–I**): unpaired 2-tailed Student t t-test ($^{*}P < 0.05$, $^{**}P < 0.01$, and $^{***}P < 0.001$ for indicated comparisons). **J,** Masson trichrome staining and quantitative analysis of fibrotic area (scale bar=1 mm, n=6). **K-L, Representative** M-mode echocardiograms and echocardiogram measurements (n=10). Mean ± SD. Statistical tests for (**G, K, and L**):1-way ANOVA with Tukey's multiple comparison test ($P$ values adjusted for 6 comparisons; $^{**}P < 0.01$, $^{***}P < 0.001$, and $^{****}P < 0.0001$ for indicated comparisons. ns, not significant).

**Supplementary Figure 16**

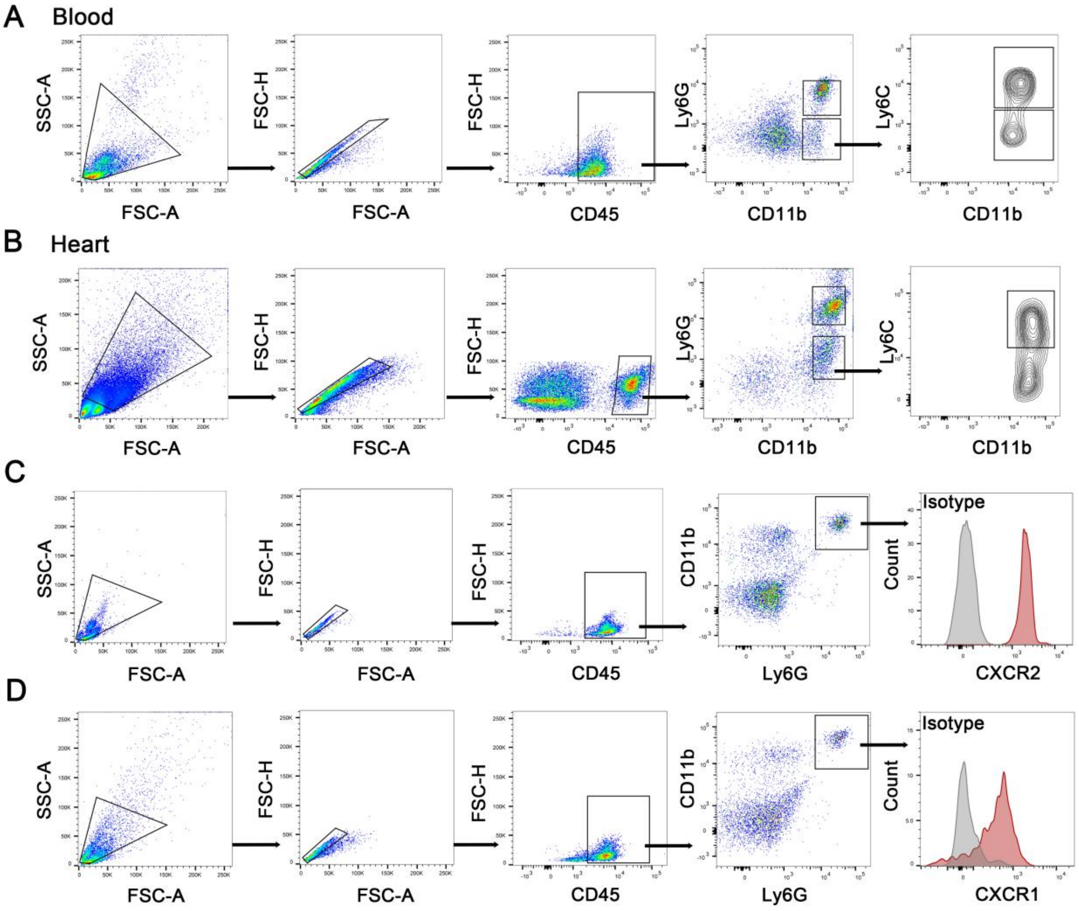


**Supplementary Figure 16: Gating strategy for identification of leukocytes.**

**A-B,** RBC-free single cell suspensions were prepared from blood samples (A) and heart tissue digests (B) were stained. Once the doublets (by FSC-H vs. FSC-A) were excluded, monocytes and macrophages were identified as $CD45^{+}CD11b^{+}Ly6G^{-}$ and further classified as $Ly6C^{hi}$ and $Ly6C^{lo}$; neutrophils as $CD45^{+}CD11b^{+}Ly6G^{+}$. **C-D**, Histograms were pre-gated on $CD45^{+}Ly6G^{+}$ cells. MFI of CXCR2 fluorescence was analyzed in $CD45^{+}Ly6G^{+}$ neutrophils (C); MFI of CXCR1 fluorescence was analyzed in $CD45^{+}Ly6G^{+}$ neutrophils (D).

**Supplementary Figure 17**

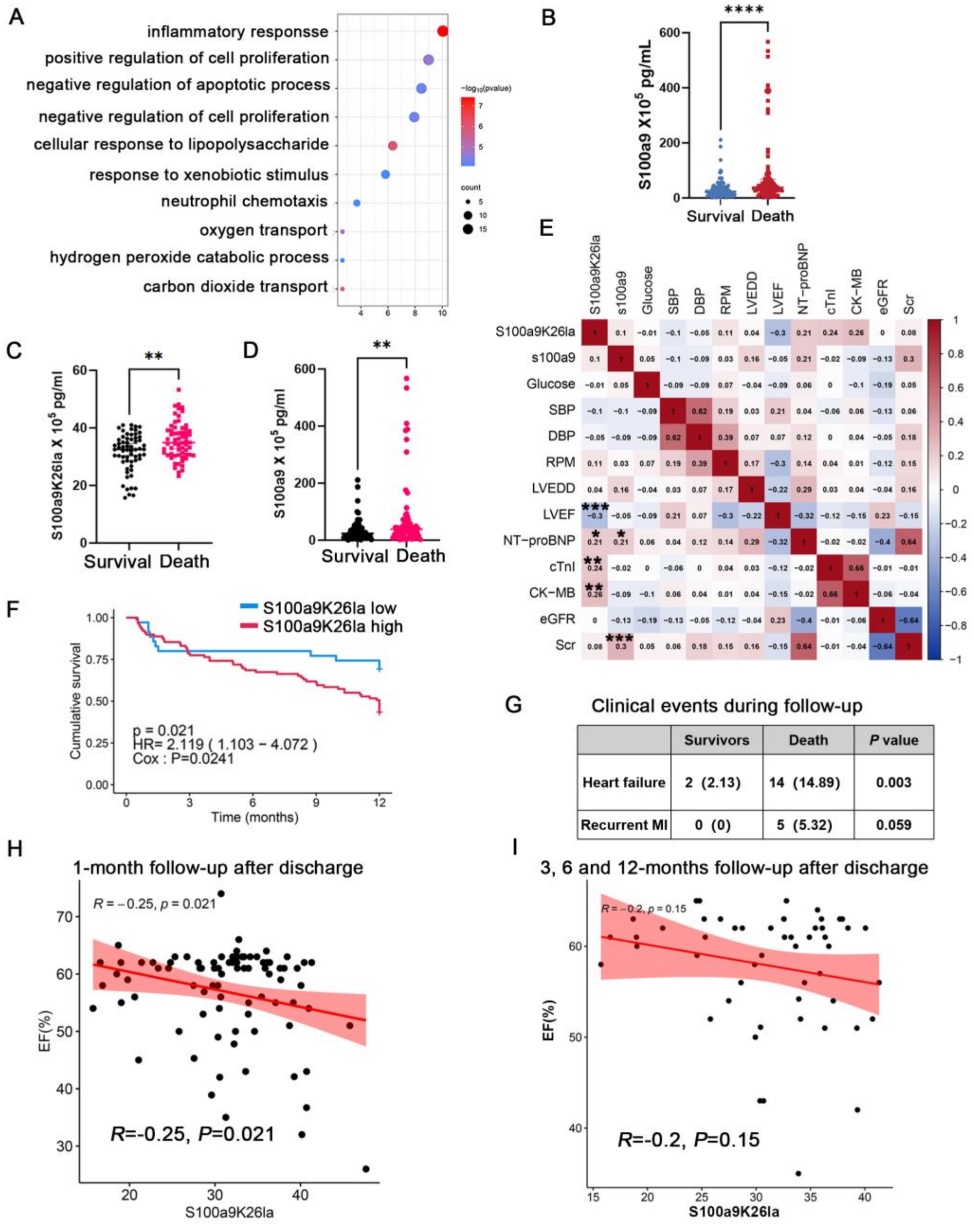


| | Survivors | Death | *P* value |
|---|---|---|---|
| Heart failure | 2 (2.13) | 14 (14.89) | 0.003 |
| Recurrent MI | 0 (0) | 5 (5.32) | 0.059 |

**Supplementary Figure 17: K26-Lactylated S100a9 is associated with cardiac death in AMI patients**.

**A,** GO analysis of DEGs from transcriptome analysis**. B,** Measurement of plasma S100a9 levels by ELISA. Median (interquartile ranges, 25th-75th percentile). Statistical tests for (**B**): 2-tailed Mann–Whitney test ($^{****}P$ < 0.0001 for indicated comparisons). **C-F,** We excluded the patients with infection in the cardiac death cohort, the indicated analysis has been performed (n=124). Measurement of plasma S100a9 levels by ELISA (**C and D**);

Spearman's correlation analysis of S100a9K26la and S100a9 with myocardial injury markers and cardiac function indexes at baseline (**E**); Kaplan–Meier plots of long-term cardiac death based on high or low S100a9K26la levels (cut-off: $30.19483\times10^{5}$pg/ml) on day 1 post-PCI in patients with AMI (**F**). Median (interquartile ranges, 25th-75th percentile). Statistical tests for (**C and D**): 2-tailed Mann–Whitney test ($^{**}P < 0.01$ for indicated comparisons). **G,** Intercurrent clinical events in the 94 death cases and 94 survivor-matched controls during 1-year follow-up. Data are expressed as number of subjects (%). Group comparisons used fisher exact test. **H-I,** Spearman's correlation analysis of S100a9K26la at baseline and ejection fraction (EF) at the first month follow-up (H) and EF at the 3rd, 6th, and 12th month follow-up (I) in AMI patients.

## References

1. Gao E, et al. A novel and efficient model of coronary artery ligation and myocardial infarction in the mouse. *Circ Res* 2010;107(12):1445-53.

2. Zacchigna S, et al. Towards standardization of echocardiography for the evaluation of left ventricular function in adult rodents: a position paper of the ESC Working Group on Myocardial Function. *Cardiovasc Res* 2021;117(1):43-59.

3. Thygesen K, et al. Fourth Universal Definition of Myocardial Infarction (2018). *J Am Coll Cardiol* 2018;72:2231-2264.

4. Jiang S, et al. Identification of High-Risk Coronary Lesions by 3-Vessel Optical Coherence Tomography. *J Am Coll Cardiol*. 2023;81(13):1217-1230.
