# Supplementary material for: S100a9 lactylation triggers neutrophil trafficking and cardiac inflammation in myocardial ischemia/reperfusion injury
Source: J Clin Invest. 2025 Oct 9;135(24):e194664. doi: 10.1172/JCI194664 (PMC12700540; doi:10.1172/JCI194664)

Main Figures

Full unedited gel for Figure F1H

Representative images(EXP1.)

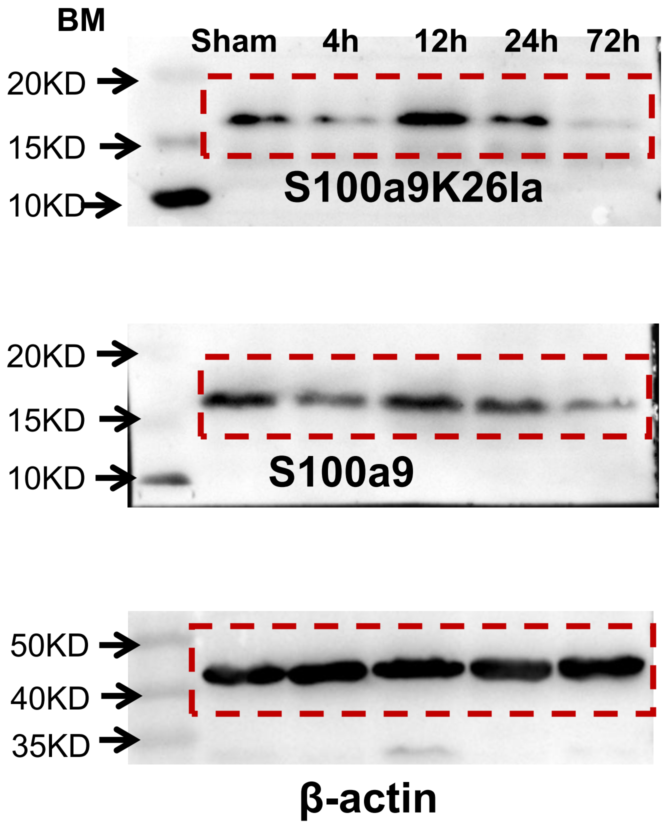

Quantification images(EXP1-6.)

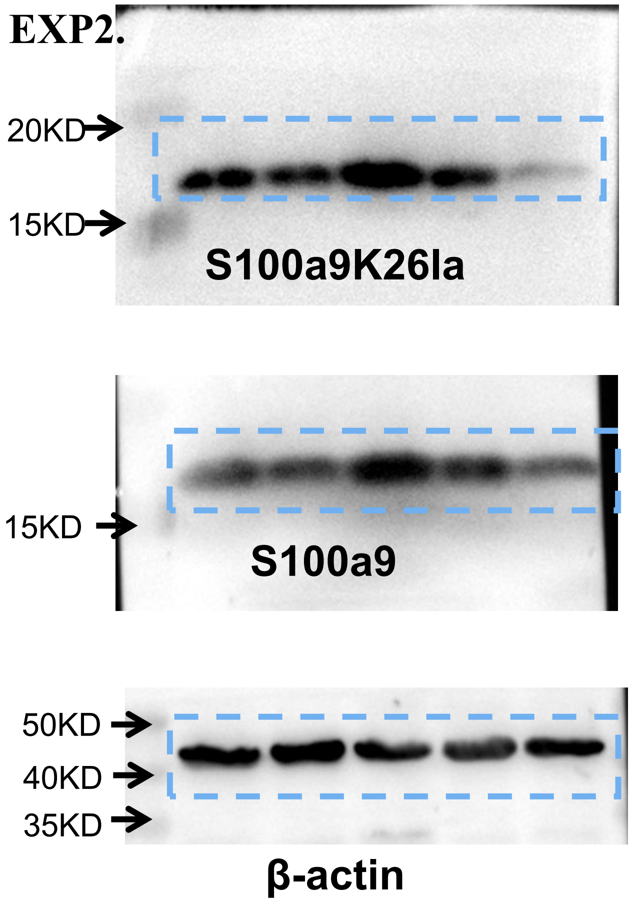

Full unedited gel for Figure F1H

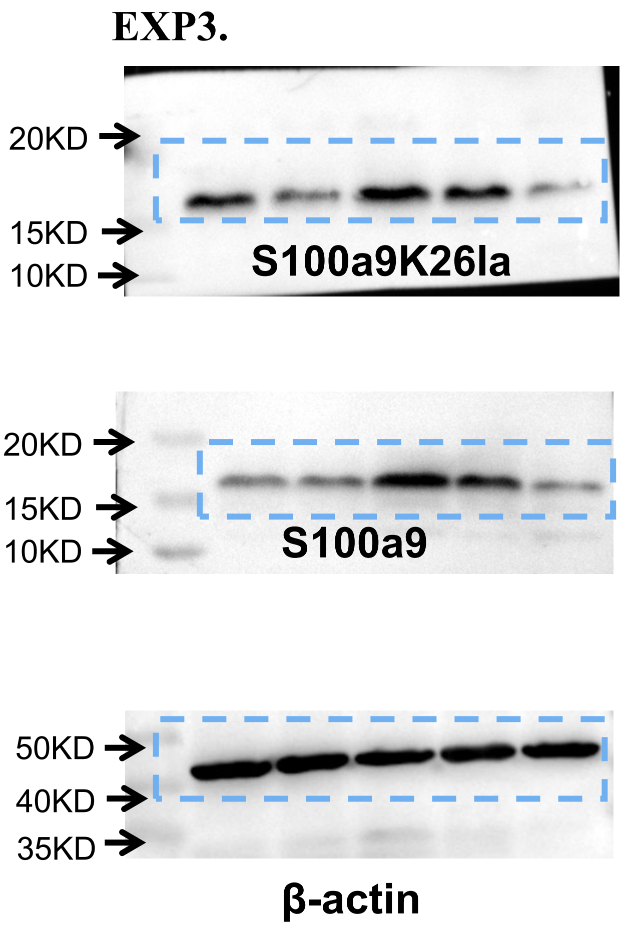

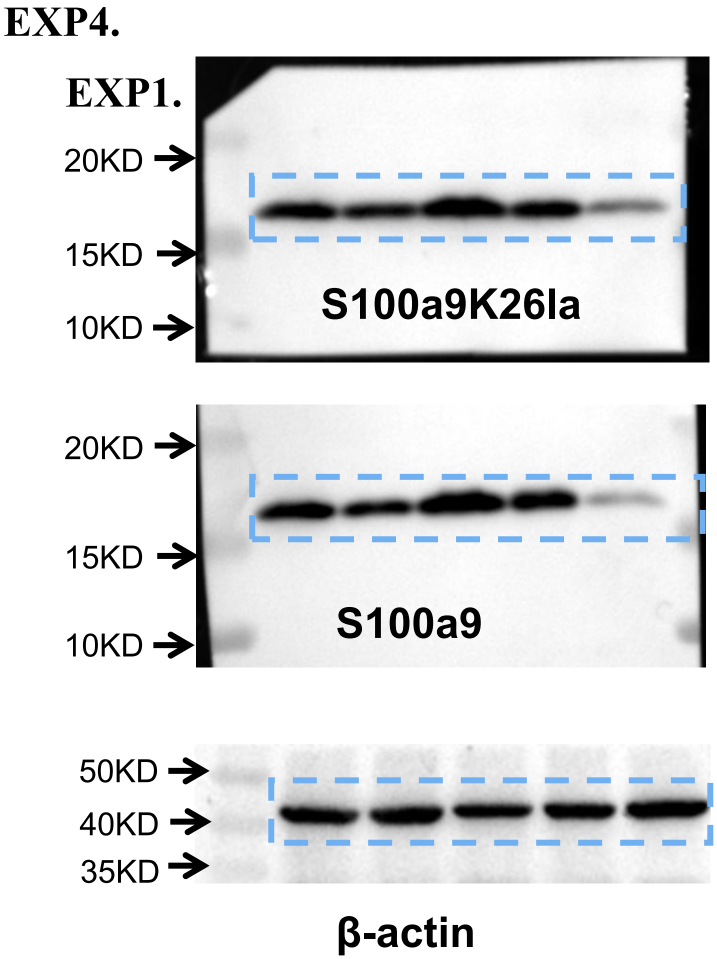

Full unedited gel for Figure F1H

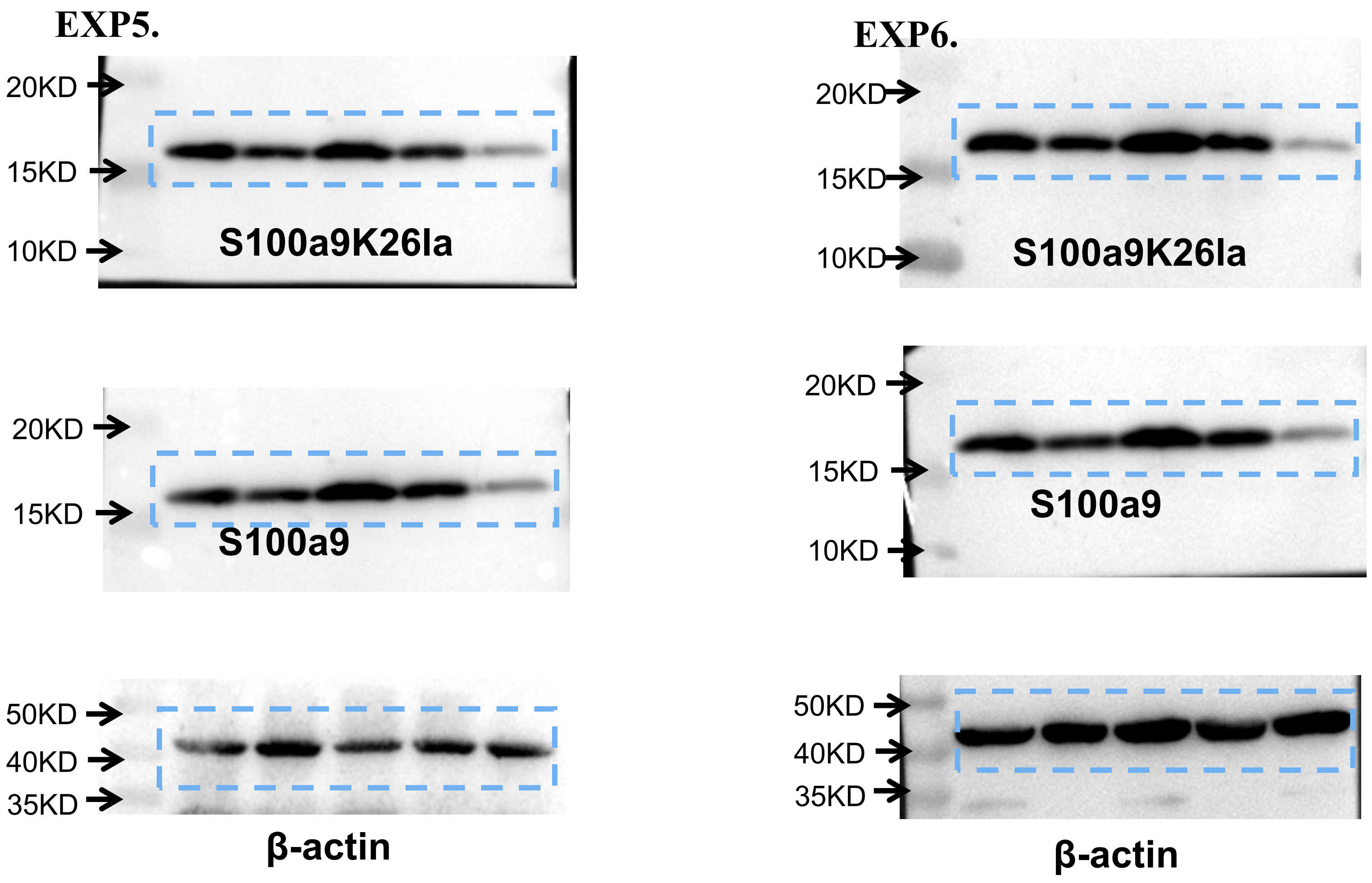

Full unedited gel for Figure F1J

Representative images(EXP1.)

Quantification images(EXP1-10.)

EXP1.

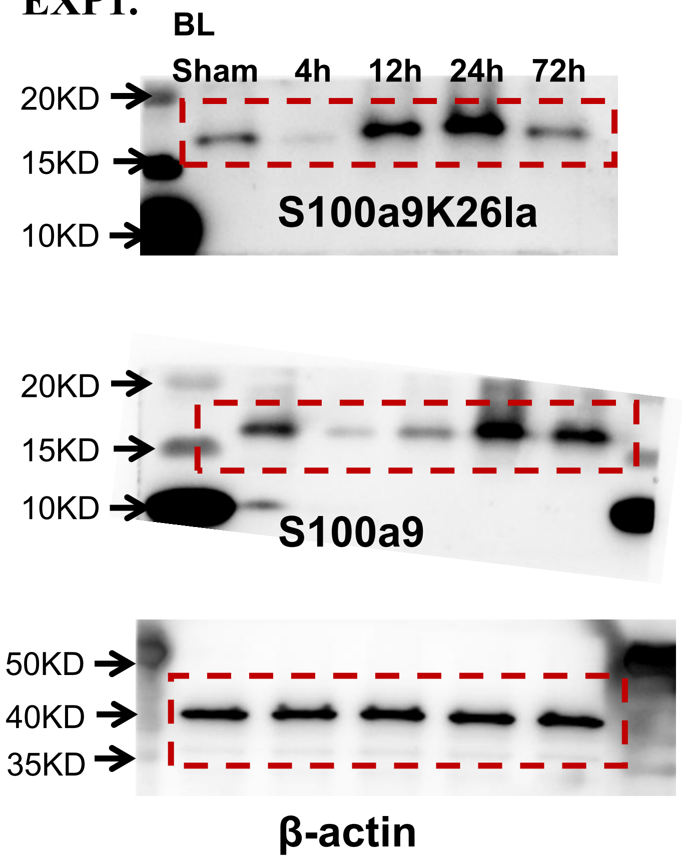

EXP2.

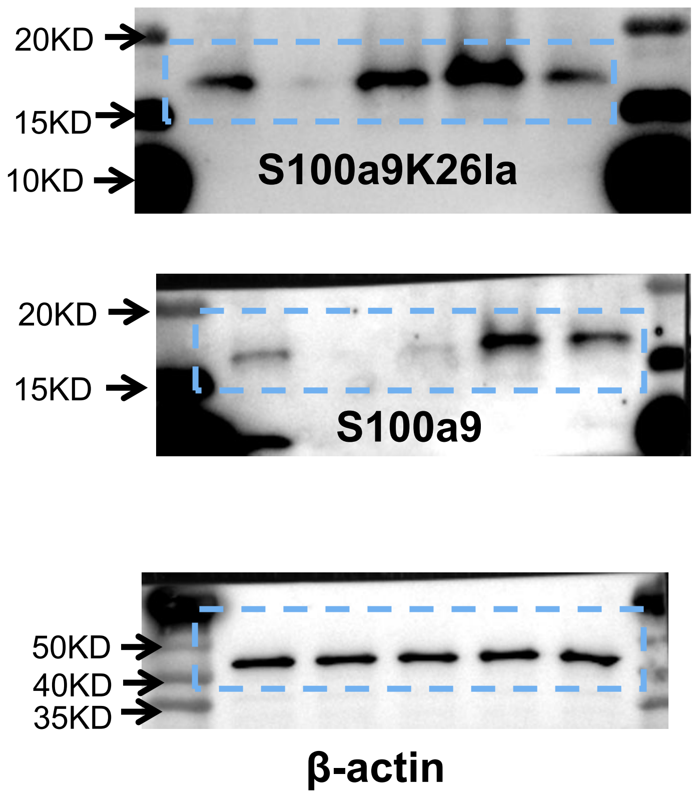

Full unedited gel for Figure F1J

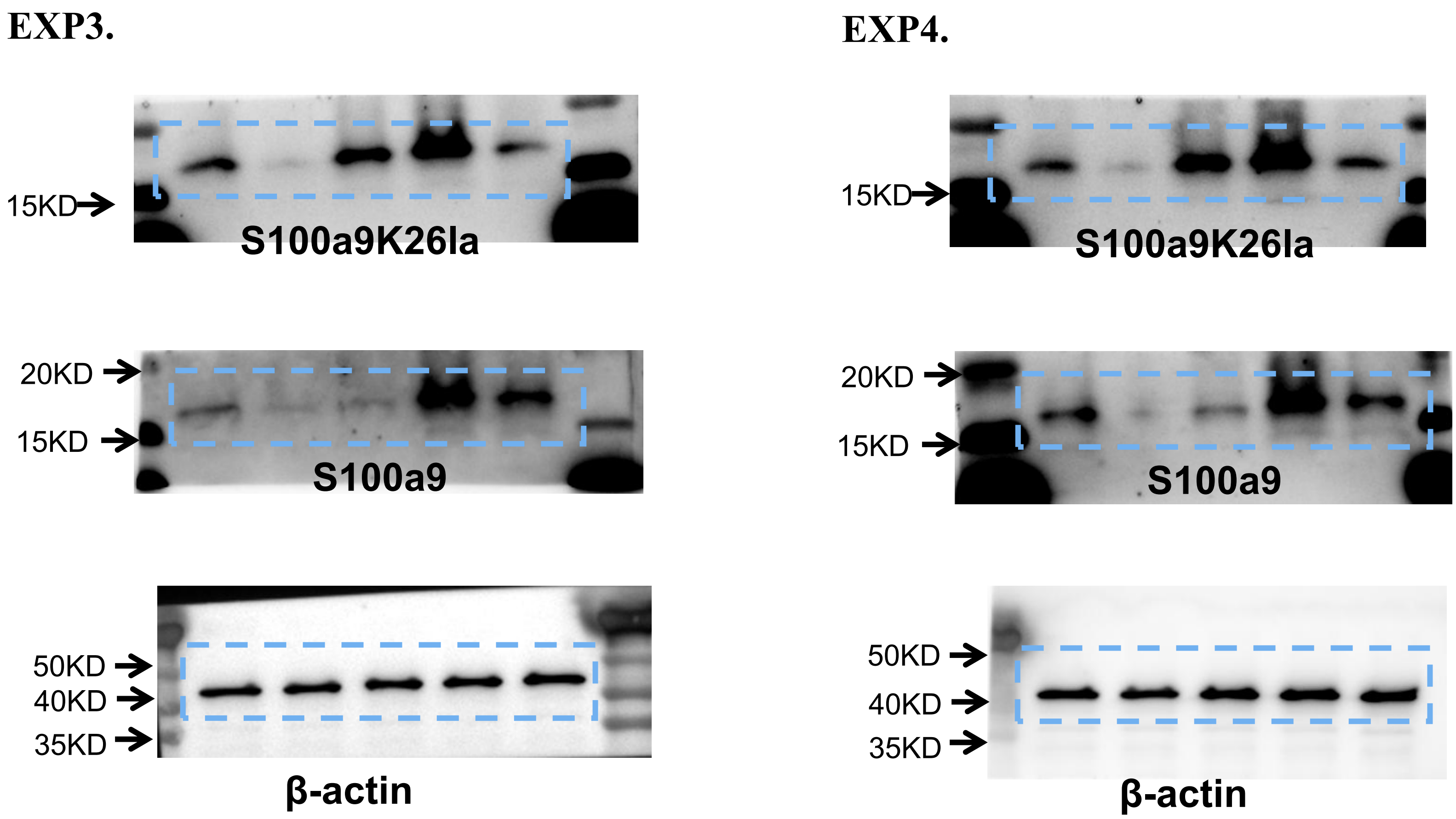

Full unedited gel for Figure F1J

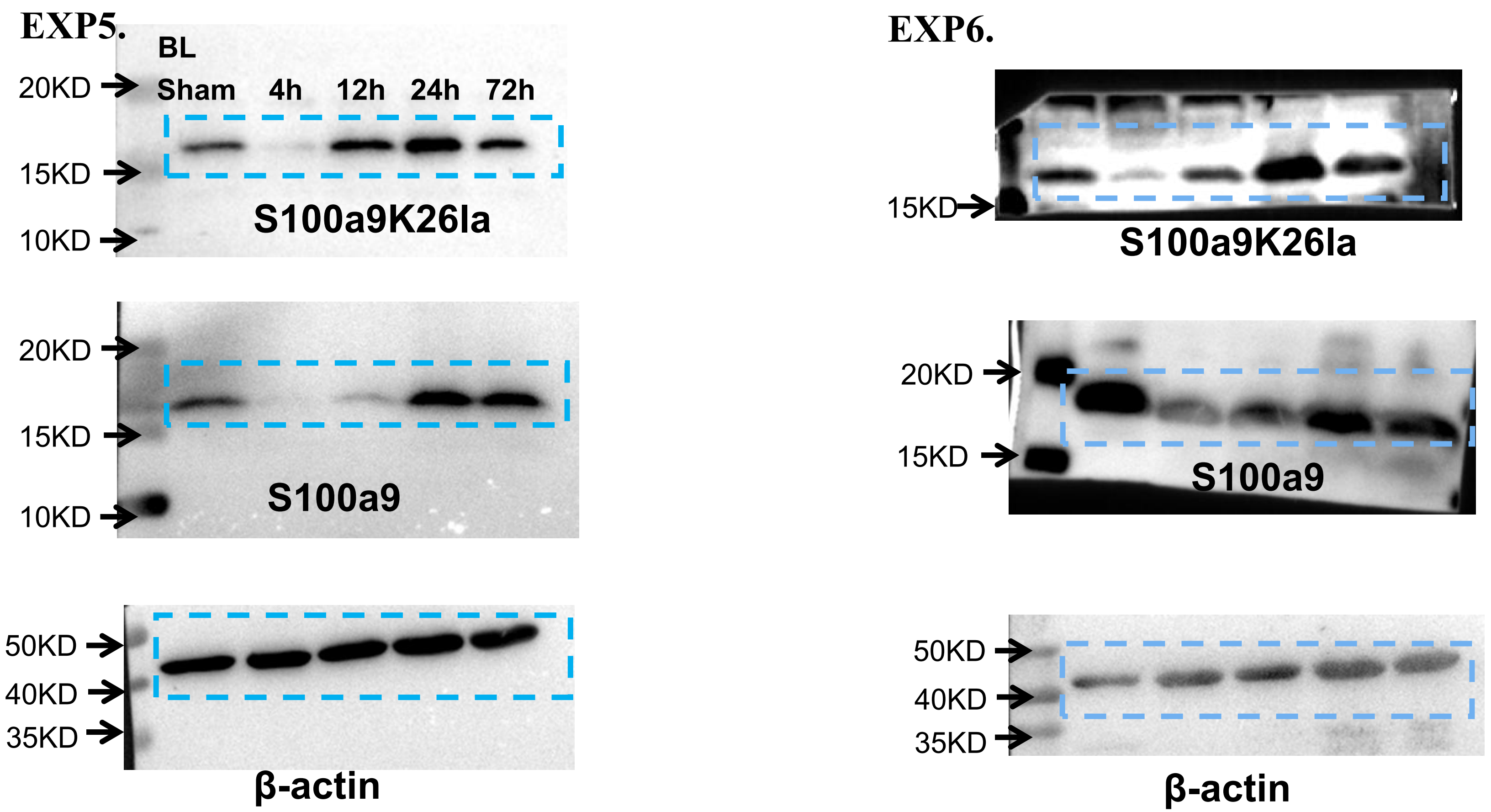

Full unedited gel for Figure F1J

EXP7.

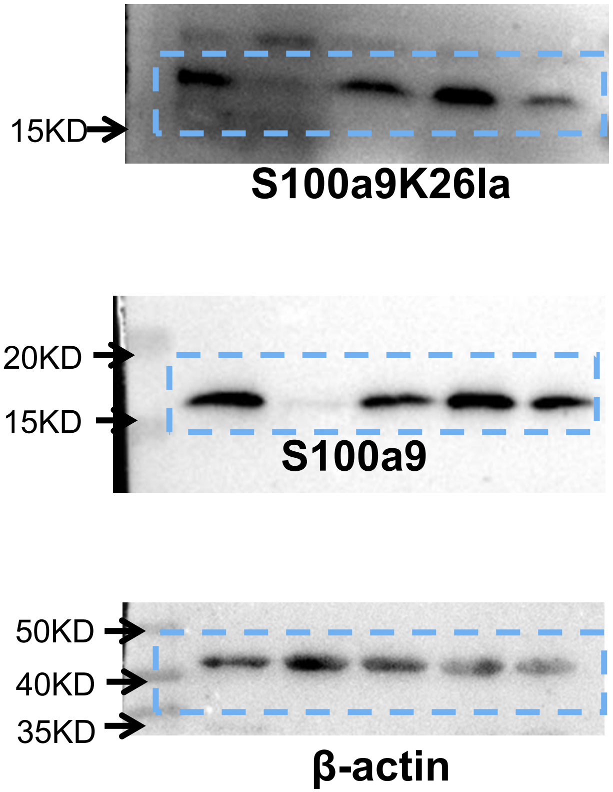

EXP8.

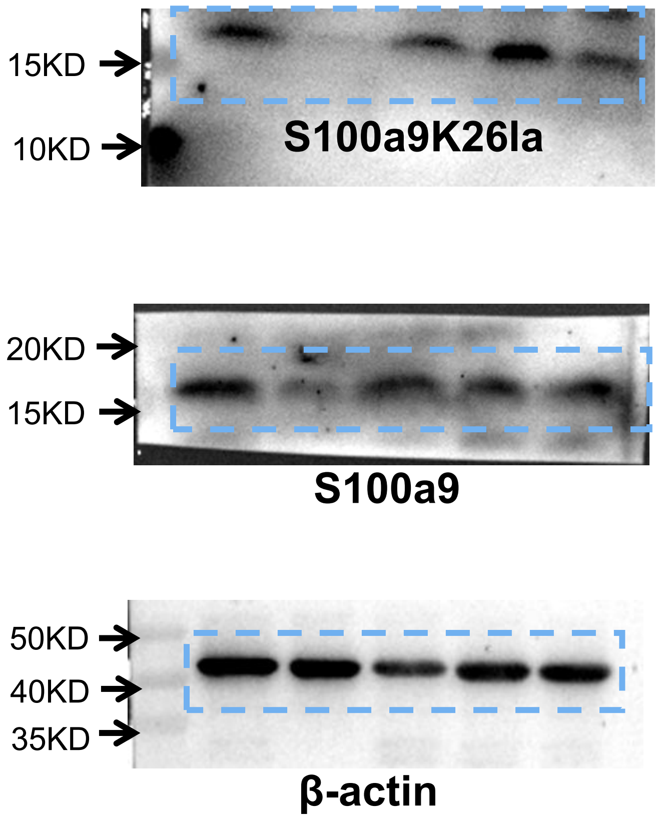

Full unedited gel for Figure F1J

EXP9.

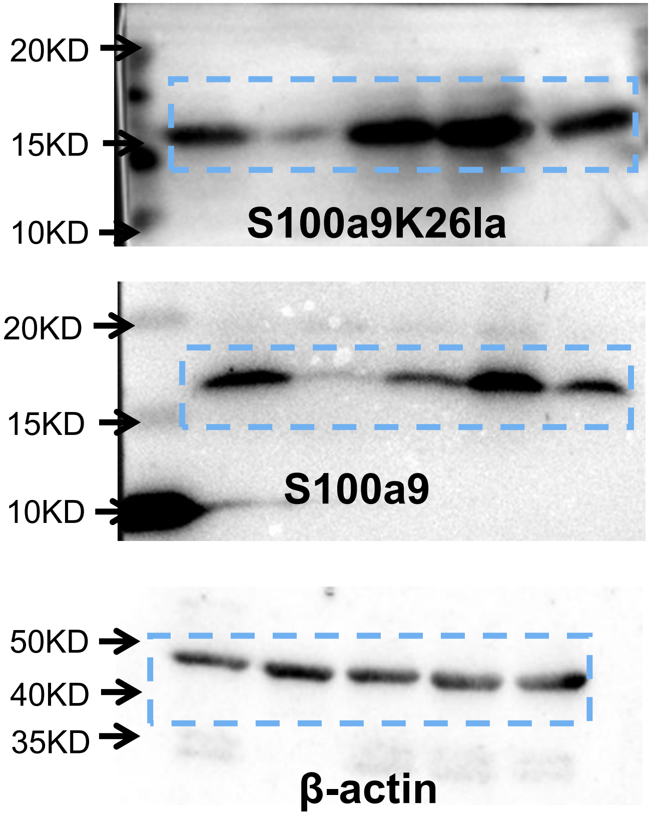

EXP10.

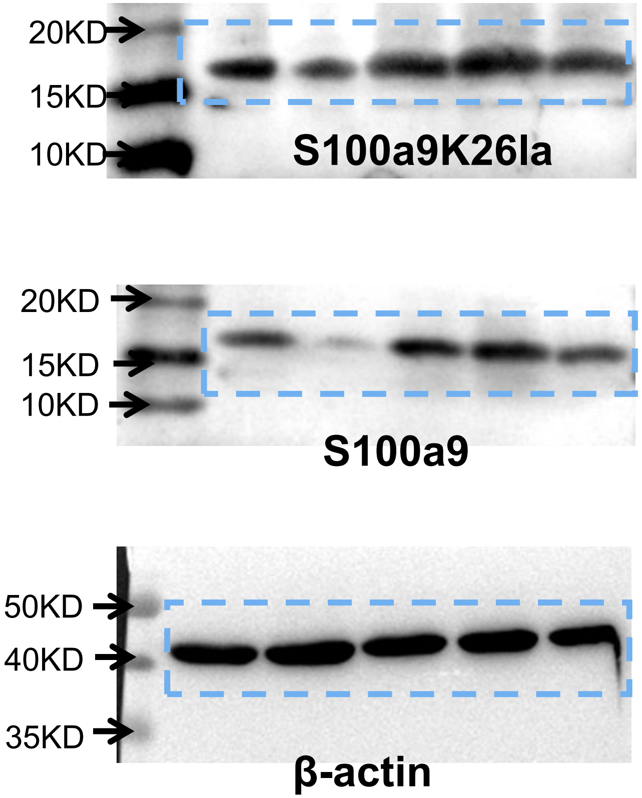

Full unedited gel for Figure F1N

Representative images

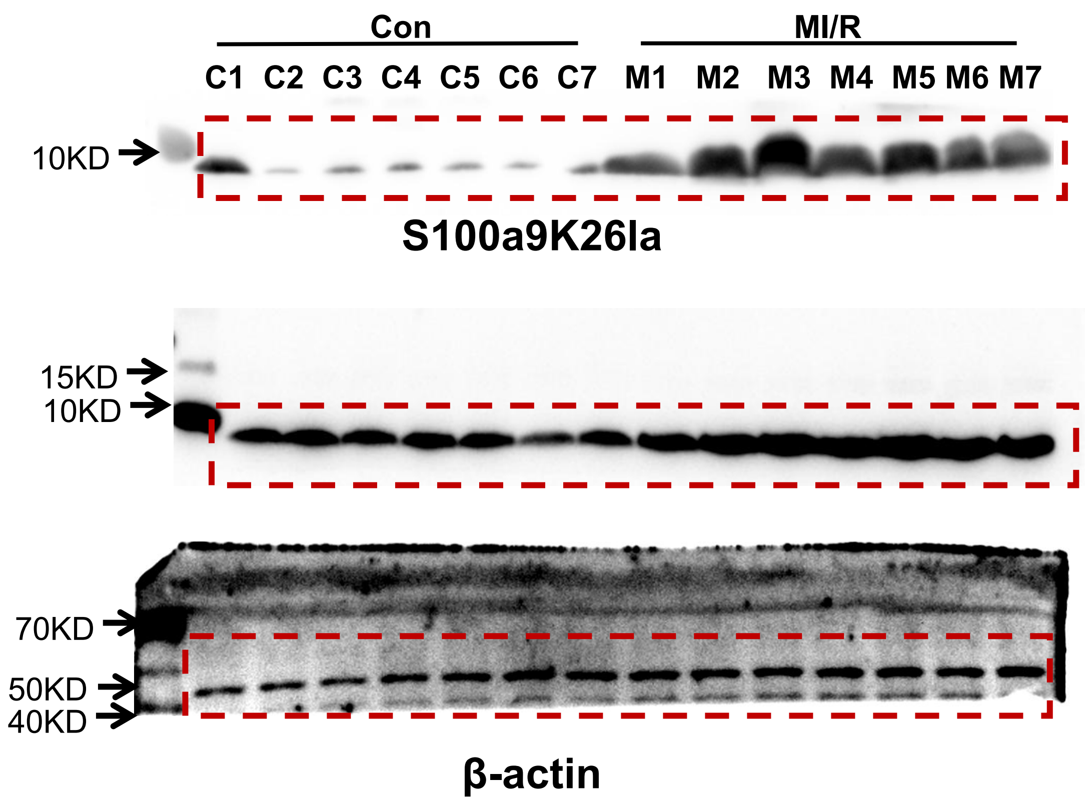

Full unedited gel for Figure 4A

Representative images(EXP1.)

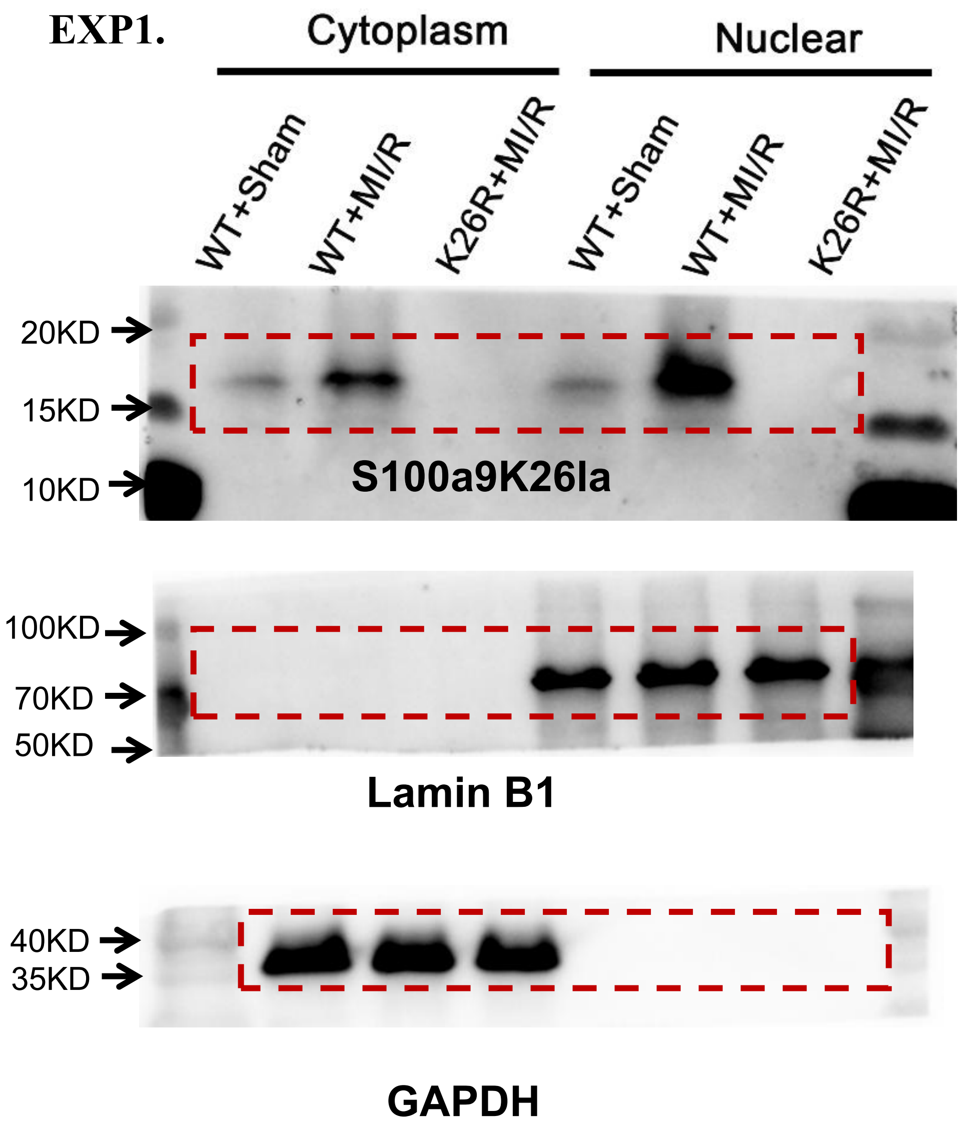

Quantification images(EXP1-4.)

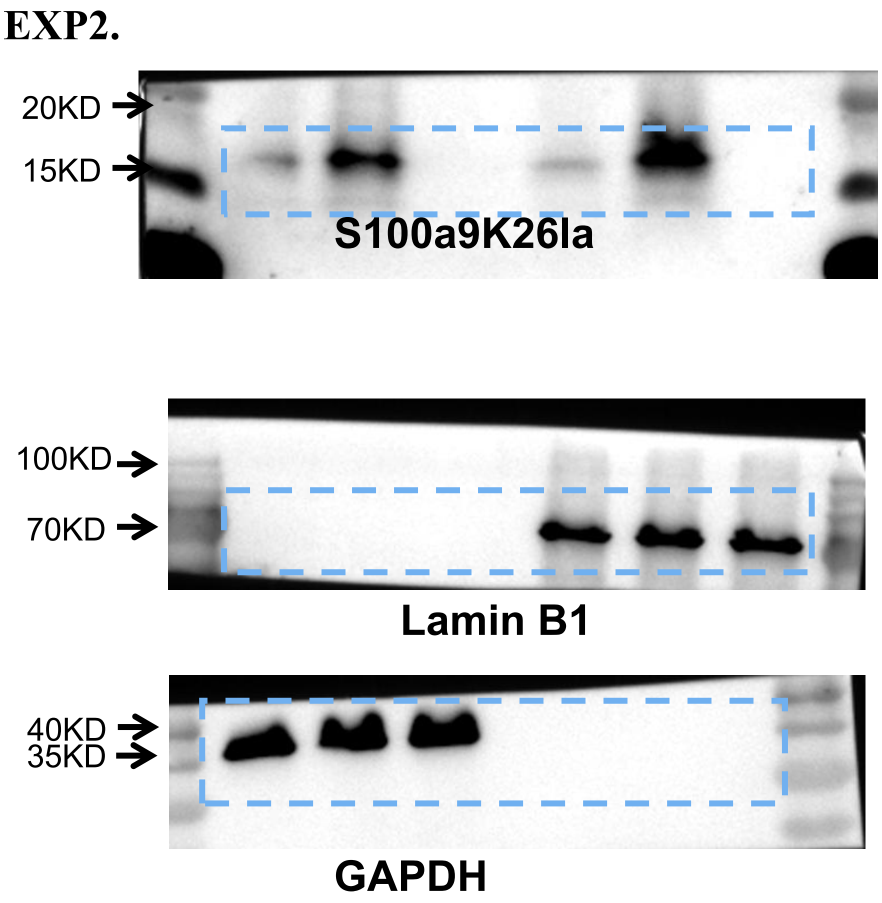

Full unedited gel for Figure 4A

EXP3.

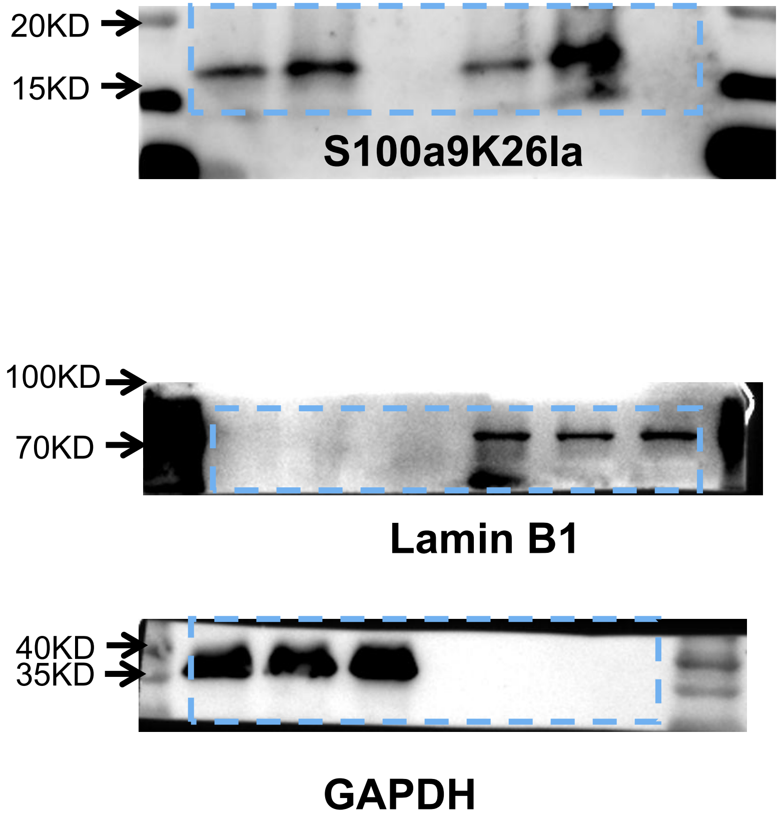

EXP4.

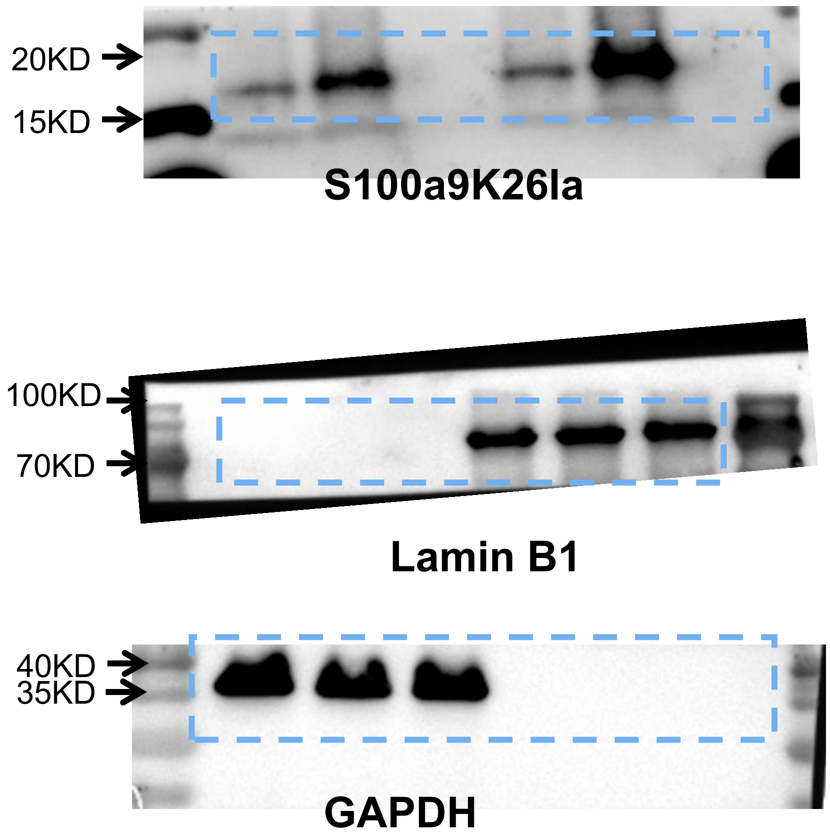

Full unedited gel for Figure F5C

Representative images(EXP1.)

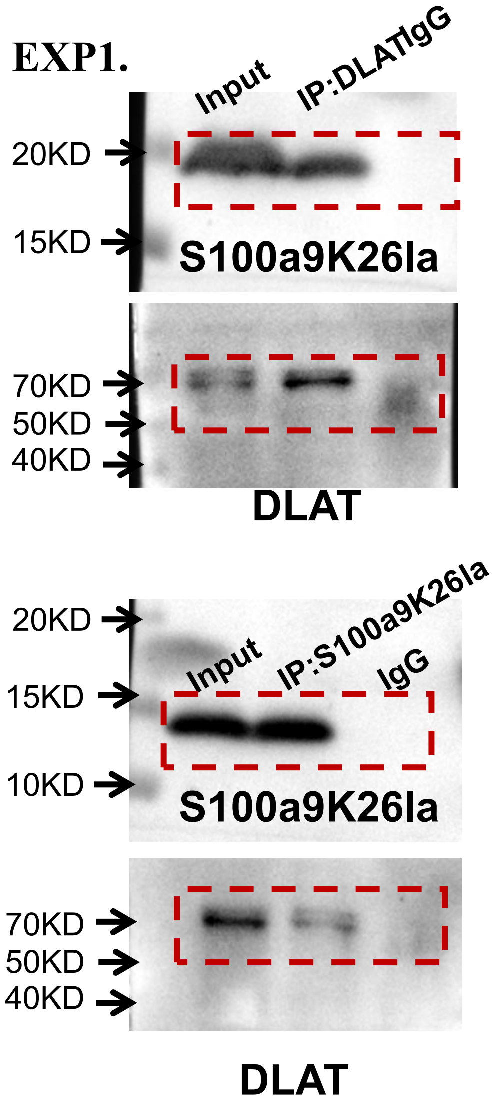

Quantification images(EXP1-4.)

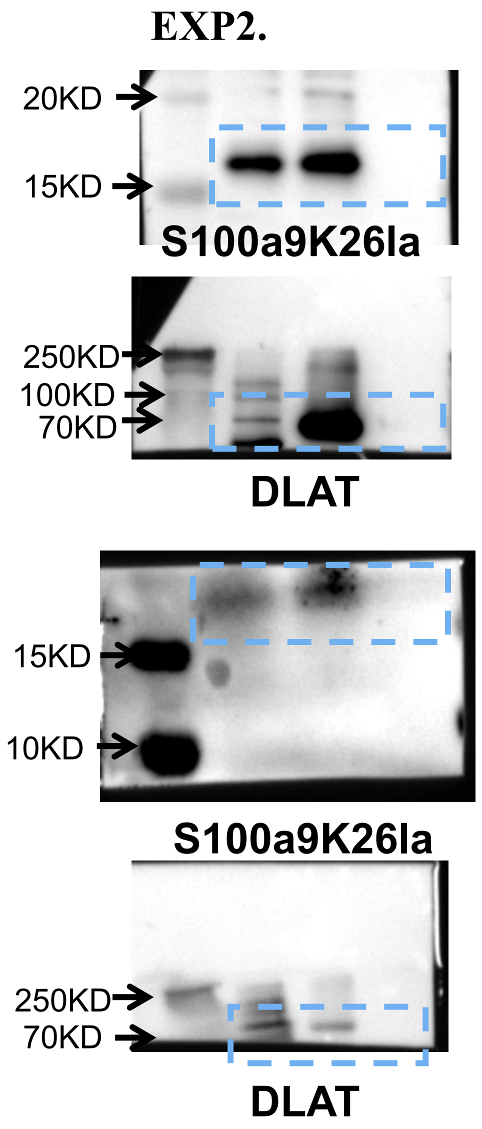

Full unedited gel for Figure F5C

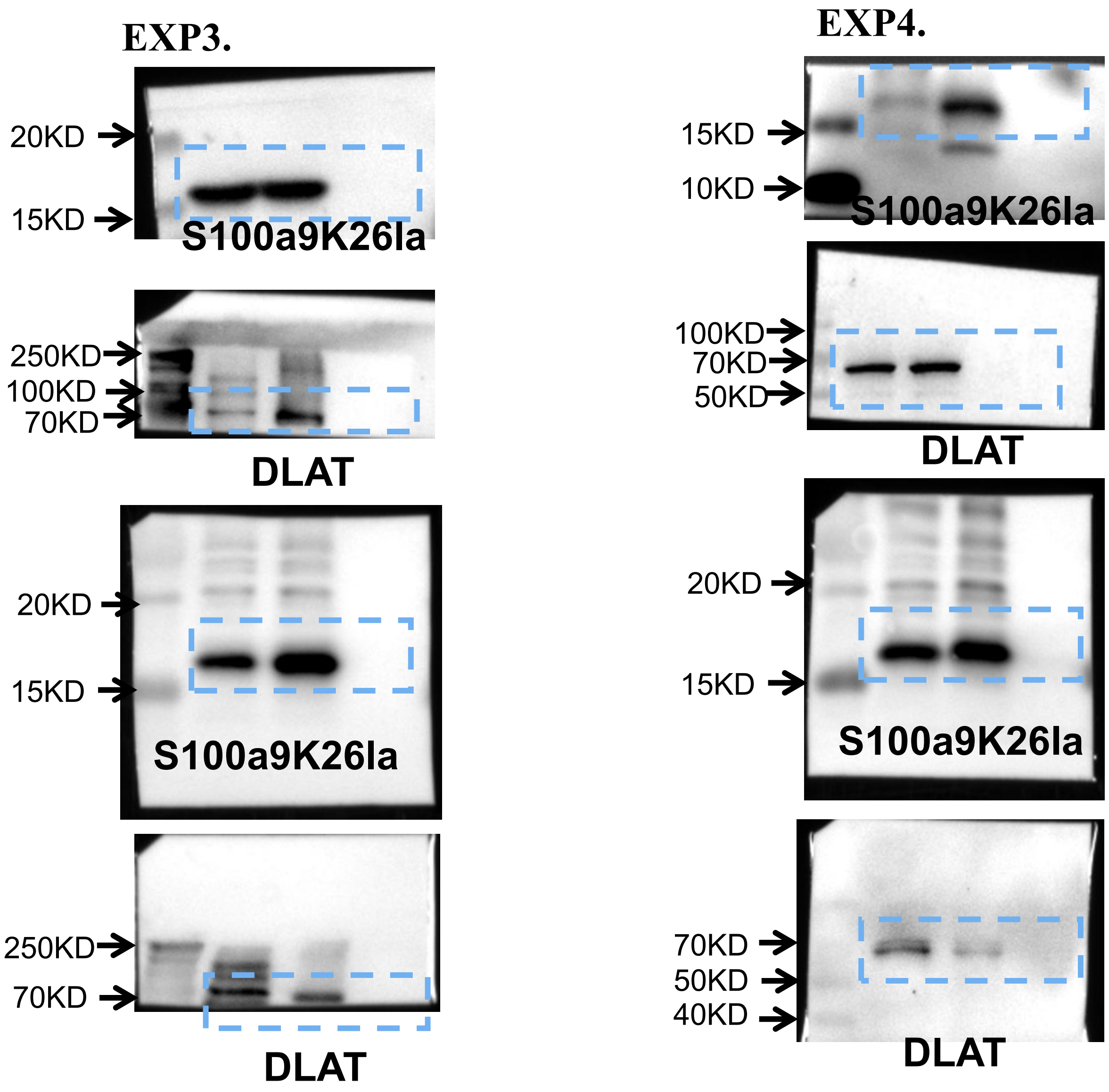

Full unedited gel for Figure F5E

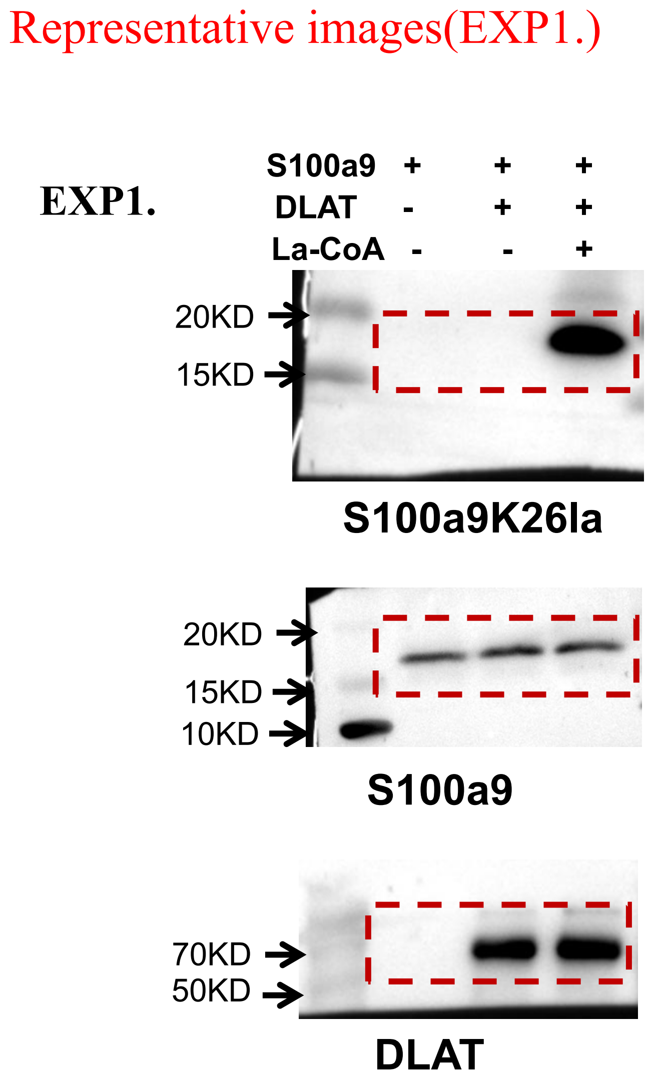

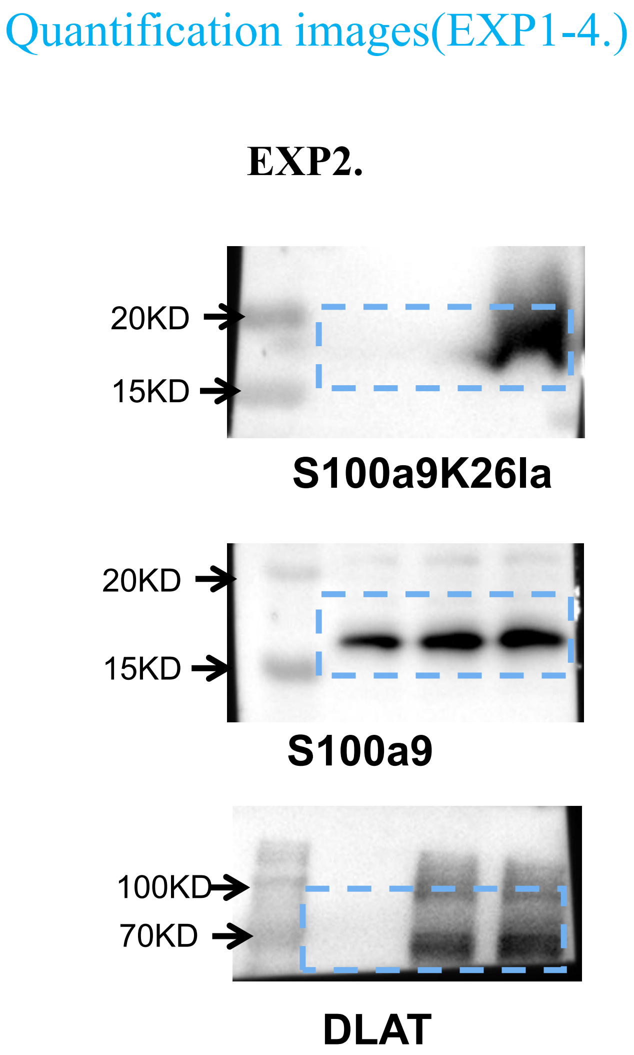

Full unedited gel for Figure F5E

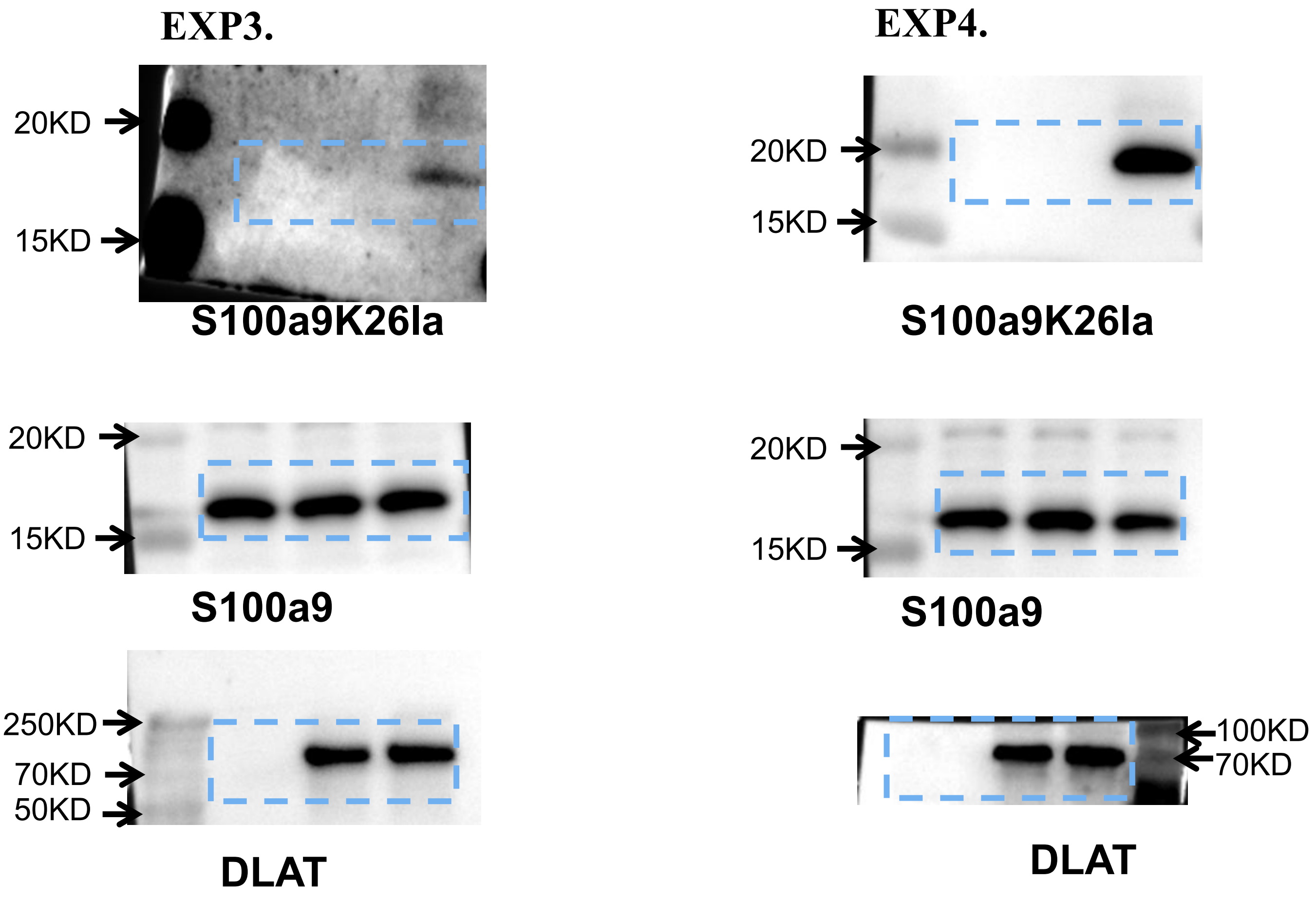

Full unedited gel for Figure F5F

Representative images(EXP1.)

Quantification images(EXP1-4.)

EXP1.

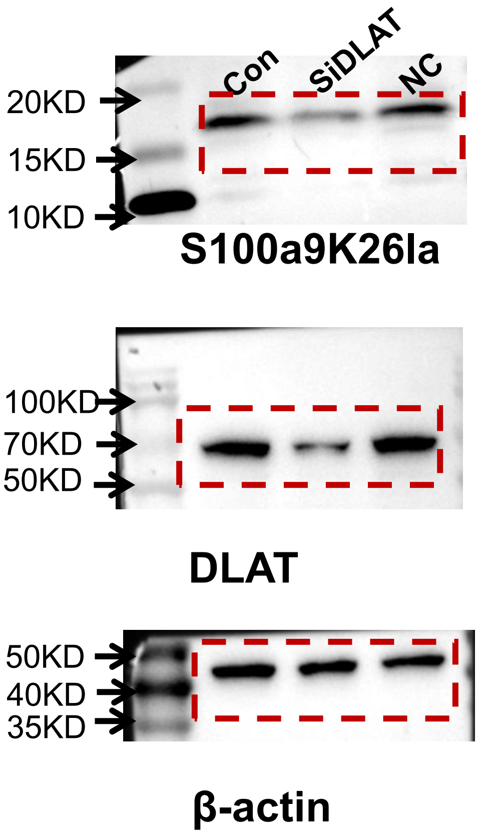

EXP2.

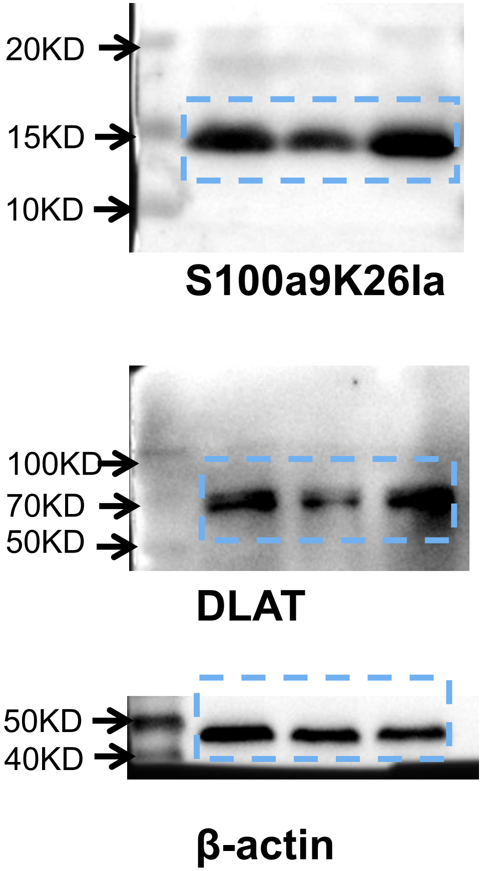

Full unedited gel for Figure F5F

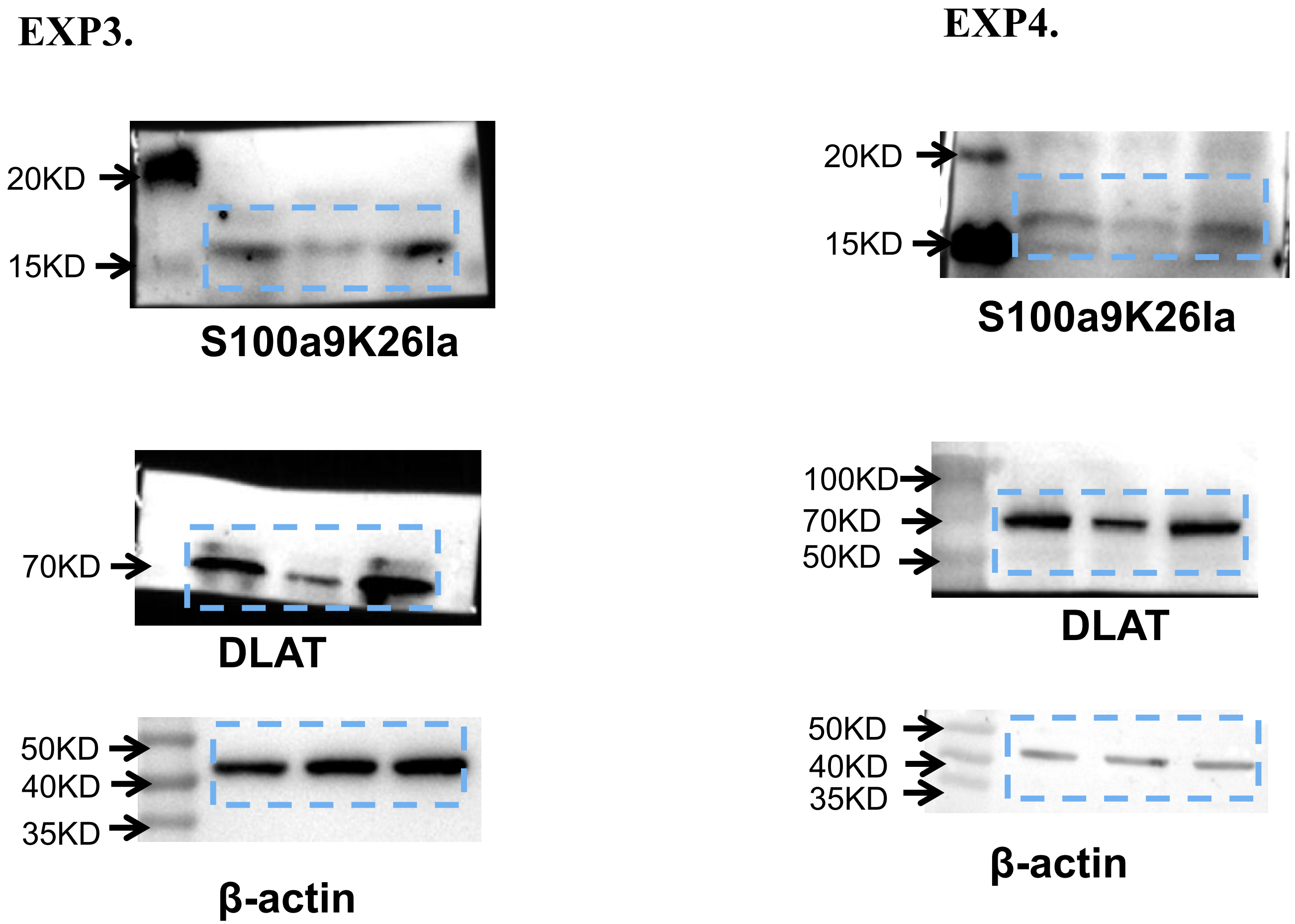

Full unedited gel for Figure F5I

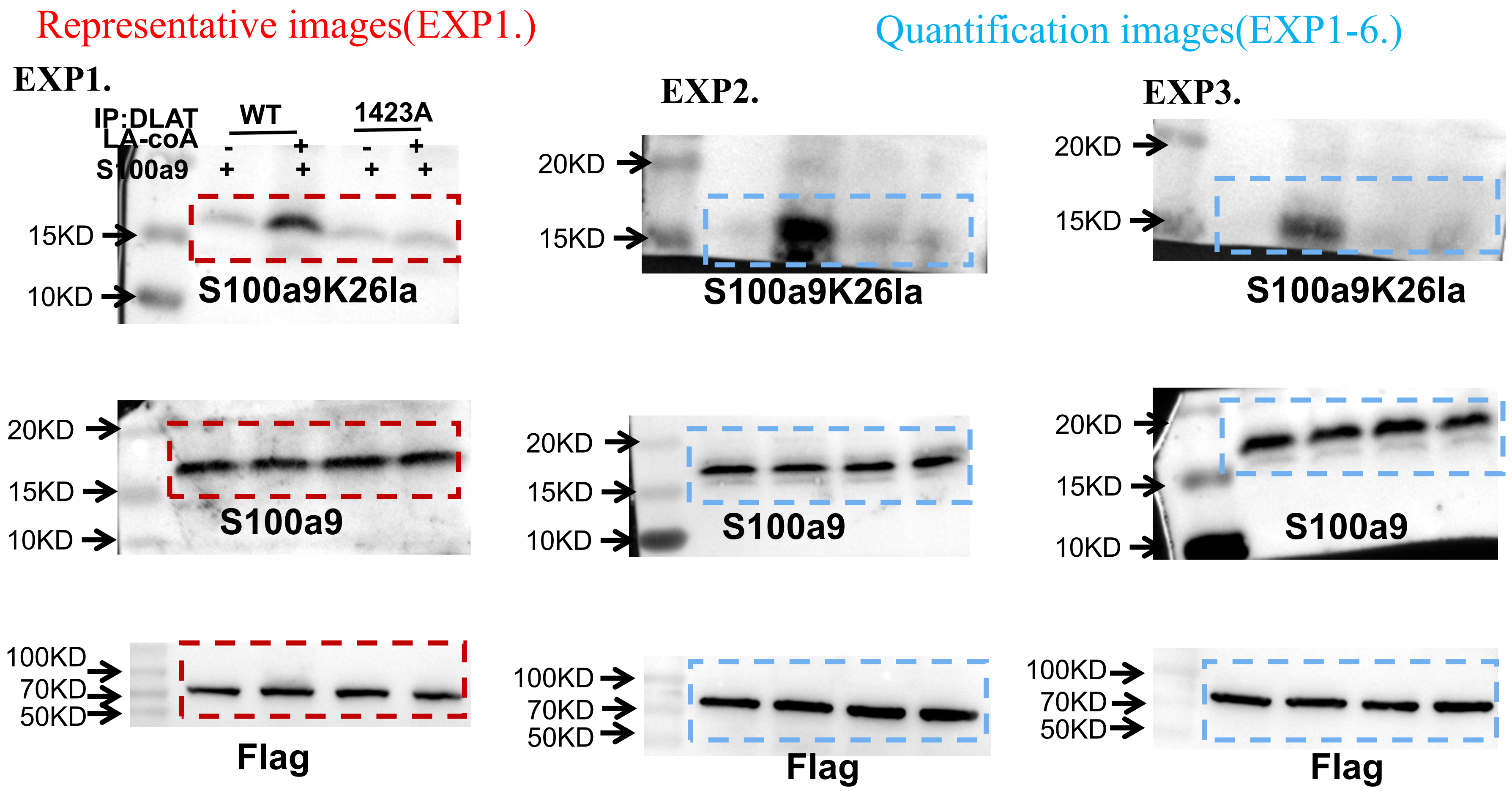

Full unedited gel for Figure F5I

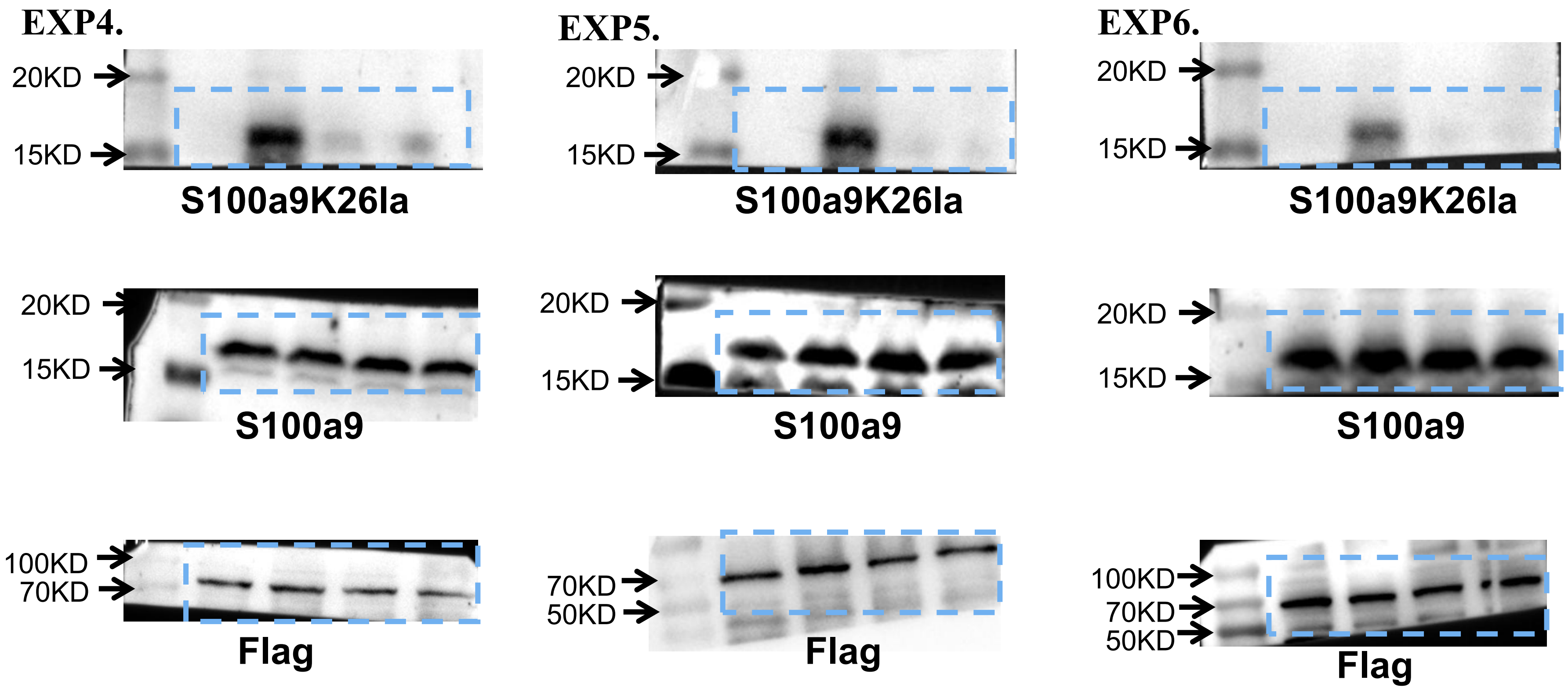

Full unedited gel for Figure F5J

Representative images(EXP1.)

Quantification images(EXP1-4.)

EXP1.

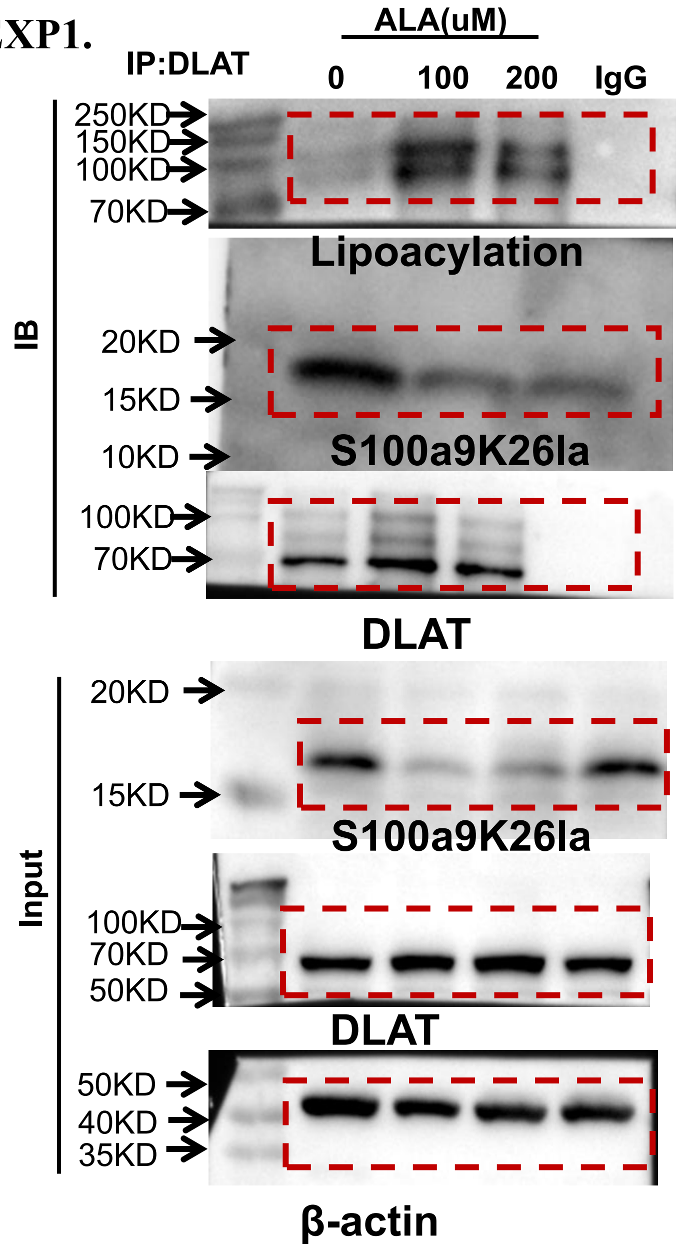

EXP2.

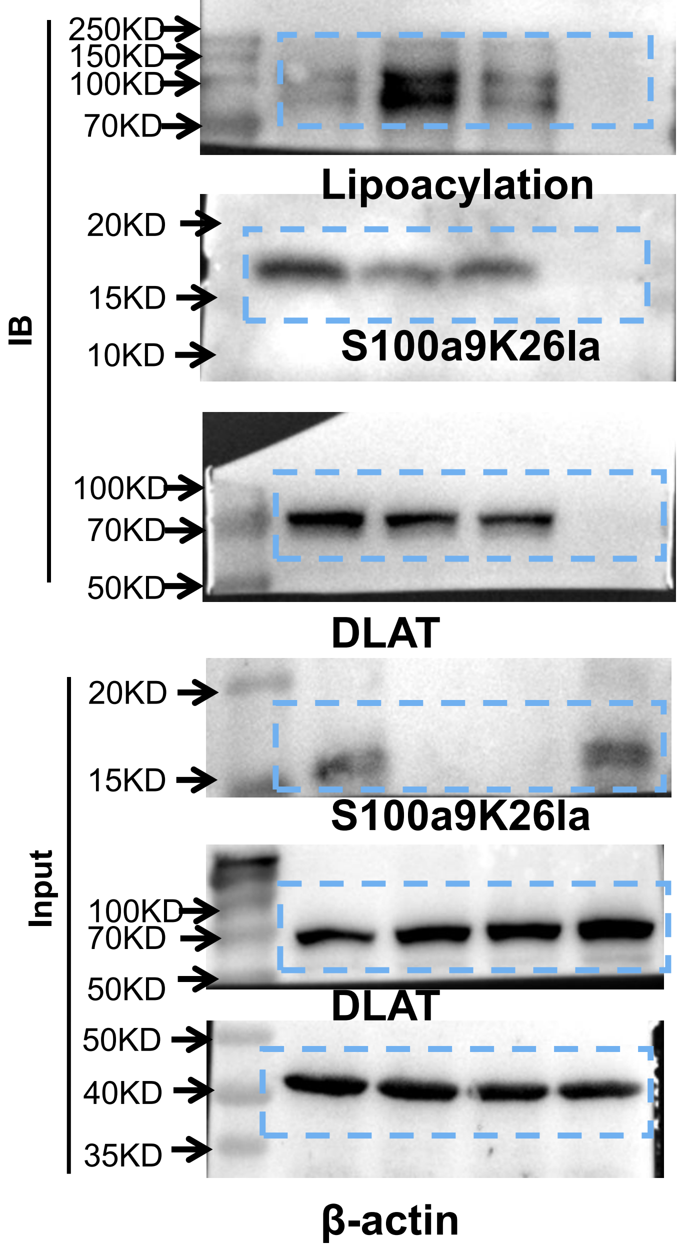

EXP3.

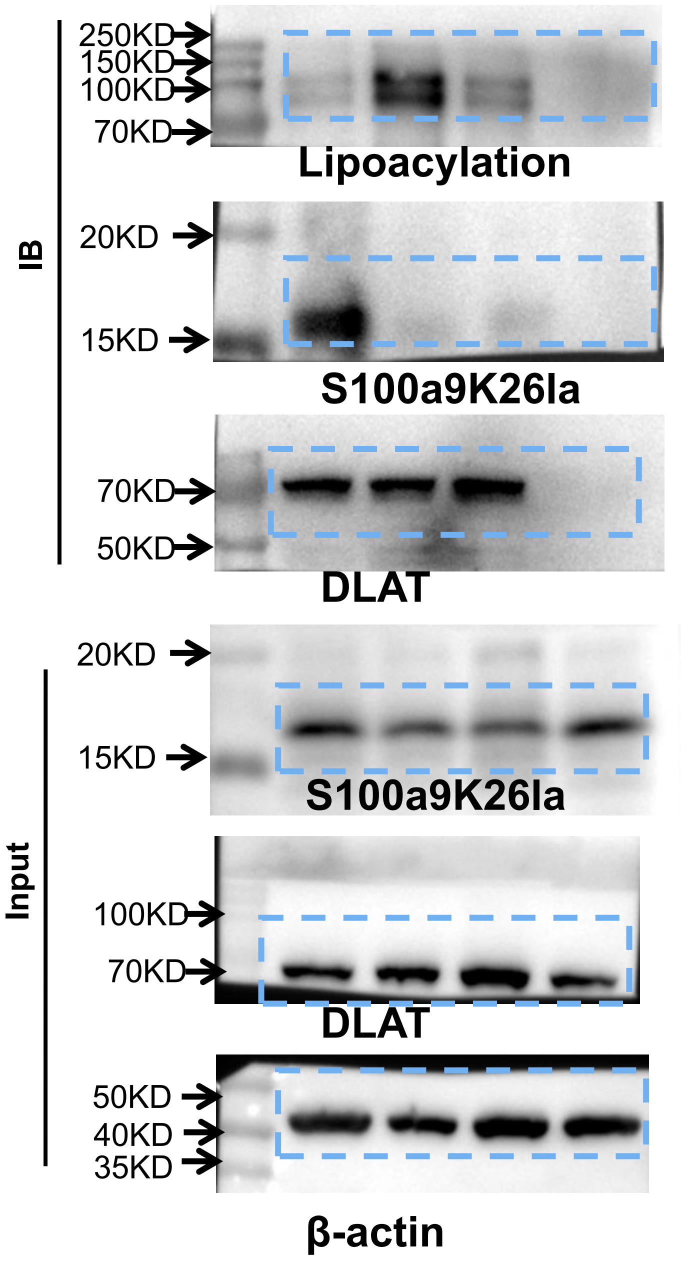
250KD
150KD
100KD
70KD
Lipoacylation
20KD
15KD
S100a9K26la
70KD
50KD
DLAT
IB
20KD
15KD
S100a9K26la
100KD
70KD
DLAT
50KD
40KD
35KD
β-actin
Input

EXP4.

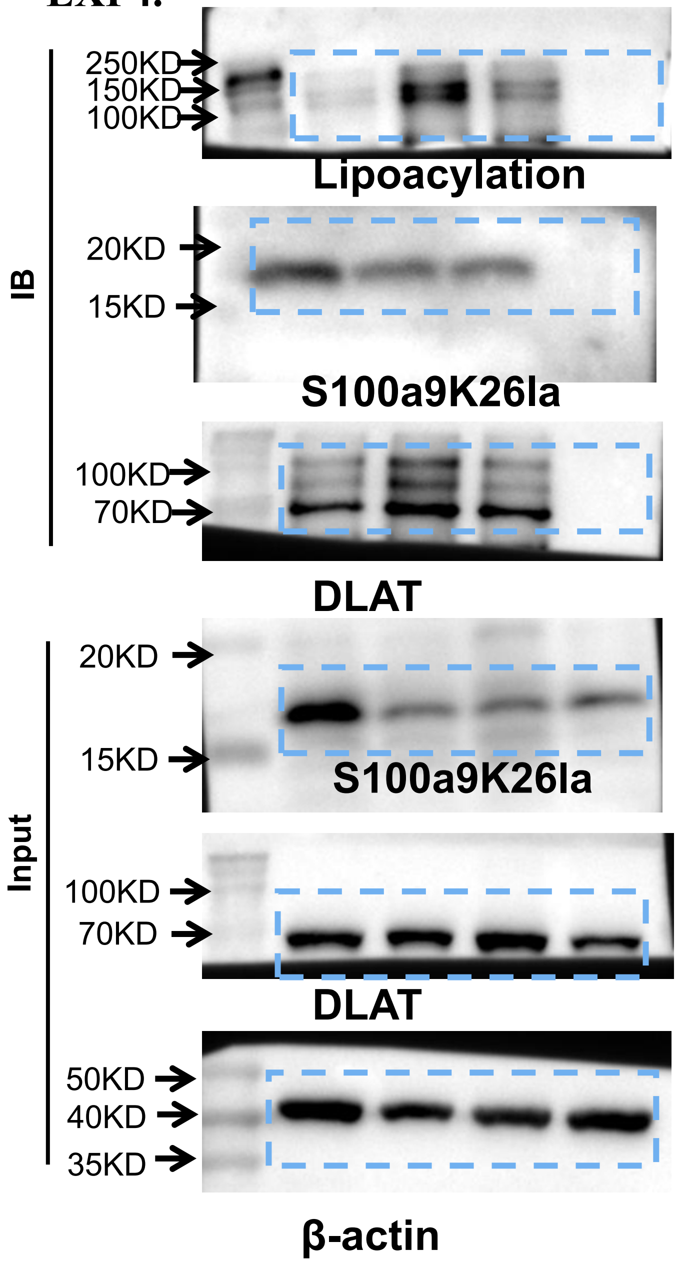
250KD
150KD
100KD
Lipoacylation
20KD
15KD
S100a9K26la
100KD
70KD
DLAT
IB
20KD
15KD
S100a9K26la
100KD
70KD
DLAT
50KD
40KD
35KD
β-actin
Input

Full unedited gel for Figure F5L

Representative images(EXP1.)

Quantification images(EXP1-6.)

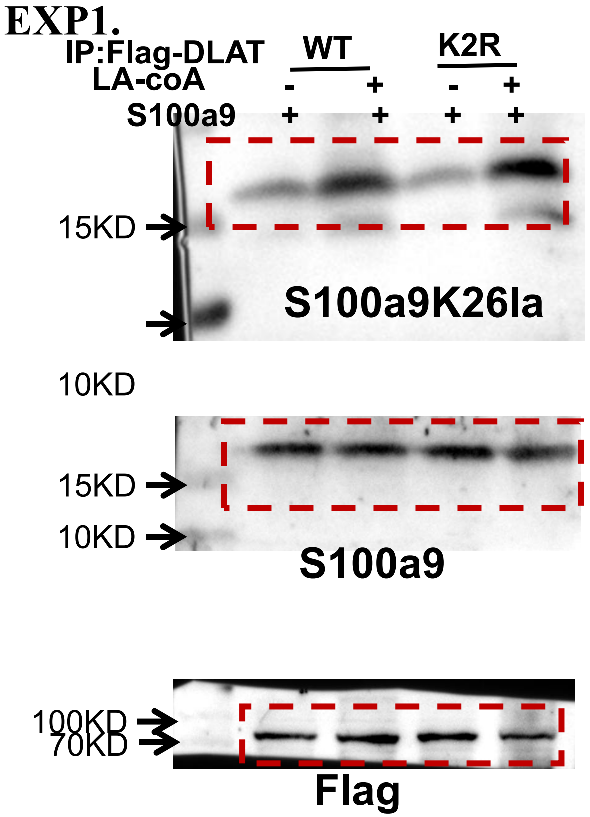

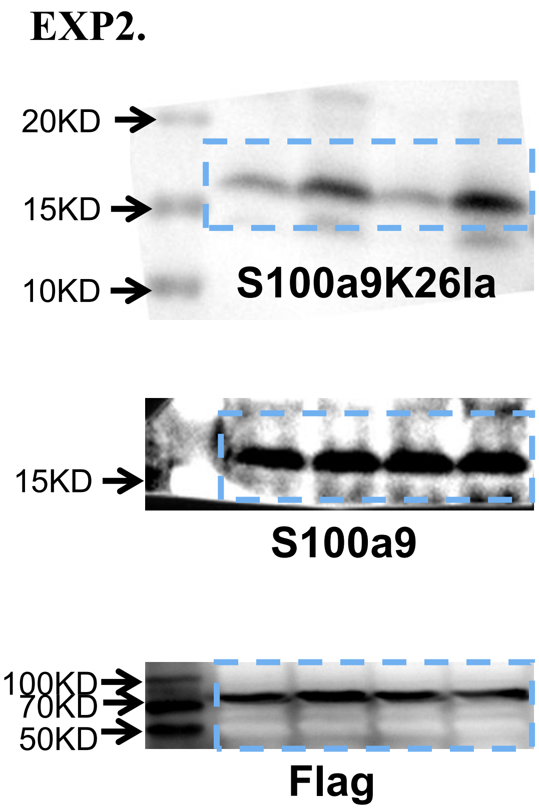

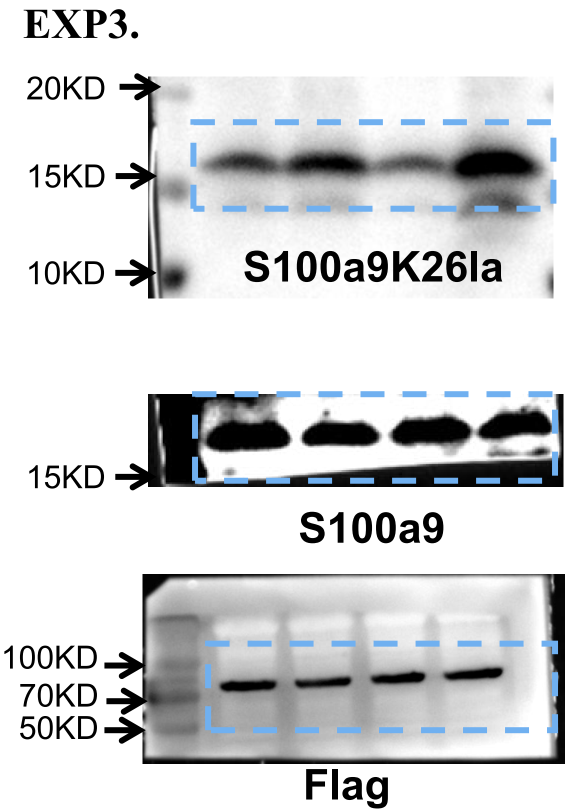

Full unedited gel for Figure F5L

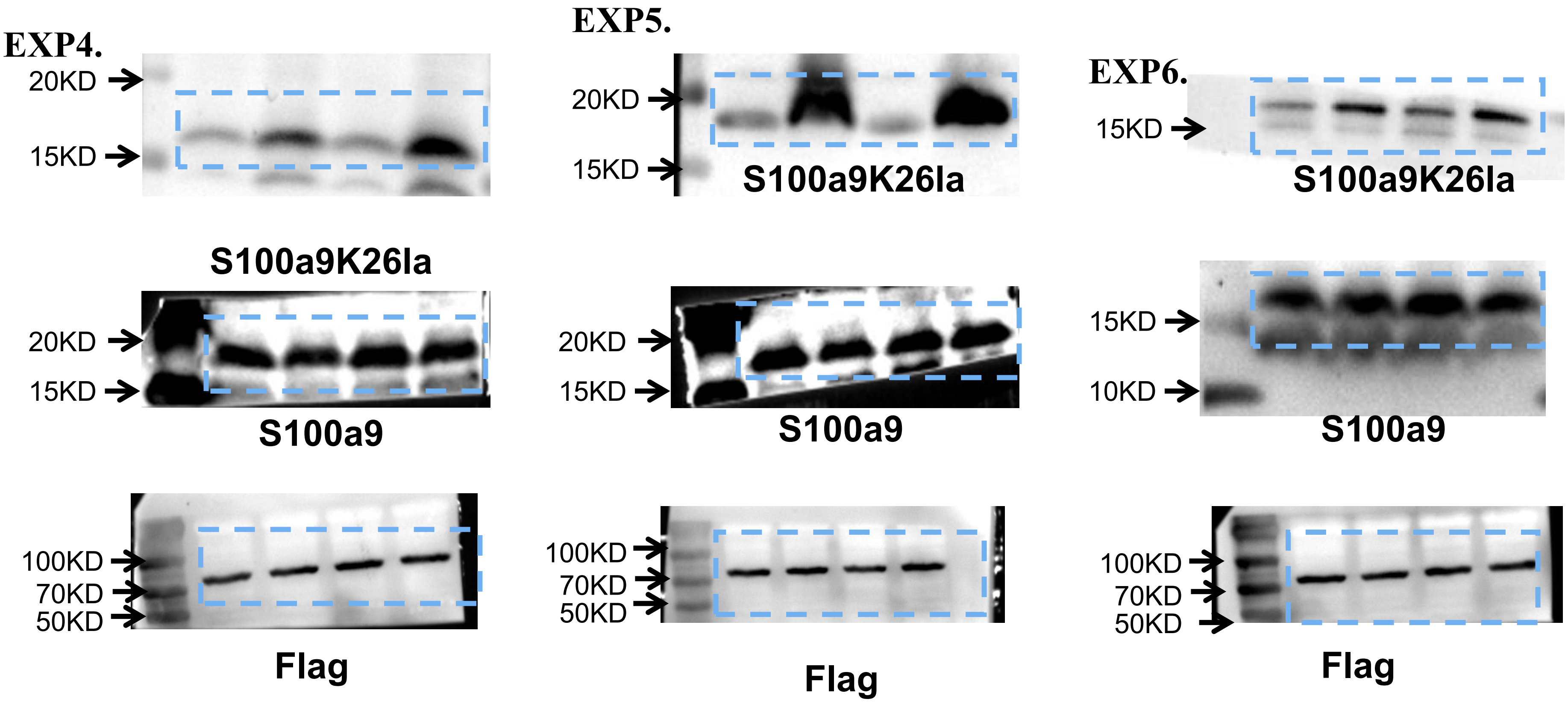

Full unedited gel for Figure F5M

Representative images(EXP1.)

Quantification images(EXP1-4.)

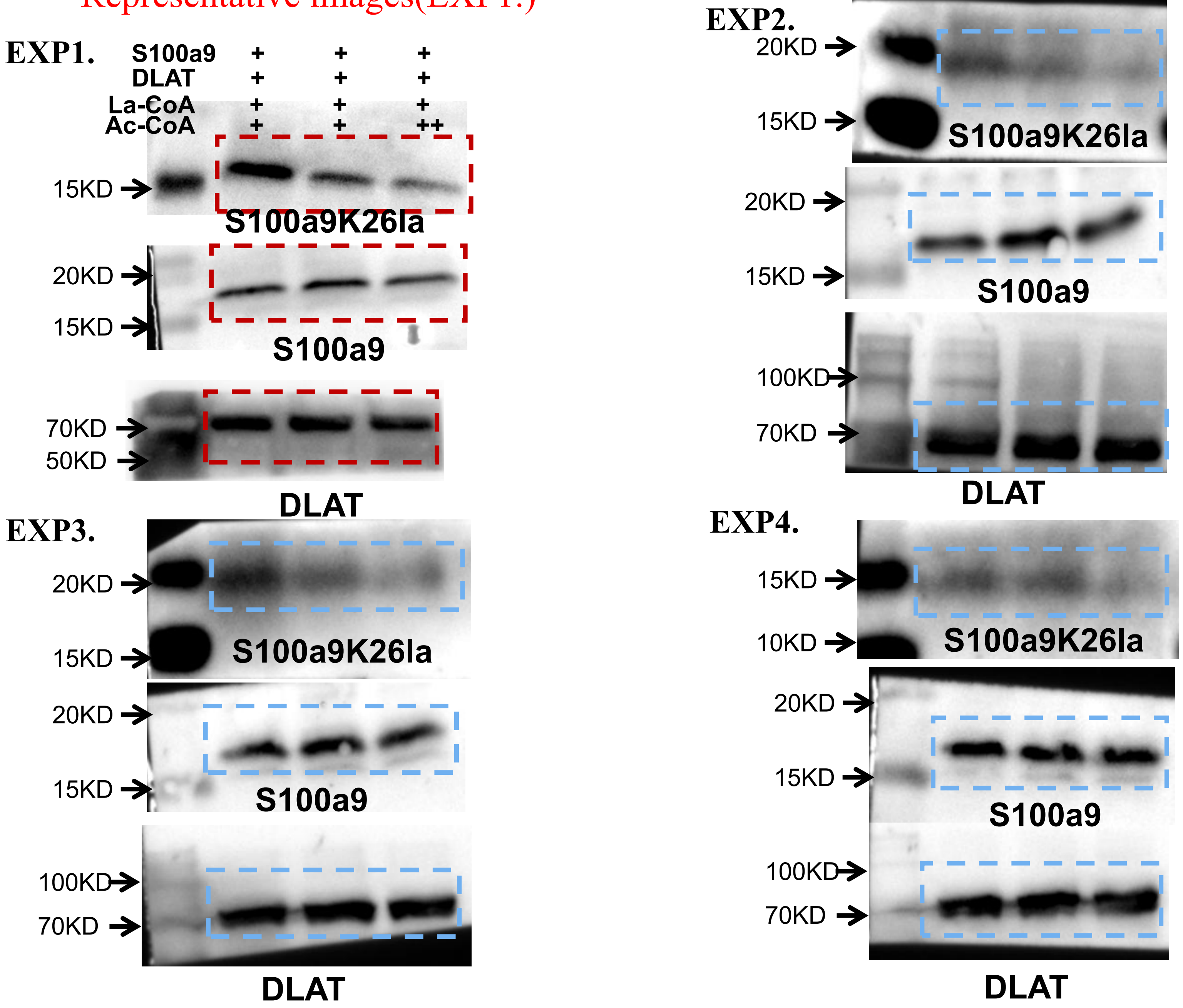

Full unedited gel for Figure F5P

Representative images(EXP1.)

Quantification images(EXP1-4.)

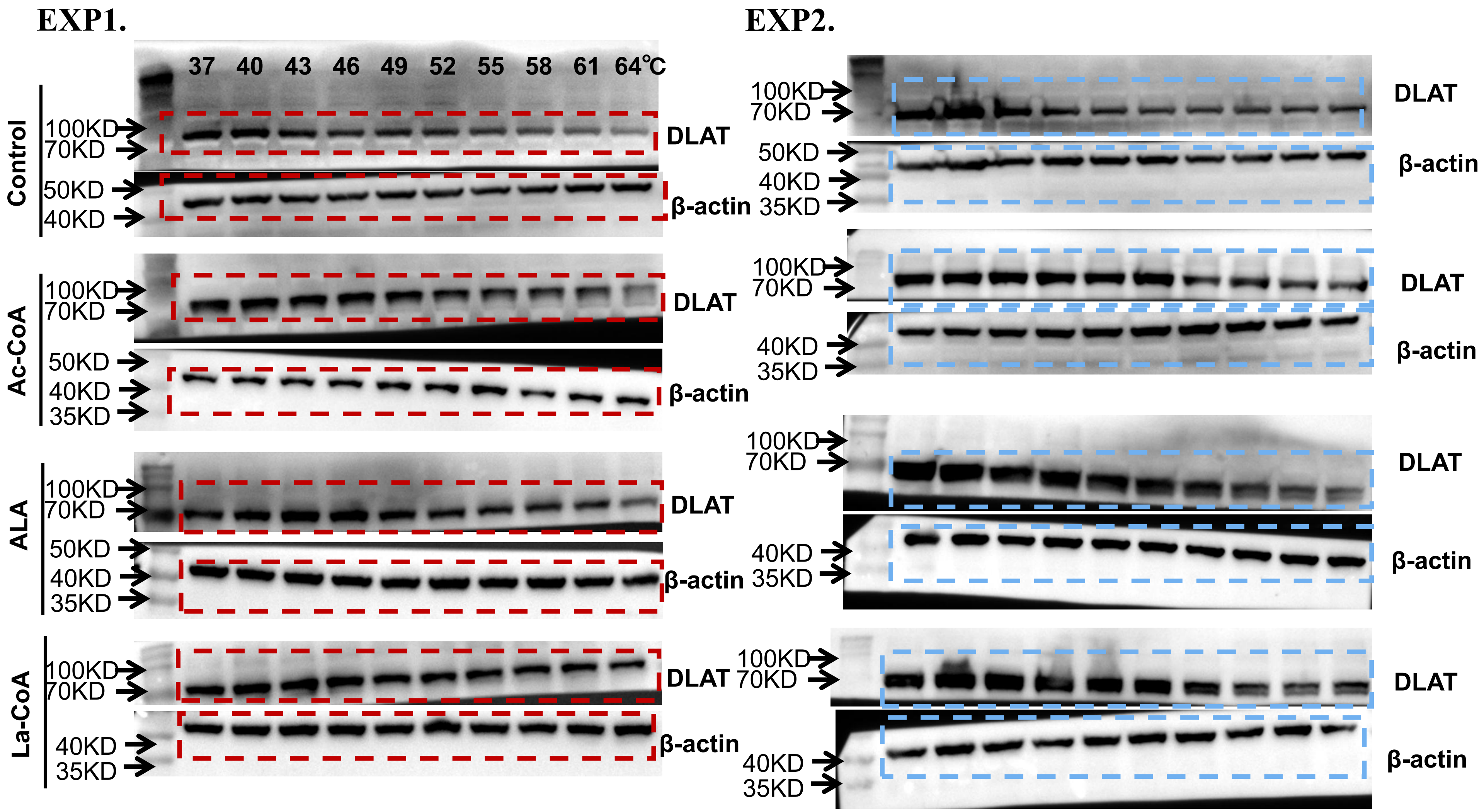

Full unedited gel for Figure F5P

EXP3.

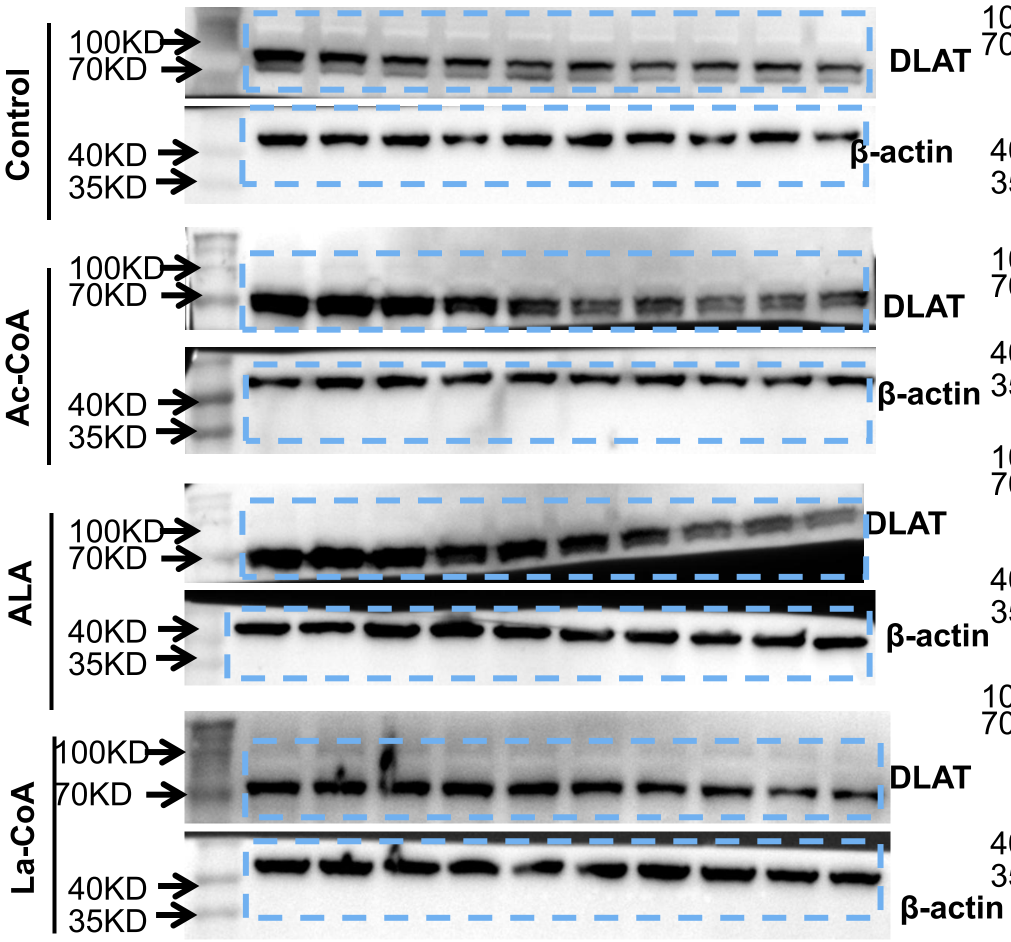

EXP4.

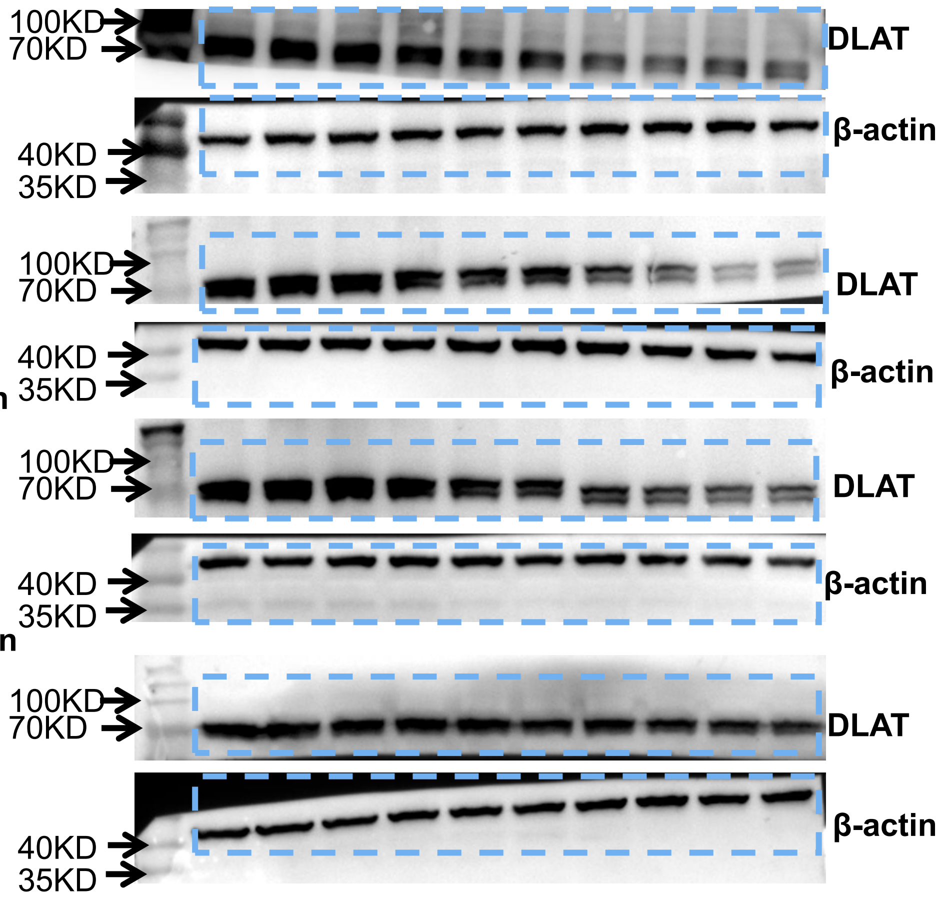

Full unedited gel for Figure 6B

Representative images(EXP1.) Quantification images(EXP1-6.)

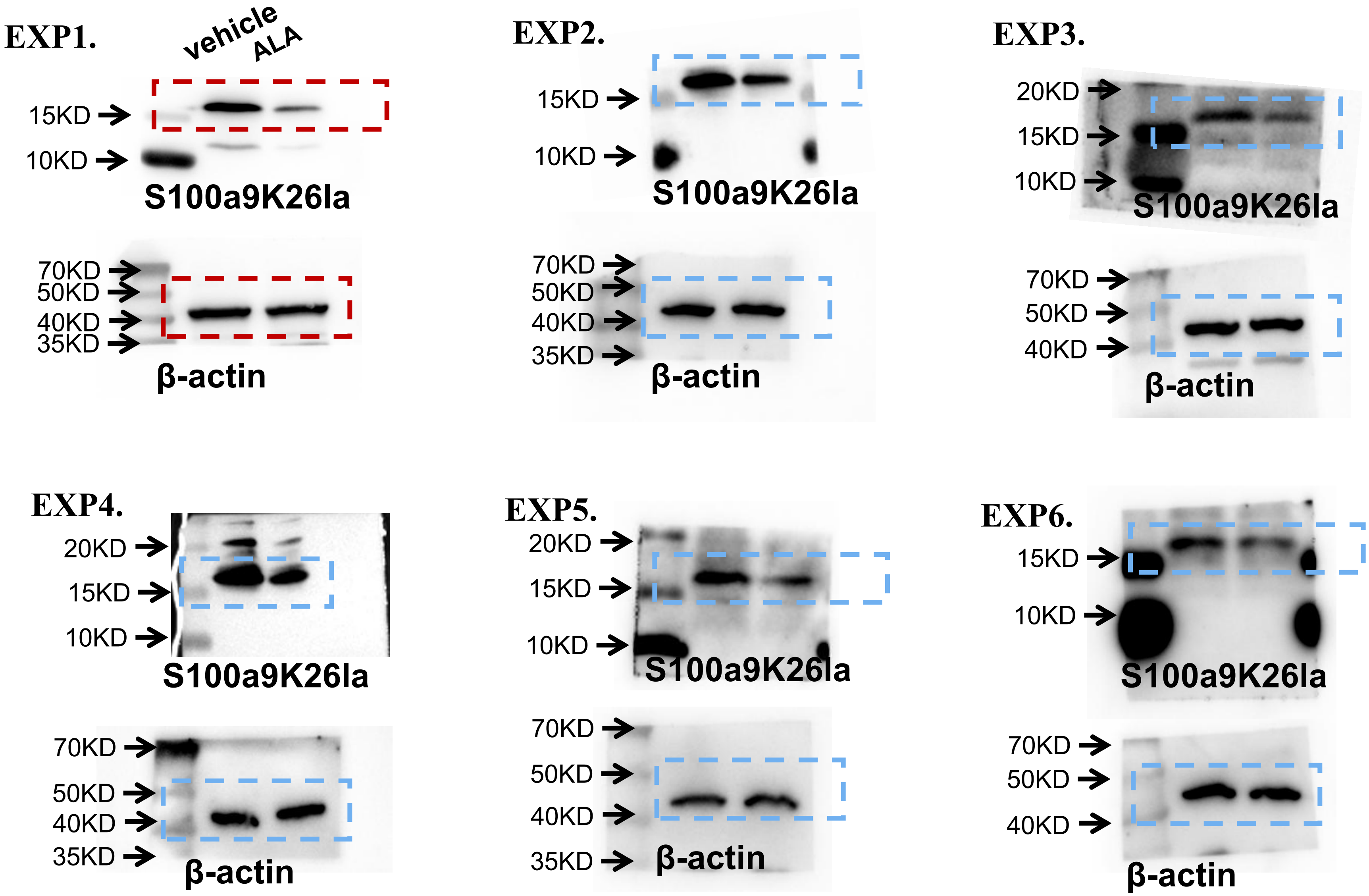

Supplementary Figures

Full unedited gel for Figure FS1A

Representative images(EXP1.)

Quantification images(EXP1-4.)

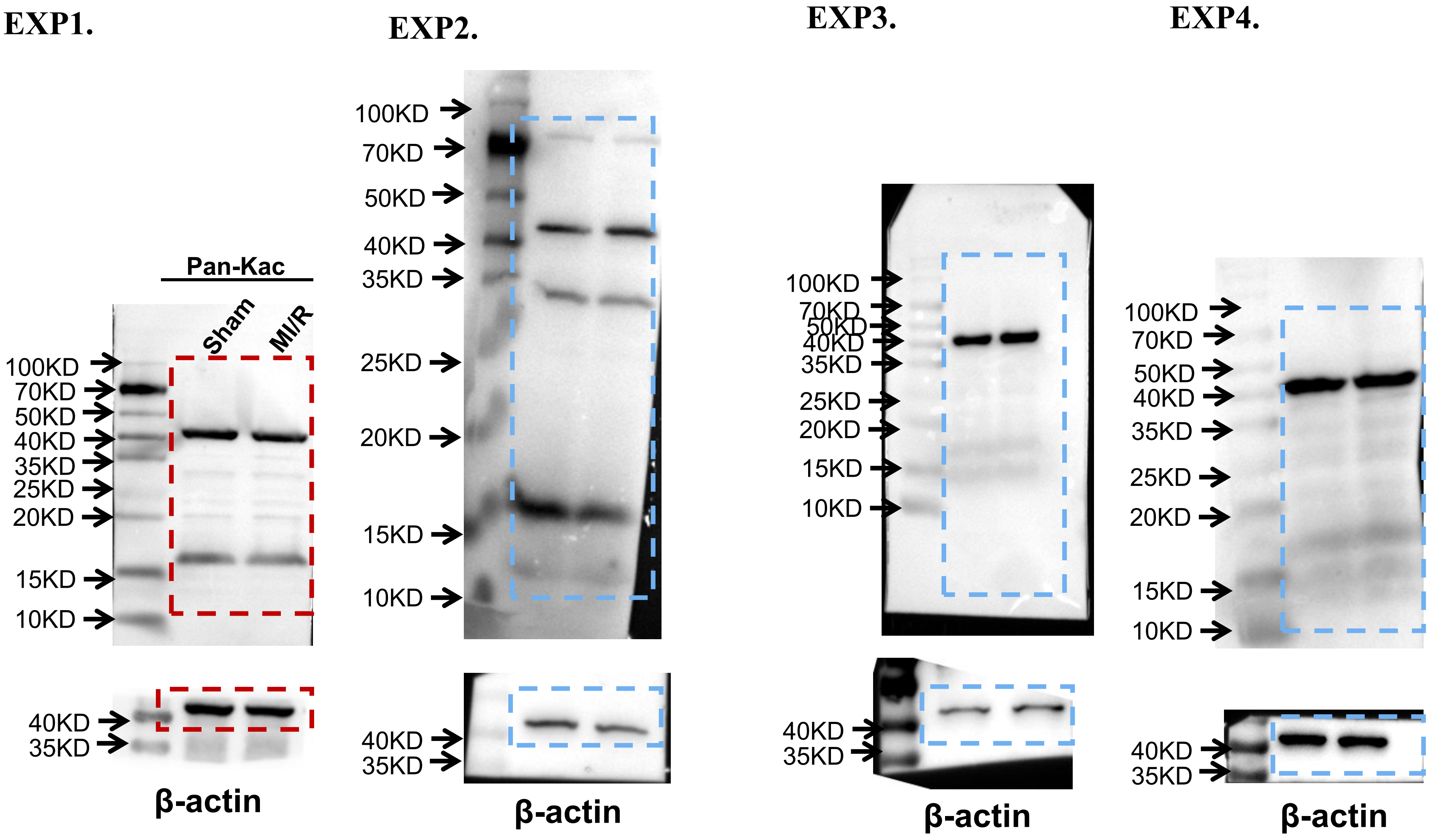

Full unedited gel for Figure FS1A

Representative images(EXP1.)

Quantification images(EXP1-4.)

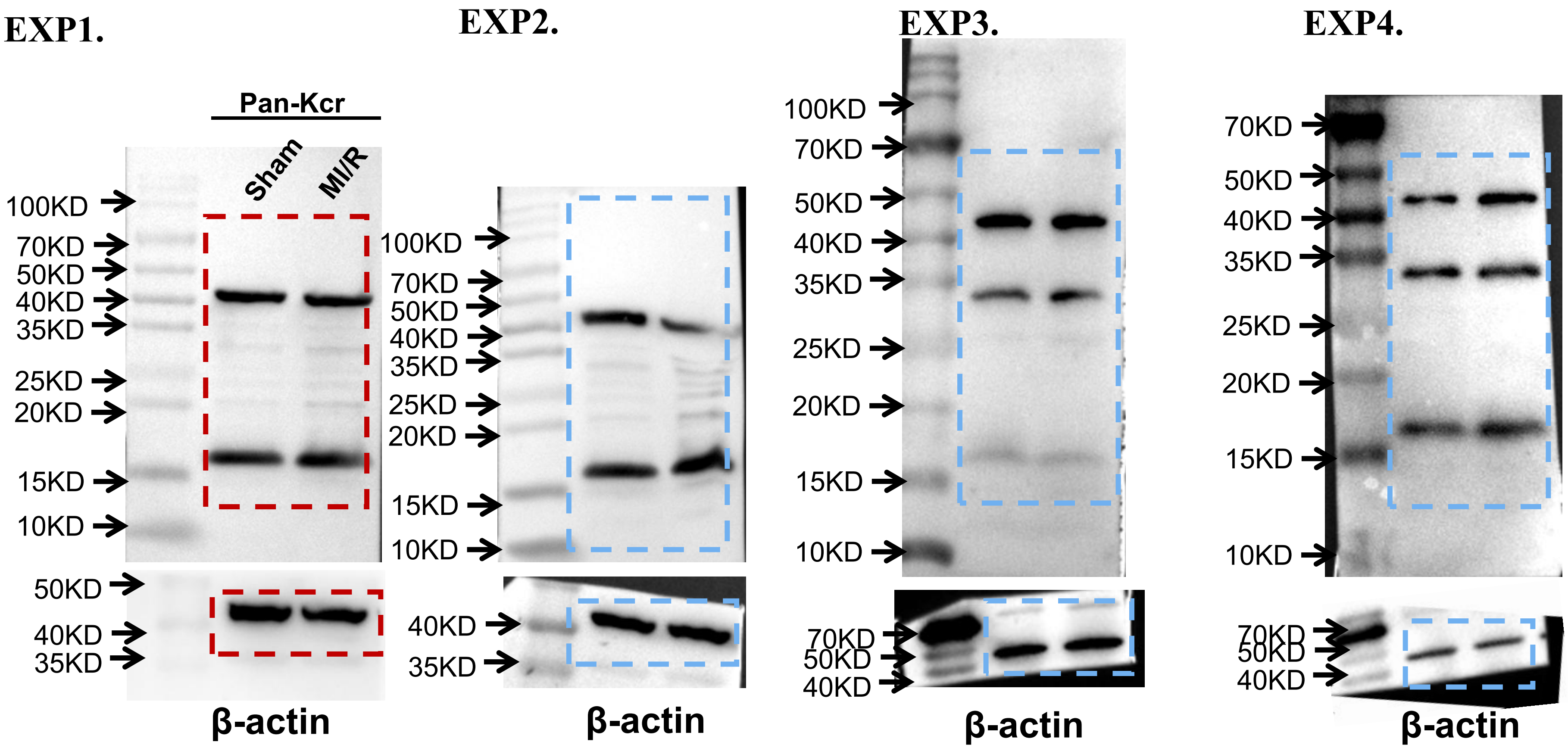

Full unedited gel for Figure FS1A

Representative images(EXP1.)

Quantification images(EXP1-4.)

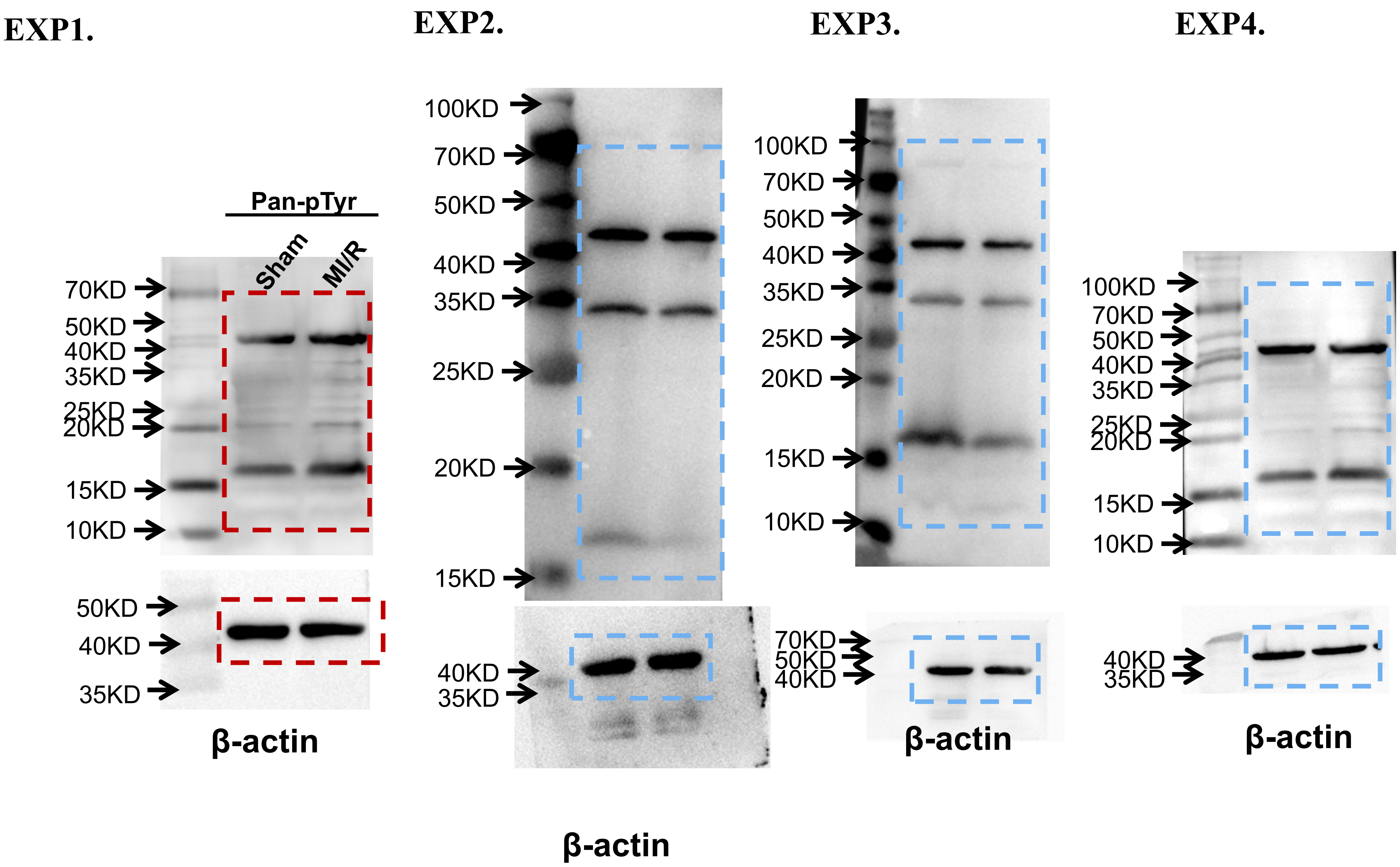

Full unedited gel for Figure FS1A

Representative images(EXP1.)

Quantification images(EXP1-4.)

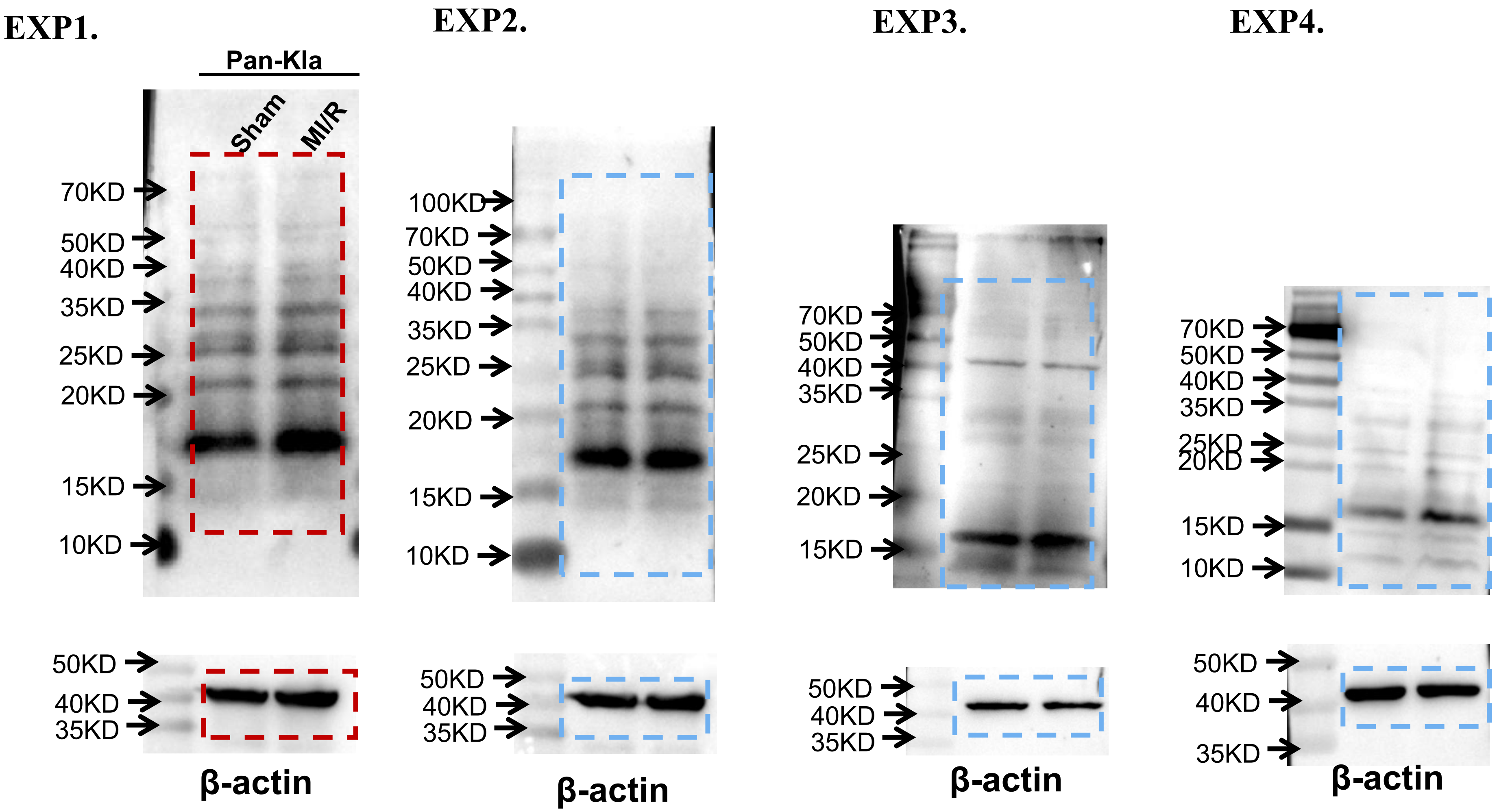

Full unedited gel for Figure FS1B

Representative images(EXP1.)

Quantification images(EXP1-4.)

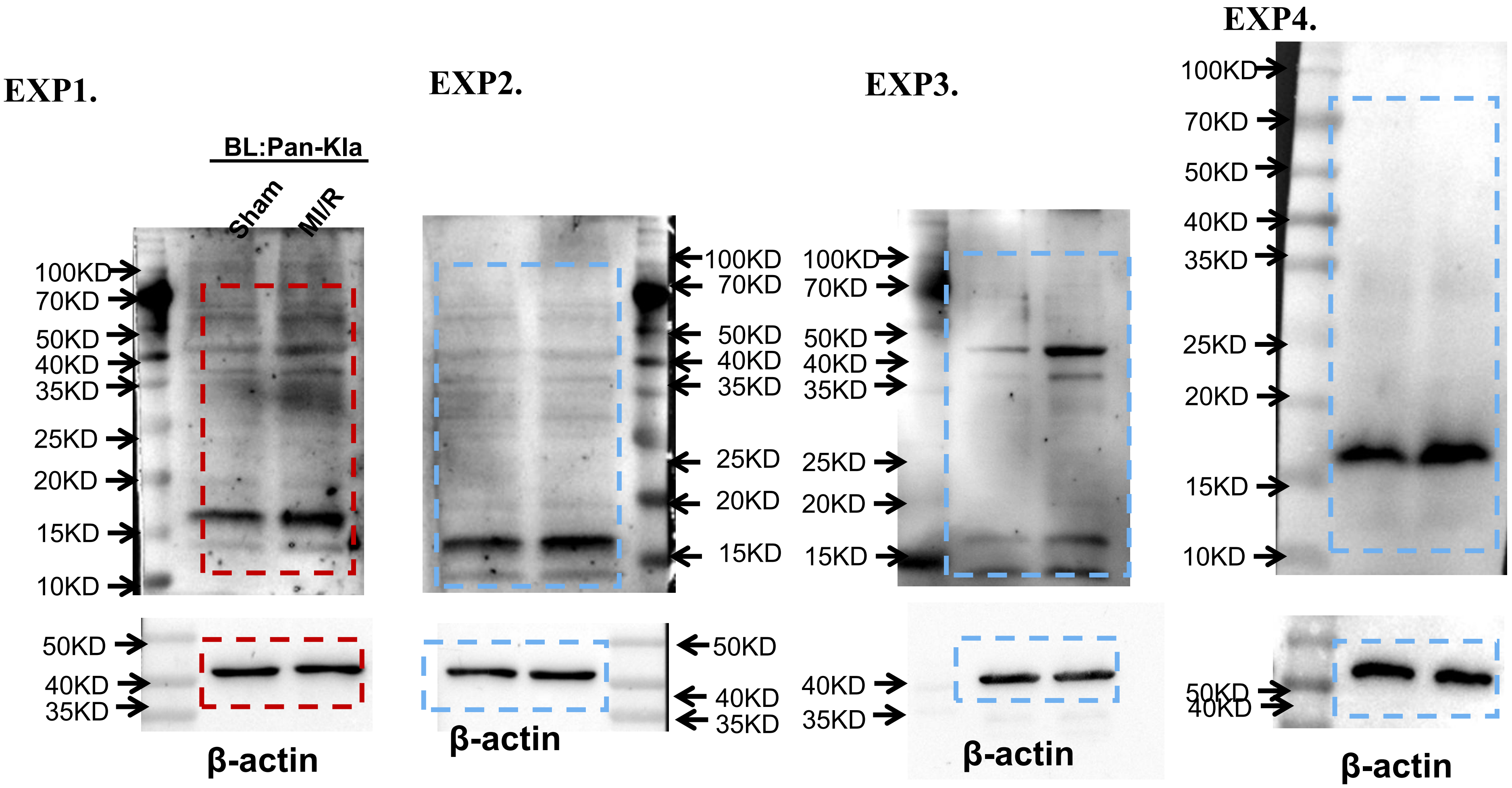

Full unedited gel for Figure FS1J

Representative images(EXP1.)

Quantification images(EXP1-4.)

EXP1.

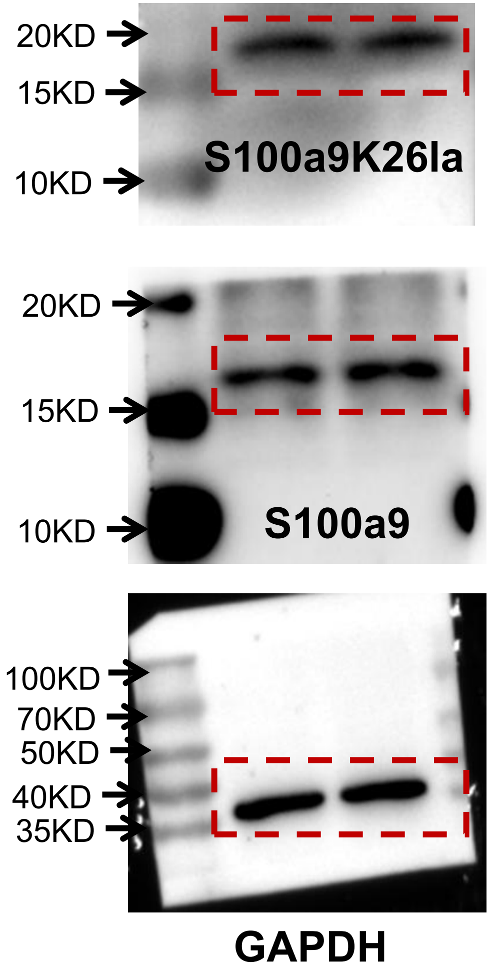

EXP2.

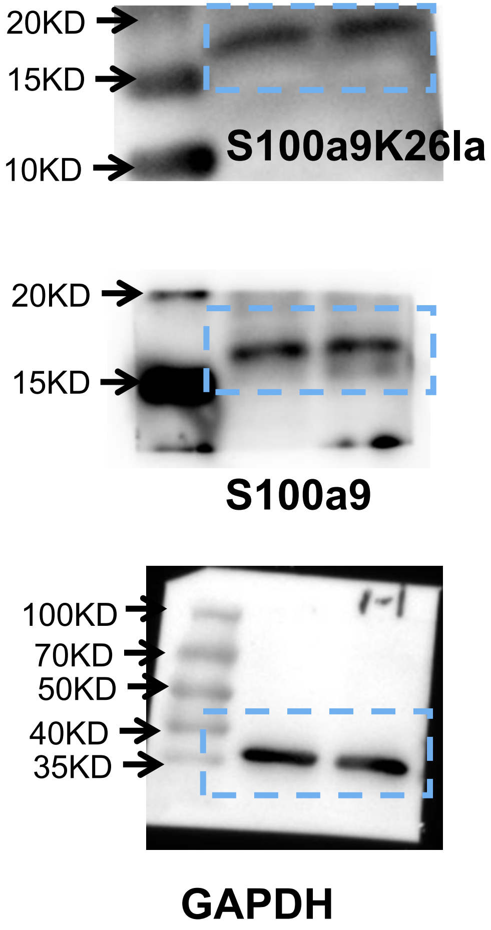

EXP3.

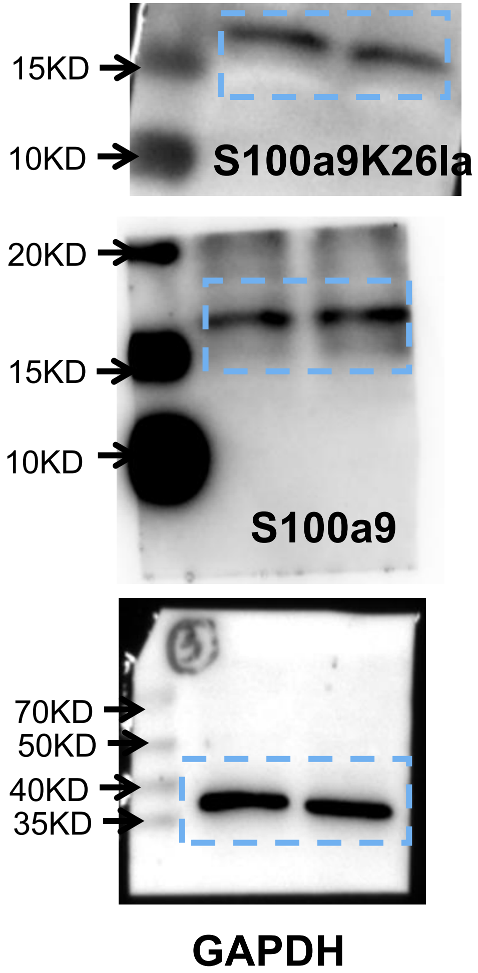
15KD
10KD
S100a9K26la
20KD
15KD
10KD
S100a9
70KD
50KD
40KD
35KD
GAPDH

EXP4.

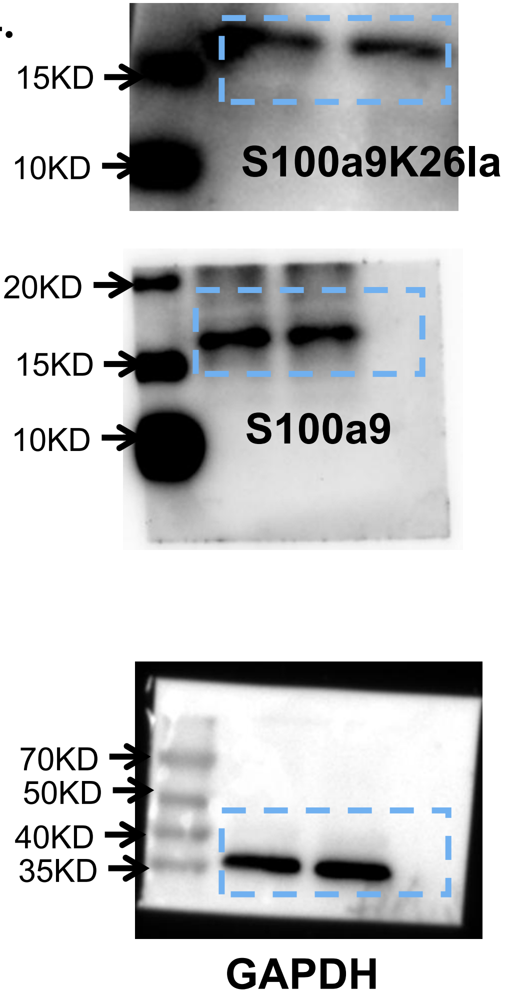
15KD
10KD
S100a9K26la
20KD
15KD
10KD
S100a9
70KD
50KD
40KD
35KD
GAPDH

Full unedited gel for Figure S1K

Quantification images(EXP1-4.)

EXP1.

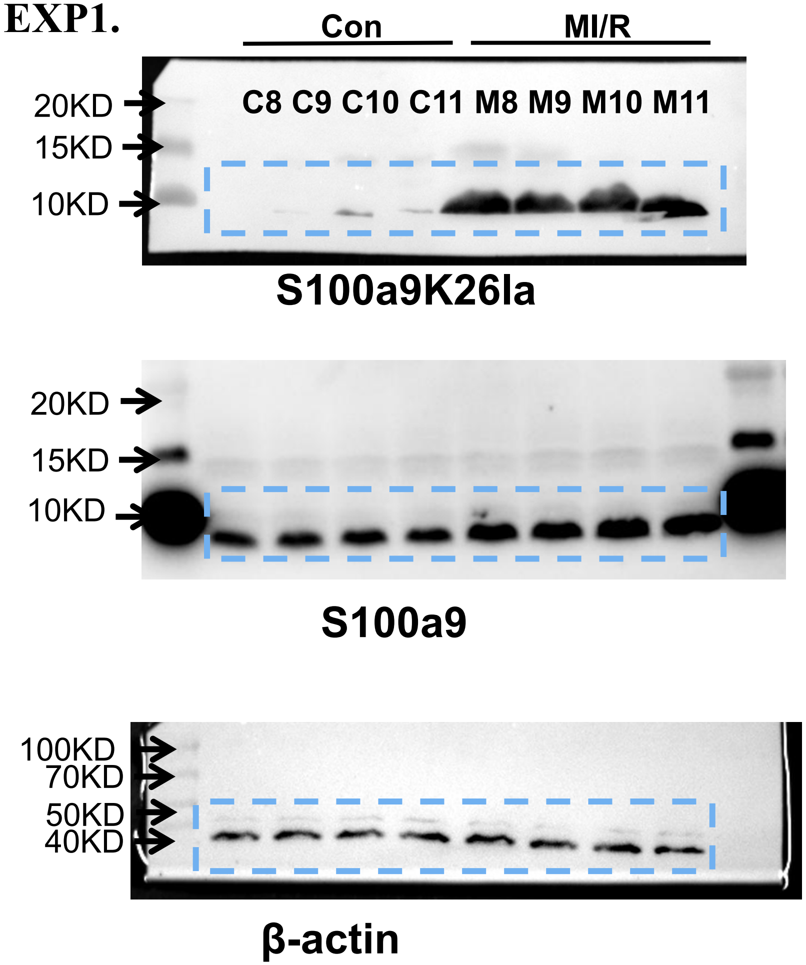

EXP2.

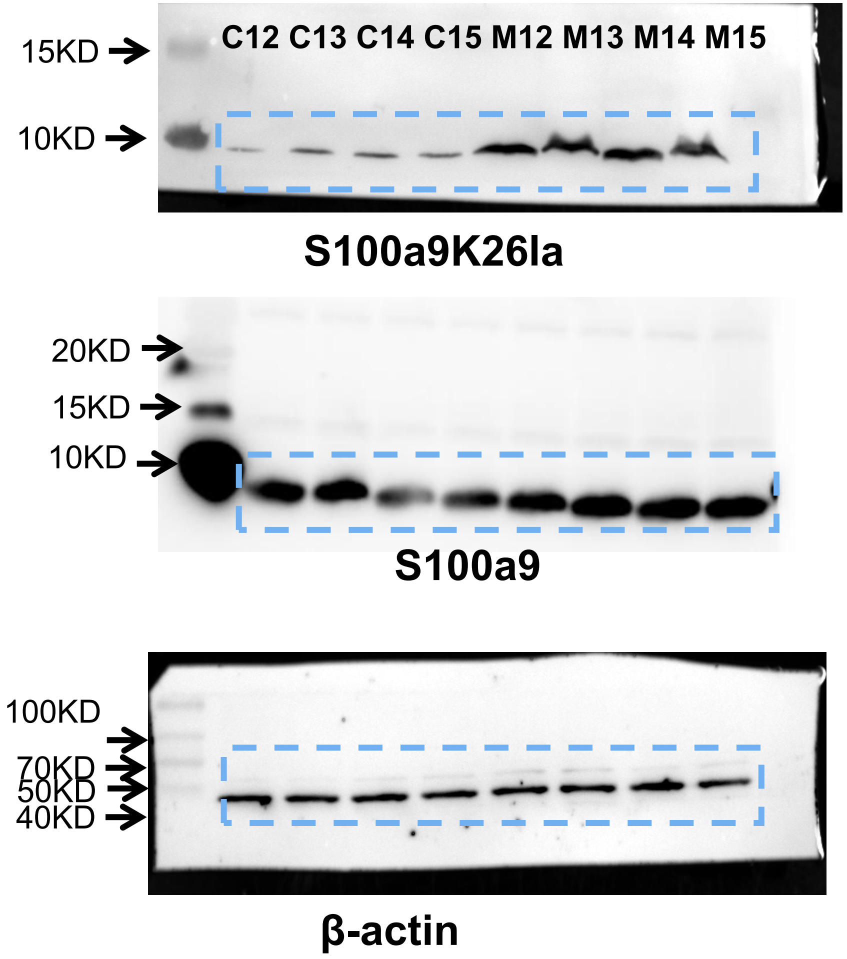

Full unedited gel for Figure S1K

Quantification images(EXP1-4.)

EXP3.

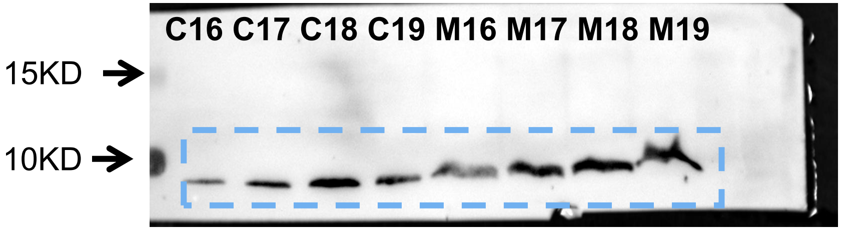

S100a9K26la

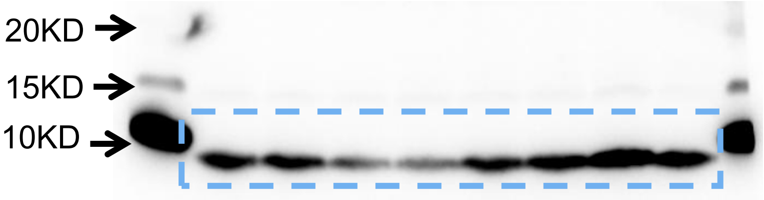

S100a9

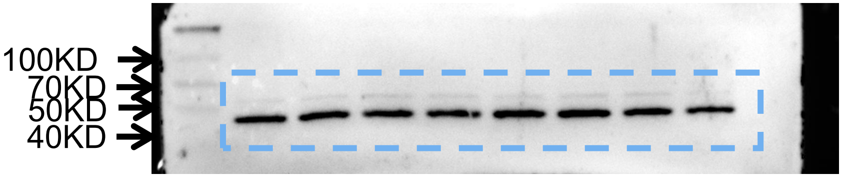

β-actin

EXP4.

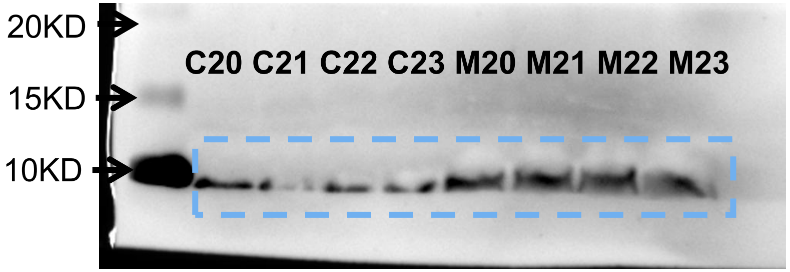

S100a9K26la

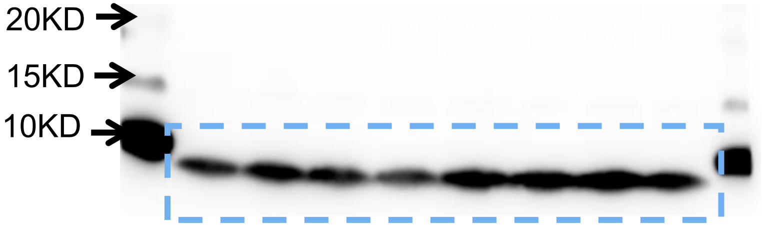

S100a9

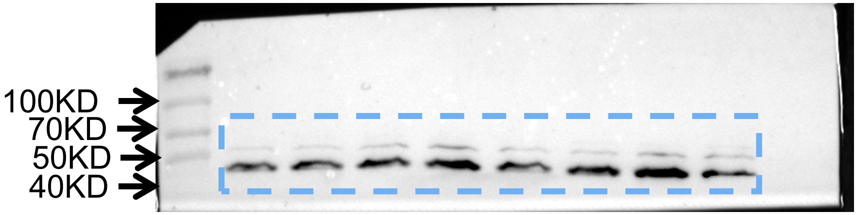

β-actin

Full unedited gel for Figure S2E

Representative images(EXP1.)

Quantification images(EXP1-4.)

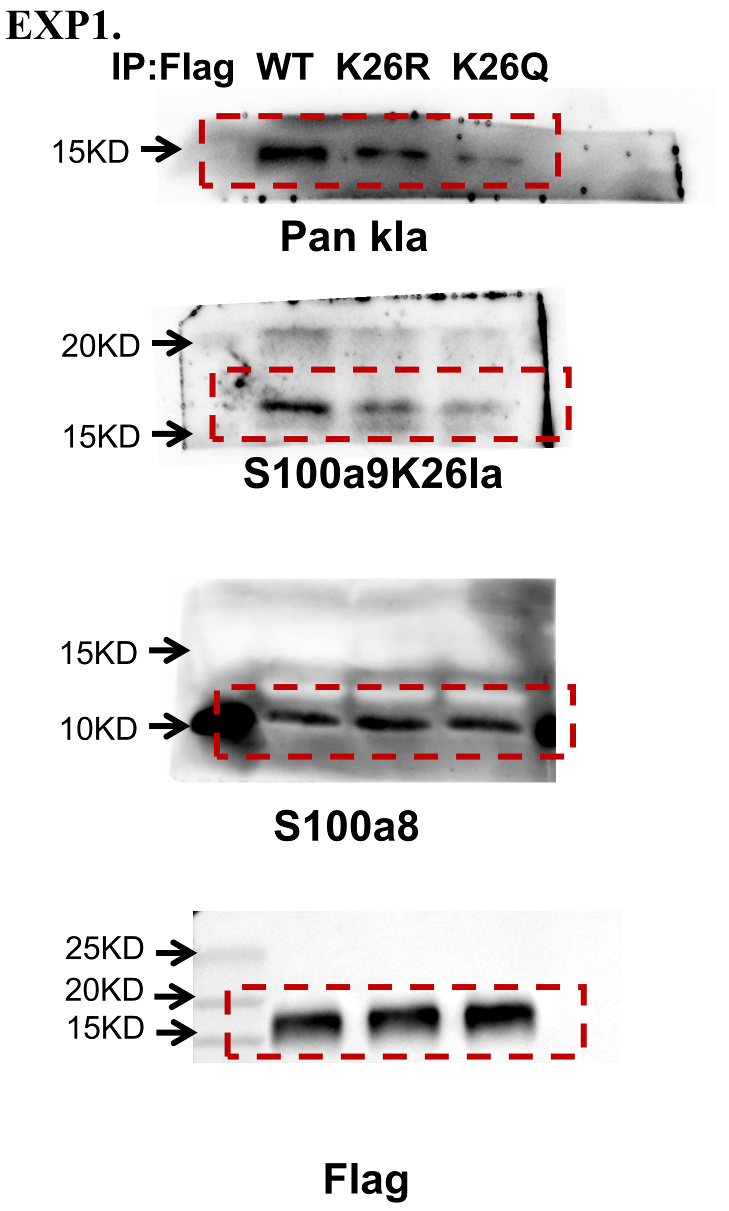

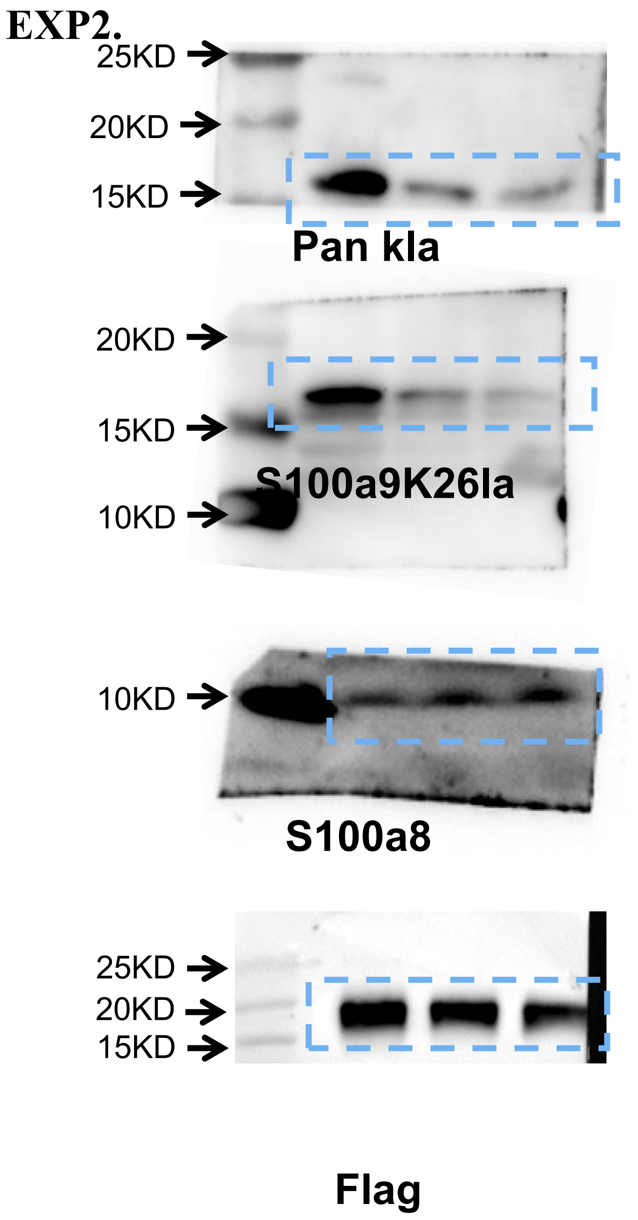

Full unedited gel for Figure S2E

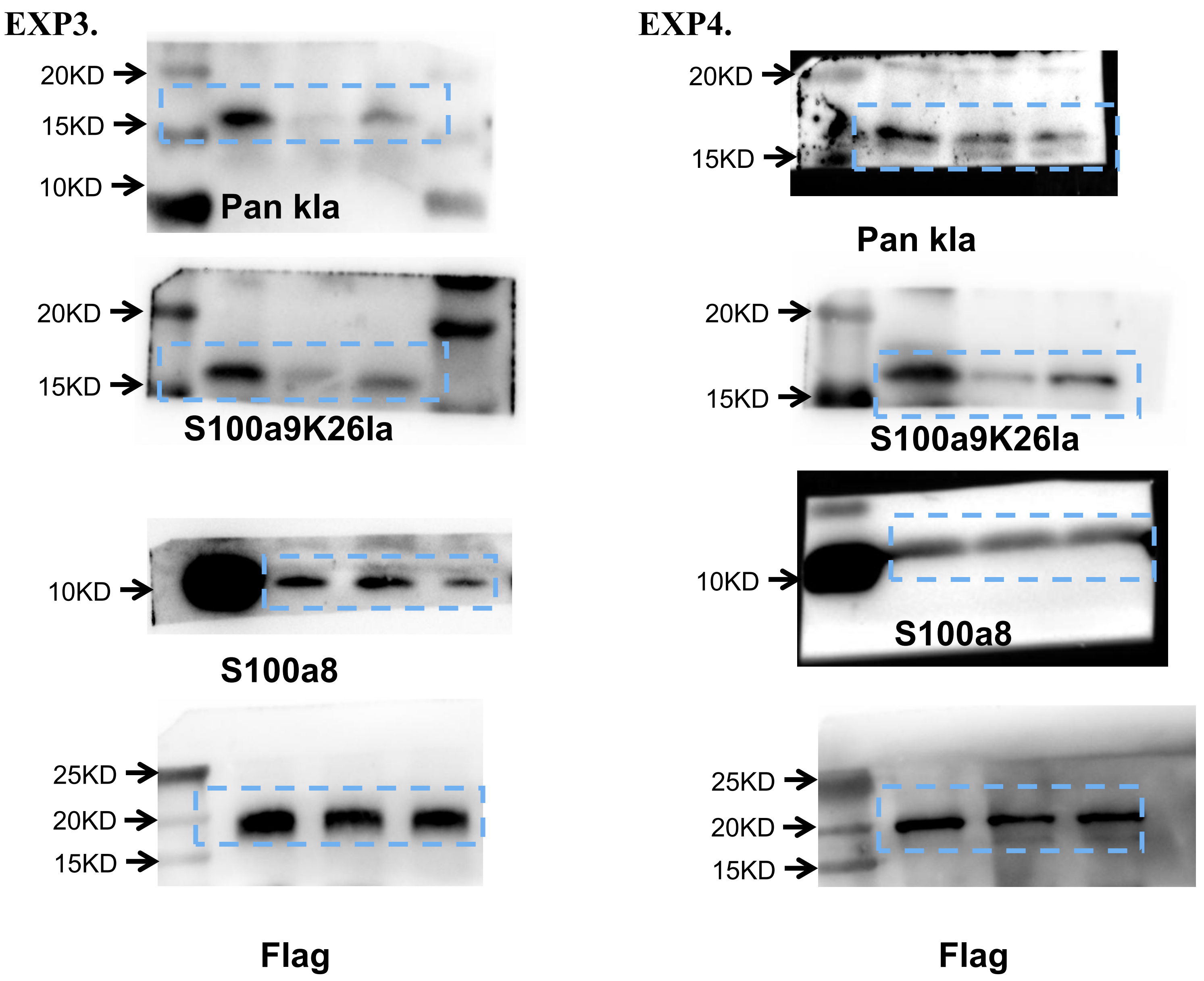

Full unedited gel for Figure S9D

Representative images(EXP1.)   Quantification images(EXP1-4.)

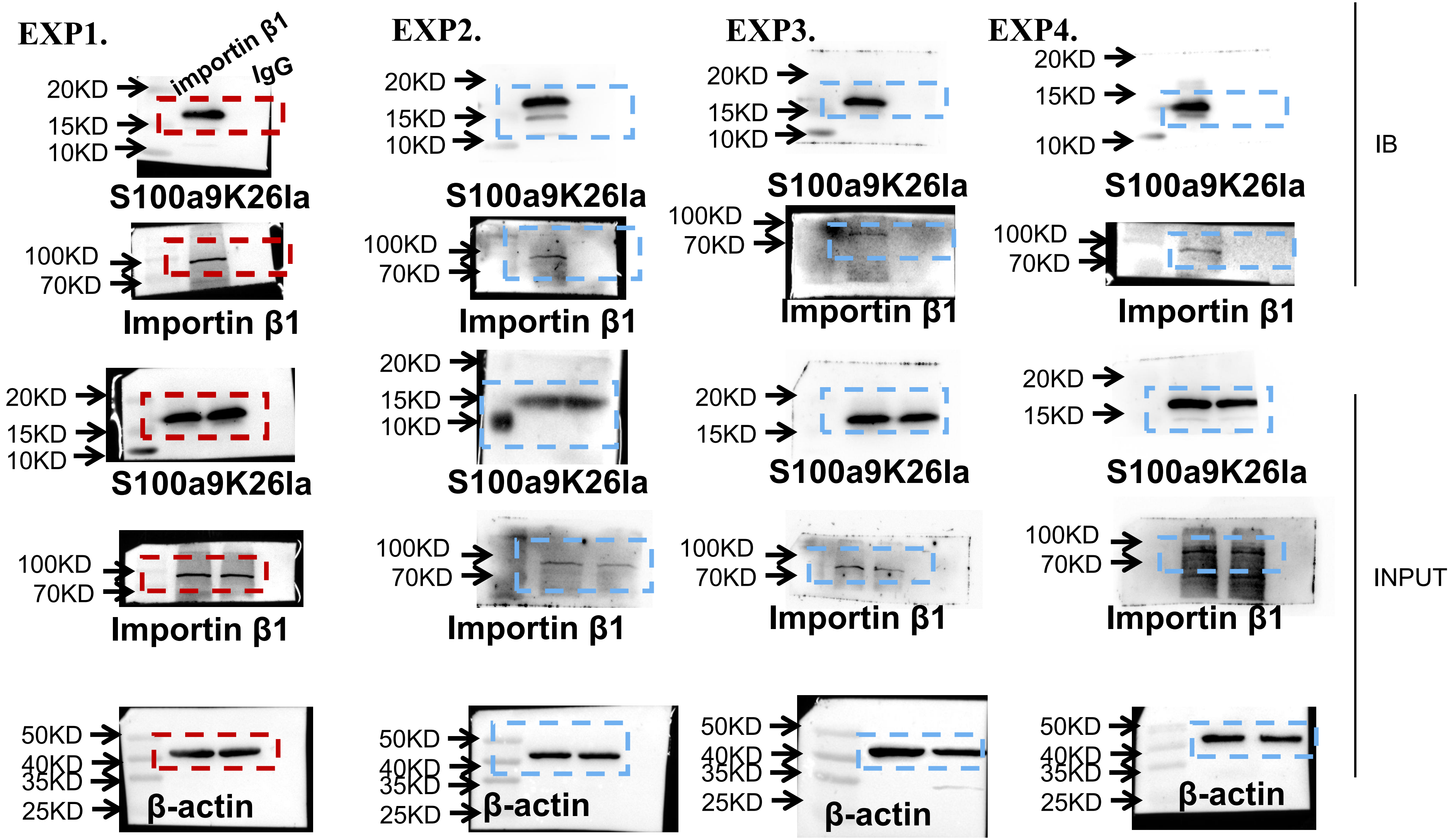

Full unedited gel for Figure S9E

Representative images(EXP1.) Quantification images(EXP1-6.)

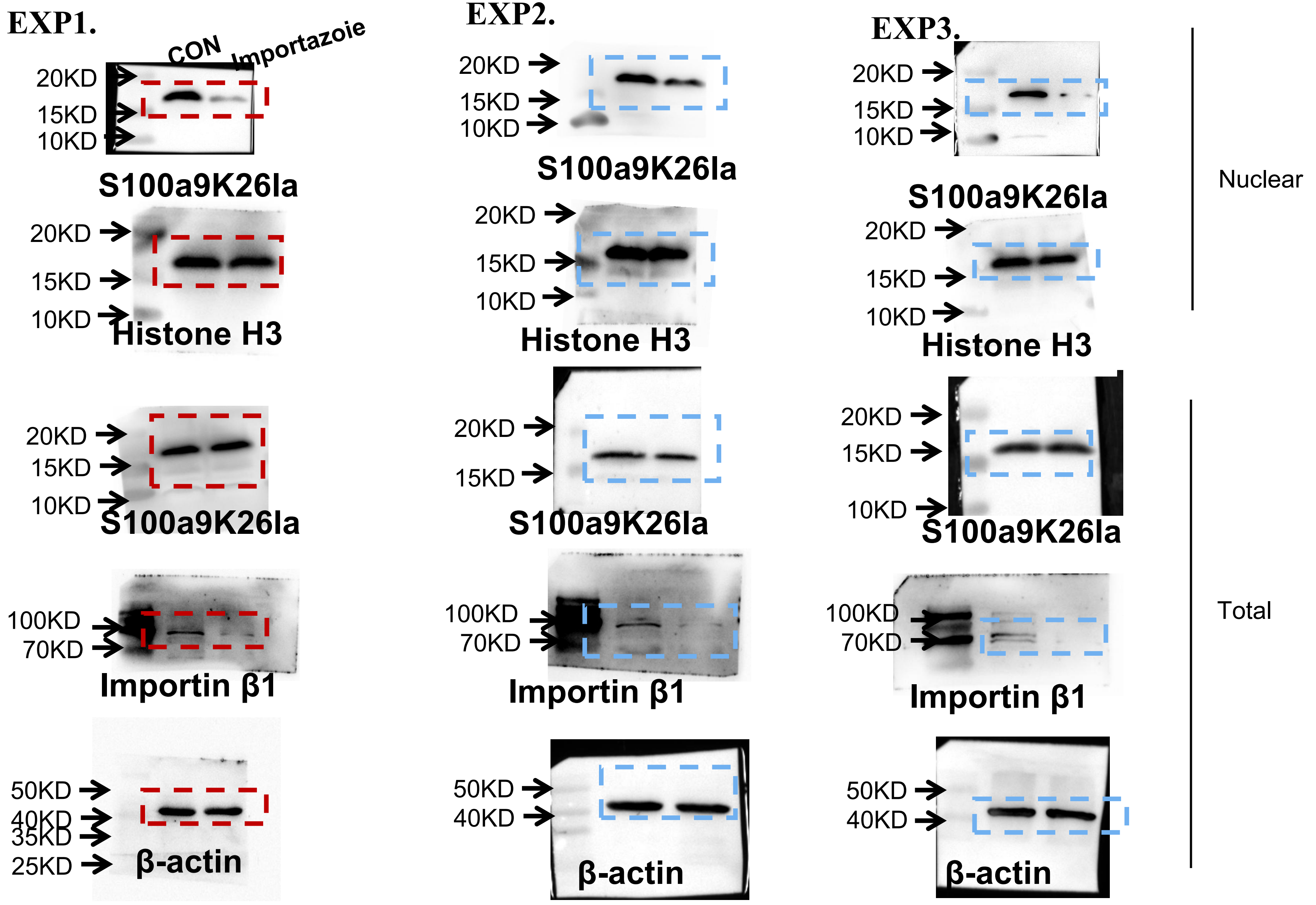

Full unedited gel for Figure S9D

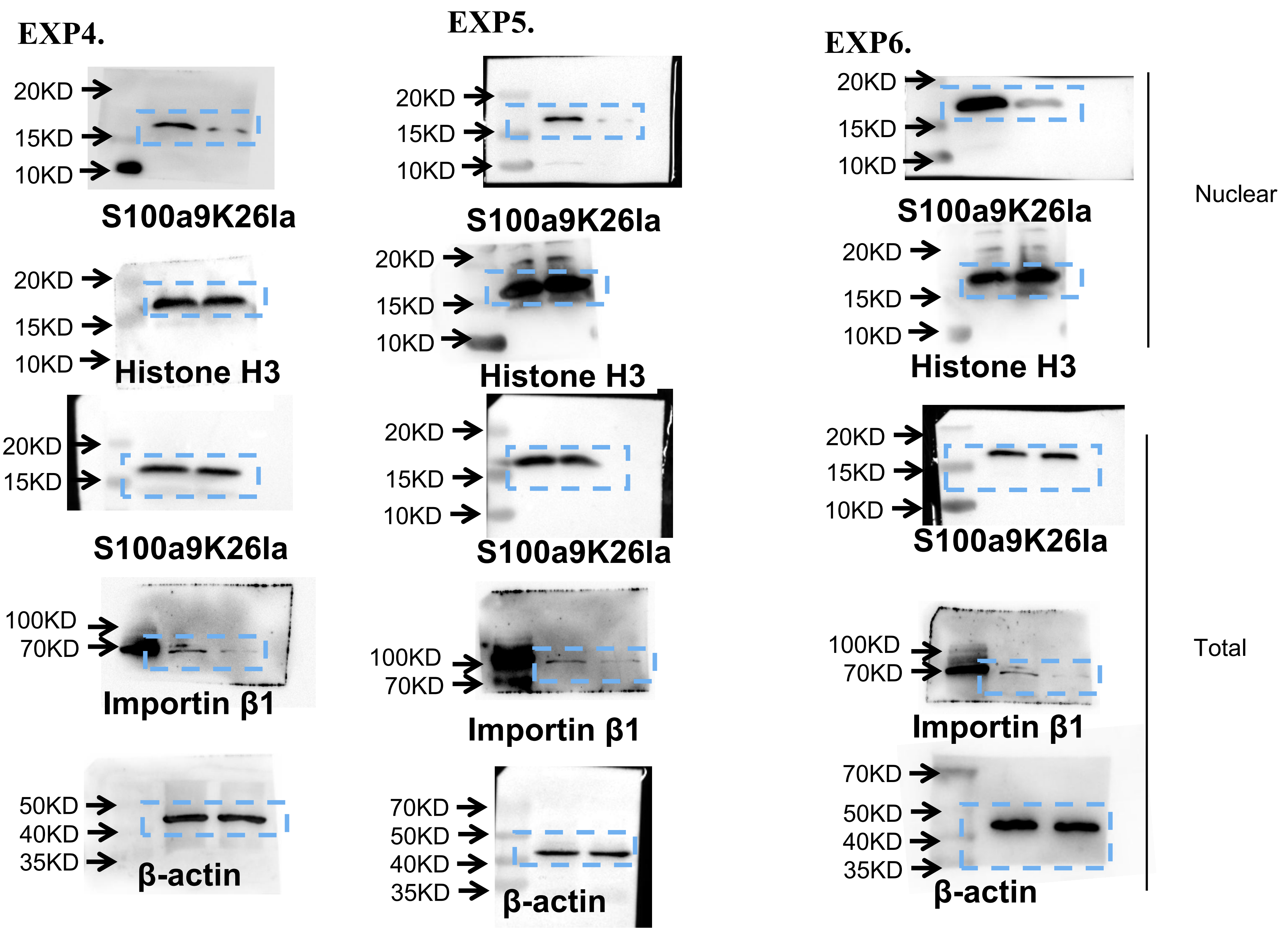

Full unedited gel for Figure S9F

Representative images(EXP1.) Quantification images(EXP1-4.)

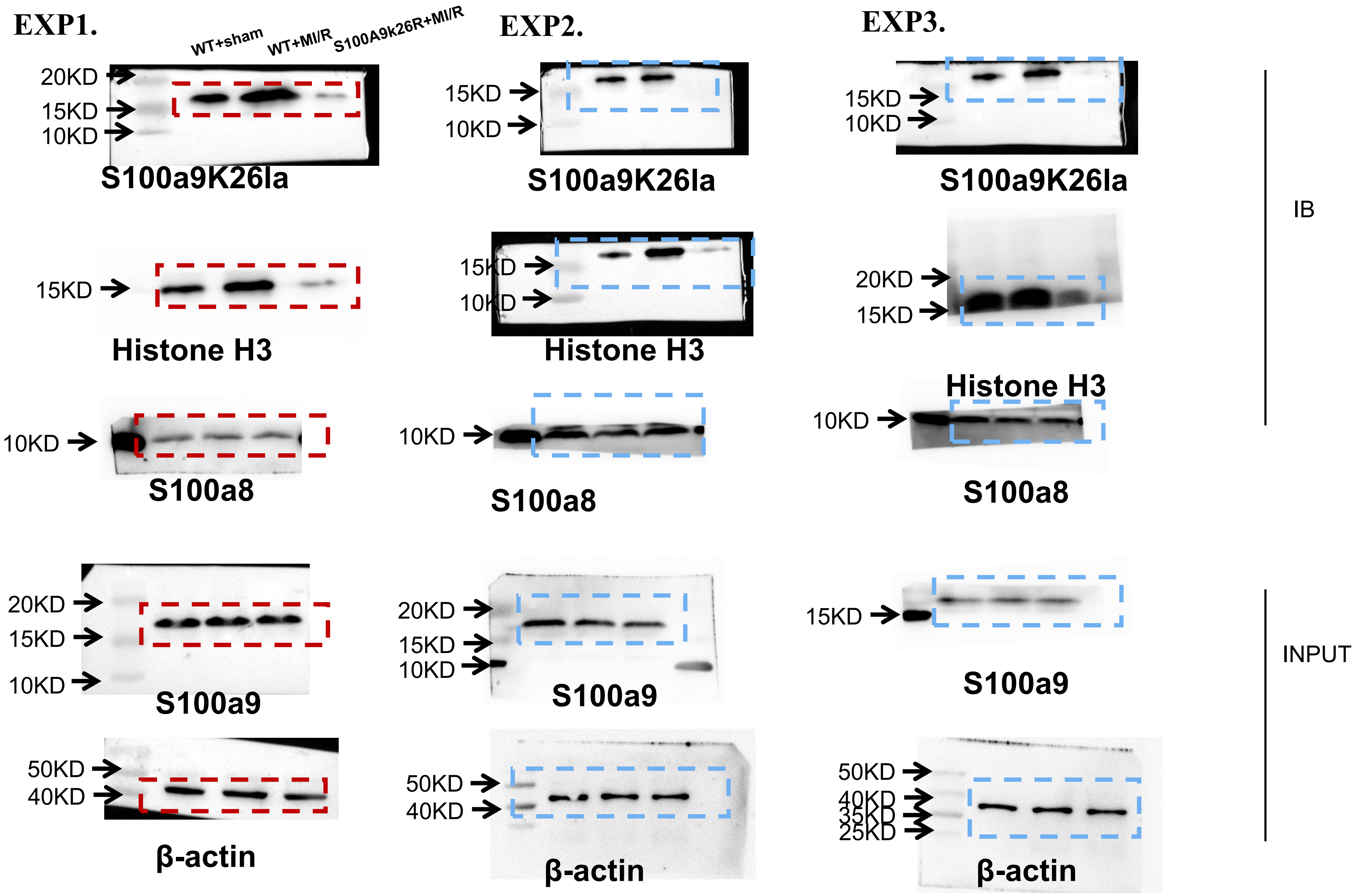

Full unedited gel for Figure S9F

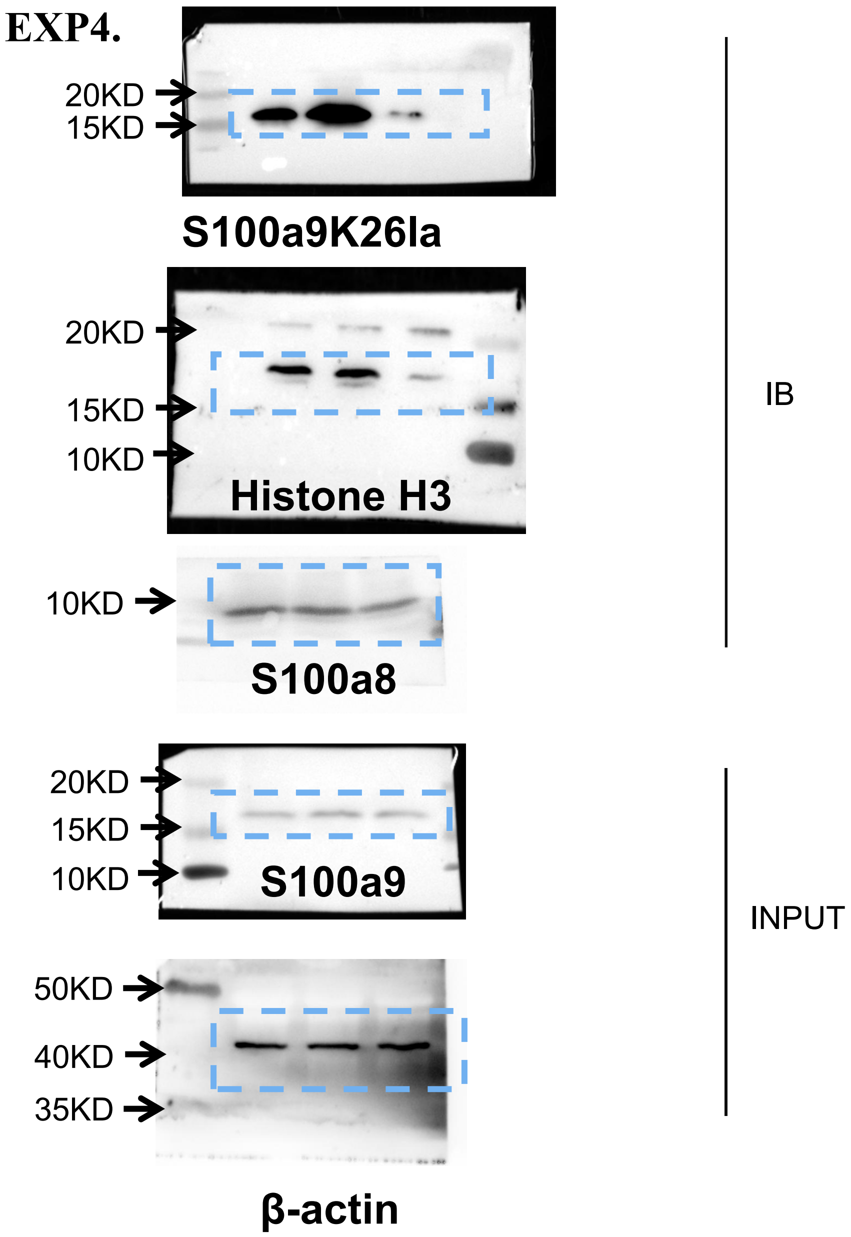

Full unedited gel for Figure S11C

Representative images(EXP1.)   Quantification images(EXP1-6.)

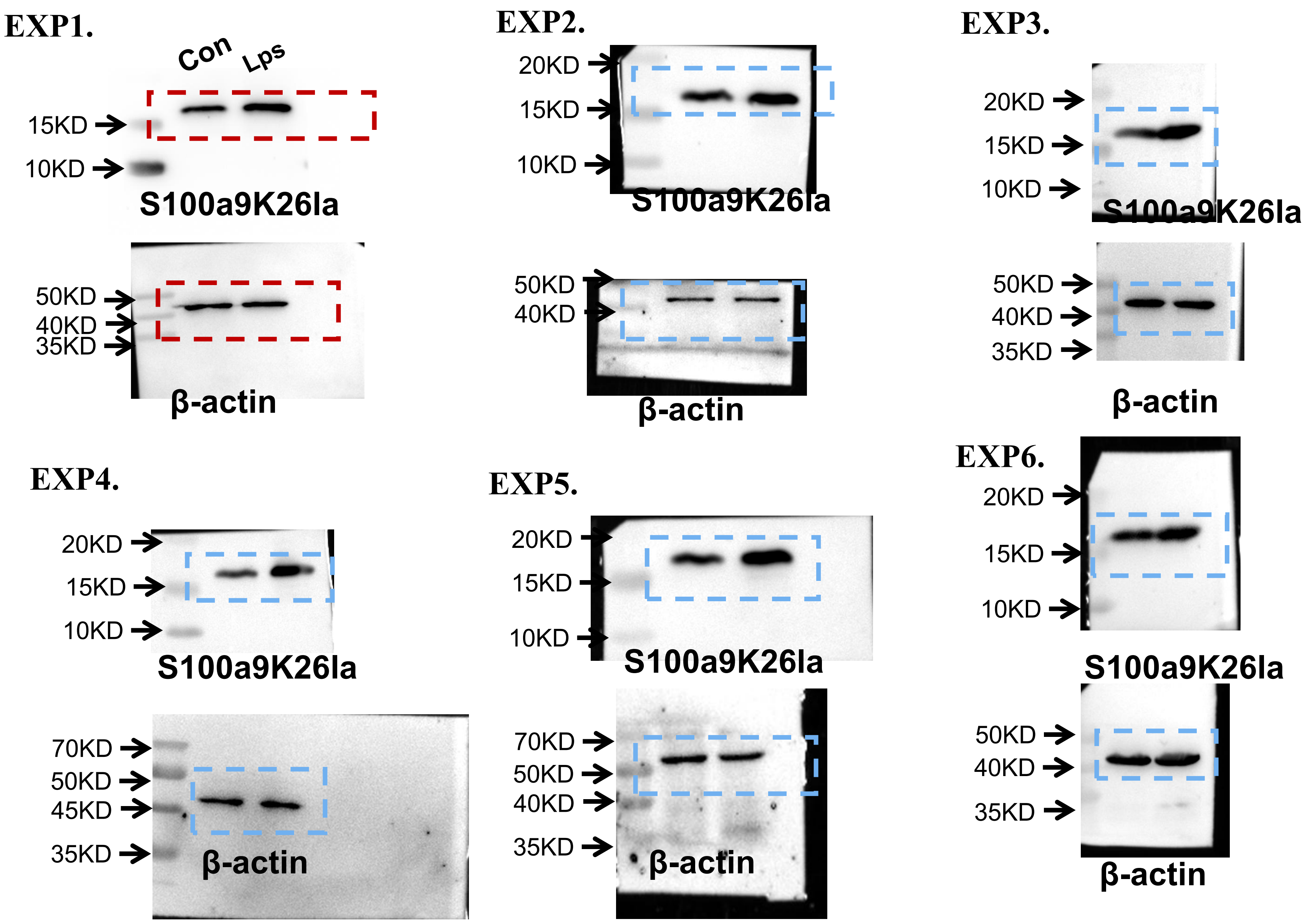

Full unedited gel for Figure S11D

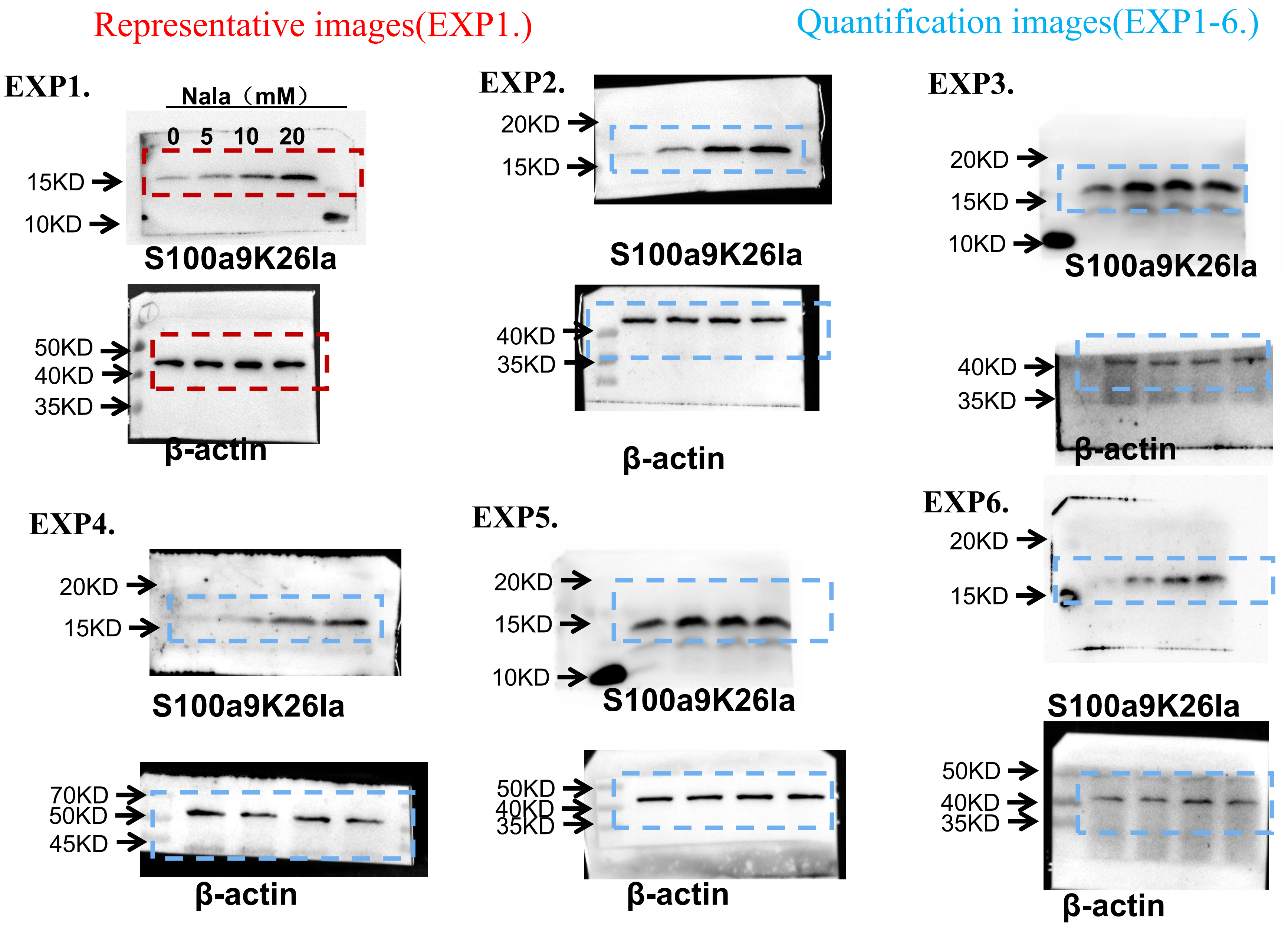

Full unedited gel for Figure S11H

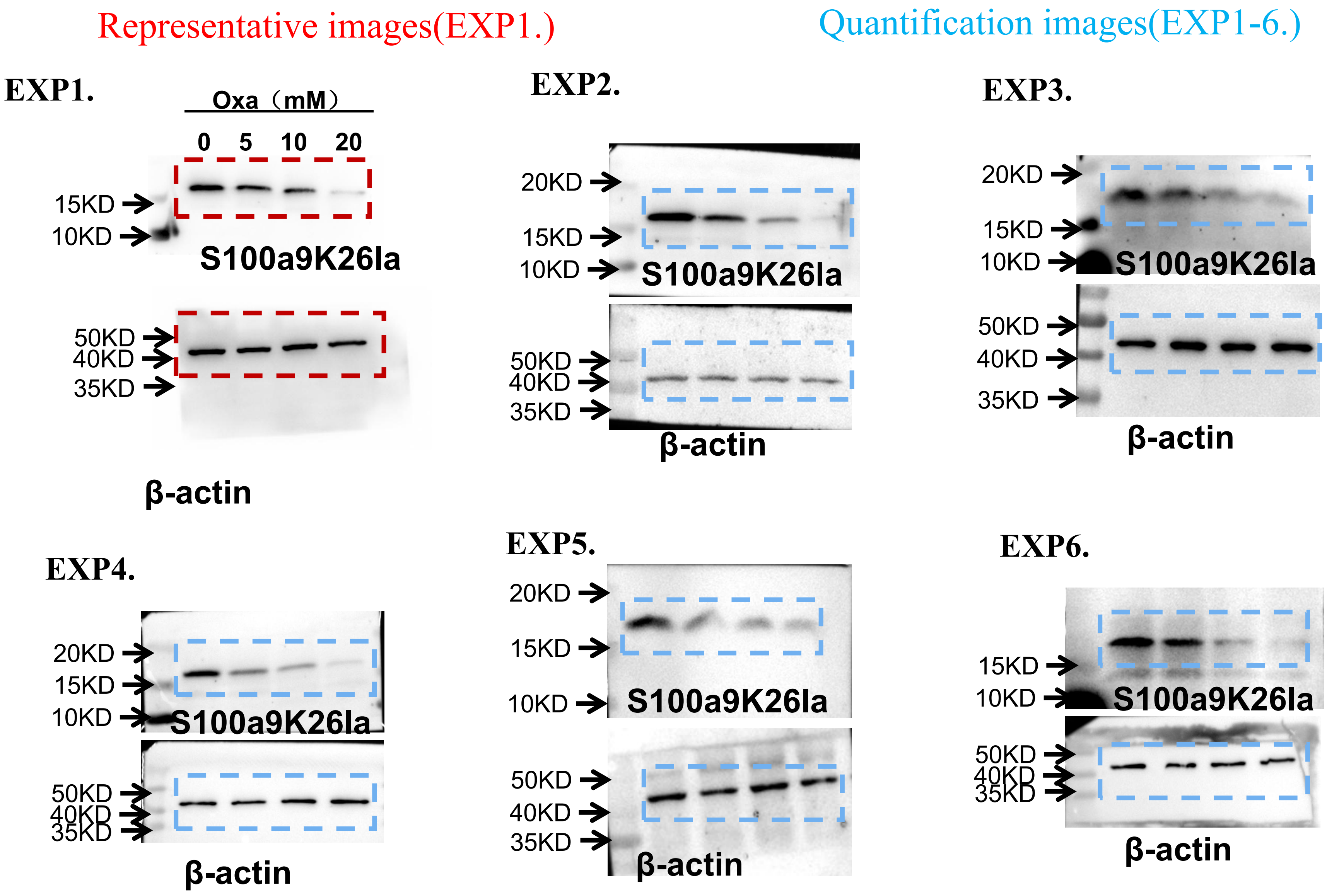

Full unedited gel for Figure S13A

Representative images(EXP1.)

Quantification images(EXP1-4.)

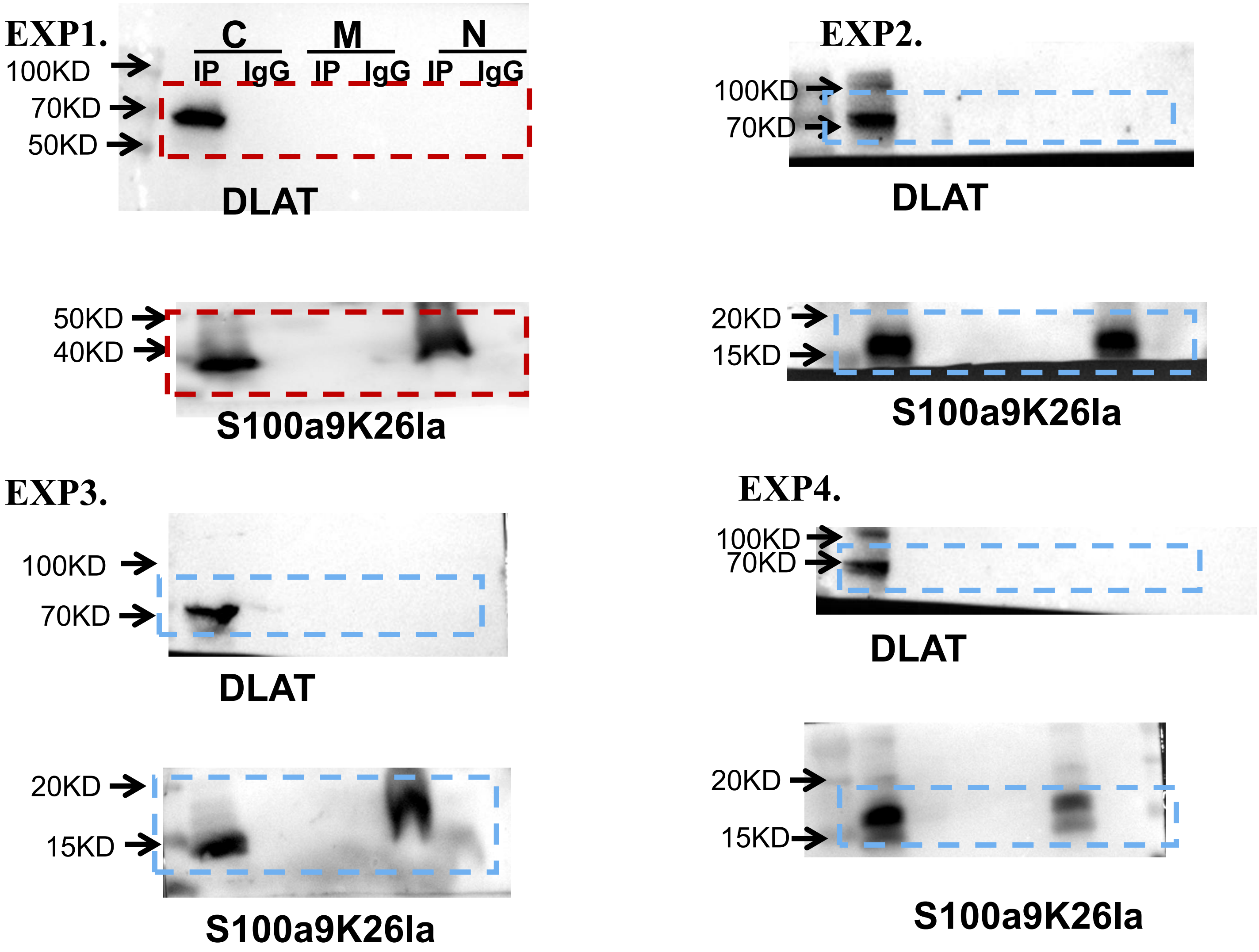

Full unedited gel for Figure S13A

Representative images(EXP1.)

Quantification images(EXP1-4.)

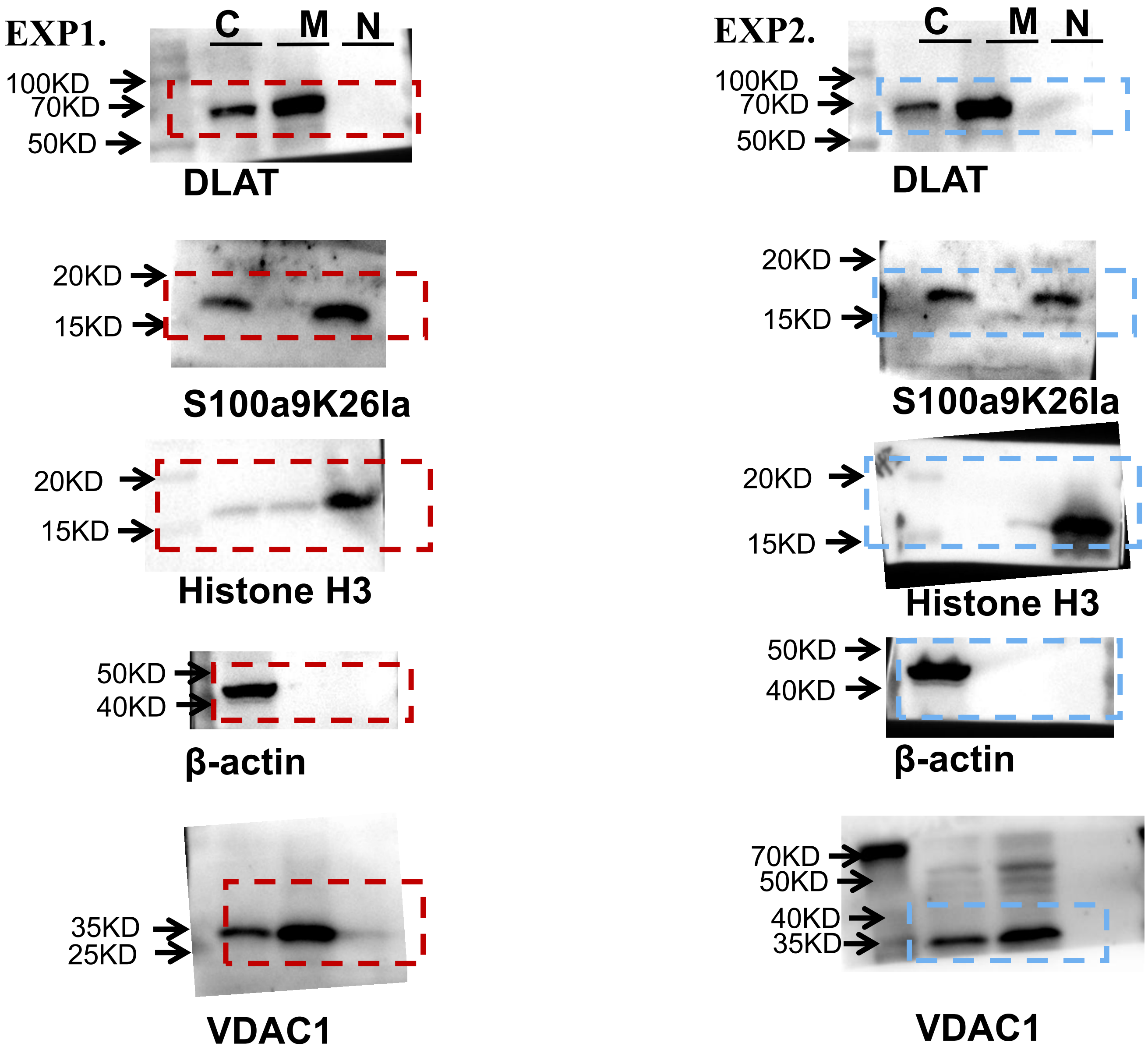

Full unedited gel for Figure S13A

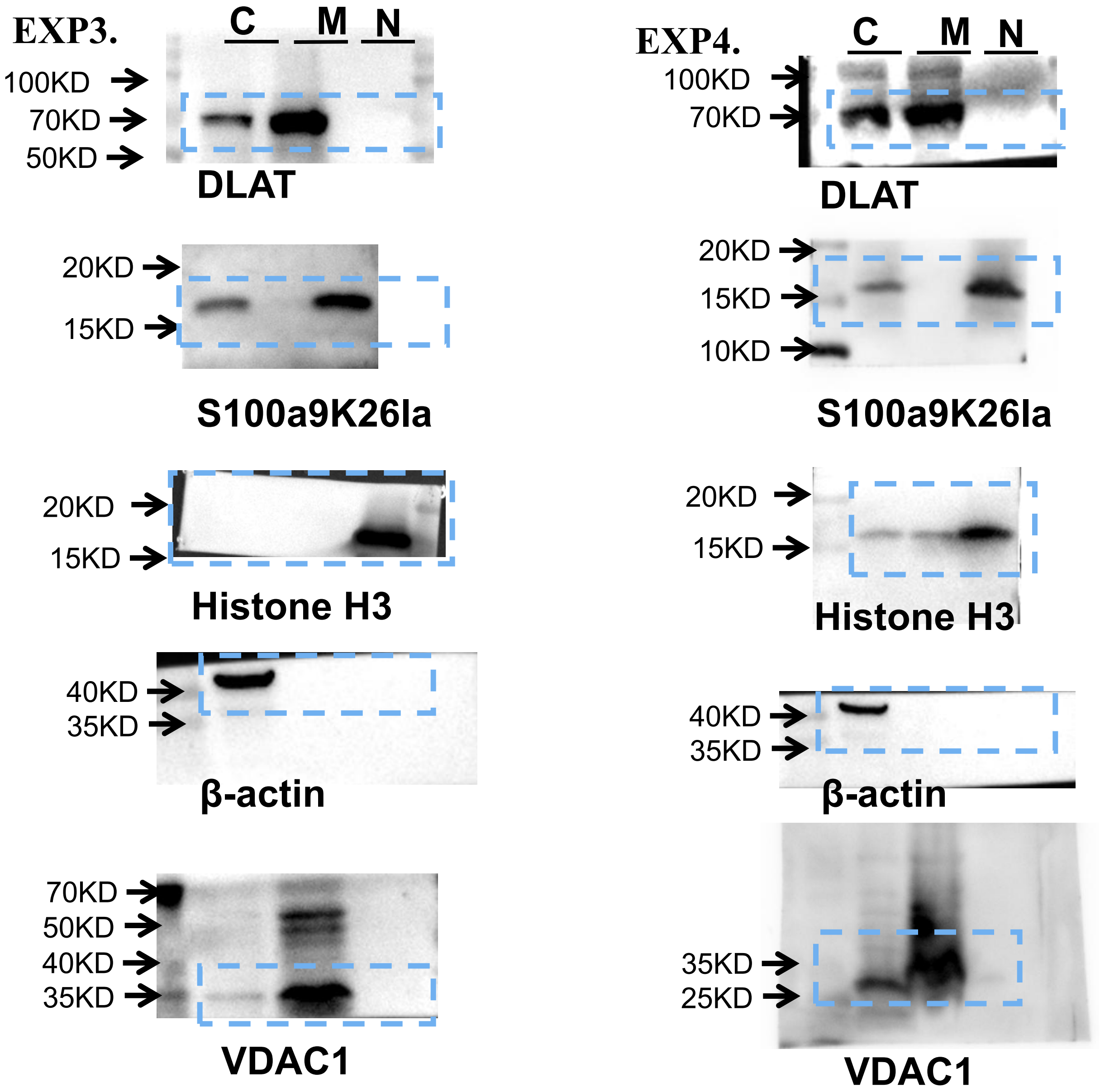

Full unedited gel for FigureS14

Representative images(EXP1.)

Quantification images(EXP1-6.)

EXP1.

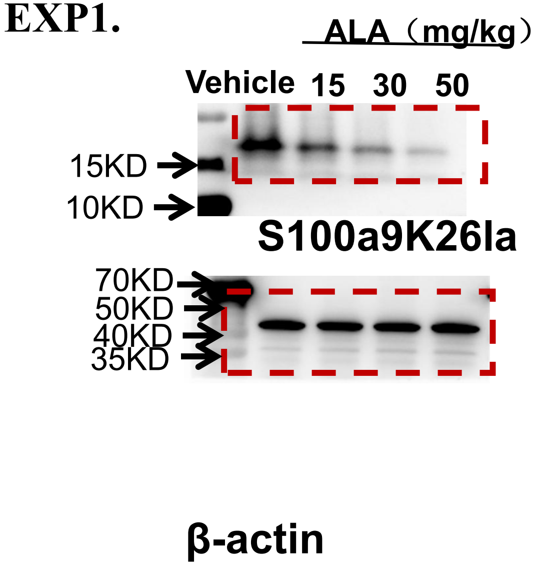

EXP2.

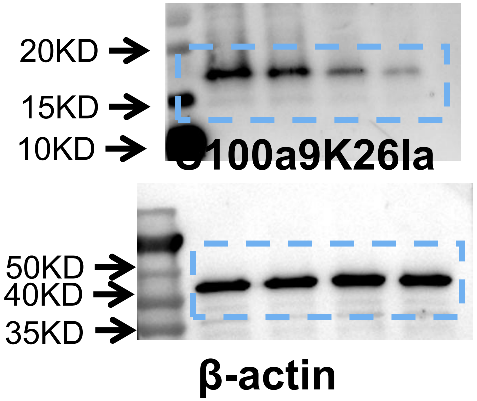

EXP3.

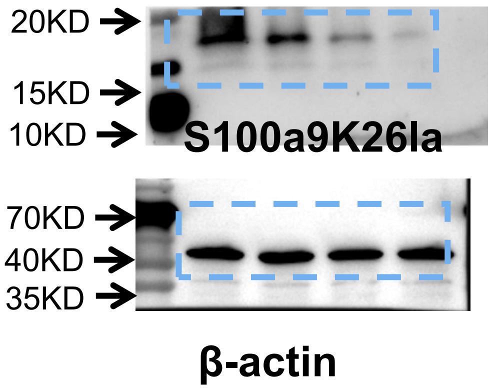

EXP4.

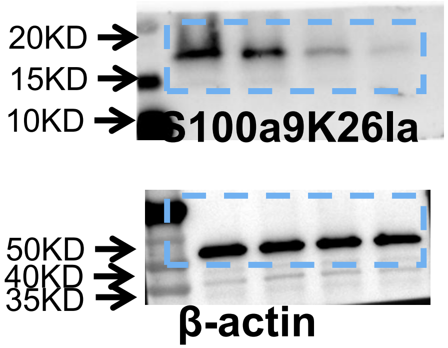

EXP5.

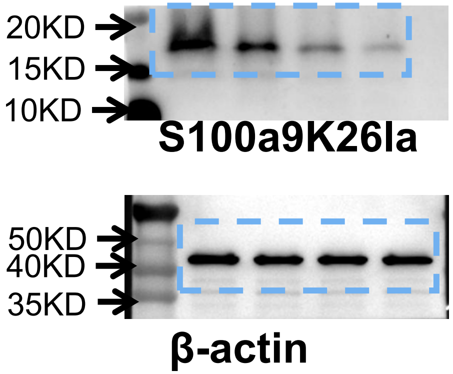

Full unedited gel for Figure S15B

Representative images(EXP1#-4#)

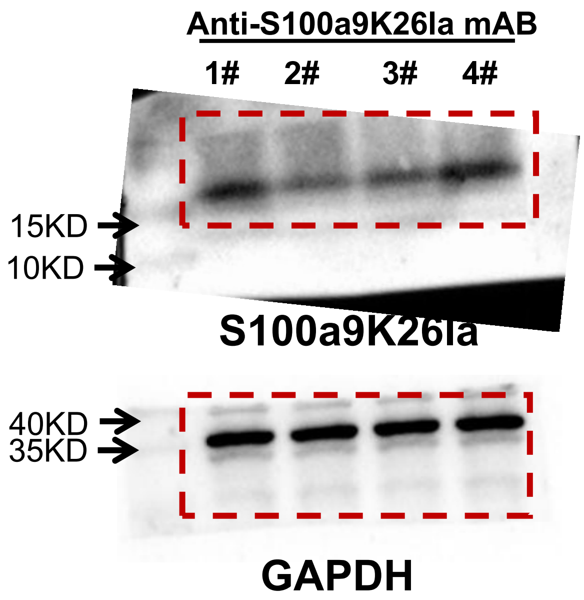

Quantification images(EXP5#-8#)

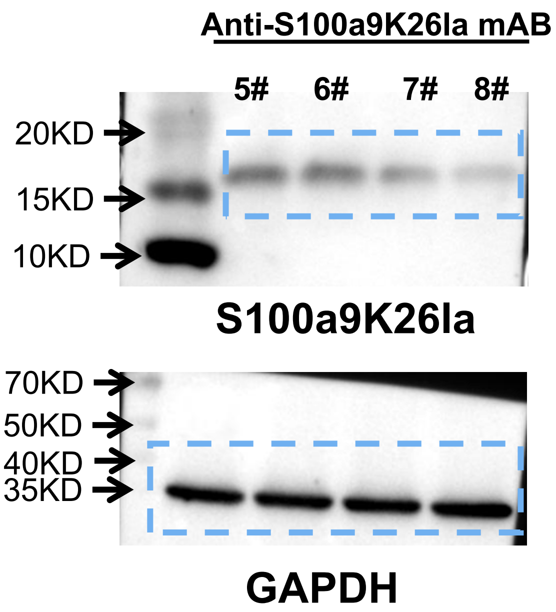

Supplement: Unedited blot and gel images [file jci-135-194664-s187.pdf]
